# Consumer attitudes towards dietary behaviors: a mediator between socioeconomic status and diet quality in European adults

**DOI:** 10.1007/s00394-025-03645-6

**Published:** 2025-03-19

**Authors:** Urte Klink, Timm Intemann, Leonie H. Bogl, Lauren Lissner, Wencke Gwozdz, Stefaan De Henauw, Dénes Molnár, Artur Mazur, Luis A. Moreno, Valeria Pala, Paola Russo, Michael Tornaritis, Toomas Veidebaum, Garrath Williams, Antje Hebestreit, Benjamin Schüz, the I.Family consortium

**Affiliations:** 1 Department of Prevention and Health Promotion, Institute for Public Health and Nursing Research, University of Bremen, 28359 Bremen, Germany; 2 Leibniz Institute for Prevention Research and Epidemiology, BIPS, 28359 Bremen, Germany; 3 School of Health Professions, Bern University of Applied Sciences, 3008 Bern, Switzerland; 4 Finnish Institute of Molecular Medicine, University of Helsinki, 00100 Helsinki, Finland; 5 Department of Public Health and Community Medicine, Institute of Medicine, Sahlgrenska Academy, University of Gothenburg, 413 90 Gothenburg, Sweden; 6 Department of Consumer Research, Communication and Food Sociology, Justus-Liebig-University Giessen, 35390 Giessen, Germany; 7 Department of Public Health and Primary Care, Ghent University, 9000 Ghent, Belgium; 8 Department of Pediatrics, Medical School, University of Pécs, 7624 Pécs, Hungary; 9 Institute of Medical Sciences, Medical College, University of Rzeszów, 35-959, Rzeszów, Poland; 10 GENUD (Growth Exercise, Nutrition and Development) Research Group, Instituto Agroalimentario de Aragón (IA2), Instituto de Investigación Sanitaria Aragón (IIS Aragón), Centro de Investigación Biomédica en Red Fisiopatología de la Obesidad y Nutrición (CIBERObn), University of Zaragoza, 50009 Saragossa, Spain; 11 Department of Preventive and Predictive Medicine, Fondazione IRCCS, Istituto Nazionale dei Tumori, 20133 Milan, Italy; 12 Institute of Food Sciences, National Research Council, Avellino, Italy; 13 Education Institute of Child Health, Strovolos, Cyprus; 14 Center of Health and Behavioral Science, National Institute for Health Development, 11619 Tallinn, Estonia; 15 Department of Politics, Philosophy and Religion, Lancaster University, Lancaster, UK

**Keywords:** Socioeconomic factors, Healthy diet, Attitude, Consumer Behavior, Mediation analysis

## Abstract

**Background:**

Socioeconomic disparities in dietary behaviors are well-known, but underlying mechanisms are not fully understood. We investigated whether consumer attitudes toward dietary behaviors mediate the relationship between socioeconomic factors and diet quality.

**Methods:**

This analysis included 4051 adult participants from eight European countries of the I.Family study (2013/2014). Dietary intake was assessed using a food frequency questionnaire, and diet quality was measured by adherence to dietary recommendations using the Healthy Dietary Adherence Score. Socioeconomic factors included education, income, and social vulnerabilities (migrant background, experiencing unemployment in the household, single parenthood). Structural equation modeling was used to model pathways between predictors, outcome, and mediators, which also allowed for accounting of the clustered study design, incorporating random intercepts for country.

**Results:**

Education and income were positively, and unemployment was negatively associated with diet quality. Attitudes reflecting favorable dietary behaviors were positively associated with diet quality, while unfavorable attitudes were inversely associated. Analysis of the path between socioeconomic factors and attitudes revealed a heterogeneous association pattern. Trusting food advertisements and frequently using ready-to-eat foods partially mediated the association between education, income, and diet quality. The association between single parenthood and diet quality was fully mediated by comparing food labels, valuing organic products, and using ready-to-eat foods.

**Conclusion:**

Our findings suggest a mediating role of consumer attitudes in the association between socioeconomic factors and diet quality, but results were not consistent across socioeconomic factors. Our findings may inform the development of interventions and regulations promoting healthy diet, such as restricting food advertisments.

**Supplementary Information:**

The online version contains supplementary material available at 10.1007/s00394-025-03645-6.

## Introduction

Dietary behaviors and diet quality are socially patterned [1, 2]. Individuals with a higher socioeconomic status (SES) generally show healthier dietary behaviors, whereas those with a lower SES are prone to poorer quality diets [3, 4]. Such differences are likely to contribute to a social gradient of diet-related diseases [5, 6]. However, the underlying mechanisms explaining these inequalities in dietary behaviors are still not fully understood.

Social vulnerability includes factors such as having a migrant background, living in a household experiencing unemployment, and being a single parent. These factors may influence various dimensions of individuals’ lives (and their social standing), including access to resources, time constraints, social support, and health cognitions, which can ultimately shape dietary behaviors and health status. These dimensions, often overlooked in studies on SES and diet quality, are nonetheless crucial and require further exploration for a comprehensive understanding of this relationship. Indeed, it has previously been found that children living in families experiencing these vulnerabilities have poorer dietary behaviors [7, 8].

In contrast to their wealthier and more educated counterparts, individuals with lower income or educational attainment tend to prioritize food costs over health considerations when making dietary decisions [9–11]. Education may enhance an individual’s ability to access and process nutrition-related information and adopt healthy dietary behaviors [12–14]. At the same time, the cost of food and nutritional quality are interrelated, where lower-priced products are often nutritionally deficient and energy-dense [15, 16]. Consequently, individuals with limited financial resources may struggle to prioritize health when purchasing food [9].

Diet quality considers the overall pattern of food consumption, and examining diet quality rather than single foods offers a more nuanced understanding of dietary behaviors [17]. In addition, it has been linked to various health outcomes [18]. However, diet quality is a complex phenomenon, and thus, multiple individual, social, and environmental factors likely contribute to socioeconomic inequalities in dietary intake [19, 20]. Previous research suggested that individual-level factors, particularly psychosocial variables such as knowledge, beliefs, and attitudes about dietary behaviors, may mediate the relationship between SES and diet quality. In particular, higher SES correlates with better nutrition knowledge [21–23], more positive attitudes toward healthy eating [24, 25], higher nutritional awareness [26], and health considerations related to food purchasing [21], which in turn are related to better dietary behaviors.

Examining the mediating role of consumer attitudes towards dietary behaviors could improve our understanding of the mechanisms underlying socioeconomic inequalities in dietary intake and could have implications for future interventions. While socioeconomic differences are hardly modifiable, psychological factors such as attitudes could be altered through interventions. Consumer attitudes describe individual and highly subjective perceptions of consequences between dietary behaviors and their affective, instrumental, or social outcomes—for example, a person might expect their consumption of plant-based foods to improve their cardiovascular health [27, 28], whereas another person might expect this to improve their social standing [28]. The content and strength of these perceptions, in turn, are related to dietary behaviors, for example, more sustainable diets [29].

In order to identify and protect vulnerable groups, different socioeconomic factors determining the diet quality need to be compared systematically. In particular, whether consumer attitudes mediate SES differences in dietary behaviors has not yet been examined across European countries.

Therefore, this study uses the adult data from the I.Family study [30] to investigate whether consumer attitudes toward dietary behaviors potentially mediate the relationship between socioeconomic factors and diet quality. We hypothesize that socioeconomic factors, including household income, level of education, and social vulnerabilities indirectly affect diet quality through consumer attitudes. I.Family is a comprehensive follow-up study of the IDEFICS study focusing on the etiology of diet and lifestyle-related diseases among children, adolescents, and their parents in eight European countries.

## Methods

### Study population

The I.Family study is a multi-center, population-based follow-up study of the original IDEFICS study [30, 31]. While only children participated in the IDEFICS study, the aim of the I.Family study was to investige whole families [30]. Its primary focus was to cross-sectionally explore associations between dietary and lifestyle factors contributing to overweight and obesity in children, adolescents, and their parents in eight European countries (Belgium, Cyprus, Estonia, Germany, Hungary, Italy, Spain, and Sweden) [30]. Therefore, in 2013 and 2014, all children who had participated in the IDEFICS study, along with their siblings and parents, were invited to join the I.Family study, which served as the third follow-up of the original IDEFICS participants.The examination involved 7123 of the 16 228 children who initially participated in the IDEFICS study, along with their parents (n = 7527) and siblings (n = 3557) [30]. Children in the IDEFICS study were recruited through kindergarten and school settings in two comparable regions per country, focusing on feasibility rather than representativeness in recruitment process [31]. In the I.Family study, parents provided sociodemographic, behavioral, medical, nutritional, and lifestyle information for themselves and their families [30]. For further details on the IDEFICS and I.Family cohort studies, please refer to the publications by Ahrens et al. [30, 31].

The present analysis, however, includes only adults participating in the I.Family study who had answered all questions regarding consumer attitudes towards dietary behaviors, for whom dietary data was available and therefore the HDAS could be calculated, and who had no missing values for any covariates. Data of children and adolescents were not included in this analysis.

All centers obtained ethical approval from their respective local institutional review board. All adults gave written informed consent for all examinations, collection of samples, subsequent analysis, and the storage of personal data and collected samples [30].

### Measures

All measures in the present investigation were assessed using standardized and harmonized protocols across all countries. Initially developed in English, questionnaires were translated into local languages, followed by back-translation to avoid translation errors. Participants filled out questionnaires at home. Additional information about the overall study design, instruments, and survey characteristics is available elsewhere [30].

#### Socioeconomic factors

To indicate educational attainment, we used the *highest level of education* of the adults or partners living in a household. This was assessed using country-specific reply categories, which were then recoded according to the International Standard Classification of Education (ISCED) [32]. Thus, parental education was collapsed into three categories: “low” (ISCED level 0–2), “medium” (ISCED level 3–5), and “high” (ISCED level 6–8). *Household income* was assessed using nine country-specific categories, based on the median equivalent income in local currency. These categories were derived from national income distributions to ensure comparability across countries. The nine income categories were then collapsed into five categories: low (combining categories 1 and 2), low-medium (category 3), medium (4, 5 and 6), medium–high (category 7), and high (categories 8 and 9). The total amount was equalized to the number of household members using the Organization for Economic Co-operation and Development’s square root scale [33].

Additional factors related to social standing were explored to identify socially vulnerable groups. A *migrant background* was determined if one or both parents of the study participant were born in a (European or non-European) country different from where the study took place. *Unemployment in the household* was defined as one or both of the adults currently unemployed or living on social assistance or welfare. *Single parenthood* was indicated if only one adult lived in the household.

#### Diet quality

To evaluate diet quality, the HDAS was calculated using a food frequency questionnaire (FFQ). Participants filled out the questionnaire at home by reporting the consumption frequency of the food groups included in the questionnaire during a typical week over the preceding month. The FFQ, established as a reproducible and valid assessment tool [34, 35], comprises 43 Pan-European food items categorized into 14 food groups based on their nutritional profiles [36].

The HDAS was developed according to the recommendations for construction of diet quality scores by Waijers et al. [37] and Ocké [38], and was designed specifically for the IDEFICS study [39]. This reflects common dietary recommendations across all eight countries in the IDEFICS study, including limiting the intake of refined sugars, reducing fat intake, especially of saturated fat, choosing whole meal when possible, consuming 400–500 g of fruits and vegetables daily, and fish 2–3 times per week. Hence, the HDAS considers five components: sugar, fat, whole meal, fruits and vegetables, and fish. For the computation of the HDAS, the intake frequency of sugary foods, low-fat foods, whole meal foods, fruits and vegetables, and fish was considered as the total consumption frequency or as the percentage of all consumed food items. The score of each component ranges from 0 to 10. Correspondingly, the HDAS is calculated as the sum of the components’ score ranging from 0 to 50, with the maximum score indicating the highest possible adherence to the dietary guidelines. For a detailed description of the HDAS and its calculation, see Arvidsson et al. (2017) [39].

#### Consumer attitudes toward dietary behaviors

One legal guardian was asked to answer questions regarding consumer attitudes towards dietary behaviors of the family. Specifically, they were asked to answer the following items on a 5-point scale with 1 “disagree”, 2 “disagree moderately”, 3 “unsure”, 4 “agree moderately”, and 5 “agree”: (1) “I compare labels to select the most nutritious food,” (2) “I have more confidence in food products that I have seen advertised than in unadvertised products,” (3) “I try to avoid food products with additives,” (4) “I make a point of using natural or ecological products,” (5) “I prefer to buy meat and vegetables fresh rather than pre-packed,” (6) “I frequently use ready-to-eat foods in our household,” (7) “I frequently use mixes, for instance, baking mixes and powder soups”, and (8) “The kids help in the kitchen, e.g., they peel the potatoes and cut the vegetables.”

For participants from Belgium and Spain, the answer categories for all questions regarding consumer attitudes were in reversed order and read as follows: 1 “agree”, 2 “agree moderately”, 3 “unsure”, 4 “disagree moderately”, and 5 “disagree”. Therefore, for the analysis, these categories were recoded to align with those of the other countries.

To improve readability of the text, the following abbreviated terms will be used henceforth: (1) “comparing food labels,” (2) “trusting food advertisements,” (3) “avoiding food additives,” (4) “valuing organic products,” (5) “preferring fresh meat and vegetables,” (6) “frequently using ready-to-eat foods,” (7) “frequently using pre-made mixes,” and (8) “having children help in the kitchen.”

#### Covariates

Sex, age, and BMI were included in the analysis as covariates. Age and sex were assessed as single items in the questionnaire. Body weight was assessed using a body composition analyzer (Tanita BC 420 SMA, Tanita Europe GmbH, Sindelfingen, Germany) to the nearest 0.1 kg. Participants were weighed without shoes and wearing light clothing. Height was determined with a calibrated stadiometer (Seca 225, Seca GmbH & Co., KG., Hamburg, Germany) and recorded to the nearest 0.1 cm. BMI was calculated by dividing body weight (kg) by the square of height (m^2^) [30].

### Statistical analysis

All analyses were performed in R (version 4.3.2). Descriptive statistics are presented as mean and standard deviation (SD) for HDAS, BMI, age and percentage for categorical variables. Additionally, the proportion of affirmative responses (answers: “agree” and “agree moderately”) to consumer attitudes is provided. Descriptive statistics are presented for each country and the overall study population.

Structural equation modeling (SEM) was used to model pathways between predictors, outcome and mediators, which also allowed for accounting for the clustered study design, incorporating random intercepts for country. Modeling was conducted using the lavaan package (version 0.6–16) in R [40]. SEM enables the simultaneous calculation of direct and indirect effects estimates. All models were adjusted for sex, age, and BMI. As we conducted multiple tests (multiple consumer attitudes as potential mediators per SES dimension), we applied Bonferroni correction with k = 8 and adjusted the local significance level to 0.006.

Figure 1 here.

**Fig. 1:**
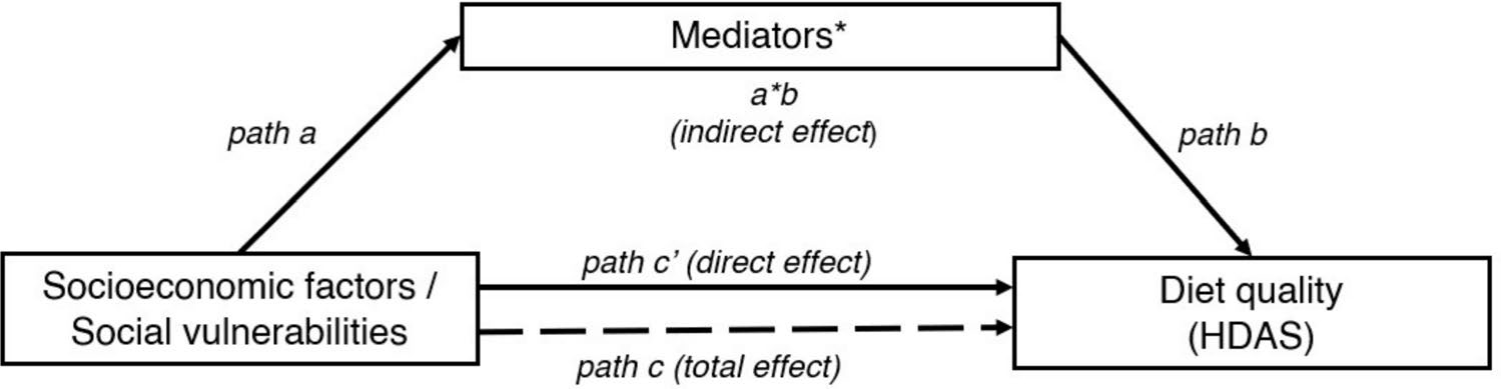
Mediation model. *The mediators included in the mediation models were: comparing food labels, trusting food advertisements, avoiding food additives, valuing organic products, preferring fresh meat and vegetables, frequently using ready-to-eat foods, frequently using pre-made mixes, and having children help in the kitchen. All paths were adjusted for sex, age, and BMI

To estimate indirect (mediated) effects [41], in five separate sets of analyses for each factor of SES, we first regressed diet quality (criterion) on each available socioeconomic factor (predictor). This reflects the total effect (c path in Fig. 1) and was estimated using linear regression. Secondly, path a reflects the association between socioeconomic factors or social vulnerabilities and consumer attitudes (mediators) using ordered logistic regression. Thirdly, path b represents the association between consumer attitudes and diet quality and was assessed via linear regression. The c’ path represents the association between predictors and outcome when adjusted for the mediators and signifies the direct effect. Finally, the indirect effect (a*b) reflects the mediating effect, wherein the predictor impacts the outcome through a specific mediator.

As the answer categories for consumer attitudes were reversed in Belgium and Spain, we performed a sensitivity analysis excluding participants from these countries. These results are presented in the supplementary information.

## Results

### Study participants

As presented in Table 1, a total of 4051 subjects (85.2% female) were included in the analysis. The mean (SD) HDAS of 25.5 (8.8), out of a maximum of 50, suggests a moderate level of diet quality. Most participants lived in households where the highest level of education was either medium (41.9%) or high (50.7%). Most households reported a medium level of income (33.5%). Concerning social vulnerabilities, 12.7% of participants had a migrant background, 8.8% experienced unemployment in their household at the time of data collection, and 10.2% were single parents.

**Table 1:** Sample characteristics by country and overall

	Belgium, N = 118	Cyprus, N = 773	Estonia, N = 595	Germany, N = 509	Hungary, N = 661	Italy, N = 748	Spain, N = 208	Sweden, N = 439	Overall, N = 4051
HDAS Mean (SD)	27.9 (7.2)	25.0 (9.1)	27.7 (7.9)	23.0 (8.9)	23.2 (8.9)	24.3 (8.0)	27.1 (7.8)	30.7 (8.1)	25.5 (8.8)
Highest level of education in household in %									
Low	2.5	1.2	0	6.3	2.1	15.8	2.4	0	4.5
Medium	16.9	36.6	27.4	57.6	47.7	60.6	30.8	23.9	41.9
High	79.7	56.5	71.9	35.6	46.6	19.3	64.9	74.5	50.7
Missing	0.8	5.7	0.7	0.6	3.6	4.4	1.9	1.6	3.0
Household income in %									
Low	2.5	27.2	11.9	16.7	14.1	41.6	6.7	2.3	19.7
Low-medium	2.5	9.7	3.9	10.6	7.0	15.0	6.3	2.7	8.3
Medium	55.1	37.1	24.9	45.8	36.3	21.4	37.5	33.3	33.5
Medium–high	15.3	10.3	9.6	11.6	13.9	1.5	16.3	27.1	11.6
High	18.6	12.5	47.7	9.4	22.5	7.9	27.9	33.3	21.3
Missing	5.9	3.1	2.0	5.9	6.2	12.7	5.3	1.4	5.6
Social vulnerability in %									
Migrant background	3.4	19.7	3.4	19.6	3.2	17.6	7.7	15.7	12.7
Unemployment in household	1.7	15.0	3.7	7.5	7.0	13.9	9.1	2.3	8.8
Single parenthood	11.0	6.7	11.8	15.1	14.5	3.6	9.1	13.2	10.2
BMI (kg/m^2^) mean (SD)	24.1 (4.6)	26.2 (5.1)	25.4 (5.4)	26.7 (5.7)	26.1 (5.6)	27.4 (5.4)	25.1 (4.4)	24.7 (3.9)	26.1 (5.3)
Age (years) mean (SD)	41.2 (4.8)	41.5 (5.9)	39.6 (5.3)	42.9 (5.9)	40.8 (5.1)	42.7 (5.5)	44.9 (4.1)	43.8 (5.3)	41.9 (5.6)
Female in %	87.3	78.3	90.6	88.2	87.9	87.7	83.2	78.6	85.2
Consumer attitudes toward dietary behaviors in %^a^									
Comparing food labels	38.1	70.5	60.0	50.5	46.9	70.2	69.3	60.8	60.5
Trusting food advertisements	7.6	20.7	3.6	5.7	6.5	27.7	15.3	2.1	12.6
Avoiding food additives	53.4	83.3	78	61.3	78.5	78.4	72.1	65.2	74.7
Valuing organic products	48.3	81.4	42.1	29.8	70.2	77.5	75.5	55.3	62.5
Preferring fresh meat and vegetables	82.3	94.3	87.3	70.8	94.4	93.8	94.3	78.6	88.1
Frequently using ready-to-eat-food	9.3	19.9	7.7	10.2	10.4	2.8	24.0	27.8	13.0
Frequently using pre-made mixes	4.2	10.4	6.6	10.0	4.4	3.1	9.2	4.8	6.6
Having children help in the kitchen	53.4	65.6	48.6	59.7	71.7	53.5	66.4	60.8	60.3

*HDAS* Healthy Dietary Adherence Score, *SD* standard deviation, *BMI* body mass index

^a^Proportion of affirmative responses (“agree” and “moderately agree”)

Table 1 here.

Most participants reported comparing food labels (60.5%), and only few trusted food advertisments (12.6%). Many claimed to avoid food products with additives (74.7%). Overall, 62.5% valued organic products. Most participants preferred buying fresh meat and vegetables over pre-packaged options (88.5%). A small number of households frequently used ready-to-eat foods (13%) and pre-made mixes (6.6%). The involvement of children in the kitchen varied, but 60.3% of participants responded affirmatively. Notably, differences between countries could be observed regarding these consumer attitudes (Table S1 in the supplementary information).

### Association between socioeconomic factors and consumer attitudes

**Fig. 2:**
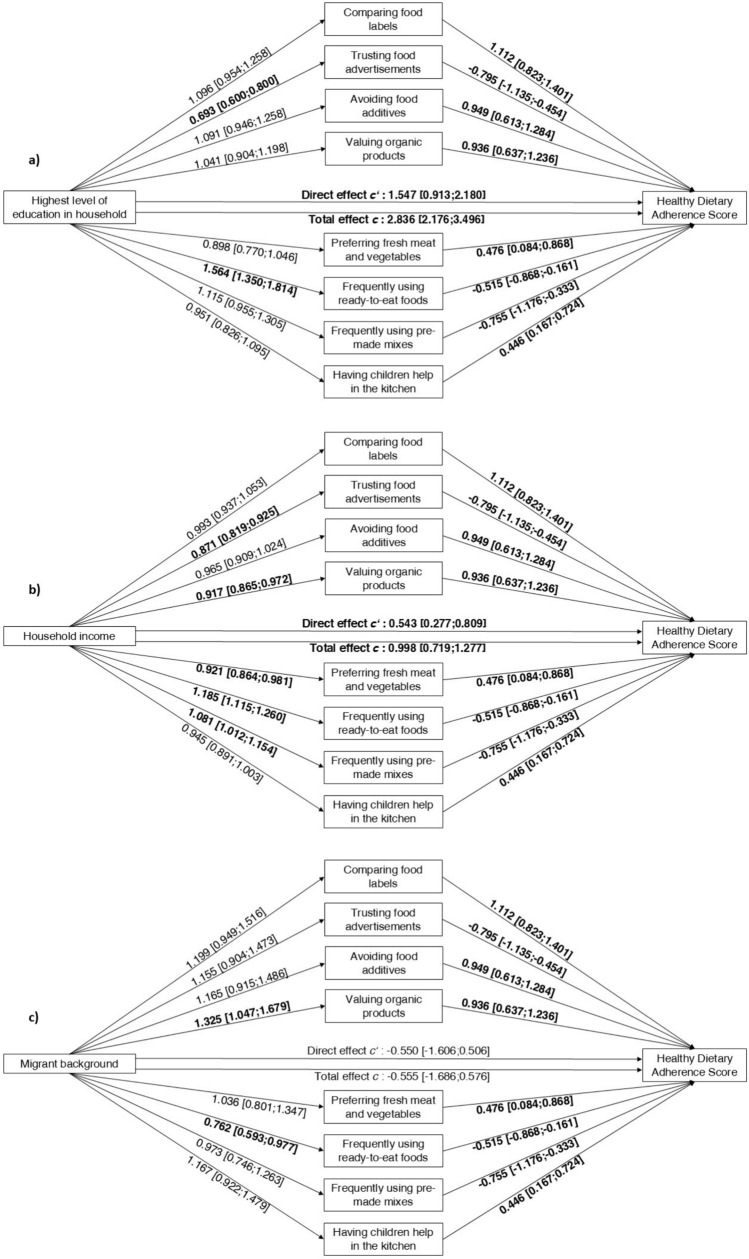

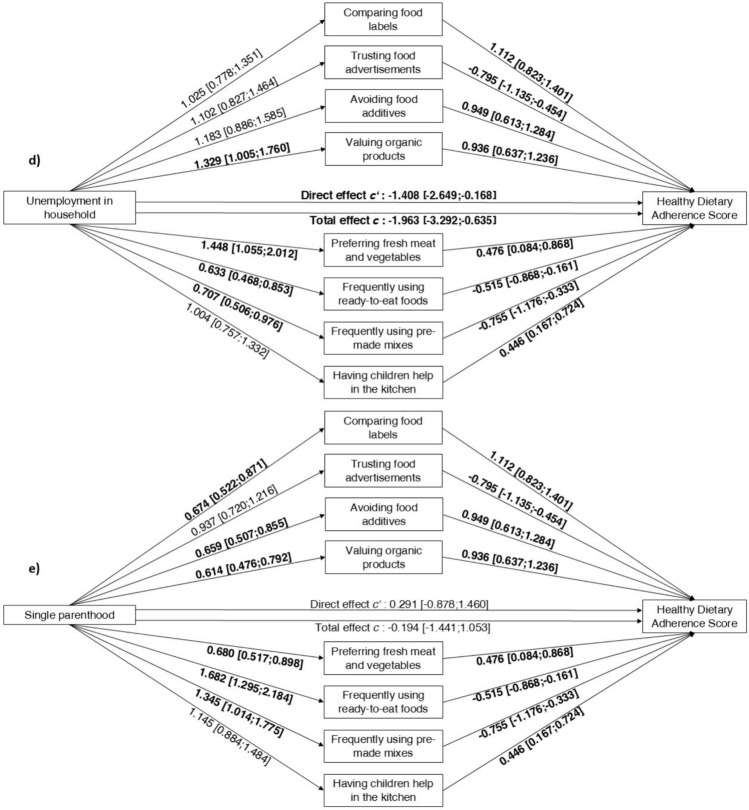
**a**-**e** The mediation of consumer attitudes between socioeconomic factors/social vulnerabilities and Healthy Dietary Adherence Score. The total effect (c-path) shows the association between socioeconomic factors/social vulnerabilities and diet quality without the adjustment for consumer attitudes, while the direct effect (cʹ-path) shows the association after adjustment for consumer attitudes. Path a shows odds ratios and 99.994% confidence interval, paths b, c and cʹ show β estimates and 99.994% confidence interval. All paths are adjusted for sex, age, and BMI

A-path odds ratios in Fig. 2 represent an increase in odds of consumer attitudes associated with each unit increase in the respective socioeconomic factors. Accordingly, those with higher levels of education were less likely to trust food advertisements (OR = 0.693, CI 0.600; 0.800) and were more likely to frequently use ready-to-eat foods (OR = 1.564, CI 1.350; 1.814) (Fig. 2a). Higher household income was negatively associated with trusting food advertisements (OR = 0.871, CI 0.819; 0.925), valuing organic products (OR = 0.917, CI 0.865; 0.972), and preferring fresh meat and vegetables over pre-packed (OR = 0.921, CI 0.864; 0.981), but positively associated with frequently using ready-to-eat foods (OR = 1.185, CI 1.115; 1.260) and pre-made mixes (OR = 1.081, CI 1.012; 1.154) (Fig. 2b). Those with a migrant background placed greater value on organic products (OR = 1.325, CI 1.047; 1.679) and less frequently used ready-to-eat foods (OR = 0.762, CI 0.593; 0.977) (Fig. 2c). Those experiencing unemployment in their household were more likely to value organic products (OR 1 = 0.329, CI 1.005; 1.760) and to prefer fresh meat and vegetables over pre-packed (OR = 1.448, CI 1.055; 2.012), and were less likely to frequently use ready-to-eat foods (OR = 0.633, CI 0.468; 0.853) and pre-made mixes (OR = 0.707, CI 0.506; 0.976) (Fig. 2d). Single parents were less likely to compare food labels (OR = 0.674, CI 0.522; 0.871), avoid food additives (OR = 0.659, CI 0.507; 0.855), value organic products (OR = 0.614, CI 0.476; 0.792), and prefer fresh meat and vegetables (OR = 0.680, CI 0.517; 0.898), and were more likely to frequently use ready-to-eat foods (OR = 1.682, CI 1.295; 2.184) and pre-made mixes (OR = 1.345, CI 1.014; 1.775) (Fig. 2e). There were no significant associations between predictors and having children help in the kitchen.

Figure 2 here.

### Association between socioeconomic factors and diet quality

Totel effect/c-path in Fig. 2 shows the association of education, income, migrant background, unemployment in the household, and single parenthood with diet quality. There was a significant positive association between education (β = 2.836, CI 2.176; 3.496) (Fig. 2a) and income (β = 0.998, CI 0.719; 1.277) (Fig. 2b) and diet quality, indicating that those with higher education and income showed higher adherence to dietary recommendations. Furthermore, experiencing unemployment in the family’s household was significantly negatively associated with diet quality (β = – 1.963, CI – 3.292; – 0.635) (Fig. 2d). There was no statistically significant association between migrant background and single parenthood with diet quality (Fig. 2c, e).

### Association between consumer attitudes and diet quality

B-path estimates in Fig. 2 represent the change in the diet quality score for a unit increase in consumer attitudes. Consumer attitudes were associated with diet quality as follows: comparing food labels (β = 1.112, CI 0.823; 1.401), avoiding food additives (β = 0.949, CI 0.613; 1.284), valuing organic products (β = 0.936, CI 0.637; 1.236), preferring fresh meat and vegetables (β = 0.476, CI 0.084; 0.868), and having children help in the kitchen (β = 0.446, CI 0.167; 0.724) were positively associated with diet quality, while trusting food advertisements (β = – 0.795, CI – 1.135; – 0.454), frequently using ready-to-eat foods (β = – 0,515, CI – 0.868; – 0.161) and pre-made mixes (β = 0.755, CI – 1.176; – 0.333) were negatively associated with diet quality (Fig. 2, b-path estimates).

### Attitudes as mediators of the relation between socioeconomic factors and diet quality

We used SEM to analyze whether socioeconomic factors are associated with diet quality and whether the direct path would be mediated by consumer attitudes toward dietary behaviors. Indirect effects (a*b) with Bonferroni-adjusted confidence intervals are presented in Table 2, and Fig. 2 shows the direct effects (c’-path). For both the level of education and household income, we found significant indirect effects for trusting food advertisements (education: β = 0.145, CI 0.048; 0.242, p < 0.001; income: β = 0.056; CI 0.016; 0.095, p < 0.001) and frequently using ready-to-eat-foods (education: β = – 0.122, CI – 0.209; – 0.034, < 0.001; income: β = – 0.038, CI – 0.069; – 0.006, p 0.001) (Fig. 2a, b). As the direct effect was significant for both predictors, their relationship with diet quality is partially mediated by these consumer attitudes. Consequently, in addition to consumer attitudes, other determinants may also play a role. For single parents, we found significant indirect effects for comparing food labels (β = – 0.224, CI – 0.396; – 0.052, p < 0.001), valuing organic products (β = – 0.197, CI – 0.355; – 0.040, p 0.001), and frequently using ready-to-eat-foods (β = – 0.130, CI – 0.251; – 0.009, p 0.003) (Fig. 2e). As the direct effect was not significant, the relationship between single parenthood and diet quality is fully mediated by these consumer attitudes. No significant indirect effects for having a migrant background and experiencing unemployment in a household were found (Table 2).

**Table 2:** Estimates and confidence intervals of multilevel structural equation modelling for the association between socioeconomic factors/social vulnerabilities and Healthy Dietary Adherence Score mediated by consumer attitudes, adjusted for sex, age and BMI

	Indirect effect a*b							
	Comparing food labels	Trusting food advertisements	Avoiding food additives	Valuing organic products	Preferring fresh meat and vegetables	Frequently using ready-to-eat foods	Frequently using pre-made mixes	Having children help in the kitchen
	β [CI]	β [CI]	β [CI]	β [CI]	β [CI]	β [CI]	β [CI]	β [CI]
Highest level of education in household, N = 3931	0.061 [– 0.017; 0.139]	**0.145 [0.048; 0.242]**	0.027 [– 0.017; 0.071]	0.049 [– 0.014; 0.112]	0.003 [– 0.010; 0.015]	**– 0.122 [– 0.209;-0.034]**	0.000 [– 0.001; 0.001]	– 0.009 [– 0.044; 0.026]
Household income, N = 3825	0.002 [– 0.032; 0.035]	**0.056 [0.016; 0.095]**	– 0.001 [– 0.011; 0.009]	– 0.026 [– 0.056; 0.004]	0.002 [– 0.009; 0.013]	**– 0.038 [– 0.069;-0.006]**	– 0.001 [– 0.006; 0.004]	– 0.012 [– 0.029; 0.006]
Migrant background, N = 4051	0.099 [– 0.042; 0.241]	– 0.093 [– 0.196; 0.011]	0.015 [– 0.038; 0.067]	0.104 [– 0.012; 0.220]	0.002 [– 0.013; 0.017]	0.043 [– 0.033; 0.120]	– 0.001 [– 0.009; 0.007]	0.031 [– 0.032; 0.095]
Unemployment in household, N = 4051	0.001 [– 0.158; 0.161]	– 0.064 [– 0.177; 0.049]	0.006 [– 0.051; 0.064]	0.094 [– 0.035; 0.222]	– 0.009 [– 0.052; 0.033]	0.083 [– 0.019; 0.185]	0.005 [– 0.031; 0.041]	– 0.004 [– 0.072; 0.064]
Single parenthood, N = 4035	**– 0.224 [– 0.396;-0.052]**	0.064 [– 0.043; 0.171]	– 0.080 [– 0.187; 0.028]	**– 0.197 [– 0.355;-0.040]**	0.014 [– 0.068; 0.097]	**– 0.130 [– 0.251;-0.009]**	– 0.005 [– 0.051; 0.041]	0.035 [– 0.034; 0.105]

β estimate; [LLCI; ULCI] = lower and upper levels of 99.994% confidence interval of the indirect effect estimate between socioeconomic factors/social vulnerabilities and HDAS

Table 2 here.

## Discussion

This study examined whether consumer attitudes toward dietary behaviors mediate the association between SES and diet quality. Highest level of education in the household and household income were positively associated with diet quality, while experiencing unemployment in one’s household was negatively associated with diet quality. Attitudes reflecting favorable dietary behaviors, including comparing food labels, avoiding food additives, valuing organic products, preferring fresh meat and vegetables, and having children help in the kitchen, were positively related to diet quality. Attitudes reflecting unfavorable dietary behaviors included trusting food advertisements, frequently using ready-to-eat foods, and frequently using pre-made mixes, and were inversely related to diet quality. A heterogeneous pattern of the relationship between socioeconomic factors and consumer attitudes was revealed. The association between education, income, and diet quality was partially mediated by trusting food advertisements and frequently using ready-to-eat foods. Comparing food labels, valuing organic products, and frequently using ready-to-eat foods fully mediated the association between single parenthood and diet quality.

The positive association between education, income, and diet quality found in this study aligns with the findings of other studies conducted in Europe [3, 42, 43]. Additionally, those experiencing unemployment in their household had lower diet quality scores than those who did not, alining with previous findings in Europe [44, 45], and Canada [46]. Several reviews have discussed the relationship between socioeconomic factors and diet quality [1, 47, 48], and generally, those of lower SES present poorer dietary behaviors, such as lower fruit and vegetable consumption [21, 22] and higher consumption of ultra-processed foods [49]. Hence, our findings align with the general consensus that socially disadvantaged groups exhibit poorer dietary behaviors than higher SES groups and vice versa.

As the effect estimate of education on diet quality was greater than the effect estimate of income on diet quality, the association between education and diet quality might be more relevant than the association with income. While income may fluctuate over time, educational attainment is stable, capturing early-life processes that typically endure over time [50]. The relationship between education and diet is believed to mirror an individual’s capacity to access and reflect nutrition-related information [12–14]. Higher levels of education suggest a deeper understanding of dietary and health-related matters, which might lead to dietary choices that align with current dietary recommendations. Indeed, several studies have linked higher educational attainment to better diet quality and enhanced nutrition knowledge [22, 23, 51].

Income data, on the other hand, might be susceptible to errors due to social desirability bias, uncertainty about household income, or reluctance to provide accurate information in surveys [52]. These factors can lead to a less reliable measure, possibly obscuring clear relationships [25], which might have contributed to the comparatively lower influence of income on diet quality observed here.

Here, unemployment was linked to poorer diet quality, likely driven in part by financial constraints. While more time at home might allow for healthier cooking [53], these benefits are often offset by the pressure to save on food costs and, thereby, to choose cheaper, less nutritious food options [15, 46]. Additionally, the impact of unemployment on stress and mental health can negatively influence dietary choices [54].

We observed a positive relationship between diet quality and attitudes reflecting favorable dietary behavior, including comparing food labels, avoiding food additives, valuing organic products, preferring fresh meat and vegetables, and having children help in the kitchen. Two previous reviews [55, 56] have concluded that food label use may have a positive effect on dietary choices, but results differed between subgroups [56], and evidence was described as inconsistent [55]. Food additives, used for various technological purposes during food processing, are usually not consumed on their own and are rarely found in minimally and unprocessed foods [57]. Additionally, as their consumption is associated with an increased risk of non-communicable diseases [49], avoiding additives suggests a preference for minimally processed foods and concern with the nutritional quality of processed foods. Valuing ecologically produced foods can be interpreted as an expression of commitment to sustainable dietary behaviors. Indeed, health consciousness has been associated with purchasing organic foods [58], and Baudry et al. [59] showed that diet quality increases with organic food consumption. Preferring to buy fresh meat and vegetables rather than pre-packed options again may signify a preference for minimally processed, nutrient-rich foods, aligning with health-conscious choices. Indeed, freshness is an important factor consumers use to evaluate food quality related to health [60], and households prioritizing low-convenience foods reported higher awareness of health and sustainability [61]. Having children involved in food preparation has been identified as a potential strategy to improve their dietary behaviors [62], particularly as their involvement presents numerous benefits, including healthier overall diet quality, self-efficacy related to eating healthy foods, and food preparation skills [63]. Here, we showed that having children help in the kitchen was associated with better diet quality, indicating that these parents might be more health-conscious and confident in their cooking skills than those who are not, but further research is needed to explore this link.

Additionally, we observed an inverse relationship between unfavorable attitudes and diet quality, including trusting food advertisements and frequently using ready-to-eat foods and pre-made mixes. It has widely been reported that TV food advertisements predominantly promote products high in sugar, salt, and/or fat (e.g. [64, 65],). A positive association between exposure to food advertising and consumption of energy-dense foods has been reported for adults [66]. Consequently, participants trusting advertisements might be more inclined to purchase the advertised products, which may explain the negative association with diet quality and underpins the need for stronger regulations of food advertisments. Both ready-to-eat foods and pre-made mixes are food products that provide convenience, require little cooking skills, and are typically highly processed foods with low nutritional value [57]. Therefore, a more frequent consumption of these food products may explain the inverse association with diet quality. Indeed, it has previously been shown that higher consumption of ultra-processed foods is associated with lower diet quality [67].

Analysis on the path between SES and attitudes revealed a heterogeneous association pattern. We found that education and income were inversely associated with trusting food advertisements, with the association being stronger for education. This suggests that individuals with higher levels of education may be better equipped to critically evaluate food advertisements. Household income and education were positively associated with frequently using ready-to-eat foods, while income was also positively associated with frequently using pre-made mixes. Although consuming a low-convenience food pattern has previously been associated with higher income and higher educational level in a German cohort [61], their higher consumption in our investigation encompassing eight European countries may also indicate time constraints for meal preparation faced by higher status groups, considering these products’ convenience and short preparation time. We also found a negative association between household income and valuing organic products and preferring fresh meat and vegetables, which suggests a lower priority for high-quality foods among the high-income group and aligns with our findings regarding ready-to-eat foods and pre-made mixes. Having a migrant background and experiencing unemployment were positively associated with valuing organic products and negatively with using ready-to-eat foods. Additionally, unemployment was positively associated with preferring fresh meat and vegetables, indicating a higher preference for minimally processed foods. This appears to contradict previous findings that claim foods with higher nutritional value cost more per calorie and tend to be avoided by disadvantaged groups [15]. Therefore, further investigation is needed.

Single-parent families have historically faced greater economic vulnerability due to limited income and time constraints arising from managing childcare and household responsibilities, while also pursuing employment. These challenges might pressure them to choose energy-dense and processed foods for affordability and convenience, as indicated by a higher risk of obesity in children living in single-parent households [68]. Indeed, in our investigation, single parenthood was negatively associated with comparing food labels, avoiding food additives, valuing organic products, and preferring fresh vegetables and meat, and was positively associated with frequently using ready-to-eat foods and pre-made mixes, which, taken together, suggests a lack of resources and opportunities for health-conscious dietary behaviors in this particular group. However, further research is needed to fully understand possible mechanisms influencing dietary choices within single-parent families.

Our findings support the notion of health behavior theories that cognitions such as attitudes influence health behaviors such as diets (for an overview, see Conner & Norman [69]). More importantly, our findings also align with a central tenet of such theories, namely that factors that are more proximal to behavior, such as attitudes may mediate between more distal factors, such as SES (e.g., [70]). Put simply, this suggests that socioeconomic differences manifest in dietary differences because people hold different ideas about diet. For example, we observed that trusting food advertisements and frequently using ready-to-eat foods partially explain the association between education, income, and diet quality, which means that education and income positively affect diet quality because those with higher educational attainment and higher income trust food advertisements less.

### Strengths and limitations

This study has both strengths and limitations that need to be addressed. The large sample size of the I.Family study, encompassing data from eight European countries and rigorously standardized data collection, documentation, and data cleaning procedures ensure high level of data quality. For the present investigation, we used standardized measures for education and income, two of the most important and most commonly assessed socioeconomic factors, to investigate their predictive potential on consumer attitudes toward dietary behaviors and diet quality. Additionally, we examined the role of social vulnerability, including migrant background, experiencing unemployment in the household, and single parenthood, which has rarely been investigated. The diet quality score used in this investigation is based on dietary recommendations common for the eight European countries included in the IDEFICS/I.Family studies, and therefore, it is based on substantial evidence of health effects underlying the food and beverages included. The FFQ used to assess dietary intakes has been validated and found to be reproducible [34, 35]; however, potential bias due to social desirability in dietary self-reporting remains.

The main limitation is the nature of our study sample, which is not representative of the European population or of each included country. In addition, the sample consists exclusively of parents, and only a minority of fathers (< 15%) were included in our investigation, thereby introducing gender bias, and consequently, results may not reflect broader population views. Participants were predominantly of higher SES compared to other cross-sectional studies, and as parents with a strong interest in health remained in the cohort by the third wave, this may have attenuated possible effects. However, our findings align with similar findings in other European populations. Our assessme nt of attitudes mainly focused on behavioral aspects, items assessing affective and cognitive aspects of attitudes might have revealed different patterns. The response options for assessing consumer attitudes were reversed in Belgium and Spain compared to the other participating countries. While this may be a potential limitation, experiments suggest that the order of response options is unlikely to influence participant responses [71]. However, to assess the potential impact of this inconsistency, we conducted a sensitivity analysis (see supplemental information) by excluding participants from Belgium and Spain, and we only found slight deviations, while the overall results pattern was upheld.

## Conclusion

Our findings suggest a mediating role of consumer attitudes in the association between socioeconomic factors and diet quality. In particular, we showed that comparing food labels, trusting food advertisements, putting value in organic products, and using convenience foods may mediate this relationship; however, results were not consistent across socioeconomic factors. This has implications for interventions and health promotion programs that promote healthy dietary behaviors. While directly modifying socioeconomic factors is undoubtedly challenging, interventions targeting cognitive and behavioral aspects may be an effective way to improve dietary behaviors. Additionally, our findings highlight the potential of nutrition and consumer education programs to help individuals adopt healthier dietary behaviors and make informed consumer choices.

## Supplementary Information

Below is the link to the electronic supplementary material.

Supplementary file1 (DOCX 1064 KB)

Supplementary file2 (HTML 1056 KB)

Supplementary file3 (HTML 1047 KB)
