# Supplementary material for: Consumer attitudes towards dietary behaviors: a mediator between socioeconomic status and diet quality in European adults
Source: Eur J Nutr. 2025 Mar 19;64(3):127. doi: 10.1007/s00394-025-03645-6 (PMC11922978; doi:10.1007/s00394-025-03645-6)
Supplement: Supplementary file 1 — Supplementary file1 (DOCX 1064 KB) [file 394_2025_3645_MOESM1_ESM.docx]

**Consumer attitudes towards dietary behaviors: a mediator between socioeconomic status and diet quality in European adults**

Urte Klink^1*^ [ORCID: 0000-0001-8963-711X]

Timm Intemann^2^ [ORCID: 0000-0001-7836-3643]

Leonie H. Bogl^3,4^ [ORCID: 0000-0003-4316-2619]

Lauren Lissner^5^

Wencke Gwozdz^6^ [ORCID: 0000-0001-7176-708X]

Stefaan De Henauw^7^

Dénes Molnár^8^

Artur Mazur^9^

Luis A. Moreno^10^

Valeria Pala^11^ [ORCID: 0000-0001-5438-970X]

Paola Russo^12^ [ORCID: 0000-0002-3603-0143]

Michael Tornaritis^13^

Toomas Veidebaum^14^

Garrath Williams^15^ [ORCID: 0000-0002-6583-8133]

Antje Hebestreit^2¶^ [ORCID: 0000-0001-7354-5958]

Benjamin Schüz^1¶^ [ORCID: 0000-0002-0801-498X]

on behalf of the I.Family consortium

^1^ Institute for Public Health and Nursing Research, Department of Prevention and Health Promotion, University of Bremen, 28359 Bremen, Germany

^2^ Leibniz Institute for Prevention Research and Epidemiology — BIPS, 28359 Bremen, Germany

^3^ School of Health Professions, Bern University of Applied Sciences, 3008 Bern, Switzerland

^4^ Finnish Institute of Molecular Medicine, University of Helsinki, 00100 Helsinki, Finland

^5^ Department of Public Health and Community Medicine, Institute of Medicine, Sahlgrenska Academy, University of Gothenburg, 413 90 Gothenburg, Sweden

^6^ Department of Consumer Research, Communication and Food Sociology, Justus-Liebig-University Giessen, 35390 Giessen, Germany

^7^ Department of Public Health and Primary Care, Ghent University, 9000 Ghent, Belgium

^8^ Department of Pediatrics, Medical School, University of Pécs, 7624 Pécs, Hungary

^9^ Institute of Medical Sciences, Medical College, University of Rzeszów, 35-959 Rzeszów, Poland

^10^ GENUD (Growth Exercise, Nutrition and Development) Research Group, Instituto Agroalimentario de Aragón (IA2), Instituto de Investigación Sanitaria Aragón (IIS Aragón), Centro de Investigación Biomédica en Red Fisiopatología de la Obesidad y Nutrición (CIBERObn), University of Zaragoza, Zaragoza 50009, Spain

^11^ Department of Preventive and Predictive Medicine, Fondazione IRCCS, Istituto Nazionale dei Tumori, 20133 Milan, Italy

^12^ Institute of Food Sciences, National Research Council, Avellino, Italy

^13^ Research and Education Institute of Child Health, Strovolos, Cyprus

^14^ National Institute for Health Development, Center of Health and Behavioral Science, 11619 Tallinn, Estonia

^15^ Department of Politics, Philosophy and Religion, Lancaster University, Lancaster, UK

^¶^ shared senior authorship

*** Correspondence:**

Urte Klink

uklink@uni-bremen.de

Table S1: Frequency of responses in % to questions regarding various consumer attitudes towards dietary behaviors

|  | **Belgium**  **N=118** | **Cyprus**  **N=773** | | **Estonia**  **N=595** | **Germany**  **N=509** | **Hungary**  **N=661** | **Italy**  **N=748** | **Spain**  **N=208** | **Sweden**  **N=439** | **Overall**  **N=4051** |
| --- | --- | --- | --- | --- | --- | --- | --- | --- | --- | --- |
| **I compare labels to select the most nutritious food.** in % | | | | | | | | | | |
| Disagree | 23.7 | | 7.5 | 8.9 | 7.9 | 16.2 | 8.8 | 11.1 | 13.9 | 10.8 |
| Moderately disagree | 28.0 | | 9.6 | 14.3 | 28.9 | 20.9 | 7.8 | 10.6 | 19.8 | 15.9 |
| Unsure | 10.2 | | 12.4 | 16.8 | 12.8 | 16.0 | 13.2 | 9.1 | 5.5 | 12.9 |
| Moderately agree | 32.2 | | 37.8 | 42.5 | 39.9 | 29.5 | 38.9 | 43.8 | 45.8 | 38.6 |
| Agree | 5.9 | | 32.7 | 17.5 | 10.6 | 17.4 | 31.3 | 25.5 | 15.0 | 21.9 |
| **I have more confidence in food products that I have seen advertised than in unadvertised products.** in % | | | | | | | | | | |
| Disagree | 50.8 | | 43.5 | 49.4 | 45.2 | 54.2 | 34.8 | 45.2 | 69.0 | 47.8 |
| Moderately disagree | 33.1 | | 24.5 | 38.3 | 41.5 | 30.6 | 22.6 | 33.7 | 23.0 | 29.8 |
| Unsure | 8.5 | | 11.4 | 8.7 | 7.7 | 8.8 | 15.0 | 5.8 | 5.9 | 9.8 |
| Moderately agree | 7.6 | | 15.7 | 3.4 | 4.9 | 5.1 | 20.2 | 11.5 | 2.1 | 9.7 |
| Agree | 0 | | 5.0 | 0.2 | 0.8 | 1.4 | 7.5 | 3.8 | 0 | 2.9 |
| **I try to avoid food products with additives.** in % | | | | | | | | | | |
| Disagree | 11.0 | | 2.8 | 2.9 | 5.3 | 3.8 | 2.7 | 5.8 | 7.1 | 4.1 |
| Moderately disagree | 18.6 | | 4.9 | 5.9 | 19.1 | 6.4 | 4.4 | 11.5 | 17.8 | 9.1 |
| Unsure | 16.9 | | 8.9 | 13.3 | 14.3 | 11.3 | 14.4 | 10.6 | 10.0 | 12.1 |
| Moderately agree | 40.7 | | 27.4 | 43.7 | 44.6 | 33.0 | 25.5 | 38.0 | 43.1 | 35.2 |
| Agree | 12.7 | | 55.9 | 34.3 | 16.7 | 45.5 | 52.9 | 34.1 | 22.1 | 39.5 |
| **I make a point of using natural or ecological products.** in % | | | | | | | | | | |
| Disagree | 8.5 | | 3.2 | 10.1 | 18.5 | 7.0 | 4.3 | 5.8 | 10.0 | 8.0 |
| Moderately disagree | 24.6 | | 5.0 | 22.9 | 38.1 | 10.1 | 7.8 | 13.0 | 24.8 | 16.3 |
| Unsure | 18.6 | | 10.3 | 25.0 | 13.6 | 12.7 | 10.4 | 5.8 | 9.8 | 13.3 |
| Moderately agree | 39.8 | | 40.0 | 30.8 | 26.7 | 37.7 | 38.5 | 48.1 | 43.7 | 37.1 |
| Agree | 8.5 | | 41.4 | 11.3 | 3.1 | 32.5 | 39.0 | 27.4 | 11.6 | 25.4 |
| **I prefer to buy meat and vegetables fresh rather than pre-packed.** in % | | | | | | | | | | |
| Disagree | 0.8 | | 1.7 | 1.7 | 4.3 | 0.6 | 1.5 | 2.9 | 4.3 | 2.1 |
| Moderately disagree | 10.2 | | 1.9 | 4.5 | 16.9 | 3.0 | 3.3 | 1.9 | 11.6 | 5.9 |
| Unsure | 6.8 | | 2.1 | 6.6 | 8.1 | 2.0 | 1.3 | 1.0 | 5.5 | 3.8 |
| Moderately agree | 33.1 | | 12.9 | 43.9 | 46.6 | 25.0 | 22.7 | 21.2 | 34.6 | 28.8 |
| Agree | 49.2 | | 81.4 | 43.4 | 24.2 | 69.4 | 71.1 | 73.1 | 44.0 | 59.3 |
| **I frequently use ready-to-eat foods in our household.** in % | | | | | | | | | | |
| Disagree | 43.2 | | 44.1 | 30.6 | 24.2 | 34.8 | 78.5 | 41.3 | 28.5 | 42.6 |
| Moderately disagree | 39.0 | | 30.7 | 52.3 | 56.8 | 46.7 | 17.5 | 32.2 | 38.5 | 38.5 |
| Unsure | 8.5 | | 5.3 | 9.4 | 8.8 | 8.0 | 1.2 | 2.4 | 5.2 | 6.0 |
| Moderately agree | 9.3 | | 15.1 | 7.4 | 9.6 | 7.7 | 1.6 | 14.9 | 22.3 | 10.2 |
| Agree | 0 | | 4.8 | 0.3 | 0.6 | 2.7 | 1.2 | 9.1 | 5.5 | 2.8 |
| **I frequently use mixes, for instance baking mixes and powder soups.** in % | | | | | | | | | | |
| Disagree | 56.8 | | 65.3 | 48.7 | 32.2 | 61.3 | 84.1 | 65.4 | 73.3 | 62.2 |
| Moderately disagree | 33.9 | | 19.8 | 37.5 | 51.3 | 30.1 | 12.2 | 24.0 | 19.8 | 27.3 |
| Unsure | 5.1 | | 4.4 | 7.2 | 6.5 | 4.2 | 0.7 | 1.4 | 2.1 | 4.0 |
| Moderately agree | 3.4 | | 7.6 | 6.1 | 9.2 | 3.5 | 2.7 | 5.8 | 3.9 | 5.4 |
| Agree | 0.8 | | 2.8 | 0.5 | 0.8 | 0.9 | 0.4 | 3.4 | 0.9 | 1.2 |
| **The kids help in the kitchen, e.g. they peel the potatoes and cut the vegetables.** in % | | | | | | | | | | |
| Disagree | 5.1 | | 12.9 | 9.4 | 4.9 | 7.6 | 31.6 | 10.1 | 7.3 | 13.0 |
| Moderately disagree | 30.5 | | 13.6 | 27.6 | 28.9 | 13.8 | 10.2 | 20.7 | 25.5 | 19.1 |
| Unsure | 11.0 | | 7.9 | 14.5 | 6.5 | 7.0 | 4.8 | 2.9 | 6.4 | 7.6 |
| Moderately agree | 44.9 | | 36.1 | 38.5 | 42.6 | 37.2 | 34.8 | 45.2 | 44.6 | 38.9 |
| Agree | 8.5 | | 29.5 | 10.1 | 17.1 | 34.5 | 18.7 | 21.2 | 16.2 | 21.4 |

Table S2: Sensitivity analysis. Sample characteristics overall, excluding Belgium and Spain

|  | Overall  N=3725 |
| --- | --- |
| **HDAS** Mean (SD) | 25.4 (8.9) |
| **Highest level of education in household** in % | |
| *Low* | 4.6 |
| *Medium* | 43.3 |
| *High* | 49.0 |
| *Missing* | 3.1 |
| **Household income** in % | |
| *Low* | 20.9 |
| *Low-medium* | 8.6 |
| *Medium* | 32.6 |
| *Medium-high* | 11.2 |
| *High* | 21.0 |
| *Missing* | 5.6 |
| **Vulnerable groups** in % | |
| Migrant background | 13.3 |
| Unemployment in household | 9.0 |
| Single parenthood | 10.2 |
| **BMI (kg/m^2^)** mean (SD) | 26.2 (5.3) |
| **Age (years)** mean (SD) | 41.8 (5.7) |
| **Female** in % | 85.2 |
| **Consumer attitudes toward dietary behaviors** in % * | |
| Comparing food labels | 60.7 |
| Trusting food advertisements | 12.6 |
| Avoiding food additives | 75.5 |
| Valuing organic products | 62.2 |
| Preferring fresh meat and vegetables | 88.0 |
| Frequently using ready-to-eat-food | 12.5 |
| Frequently using pre-made mixes | 6.5 |
| Having children help in the kitchen | 60.2 |

Abbreviations: HDAS Healthy Dietary Adherence Score; SD standard deviation; BMI body mass index

*proportion of affirmative responses (“agree” and “moderately agree”)

Table S3: Sensitivity analysis. Frequency of responses in % to questions regarding various consumer attitudes, excluding Belgium and Spain

|  | Overall  N=3725 |
| --- | --- |
| **I compare labels to select the most nutritious food.** in % | |
| Disagree | 10.3 |
| Moderately disagree | 15.8 |
| Unsure | 13.2 |
| Moderately agree | 38.5 |
| Agree | 22.2 |
| **I have more confidence in food products that I have seen advertised than in unadvertised products.** in % | |
| Disagree | 47.8 |
| Moderately disagree | 29.5 |
| Unsure | 10.1 |
| Moderately agree | 9.7 |
| Agree | 2.9 |
| **I try to avoid food products with additives.** in % | |
| Disagree | 3.8 |
| Moderately disagree | 8.7 |
| Unsure | 12.0 |
| Moderately agree | 34.8 |
| Agree | 40.7 |
| **I make a point of using natural or ecological products.** in % | |
| Disagree | 8.1 |
| Moderately disagree | 16.2 |
| Unsure | 13.5 |
| Moderately agree | 36.4 |
| Agree | 25.8 |
| **I prefer to buy meat and vegetables fresh rather than pre-packed.** in % | |
| Disagree | 2.1 |
| Moderately disagree | 6.0 |
| Unsure | 3.8 |
| Moderately agree | 29.1 |
| Agree | 58.9 |
| **I frequently use ready-to-eat foods in our household.** in % | |
| Disagree | 42.6 |
| Moderately disagree | 38.8 |
| Unsure | 6.1 |
| Moderately agree | 10.0 |
| Agree | 2.5 |
| **I frequently use mixes, for instance baking mixes and powder soups.** in % | |
| Disagree | 62.1 |
| Moderately disagree | 27.2 |
| Unsure | 4.1 |
| Moderately agree | 5.4 |
| Agree | 1.1 |
| **The kids help in the kitchen, e.g. they peel the potatoes and cut the vegetables.** in % | |
| Disagree | 13.4 |
| Moderately disagree | 18.7 |
| Unsure | 7.8 |
| Moderately agree | 38.3 |
| Agree | 21.9 |


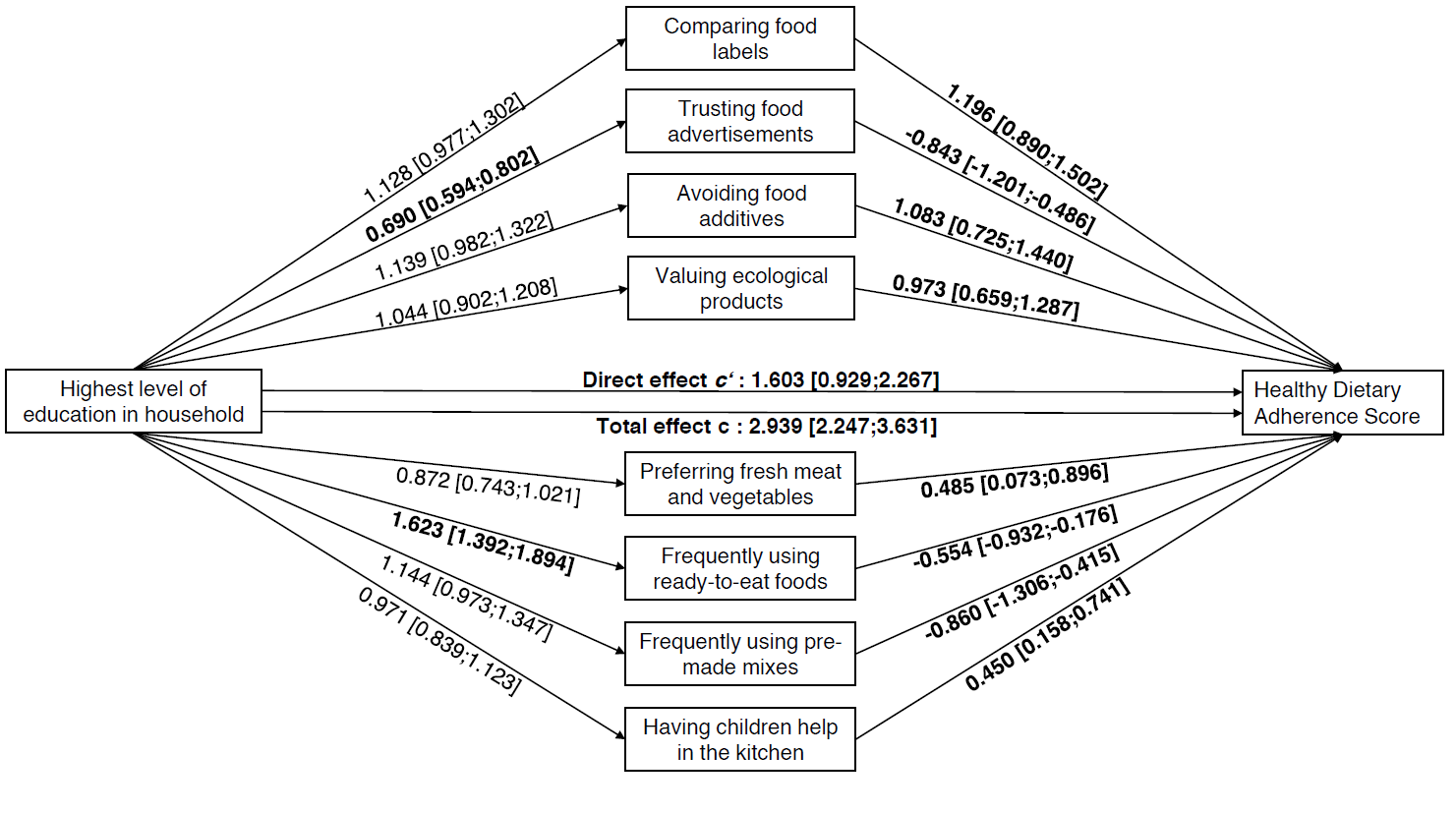


a)


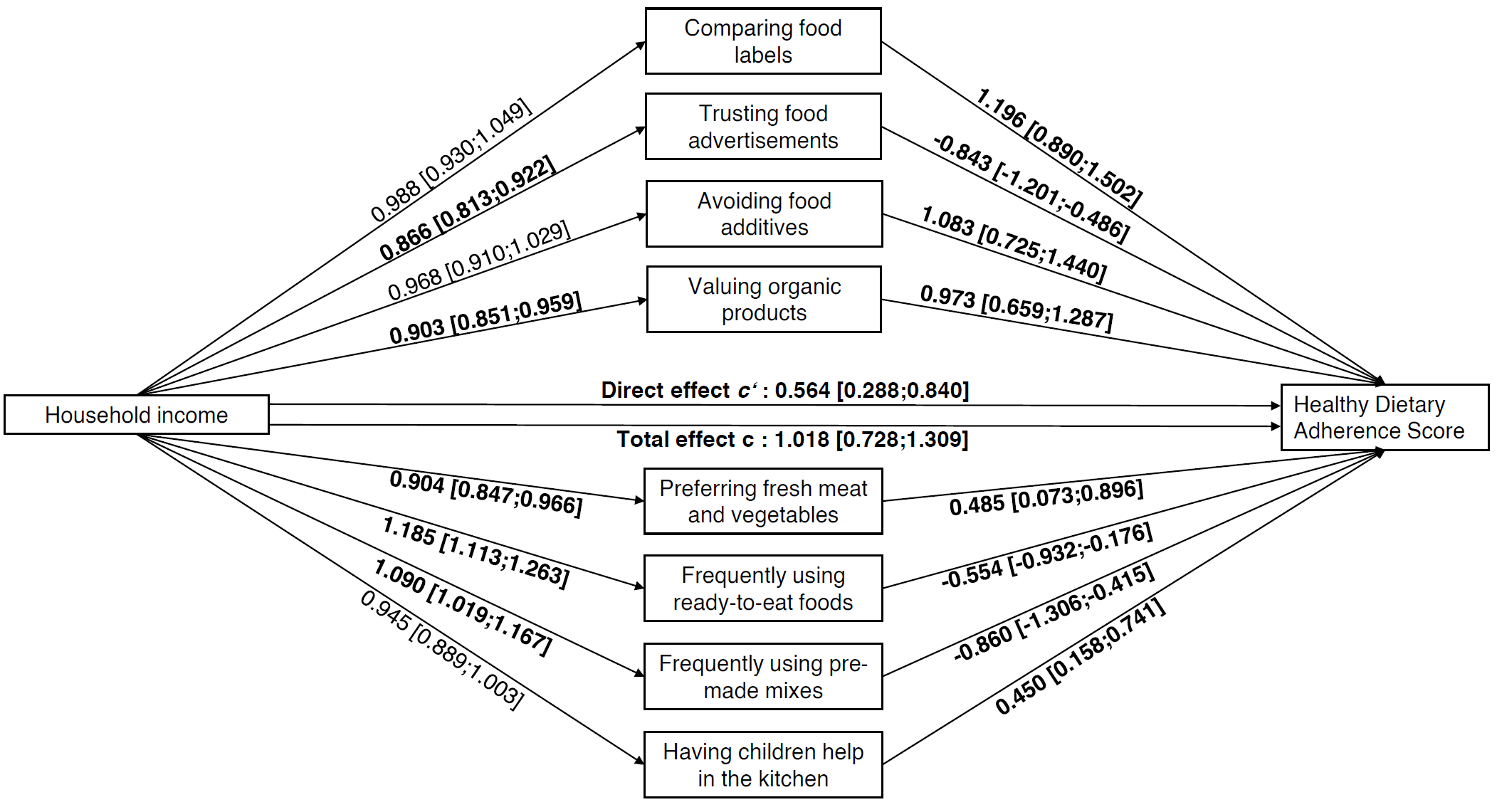


b)


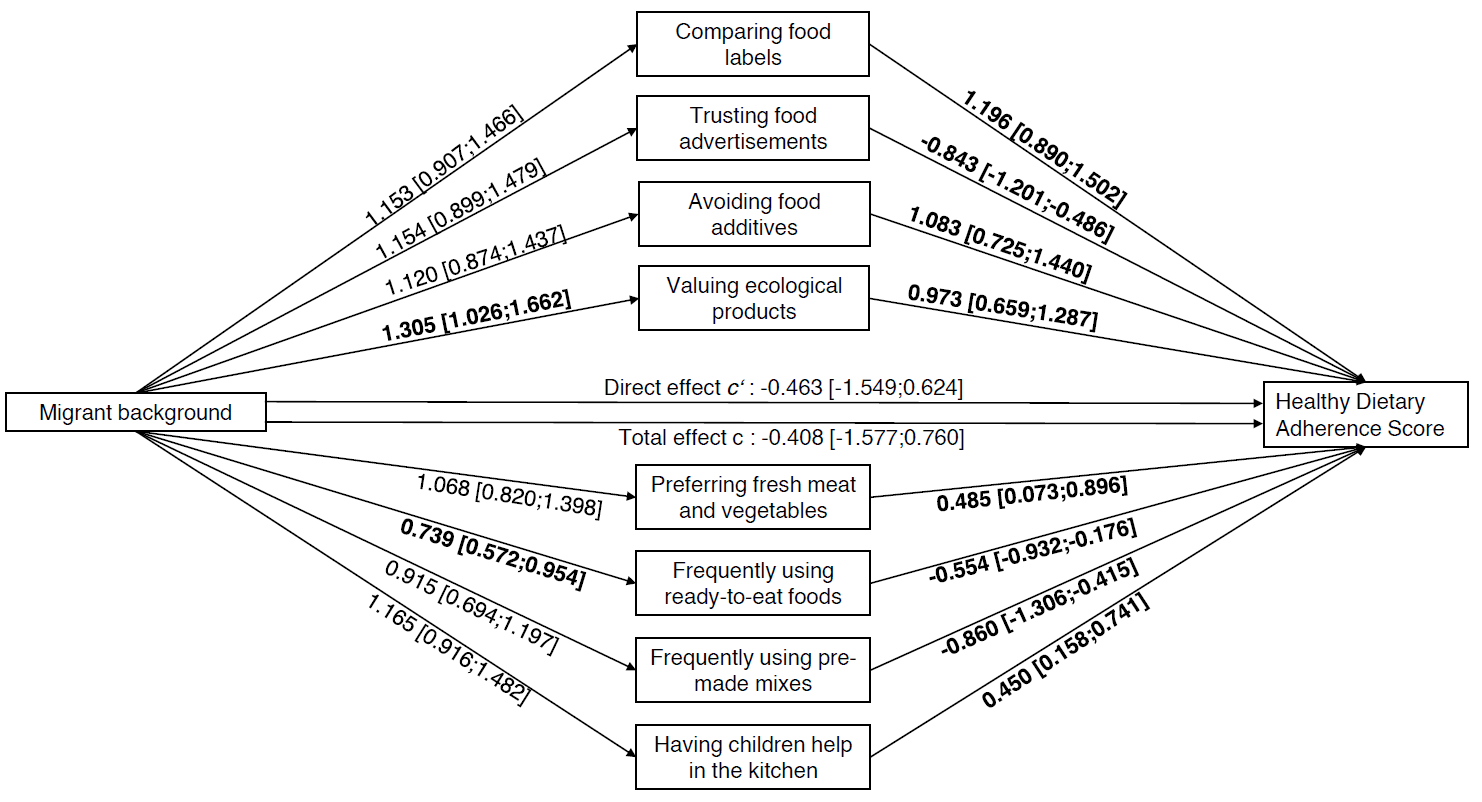


c)


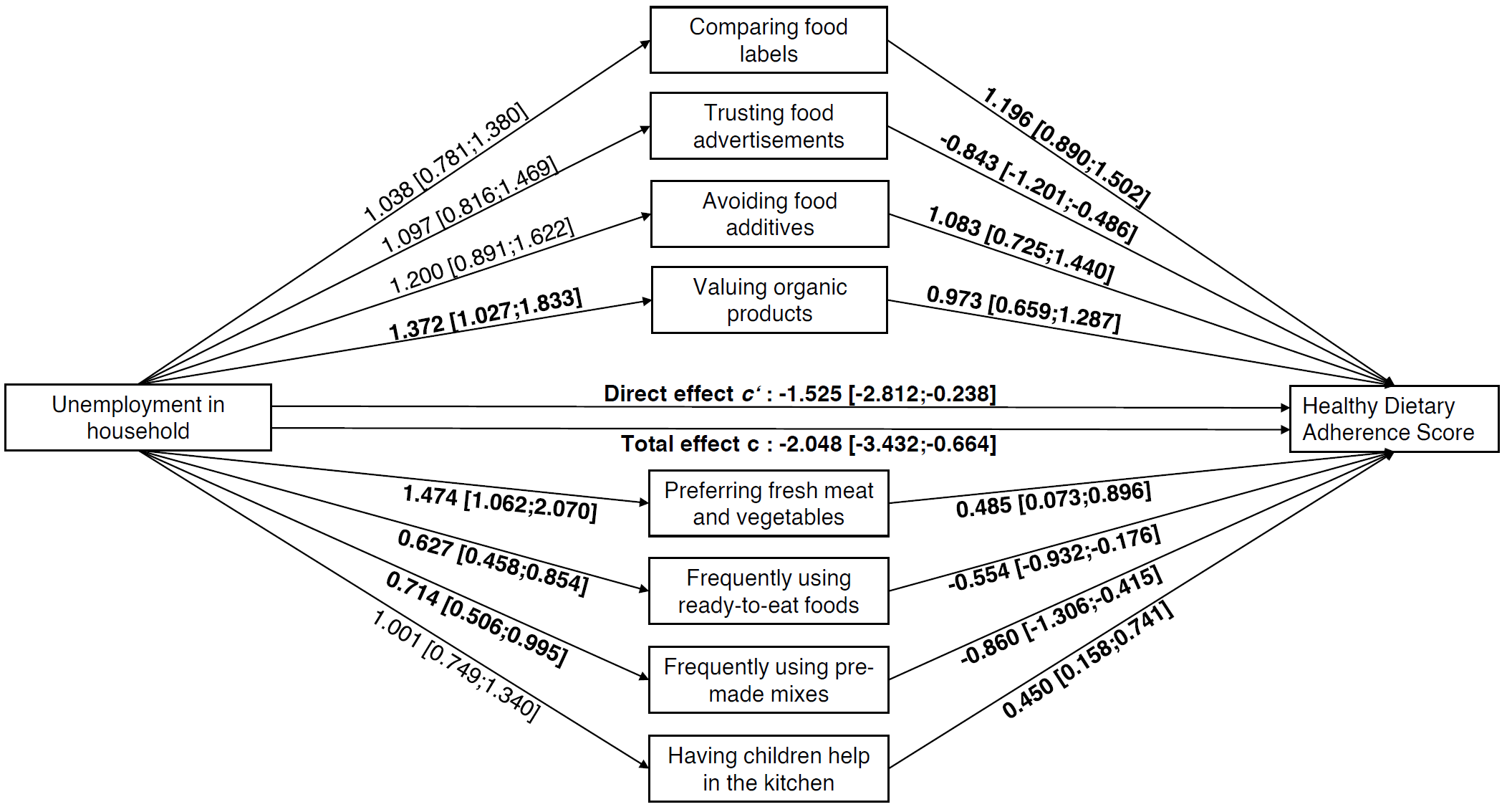


d)


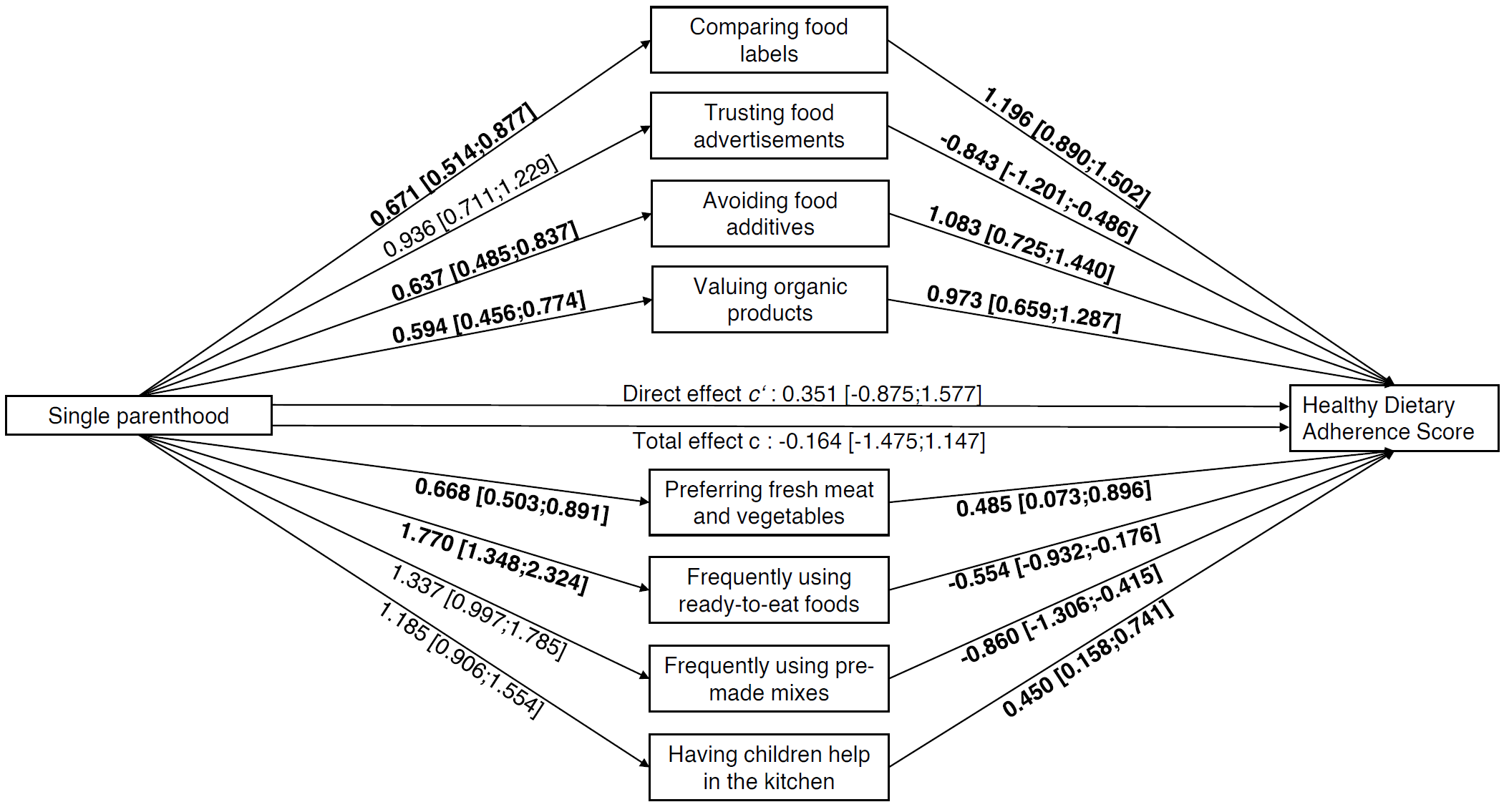


e)

Figure S1: Sensitivity analysis. The mediation of consumer attitudes between socioeconomic factors / social vulnerabilities and diet quality. The total effect shows the association between socioeconomic factors / social vulnerabilities and diet quality without the adjustment for consumer attitudes, while the direct effect shows the association after adjustment for consumer attitudes. Path a shows odds ratios and 99.994% confidence interval, paths b, c and c’ shows β estimate and 99.994% confidence interval. All paths are adjusted for sex, age, and BMI. Excluding Belgium and Spain.

Table S4: Sensitivity analysis. Estimates and confidence intervals of multilevel structural equation modelling for the association between socioeconomic factors / social vulnerabilities and Healthy Dietary Adherence Score mediated by consumer attitudes, adjusted for sex, age and BMI, excluding Belgium and Spain

|  | **Indirect effect *a*b*** | | | | | | | |
| --- | --- | --- | --- | --- | --- | --- | --- | --- |
|  | Comparing food labels | Trusting food advertisements | Avoiding food additives | Valuing organic products | Preferring fresh meat and vegetables | Frequently using ready-to-eat foods | Frequently using pre-made mixes | Having children help in the kitchen |
|  | β [CI] | β [CI] | β [CI] | β [CI] | β [CI] | β [CI] | β [CI] | β [CI] |
| **Highest level of education in household** N=3610 | 0.085  [-0.002;0.171] | **0.160**  **[0.054;0.265]** | 0.043  [-0.017;0.103] | 0.050  [-0.017;0.118] | 0.004  [-0.013;0.021] | **-0.129**  **[-0.225;-0.033]** | -0.000  [-0.006;0.005] | -0.006  [-0.044;0.032] |
| **Household income**  N=3517 | 0.000  [-0.036;0.036] | **0.064**  **[0.020;0.108]** | -0.000  [-0.013;0.013] | -0.035  [-0.069;0.000] | 0.003  [-0.012;0.017] | **-0.034**  **[-0.067;-0.002]** | -0.001  [-0.009;0.006] | -0.013  [-0.031;0.006] |
| **Migrant background**  N=3725 | 0.078  [-0.070;0.226] | -0.096  [-0.209;0.016] | 0.008  [-0.054;0.069] | 0.100  [-0.019;0.219] | 0.001  [-0.016;0.019] | 0.048  [-0.030;0.126] | 0.000  [-0.012;0.013] | 0.034  [-0.034;0.102] |
| **Unemployment in household**  N=3725 | 0.006  [-0.165;0.177] | -0.061  [-0.186;0.064] | 0.014  [-0.059;0.088] | 0.105  [-0.033;0.243] | -0.013  [-0.060;0.035] | 0.077  [-0.025;0.179] | 0.008  [-0.028;0.044] | -0.003  [-0.076;0.070] |
| **Single parenthood**  N=3724 | **-0.235**  **[-0.420;-0.051]** | 0.065  [-0.055;0.184] | -0.106  [-0.233;0.021] | **-0.218**  **[-0.391;-0.044]** | 0.025  [-0.067;0.117] | **-0.135**  **[-0.268;-0.003]** | -0.010  [-0.058;0.038] | 0.047  [-0.032;0.125] |

Note: β̂ estimate; [LLCI; ULCI] = lower and upper levels of 99.994% confidence of the indirect effect estimates between socioeconomic factors / social vulnerabilities and HDAS
