# Supplementary material for: Consumer attitudes towards dietary behaviors: a mediator between socioeconomic status and diet quality in European adults
Source: Eur J Nutr. 2025 Mar 19;64(3):127. doi: 10.1007/s00394-025-03645-6 (PMC11922978; doi:10.1007/s00394-025-03645-6)
Supplement: Supplementary file 2 — Supplementary file2 (HTML 1056 KB) [file 394_2025_3645_MOESM2_ESM.html]

Consumer attitudes towards dietary behaviors: a mediator between socioeconomic status and diet quality in European adults


# Consumer attitudes towards dietary behaviors: a mediator between socioeconomic status and diet quality in European adults

### Online supplement

- Study
  description
  - Description of variables
- 0. Packages, sources,
  directories
- 1.
  Load and prepare data
  - 1.1 Repool
    foodst\_0X values for Belgium and Spain
- 2. Table 1: Study sample
  characteristics
- 3. Table
  2: Responses to consumer attitudes questionnaire
- 4. Analysis
  - 4.1 Path
    analysis
    - 4.1.2 Path a:
      Socioeconomic factors -> Consumer attitudes
    - 4.1.3 Path b: Consumer
      attitudes -> HDAS
    - 5.1.4 Path c:
      Socioeconomic factors -> HDAS (Total effect)
  - 4.2
    Structural equation modelling using lavaan package (indirect and direct
    effect)
    - 4.2.1 Highest level of
      education in household
    - 4.2.2
      Household income
    - 4.2.3
      Migration background
    - 4.2.4 Unemployment in
      household
    - 4.2.5 Single
      parenthood

# Study description

Based on the I.Family survey and only using data of parents, we aimed
to investigate whether consumer attitudes as assessed in the I.Family
cohort serve as mediators in the relationship between socioeconomic
factors and diet quality as indicated by a healthy eating score.

## Description of variables

**family\_id**: family ID number  
**ID\_cohort** / **ID\_no**: Matched ID of
IDEFICS and I. family / ID-Number (I.Family ID)  
**country**: 1 Italy 2 Estonia 3 Cyprus 4 Belgium 6 Sweden
7 Germany 8 Hungary 9 Spain  
**sex\_T3**: Sex of participant (1 male, 2 female)  
**age\_status\_T3**: Age status of participant (1 child, 2
teen, 3 adult)  
**age\_T3**: Age of participant (years)  
**bmi\_T3**: Body Mass Index of participant
(kg/m2)  
**isced\_cat2011\_T3**: ISCED level maximum of both parents
(1 low, 2 medium, 3 high)  
**income\_cat\_T3**: Income categories (1 low, 2 low/medium,
3 medium, 4 medium/high, 5 high)  
**migration\_T3**: Migration status of parents (0 both
parents non-, 1 one parent migrant, 2 both parents migrant)  
**occupst\_1\_T3**: Employment status of parent (1
full-time/30hrs, 2 part-time/15-29hrs, 3 part-time/<15hrs, 4
temporary company leave, 5 apprentice/retrainee, 6 currently
unemployed)  
**occupst\_2\_T3**: Employment status of partner of parent
(…)  
**no\_occupst\_1**: Employment status of parent if working
part-time/not employed (1 attend school, 2 attend university, 3
homemaker, 4 retired, 5 unemployed less than 1 year, 6 unemployed for
more than 1 year, 7 on welfare, 8 doing military, 9 others)  
**no\_occupst\_2**: Employment status of partner of parent if
working part-time/not employed (…)  
**migration**: (Newly created) Migration status of parents
of participant (1 no migrant background, 2 migrant background)  
**unemploy**: (Newly created) Unemployment in household,
one adult/parent is unemployed (1 no unemployment, 2 unemployment)  
**singlepar**: (Newly created) Single parenthood (1 no
single parent, 2 single parent)  
**hds\_T3**: Healthy Dietary Adherence Score (range
0-50)

**Consumer attitudes as mediators**

5-point likert scale: 1 disagree, 2 moderately disagree, 3 unsure, 4
moderately agree, 5 agree

**foodst\_01\_T3**: I compare labels to select the most
nutritious food.  
**foodst\_02\_T3**: I have more confidence in food products
that I have seen advertised than in unadvertised products.  
**foodst\_03\_T3**: I try to avoid food products with
additives.  
**foodst\_04\_T3**: I make a point of using natural or
ecological products.  
**foodst\_05\_T3**: I prefer to buy meat and vegetables fresh
rather than pre-packed.  
**foodst\_06\_T3**: I frequently use ready-to-eat foods in
our household.  
**foodst\_07\_T3**: I frequently use mixes, for instance
baking mixes and powder soups.  
**foodst\_08\_T3**: The kids help in the kitchen, e.g. they
peel the potatoes and cut the vegetables.

# 0. Packages, sources, directories

```
knitr::opts_chunk$set(echo = TRUE)

require(knitr)
require(kabelExtra)
require(tidyverse)
require(table1)
require(DiagrammeR)
require(lavaan)
require(car)
```

# 1. Load and prepare data

```
## create data frame subset with all relevant variables

core_subset <- core %>%
  #only relevant variables
  select(ID_cohort, ID_no, family_id, country, age_T3, age_status_T3, sex_T3, bmi_T3, isced_cat2011_T3, income_cat_T3, migration_T3, hds_T3) %>%
  #adults only
  filter(age_status_T3 == 3) %>% 
  #exclude those with missings for HDAS and BMI
  filter(hds_T3 != "NA", bmi_T3 != "NA") %>%
  #create new variable for participant whose parents have migration background (no = 1, yes = 2)
  mutate(migration = ifelse(migration_T3 %in% c(1,2), 2, 1)) %>%
  #migration as integer
  mutate(migration = as.integer(migration)) %>%
  #create new categorical variable for country
  mutate(country_name = case_when(
    country == "1" ~ "Italy",
    country == "2" ~ "Estonia",
    country == "3" ~ "Cyprus",
    country == "4" ~ "Belgium",
    country == "6" ~ "Sweden",
    country == "7" ~ "Germany",
    country == "8" ~ "Hungary",
    country == "9" ~ "Spain",
    TRUE ~ as.character(country)
  ) )

fa_subset <- fa %>%
  #only relevant variables
  select(FAMILY_ID, country, sex_fill_T3, occupst_1_T3, occupst_2_T3, no_occupst_1_T3, no_occupst_2_T3,foodst_01_T3, foodst_02_T3, foodst_03_T3, foodst_04_T3, foodst_05_T3, foodst_06_T3, foodst_07_T3, foodst_08_T3) %>%
  #exclude those with missings for consumer attitudes
  filter(foodst_01_T3 != "NA",  foodst_02_T3 != "NA", foodst_03_T3 != "NA", foodst_04_T3 != "NA", foodst_05_T3 != "NA", foodst_06_T3 != "NA", foodst_07_T3 != "NA", foodst_08_T3 != "NA"  ) %>% 
  #new variable for adults living in families with one or both adult family members unemployed (no = 1, yes = 2)
  mutate(unemploy = ifelse(no_occupst_1_T3 %in% c(5,6,7) | no_occupst_2_T3 %in% c(5,6,7), 2, 1)) %>%
  #unemployment as integer
  mutate(unemploy = as.integer(unemploy))

kh_subset <- kh %>%
  select(family_ID, country, househ_o18_T3) %>%
  #new variable for single parenthood (no = 1, yes = 2)
  mutate(singlepar = ifelse(househ_o18_T3 == 1, 2, 1)) %>%
  #singlepar as integer
  mutate(singlepar = as.integer(singlepar))

# Rename family_ID in fa and kh data frame
names(fa_subset)[names(fa_subset) == "FAMILY_ID"] <- "family_id"
names(fa_subset)[names(fa_subset) == "sex_fill_T3"] <- "sex_T3"
names(kh_subset)[names(kh_subset) == "family_ID"] <- "family_id"

# Merge data frames - automatic merging by family_id, sex, country
subset <- merge(core_subset, fa_subset)
subset <- merge(subset, kh_subset)
```

## 1.1 Repool foodst\_0X values for Belgium and Spain

```
subset <- subset %>%
  mutate(foodst_01_T3 = case_when(
    country == "4" & foodst_01_T3 == 1 ~ 5,
    country == "4" & foodst_01_T3 == 5 ~ 1,
    country == "4" & foodst_01_T3 == 2 ~ 4,
    country == "4" & foodst_01_T3 == 4 ~ 2,
    TRUE ~ foodst_01_T3
  )) %>%
  mutate(foodst_02_T3 = case_when(
    country == "4" & foodst_02_T3 == 1 ~ 5,
    country == "4" & foodst_02_T3 == 5 ~ 1,
    country == "4" & foodst_02_T3 == 2 ~ 4,
    country == "4" & foodst_02_T3 == 4 ~ 2,
    TRUE ~ foodst_02_T3
  )) %>%
  mutate(foodst_03_T3 = case_when(
    country == "4" & foodst_03_T3 == 1 ~ 5,
    country == "4" & foodst_03_T3 == 5 ~ 1,
    country == "4" & foodst_03_T3 == 2 ~ 4,
    country == "4" & foodst_03_T3 == 4 ~ 2,
    TRUE ~ foodst_03_T3
  )) %>%
  mutate(foodst_04_T3 = case_when(
    country == "4" & foodst_04_T3 == 1 ~ 5,
    country == "4" & foodst_04_T3 == 5 ~ 1,
    country == "4" & foodst_04_T3 == 2 ~ 4,
    country == "4" & foodst_04_T3 == 4 ~ 2,
    TRUE ~ foodst_04_T3
  )) %>%
  mutate(foodst_05_T3 = case_when(
    country == "4" & foodst_05_T3 == 1 ~ 5,
    country == "4" & foodst_05_T3 == 5 ~ 1,
    country == "4" & foodst_05_T3 == 2 ~ 4,
    country == "4" & foodst_05_T3 == 4 ~ 2,
    TRUE ~ foodst_05_T3
  )) %>%
  mutate(foodst_06_T3 = case_when(
    country == "4" & foodst_06_T3 == 1 ~ 5,
    country == "4" & foodst_06_T3 == 5 ~ 1,
    country == "4" & foodst_06_T3 == 2 ~ 4,
    country == "4" & foodst_06_T3 == 4 ~ 2,
    TRUE ~ foodst_06_T3
  )) %>%
  mutate(foodst_07_T3 = case_when(
    country == "4" & foodst_07_T3 == 1 ~ 5,
    country == "4" & foodst_07_T3 == 5 ~ 1,
    country == "4" & foodst_07_T3 == 2 ~ 4,
    country == "4" & foodst_07_T3 == 4 ~ 2,
    TRUE ~ foodst_07_T3
  )) %>%
  mutate(foodst_08_T3 = case_when(
    country == "4" & foodst_08_T3 == 1 ~ 5,
    country == "4" & foodst_08_T3 == 5 ~ 1,
    country == "4" & foodst_08_T3 == 2 ~ 4,
    country == "4" & foodst_08_T3 == 4 ~ 2,
    TRUE ~ foodst_08_T3
  )) %>%
  mutate(foodst_01_T3 = case_when(
    country == "9" & foodst_01_T3 == 1 ~ 5,
    country == "9" & foodst_01_T3 == 5 ~ 1,
    country == "9" & foodst_01_T3 == 2 ~ 4,
    country == "9" & foodst_01_T3 == 4 ~ 2,
    TRUE ~ foodst_01_T3
  )) %>%
  mutate(foodst_02_T3 = case_when(
    country == "9" & foodst_02_T3 == 1 ~ 5,
    country == "9" & foodst_02_T3 == 5 ~ 1,
    country == "9" & foodst_02_T3 == 2 ~ 4,
    country == "9" & foodst_02_T3 == 4 ~ 2,
    TRUE ~ foodst_02_T3
  )) %>%
  mutate(foodst_03_T3 = case_when(
    country == "9" & foodst_03_T3 == 1 ~ 5,
    country == "9" & foodst_03_T3 == 5 ~ 1,
    country == "9" & foodst_03_T3 == 2 ~ 4,
    country == "9" & foodst_03_T3 == 4 ~ 2,
    TRUE ~ foodst_03_T3
  )) %>%
  mutate(foodst_04_T3 = case_when(
    country == "9" & foodst_04_T3 == 1 ~ 5,
    country == "9" & foodst_04_T3 == 5 ~ 1,
    country == "9" & foodst_04_T3 == 2 ~ 4,
    country == "9" & foodst_04_T3 == 4 ~ 2,
    TRUE ~ foodst_04_T3
  )) %>%
  mutate(foodst_05_T3 = case_when(
    country == "9" & foodst_05_T3 == 1 ~ 5,
    country == "9" & foodst_05_T3 == 5 ~ 1,
    country == "9" & foodst_05_T3 == 2 ~ 4,
    country == "9" & foodst_05_T3 == 4 ~ 2,
    TRUE ~ foodst_05_T3
  )) %>%
  mutate(foodst_06_T3 = case_when(
    country == "9" & foodst_06_T3 == 1 ~ 5,
    country == "9" & foodst_06_T3 == 5 ~ 1,
    country == "9" & foodst_06_T3 == 2 ~ 4,
    country == "9" & foodst_06_T3 == 4 ~ 2,
    TRUE ~ foodst_06_T3
  )) %>%
  mutate(foodst_07_T3 = case_when(
    country == "9" & foodst_07_T3 == 1 ~ 5,
    country == "9" & foodst_07_T3 == 5 ~ 1,
    country == "9" & foodst_07_T3 == 2 ~ 4,
    country == "9" & foodst_07_T3 == 4 ~ 2,
    TRUE ~ foodst_07_T3
  )) %>%
  mutate(foodst_08_T3 = case_when(
    country == "9" & foodst_08_T3 == 1 ~ 5,
    country == "9" & foodst_08_T3 == 5 ~ 1,
    country == "9" & foodst_08_T3 == 2 ~ 4,
    country == "9" & foodst_08_T3 == 4 ~ 2,
    TRUE ~ foodst_08_T3
  ))
```

# 2. Table 1: Study sample characteristics

```
subset$isced_cat2011_T3 <-
  factor(subset$isced_cat2011_T3, levels=c(1,2,3),
         labels = c("low", "medium", "high"))
label(subset$isced_cat2011_T3) <- "Highest education level in household"

subset$income_cat_T3 <-
  factor(subset$income_cat_T3, levels=c(1,2,3,4,5),
         labels = c("low", "low/medium", "medium", "medium/high", "high"))
label(subset$income_cat_T3) <- "Household income"

subset$migration <- 
  factor(subset$migration, levels=c(2, 1),
         labels=c("Yes", 
                  "No"))
label(subset$migration) <- "Migrant background"

subset$unemploy <- 
  factor(subset$unemploy, levels=c(2, 1),
         labels=c("Yes", 
                  "No"))
label(subset$unemploy) <- "Unemployment in household"

subset$singlepar <- 
  factor(subset$singlepar, levels=c(2, 1),
         labels=c("Yes", 
                  "No"))
label(subset$singlepar) <- "Single parenthood"

subset$sex_T3 <- 
  factor(subset$sex_T3, levels=c(1, 2),
         labels=c("Male", 
                  "Female"))
label(subset$sex_T3) <- "Sex"

label(subset$hds_T3) <- "HDAS"
label(subset$bmi_T3) <- "BMI"
label(subset$age_T3) <- "Age"

units(subset$bmi_T3) <- "kg/m^2"
units(subset$age_T3) <- "years"

caption1 <- "Table 1: Study sample characteristics"
footnote1 <- "Abbreviations: HDAS = Healthy Dietary Adherence Score ; SD  = standard deviation ; BMI  = body mass index "

table1(~ hds_T3 +
         isced_cat2011_T3 + income_cat_T3 +  
         migration + unemploy + singlepar +
         bmi_T3 + age_T3 + sex_T3  | 
         country_name, data = subset, caption=caption1, footnote=footnote1)
```

Table 1: Study sample characteristics

|  | Belgium (N=118) | Cyprus (N=773) | Estonia (N=595) | Germany (N=509) | Hungary (N=661) | Italy (N=748) | Spain (N=208) | Sweden (N=439) | Overall (N=4051) |
| --- | --- | --- | --- | --- | --- | --- | --- | --- | --- |
|  |  |  |  |  |  |  |  |  |  |
| --- | --- | --- | --- | --- | --- | --- | --- | --- | --- |
| Abbreviations: HDAS = Healthy Dietary Adherence Score ; SD = standard deviation ; BMI = body mass index | | | | | | | | | |
| HDAS |  |  |  |  |  |  |  |  |  |
| Mean (SD) | 27.9 (7.23) | 25.0 (9.11) | 27.7 (7.89) | 23.0 (8.89) | 23.2 (8.88) | 24.3 (8.03) | 27.1 (7.82) | 30.7 (8.06) | 25.5 (8.79) |
| Median [Min, Max] | 28.0 [6.00, 45.0] | 25.0 [1.00, 48.0] | 28.0 [3.00, 47.0] | 23.0 [1.00, 45.0] | 23.0 [2.00, 48.0] | 24.5 [0, 48.0] | 27.0 [10.0, 47.0] | 32.0 [7.00, 48.0] | 26.0 [0, 48.0] |
| Highest education level in household |  |  |  |  |  |  |  |  |  |
| low | 3 (2.5%) | 9 (1.2%) | 0 (0%) | 32 (6.3%) | 14 (2.1%) | 118 (15.8%) | 5 (2.4%) | 0 (0%) | 181 (4.5%) |
| medium | 20 (16.9%) | 283 (36.6%) | 163 (27.4%) | 293 (57.6%) | 315 (47.7%) | 453 (60.6%) | 64 (30.8%) | 105 (23.9%) | 1696 (41.9%) |
| high | 94 (79.7%) | 437 (56.5%) | 428 (71.9%) | 181 (35.6%) | 308 (46.6%) | 144 (19.3%) | 135 (64.9%) | 327 (74.5%) | 2054 (50.7%) |
| Missing | 1 (0.8%) | 44 (5.7%) | 4 (0.7%) | 3 (0.6%) | 24 (3.6%) | 33 (4.4%) | 4 (1.9%) | 7 (1.6%) | 120 (3.0%) |
| Household income |  |  |  |  |  |  |  |  |  |
| low | 3 (2.5%) | 210 (27.2%) | 71 (11.9%) | 85 (16.7%) | 93 (14.1%) | 311 (41.6%) | 14 (6.7%) | 10 (2.3%) | 797 (19.7%) |
| low/medium | 3 (2.5%) | 75 (9.7%) | 23 (3.9%) | 54 (10.6%) | 46 (7.0%) | 112 (15.0%) | 13 (6.3%) | 12 (2.7%) | 338 (8.3%) |
| medium | 65 (55.1%) | 287 (37.1%) | 148 (24.9%) | 233 (45.8%) | 240 (36.3%) | 160 (21.4%) | 78 (37.5%) | 146 (33.3%) | 1357 (33.5%) |
| medium/high | 18 (15.3%) | 80 (10.3%) | 57 (9.6%) | 59 (11.6%) | 92 (13.9%) | 11 (1.5%) | 34 (16.3%) | 119 (27.1%) | 470 (11.6%) |
| high | 22 (18.6%) | 97 (12.5%) | 284 (47.7%) | 48 (9.4%) | 149 (22.5%) | 59 (7.9%) | 58 (27.9%) | 146 (33.3%) | 863 (21.3%) |
| Missing | 7 (5.9%) | 24 (3.1%) | 12 (2.0%) | 30 (5.9%) | 41 (6.2%) | 95 (12.7%) | 11 (5.3%) | 6 (1.4%) | 226 (5.6%) |
| Migrant background |  |  |  |  |  |  |  |  |  |
| Yes | 4 (3.4%) | 152 (19.7%) | 20 (3.4%) | 100 (19.6%) | 21 (3.2%) | 132 (17.6%) | 16 (7.7%) | 69 (15.7%) | 514 (12.7%) |
| No | 114 (96.6%) | 621 (80.3%) | 575 (96.6%) | 409 (80.4%) | 640 (96.8%) | 616 (82.4%) | 192 (92.3%) | 370 (84.3%) | 3537 (87.3%) |
| Unemployment in household |  |  |  |  |  |  |  |  |  |
| Yes | 2 (1.7%) | 116 (15.0%) | 22 (3.7%) | 38 (7.5%) | 46 (7.0%) | 104 (13.9%) | 19 (9.1%) | 10 (2.3%) | 357 (8.8%) |
| No | 116 (98.3%) | 657 (85.0%) | 573 (96.3%) | 471 (92.5%) | 615 (93.0%) | 644 (86.1%) | 189 (90.9%) | 429 (97.7%) | 3694 (91.2%) |
| Single parenthood |  |  |  |  |  |  |  |  |  |
| Yes | 13 (11.0%) | 52 (6.7%) | 70 (11.8%) | 77 (15.1%) | 96 (14.5%) | 27 (3.6%) | 19 (9.1%) | 58 (13.2%) | 412 (10.2%) |
| No | 105 (89.0%) | 721 (93.3%) | 525 (88.2%) | 431 (84.7%) | 565 (85.5%) | 721 (96.4%) | 174 (83.7%) | 381 (86.8%) | 3623 (89.4%) |
| Missing | 0 (0%) | 0 (0%) | 0 (0%) | 1 (0.2%) | 0 (0%) | 0 (0%) | 15 (7.2%) | 0 (0%) | 16 (0.4%) |
| BMI (kg/m^2) |  |  |  |  |  |  |  |  |  |
| Mean (SD) | 24.1 (4.58) | 26.2 (5.11) | 25.4 (5.37) | 26.7 (5.68) | 26.1 (5.57) | 27.4 (5.38) | 25.1 (4.38) | 24.7 (3.90) | 26.1 (5.27) |
| Median [Min, Max] | 23.1 [17.6, 40.5] | 25.3 [17.3, 46.7] | 24.1 [16.4, 53.5] | 25.4 [16.7, 53.7] | 25.1 [15.5, 55.6] | 26.5 [17.3, 49.7] | 24.2 [17.3, 42.4] | 23.9 [17.1, 50.3] | 25.0 [15.5, 55.6] |
| Age (years) |  |  |  |  |  |  |  |  |  |
| Mean (SD) | 41.2 (4.79) | 41.5 (5.91) | 39.6 (5.34) | 42.9 (5.87) | 40.8 (5.12) | 42.7 (5.53) | 44.9 (4.08) | 43.8 (5.26) | 41.9 (5.63) |
| Median [Min, Max] | 41.0 [29.4, 60.8] | 41.0 [27.0, 70.6] | 39.4 [24.0, 75.9] | 42.9 [26.2, 63.0] | 40.0 [24.4, 61.1] | 42.7 [26.7, 65.9] | 44.9 [33.3, 62.1] | 43.7 [30.6, 65.4] | 41.7 [24.0, 75.9] |
| Sex |  |  |  |  |  |  |  |  |  |
| Male | 15 (12.7%) | 168 (21.7%) | 56 (9.4%) | 60 (11.8%) | 80 (12.1%) | 92 (12.3%) | 35 (16.8%) | 94 (21.4%) | 600 (14.8%) |
| Female | 103 (87.3%) | 605 (78.3%) | 539 (90.6%) | 449 (88.2%) | 581 (87.9%) | 656 (87.7%) | 173 (83.2%) | 345 (78.6%) | 3451 (85.2%) |

# 3. Table 2: Responses to consumer attitudes questionnaire

```
subset$foodst_01_T3 <-
  factor(subset$foodst_01_T3, levels=c(1,2,3,4,5),
         labels = c("Disagree", "Moderately disagree", "Unsure", "Moderately agree", "Agree"))
subset$foodst_02_T3 <-
  factor(subset$foodst_02_T3, levels=c(1,2,3,4,5),
         labels = c("Disagree", "Moderately disagree", "Unsure", "Moderately agree", "Agree"))
subset$foodst_03_T3 <-
  factor(subset$foodst_03_T3, levels=c(1,2,3,4,5),
         labels = c("Disagree", "Moderately disagree", "Unsure", "Moderately agree", "Agree"))
subset$foodst_04_T3 <-
  factor(subset$foodst_04_T3, levels=c(1,2,3,4,5),
         labels = c("Disagree", "Moderately disagree", "Unsure", "Moderately agree", "Agree"))
subset$foodst_05_T3 <-
  factor(subset$foodst_05_T3, levels=c(1,2,3,4,5),
         labels = c("Disagree", "Moderately disagree", "Unsure", "Moderately agree", "Agree"))
subset$foodst_06_T3 <-
  factor(subset$foodst_06_T3, levels=c(1,2,3,4,5),
         labels = c("Disagree", "Moderately disagree", "Unsure", "Moderately agree", "Agree"))
subset$foodst_07_T3 <-
  factor(subset$foodst_07_T3, levels=c(1,2,3,4,5),
         labels = c("Disagree", "Moderately disagree", "Unsure", "Moderately agree", "Agree"))
subset$foodst_08_T3 <-
  factor(subset$foodst_08_T3, levels=c(1,2,3,4,5),
         labels = c("Disagree", "Moderately disagree", "Unsure", "Moderately agree", "Agree"))

label(subset$foodst_01_T3) <- "Comparing food labels"
label(subset$foodst_02_T3) <- "Trusting food advertisements"
label(subset$foodst_03_T3) <- "Avoiding food additives"
label(subset$foodst_04_T3) <- "Valuing ecological products"
label(subset$foodst_05_T3) <- "Preferring fresh meat and vegetables"
label(subset$foodst_06_T3) <- "Frequently using ready-to-eat foods"
label(subset$foodst_07_T3) <- "Frequently using pre-made mixes"
label(subset$foodst_08_T3) <- "Having children help in the kitchen"

caption2 <- "Table 2: Frequency of responses in % to questions regarding various consumer attitudes"

table1(~foodst_01_T3 + foodst_02_T3 + foodst_03_T3 + foodst_04_T3 + foodst_05_T3 + foodst_06_T3 + foodst_07_T3 + foodst_08_T3 | country_name, data = subset, caption=caption2)
```

Table 2: Frequency of responses in % to questions regarding various consumer attitudes

|  | Belgium (N=118) | Cyprus (N=773) | Estonia (N=595) | Germany (N=509) | Hungary (N=661) | Italy (N=748) | Spain (N=208) | Sweden (N=439) | Overall (N=4051) |
| --- | --- | --- | --- | --- | --- | --- | --- | --- | --- |
| Comparing food labels |  |  |  |  |  |  |  |  |  |
| Disagree | 28 (23.7%) | 58 (7.5%) | 53 (8.9%) | 40 (7.9%) | 107 (16.2%) | 66 (8.8%) | 23 (11.1%) | 61 (13.9%) | 436 (10.8%) |
| Moderately disagree | 33 (28.0%) | 74 (9.6%) | 85 (14.3%) | 147 (28.9%) | 138 (20.9%) | 58 (7.8%) | 22 (10.6%) | 87 (19.8%) | 644 (15.9%) |
| Unsure | 12 (10.2%) | 96 (12.4%) | 100 (16.8%) | 65 (12.8%) | 106 (16.0%) | 99 (13.2%) | 19 (9.1%) | 24 (5.5%) | 521 (12.9%) |
| Moderately agree | 38 (32.2%) | 292 (37.8%) | 253 (42.5%) | 203 (39.9%) | 195 (29.5%) | 291 (38.9%) | 91 (43.8%) | 201 (45.8%) | 1564 (38.6%) |
| Agree | 7 (5.9%) | 253 (32.7%) | 104 (17.5%) | 54 (10.6%) | 115 (17.4%) | 234 (31.3%) | 53 (25.5%) | 66 (15.0%) | 886 (21.9%) |
| Trusting food advertisements |  |  |  |  |  |  |  |  |  |
| Disagree | 60 (50.8%) | 336 (43.5%) | 294 (49.4%) | 230 (45.2%) | 358 (54.2%) | 260 (34.8%) | 94 (45.2%) | 303 (69.0%) | 1935 (47.8%) |
| Moderately disagree | 39 (33.1%) | 189 (24.5%) | 228 (38.3%) | 211 (41.5%) | 202 (30.6%) | 169 (22.6%) | 70 (33.7%) | 101 (23.0%) | 1209 (29.8%) |
| Unsure | 10 (8.5%) | 88 (11.4%) | 52 (8.7%) | 39 (7.7%) | 58 (8.8%) | 112 (15.0%) | 12 (5.8%) | 26 (5.9%) | 397 (9.8%) |
| Moderately agree | 9 (7.6%) | 121 (15.7%) | 20 (3.4%) | 25 (4.9%) | 34 (5.1%) | 151 (20.2%) | 24 (11.5%) | 9 (2.1%) | 393 (9.7%) |
| Agree | 0 (0%) | 39 (5.0%) | 1 (0.2%) | 4 (0.8%) | 9 (1.4%) | 56 (7.5%) | 8 (3.8%) | 0 (0%) | 117 (2.9%) |
| Avoiding food additives |  |  |  |  |  |  |  |  |  |
| Disagree | 13 (11.0%) | 22 (2.8%) | 17 (2.9%) | 27 (5.3%) | 25 (3.8%) | 20 (2.7%) | 12 (5.8%) | 31 (7.1%) | 167 (4.1%) |
| Moderately disagree | 22 (18.6%) | 38 (4.9%) | 35 (5.9%) | 97 (19.1%) | 42 (6.4%) | 33 (4.4%) | 24 (11.5%) | 78 (17.8%) | 369 (9.1%) |
| Unsure | 20 (16.9%) | 69 (8.9%) | 79 (13.3%) | 73 (14.3%) | 75 (11.3%) | 108 (14.4%) | 22 (10.6%) | 44 (10.0%) | 490 (12.1%) |
| Moderately agree | 48 (40.7%) | 212 (27.4%) | 260 (43.7%) | 227 (44.6%) | 218 (33.0%) | 191 (25.5%) | 79 (38.0%) | 189 (43.1%) | 1424 (35.2%) |
| Agree | 15 (12.7%) | 432 (55.9%) | 204 (34.3%) | 85 (16.7%) | 301 (45.5%) | 396 (52.9%) | 71 (34.1%) | 97 (22.1%) | 1601 (39.5%) |
| Valuing ecological products |  |  |  |  |  |  |  |  |  |
| Disagree | 10 (8.5%) | 25 (3.2%) | 60 (10.1%) | 94 (18.5%) | 46 (7.0%) | 32 (4.3%) | 12 (5.8%) | 44 (10.0%) | 323 (8.0%) |
| Moderately disagree | 29 (24.6%) | 39 (5.0%) | 136 (22.9%) | 194 (38.1%) | 67 (10.1%) | 58 (7.8%) | 27 (13.0%) | 109 (24.8%) | 659 (16.3%) |
| Unsure | 22 (18.6%) | 80 (10.3%) | 149 (25.0%) | 69 (13.6%) | 84 (12.7%) | 78 (10.4%) | 12 (5.8%) | 43 (9.8%) | 537 (13.3%) |
| Moderately agree | 47 (39.8%) | 309 (40.0%) | 183 (30.8%) | 136 (26.7%) | 249 (37.7%) | 288 (38.5%) | 100 (48.1%) | 192 (43.7%) | 1504 (37.1%) |
| Agree | 10 (8.5%) | 320 (41.4%) | 67 (11.3%) | 16 (3.1%) | 215 (32.5%) | 292 (39.0%) | 57 (27.4%) | 51 (11.6%) | 1028 (25.4%) |
| Preferring fresh meat and vegetables |  |  |  |  |  |  |  |  |  |
| Disagree | 1 (0.8%) | 13 (1.7%) | 10 (1.7%) | 22 (4.3%) | 4 (0.6%) | 11 (1.5%) | 6 (2.9%) | 19 (4.3%) | 86 (2.1%) |
| Moderately disagree | 12 (10.2%) | 15 (1.9%) | 27 (4.5%) | 86 (16.9%) | 20 (3.0%) | 25 (3.3%) | 4 (1.9%) | 51 (11.6%) | 240 (5.9%) |
| Unsure | 8 (6.8%) | 16 (2.1%) | 39 (6.6%) | 41 (8.1%) | 13 (2.0%) | 10 (1.3%) | 2 (1.0%) | 24 (5.5%) | 153 (3.8%) |
| Moderately agree | 39 (33.1%) | 100 (12.9%) | 261 (43.9%) | 237 (46.6%) | 165 (25.0%) | 170 (22.7%) | 44 (21.2%) | 152 (34.6%) | 1168 (28.8%) |
| Agree | 58 (49.2%) | 629 (81.4%) | 258 (43.4%) | 123 (24.2%) | 459 (69.4%) | 532 (71.1%) | 152 (73.1%) | 193 (44.0%) | 2404 (59.3%) |
| Frequently using ready-to-eat foods |  |  |  |  |  |  |  |  |  |
| Disagree | 51 (43.2%) | 341 (44.1%) | 182 (30.6%) | 123 (24.2%) | 230 (34.8%) | 587 (78.5%) | 86 (41.3%) | 125 (28.5%) | 1725 (42.6%) |
| Moderately disagree | 46 (39.0%) | 237 (30.7%) | 311 (52.3%) | 289 (56.8%) | 309 (46.7%) | 131 (17.5%) | 67 (32.2%) | 169 (38.5%) | 1559 (38.5%) |
| Unsure | 10 (8.5%) | 41 (5.3%) | 56 (9.4%) | 45 (8.8%) | 53 (8.0%) | 9 (1.2%) | 5 (2.4%) | 23 (5.2%) | 242 (6.0%) |
| Moderately agree | 11 (9.3%) | 117 (15.1%) | 44 (7.4%) | 49 (9.6%) | 51 (7.7%) | 12 (1.6%) | 31 (14.9%) | 98 (22.3%) | 413 (10.2%) |
| Agree | 0 (0%) | 37 (4.8%) | 2 (0.3%) | 3 (0.6%) | 18 (2.7%) | 9 (1.2%) | 19 (9.1%) | 24 (5.5%) | 112 (2.8%) |
| Frequently using pre-made mixes |  |  |  |  |  |  |  |  |  |
| Disagree | 67 (56.8%) | 505 (65.3%) | 290 (48.7%) | 164 (32.2%) | 405 (61.3%) | 629 (84.1%) | 136 (65.4%) | 322 (73.3%) | 2518 (62.2%) |
| Moderately disagree | 40 (33.9%) | 153 (19.8%) | 223 (37.5%) | 261 (51.3%) | 199 (30.1%) | 91 (12.2%) | 50 (24.0%) | 87 (19.8%) | 1104 (27.3%) |
| Unsure | 6 (5.1%) | 34 (4.4%) | 43 (7.2%) | 33 (6.5%) | 28 (4.2%) | 5 (0.7%) | 3 (1.4%) | 9 (2.1%) | 161 (4.0%) |
| Moderately agree | 4 (3.4%) | 59 (7.6%) | 36 (6.1%) | 47 (9.2%) | 23 (3.5%) | 20 (2.7%) | 12 (5.8%) | 17 (3.9%) | 218 (5.4%) |
| Agree | 1 (0.8%) | 22 (2.8%) | 3 (0.5%) | 4 (0.8%) | 6 (0.9%) | 3 (0.4%) | 7 (3.4%) | 4 (0.9%) | 50 (1.2%) |
| Having children help in the kitchen |  |  |  |  |  |  |  |  |  |
| Disagree | 6 (5.1%) | 100 (12.9%) | 56 (9.4%) | 25 (4.9%) | 50 (7.6%) | 236 (31.6%) | 21 (10.1%) | 32 (7.3%) | 526 (13.0%) |
| Moderately disagree | 36 (30.5%) | 105 (13.6%) | 164 (27.6%) | 147 (28.9%) | 91 (13.8%) | 76 (10.2%) | 43 (20.7%) | 112 (25.5%) | 774 (19.1%) |
| Unsure | 13 (11.0%) | 61 (7.9%) | 86 (14.5%) | 33 (6.5%) | 46 (7.0%) | 36 (4.8%) | 6 (2.9%) | 28 (6.4%) | 309 (7.6%) |
| Moderately agree | 53 (44.9%) | 279 (36.1%) | 229 (38.5%) | 217 (42.6%) | 246 (37.2%) | 260 (34.8%) | 94 (45.2%) | 196 (44.6%) | 1574 (38.9%) |
| Agree | 10 (8.5%) | 228 (29.5%) | 60 (10.1%) | 87 (17.1%) | 228 (34.5%) | 140 (18.7%) | 44 (21.2%) | 71 (16.2%) | 868 (21.4%) |

# 4. Analysis

Check for multicollinearity

```
model_mc <- lm(hds_T3~income_cat_T3+isced_cat2011_T3+migration+singlepar+unemploy, data=subset)

vif(model_mc)
```

```
##    income_cat_T3 isced_cat2011_T3        migration        singlepar 
##         1.412247         1.264254         1.022167         1.057336 
##         unemploy 
##         1.074787
```

## 4.1 Path analysis

We performed regression analysis for the association between
socioeconomic factors / vulnerabilities and consumer attitudes (path a),
the association between consumer attitudes and HDAS (path b) and the
association between socioeconomic factors / vulnerabilites and HDAS
(total effect, path c). All models are adjusted for age, sex and
BMI.

```
require(MASS)
```

### 4.1.2 Path a: Socioeconomic factors -> Consumer attitudes

```
# Consumer attitudes as factors

subset$foodst_01_T3 <- relevel(as.factor(subset$foodst_01_T3), ref = 1)
subset$foodst_02_T3 <- relevel(as.factor(subset$foodst_02_T3), ref = 1)
subset$foodst_03_T3 <- relevel(as.factor(subset$foodst_03_T3), ref = 1)
subset$foodst_04_T3 <- relevel(as.factor(subset$foodst_04_T3), ref = 1)
subset$foodst_05_T3 <- relevel(as.factor(subset$foodst_05_T3), ref = 1)
subset$foodst_06_T3 <- relevel(as.factor(subset$foodst_06_T3), ref = 1)
subset$foodst_07_T3 <- relevel(as.factor(subset$foodst_07_T3), ref = 1)
subset$foodst_08_T3 <- relevel(as.factor(subset$foodst_08_T3), ref = 1)
```

```
# path a
# Education -> Consumer attitudes

edu1 <- polr(foodst_01_T3 ~ isced_cat2011_T3 + age_T3 + sex_T3 + bmi_T3, data = subset, Hess=TRUE)
summary(edu1)
```

```
## Call:
## polr(formula = foodst_01_T3 ~ isced_cat2011_T3 + age_T3 + sex_T3 + 
##     bmi_T3, data = subset, Hess = TRUE)
## 
## Coefficients:
##                      Value Std. Error t value
## isced_cat2011_T3  0.091438   0.050538  1.8093
## age_T3            0.009938   0.005331  1.8644
## sex_T3            0.048696   0.084497  0.5763
## bmi_T3           -0.007941   0.005585 -1.4218
## 
## Intercepts:
##     Value   Std. Error t value
## 1|2 -1.6008  0.3688    -4.3404
## 2|3 -0.4852  0.3670    -1.3222
## 3|4  0.1041  0.3667     0.2840
## 4|5  1.8132  0.3681     4.9260
## 
## Residual Deviance: 11749.68 
## AIC: 11765.68 
## (120 observations deleted due to missingness)
```

```
ctable_edu1 <- coef(summary(edu1))
p_edu1 <- pnorm(abs(ctable_edu1[, "t value"]), lower.tail = FALSE) * 2
(ctable_edu1 <- cbind(ctable_edu1, "p-value" =  round(p_edu1, 4)))
```

```
##                         Value  Std. Error    t value p-value
## isced_cat2011_T3  0.091438323 0.050538467  1.8092817  0.0704
## age_T3            0.009938352 0.005330591  1.8643997  0.0623
## sex_T3            0.048696142 0.084497066  0.5763057  0.5644
## bmi_T3           -0.007940528 0.005584733 -1.4218277  0.1551
## 1|2              -1.600762674 0.368803159 -4.3404256  0.0000
## 2|3              -0.485207184 0.366976417 -1.3221754  0.1861
## 3|4               0.104131360 0.366710256  0.2839609  0.7764
## 4|5               1.813249492 0.368097782  4.9259995  0.0000
```

```
(ci_edu1 <- confint(edu1, level=0.99375))
```

```
## Waiting for profiling to be done...
```

```
##                        0.3 %     99.7 %
## isced_cat2011_T3 -0.04673295 0.22970178
## age_T3           -0.00463682 0.02451905
## sex_T3           -0.18261350 0.27961389
## bmi_T3           -0.02320238 0.00734912
```

```
exp(cbind(OR = coef(edu1), ci_edu1))
```

```
##                         OR     0.3 %   99.7 %
## isced_cat2011_T3 1.0957492 0.9543422 1.258225
## age_T3           1.0099879 0.9953739 1.024822
## sex_T3           1.0499013 0.8330901 1.322619
## bmi_T3           0.9920909 0.9770647 1.007376
```

```
edu2 <- polr(foodst_02_T3 ~ isced_cat2011_T3 + age_T3 + sex_T3 + bmi_T3, data = subset, Hess=TRUE)
summary(edu2)
```

```
## Call:
## polr(formula = foodst_02_T3 ~ isced_cat2011_T3 + age_T3 + sex_T3 + 
##     bmi_T3, data = subset, Hess = TRUE)
## 
## Coefficients:
##                       Value Std. Error   t value
## isced_cat2011_T3 -3.671e-01   0.052844 -6.946003
## age_T3           -1.041e-05   0.005533 -0.001882
## sex_T3           -5.224e-02   0.087908 -0.594310
## bmi_T3            9.911e-03   0.005831  1.699554
## 
## Intercepts:
##     Value   Std. Error t value
## 1|2 -0.8536  0.3785    -2.2553
## 2|3  0.5104  0.3783     1.3492
## 3|4  1.2281  0.3791     3.2394
## 4|5  2.8219  0.3880     7.2725
## 
## Residual Deviance: 9912.099 
## AIC: 9928.099 
## (120 observations deleted due to missingness)
```

```
ctable_edu2 <- coef(summary(edu2))
p_edu2 <- pnorm(abs(ctable_edu2[, "t value"]), lower.tail = FALSE) * 2
(ctable_edu2 <- cbind(ctable_edu2, "p-value" =  round(p_edu2, 4)))
```

```
##                          Value  Std. Error      t value p-value
## isced_cat2011_T3 -3.670574e-01 0.052844409 -6.946002768  0.0000
## age_T3           -1.041159e-05 0.005533139 -0.001881679  0.9985
## sex_T3           -5.224481e-02 0.087908306 -0.594310335  0.5523
## bmi_T3            9.910583e-03 0.005831285  1.699553789  0.0892
## 1|2              -8.536021e-01 0.378479881 -2.255343418  0.0241
## 2|3               5.103684e-01 0.378266612  1.349229212  0.1773
## 3|4               1.228101e+00 0.379116241  3.239377385  0.0012
## 4|5               2.821854e+00 0.388015222  7.272535431  0.0000
```

```
(ci_edu2 <- confint(edu2, level=0.99375))
```

```
## Waiting for profiling to be done...
```

```
##                         0.3 %      99.7 %
## isced_cat2011_T3 -0.511638605 -0.22258179
## age_T3           -0.015153043  0.01511259
## sex_T3           -0.291800427  0.18918981
## bmi_T3           -0.006065622  0.02583794
```

```
exp(cbind(OR = coef(edu2), ci_edu2))
```

```
##                         OR     0.3 %    99.7 %
## isced_cat2011_T3 0.6927699 0.5995124 0.8004495
## age_T3           0.9999896 0.9849612 1.0152274
## sex_T3           0.9490965 0.7469176 1.2082703
## bmi_T3           1.0099599 0.9939527 1.0261746
```

```
edu3 <- polr(foodst_03_T3 ~ isced_cat2011_T3 + age_T3 + sex_T3 + bmi_T3, data = subset, Hess=TRUE)
summary(edu3)
```

```
## Call:
## polr(formula = foodst_03_T3 ~ isced_cat2011_T3 + age_T3 + sex_T3 + 
##     bmi_T3, data = subset, Hess = TRUE)
## 
## Coefficients:
##                     Value Std. Error t value
## isced_cat2011_T3  0.08707   0.051998   1.675
## age_T3            0.02219   0.005396   4.113
## sex_T3            0.30008   0.085407   3.513
## bmi_T3           -0.01107   0.005703  -1.942
## 
## Intercepts:
##     Value   Std. Error t value
## 1|2 -1.7408  0.3773    -4.6136
## 2|3 -0.4780  0.3722    -1.2844
## 3|4  0.3276  0.3717     0.8813
## 4|5  1.8572  0.3729     4.9801
## 
## Residual Deviance: 10502.87 
## AIC: 10518.87 
## (120 observations deleted due to missingness)
```

```
ctable_edu3 <- coef(summary(edu3))
p_edu3 <- pnorm(abs(ctable_edu3[, "t value"]), lower.tail = FALSE) * 2
(ctable_edu3 <- cbind(ctable_edu3, "p-value" =  round(p_edu3, 4)))
```

```
##                        Value  Std. Error    t value p-value
## isced_cat2011_T3  0.08707306 0.051997575  1.6745599  0.0940
## age_T3            0.02219129 0.005395885  4.1126317  0.0000
## sex_T3            0.30007813 0.085407234  3.5134979  0.0004
## bmi_T3           -0.01107335 0.005702560 -1.9418204  0.0522
## 1|2              -1.74076268 0.377309077 -4.6136252  0.0000
## 2|3              -0.47803727 0.372200841 -1.2843530  0.1990
## 3|4               0.32756755 0.371683264  0.8813083  0.3782
## 4|5               1.85716812 0.372919990  4.9800712  0.0000
```

```
(ci_edu3 <- confint(edu3, level=0.99375))
```

```
## Waiting for profiling to be done...
```

```
##                        0.3 %      99.7 %
## isced_cat2011_T3 -0.05518181 0.229241443
## age_T3            0.00745838 0.036971703
## sex_T3            0.06623416 0.533470865
## bmi_T3           -0.02664443 0.004552253
```

```
exp(cbind(OR = coef(edu3), ci_edu3))
```

```
##                         OR     0.3 %   99.7 %
## isced_cat2011_T3 1.0909764 0.9463131 1.257646
## age_T3           1.0224393 1.0074863 1.037664
## sex_T3           1.3499643 1.0684769 1.704839
## bmi_T3           0.9889877 0.9737074 1.004563
```

```
edu4 <- polr(foodst_04_T3 ~ isced_cat2011_T3 + age_T3 + sex_T3 + bmi_T3, data = subset, Hess=TRUE)
summary(edu4)
```

```
## Call:
## polr(formula = foodst_04_T3 ~ isced_cat2011_T3 + age_T3 + sex_T3 + 
##     bmi_T3, data = subset, Hess = TRUE)
## 
## Coefficients:
##                     Value Std. Error t value
## isced_cat2011_T3  0.03998   0.051490  0.7764
## age_T3            0.02745   0.005385  5.0982
## sex_T3            0.04184   0.084840  0.4932
## bmi_T3           -0.00467   0.005613 -0.8320
## 
## Intercepts:
##     Value   Std. Error t value
## 1|2 -1.2449  0.3717    -3.3495
## 2|3  0.0735  0.3696     0.1989
## 3|4  0.7076  0.3697     1.9140
## 4|5  2.3169  0.3714     6.2379
## 
## Residual Deviance: 11626.99 
## AIC: 11642.99 
## (120 observations deleted due to missingness)
```

```
ctable_edu4 <- coef(summary(edu4))
p_edu4 <- pnorm(abs(ctable_edu4[, "t value"]), lower.tail = FALSE) * 2
(ctable_edu4 <- cbind(ctable_edu4, "p-value" =  round(p_edu4, 4)))
```

```
##                         Value  Std. Error    t value p-value
## isced_cat2011_T3  0.039976344 0.051489972  0.7763909  0.4375
## age_T3            0.027451346 0.005384567  5.0981526  0.0000
## sex_T3            0.041843951 0.084839623  0.4932124  0.6219
## bmi_T3           -0.004670169 0.005613369 -0.8319725  0.4054
## 1|2              -1.244855288 0.371650614 -3.3495311  0.0008
## 2|3               0.073510270 0.369617754  0.1988819  0.8424
## 3|4               0.707645831 0.369726895  1.9139690  0.0556
## 4|5               2.316926769 0.371428899  6.2378743  0.0000
```

```
(ci_edu4 <- confint(edu4, level=0.99375))
```

```
## Waiting for profiling to be done...
```

```
##                        0.3 %     99.7 %
## isced_cat2011_T3 -0.10081851 0.18081736
## age_T3            0.01274421 0.04219584
## sex_T3           -0.19042073 0.27368701
## bmi_T3           -0.02001034 0.01069763
```

```
exp(cbind(OR = coef(edu4), ci_edu4))
```

```
##                         OR     0.3 %   99.7 %
## isced_cat2011_T3 1.0407862 0.9040971 1.198196
## age_T3           1.0278316 1.0128258 1.043099
## sex_T3           1.0427317 0.8266113 1.314803
## bmi_T3           0.9953407 0.9801885 1.010755
```

```
edu5 <- polr(foodst_05_T3 ~ isced_cat2011_T3 + age_T3 + sex_T3 + bmi_T3, data = subset, Hess=TRUE)
summary(edu5)
```

```
## Call:
## polr(formula = foodst_05_T3 ~ isced_cat2011_T3 + age_T3 + sex_T3 + 
##     bmi_T3, data = subset, Hess = TRUE)
## 
## Coefficients:
##                       Value Std. Error t value
## isced_cat2011_T3 -0.1075927   0.055925 -1.9239
## age_T3            0.0106768   0.005869  1.8193
## sex_T3            0.1355348   0.092450  1.4660
## bmi_T3            0.0009313   0.006156  0.1513
## 
## Intercepts:
##     Value   Std. Error t value
## 1|2 -3.3601  0.4141    -8.1142
## 2|3 -1.9680  0.4036    -4.8759
## 3|4 -1.5465  0.4026    -3.8418
## 4|5  0.0962  0.4014     0.2397
## 
## Residual Deviance: 8218.918 
## AIC: 8234.918 
## (120 observations deleted due to missingness)
```

```
ctable_edu5 <- coef(summary(edu5))
p_edu5 <- pnorm(abs(ctable_edu5[, "t value"]), lower.tail = FALSE) * 2
(ctable_edu5 <- cbind(ctable_edu5, "p-value" =  round(p_edu5, 4)))
```

```
##                          Value  Std. Error    t value p-value
## isced_cat2011_T3 -0.1075926622 0.055925046 -1.9238726  0.0544
## age_T3            0.0106767637 0.005868534  1.8193239  0.0689
## sex_T3            0.1355347750 0.092449939  1.4660342  0.1426
## bmi_T3            0.0009312951 0.006156020  0.1512820  0.8798
## 1|2              -3.3600800422 0.414099989 -8.1141756  0.0000
## 2|3              -1.9679804781 0.403611776 -4.8759243  0.0000
## 3|4              -1.5465192420 0.402551615 -3.8417912  0.0001
## 4|5               0.0962301563 0.401444818  0.2397095  0.8106
```

```
(ci_edu5 <- confint(edu5, level=0.99375))
```

```
## Waiting for profiling to be done...
```

```
##                        0.3 %     99.7 %
## isced_cat2011_T3 -0.26102249 0.04492039
## age_T3           -0.00534058 0.02676261
## sex_T3           -0.11910227 0.38681551
## bmi_T3           -0.01581825 0.01786904
```

```
exp(cbind(OR = coef(edu5), ci_edu5))
```

```
##                         OR     0.3 %   99.7 %
## isced_cat2011_T3 0.8979933 0.7702636 1.045945
## age_T3           1.0107340 0.9946737 1.027124
## sex_T3           1.1451490 0.8877170 1.472285
## bmi_T3           1.0009317 0.9843062 1.018030
```

```
edu6 <- polr(foodst_06_T3 ~ isced_cat2011_T3 + age_T3 + sex_T3 + bmi_T3, data = subset, Hess=TRUE)
summary(edu6)
```

```
## Call:
## polr(formula = foodst_06_T3 ~ isced_cat2011_T3 + age_T3 + sex_T3 + 
##     bmi_T3, data = subset, Hess = TRUE)
## 
## Coefficients:
##                     Value Std. Error t value
## isced_cat2011_T3  0.44755   0.053972  8.2923
## age_T3           -0.02691   0.005563 -4.8379
## sex_T3           -0.33343   0.086985 -3.8332
## bmi_T3           -0.00548   0.005877 -0.9326
## 
## Intercepts:
##     Value   Std. Error t value
## 1|2 -1.0896  0.3819    -2.8530
## 2|3  0.7175  0.3817     1.8799
## 3|4  1.1676  0.3823     3.0541
## 4|5  2.8221  0.3913     7.2118
## 
## Residual Deviance: 9562.962 
## AIC: 9578.962 
## (120 observations deleted due to missingness)
```

```
ctable_edu6 <- coef(summary(edu6))
p_edu6 <- pnorm(abs(ctable_edu6[, "t value"]), lower.tail = FALSE) * 2
(ctable_edu6 <- cbind(ctable_edu6, "p-value" =  round(p_edu6, 4)))
```

```
##                         Value  Std. Error    t value p-value
## isced_cat2011_T3  0.447552787 0.053971949  8.2923222  0.0000
## age_T3           -0.026914149 0.005563196 -4.8378935  0.0000
## sex_T3           -0.333434984 0.086985061 -3.8332442  0.0001
## bmi_T3           -0.005480449 0.005876795 -0.9325574  0.3510
## 1|2              -1.089561110 0.381898171 -2.8530147  0.0043
## 2|3               0.717496324 0.381661777  1.8799271  0.0601
## 3|4               1.167561589 0.382298299  3.0540591  0.0023
## 4|5               2.822052381 0.391311756  7.2117751  0.0000
```

```
(ci_edu6 <- confint(edu6, level=0.99375))
```

```
## Waiting for profiling to be done...
```

```
##                        0.3 %      99.7 %
## isced_cat2011_T3  0.30043715  0.59567642
## age_T3           -0.04216387 -0.01173394
## sex_T3           -0.57107457 -0.09520116
## bmi_T3           -0.02160535  0.01054729
```

```
exp(cbind(OR = coef(edu6), ci_edu6))
```

```
##                         OR     0.3 %    99.7 %
## isced_cat2011_T3 1.5644789 1.3504490 1.8142577
## age_T3           0.9734448 0.9587127 0.9883346
## sex_T3           0.7164585 0.5649181 0.9091900
## bmi_T3           0.9945345 0.9786264 1.0106031
```

```
edu7 <- polr(foodst_07_T3 ~ isced_cat2011_T3 + age_T3 + sex_T3 + bmi_T3, data = subset, Hess=TRUE)
summary(edu7)
```

```
## Call:
## polr(formula = foodst_07_T3 ~ isced_cat2011_T3 + age_T3 + sex_T3 + 
##     bmi_T3, data = subset, Hess = TRUE)
## 
## Coefficients:
##                     Value Std. Error t value
## isced_cat2011_T3  0.10912   0.057108   1.911
## age_T3           -0.02325   0.005996  -3.877
## sex_T3           -0.35063   0.091742  -3.822
## bmi_T3            0.01337   0.006251   2.139
## 
## Intercepts:
##     Value   Std. Error t value
## 1|2 -0.5207  0.4068    -1.2800
## 2|3  1.1376  0.4078     2.7895
## 3|4  1.6540  0.4094     4.0399
## 4|5  3.4256  0.4300     7.9660
## 
## Residual Deviance: 7770.595 
## AIC: 7786.595 
## (120 observations deleted due to missingness)
```

```
ctable_edu7 <- coef(summary(edu7))
p_edu7 <- pnorm(abs(ctable_edu7[, "t value"]), lower.tail = FALSE) * 2
(ctable_edu7 <- cbind(ctable_edu7, "p-value" =  round(p_edu7, 4)))
```

```
##                        Value  Std. Error   t value p-value
## isced_cat2011_T3  0.10911887 0.057107860  1.910750  0.0560
## age_T3           -0.02324836 0.005996308 -3.877112  0.0001
## sex_T3           -0.35062675 0.091742287 -3.821866  0.0001
## bmi_T3            0.01337050 0.006250810  2.139004  0.0324
## 1|2              -0.52070242 0.406812749 -1.279956  0.2006
## 2|3               1.13760163 0.407808338  2.789550  0.0053
## 3|4               1.65399818 0.409413292  4.039923  0.0001
## 4|5               3.42560734 0.430030921  7.965956  0.0000
```

```
(ci_edu7 <- confint(edu7, level=0.99375))
```

```
## Waiting for profiling to be done...
```

```
##                         0.3 %       99.7 %
## isced_cat2011_T3 -0.046542262  0.265877410
## age_T3           -0.039708828 -0.006905864
## sex_T3           -0.600312483 -0.098281432
## bmi_T3           -0.003817573  0.030385845
```

```
exp(cbind(OR = coef(edu7), ci_edu7))
```

```
##                         OR     0.3 %    99.7 %
## isced_cat2011_T3 1.1152949 0.9545242 1.3045751
## age_T3           0.9770198 0.9610692 0.9931179
## sex_T3           0.7042466 0.5486402 0.9063938
## bmi_T3           1.0134603 0.9961897 1.0308522
```

```
edu8 <- polr(foodst_08_T3 ~ isced_cat2011_T3 + age_T3 + sex_T3 + bmi_T3, data = subset, Hess=TRUE)
summary(edu8)
```

```
## Call:
## polr(formula = foodst_08_T3 ~ isced_cat2011_T3 + age_T3 + sex_T3 + 
##     bmi_T3, data = subset, Hess = TRUE)
## 
## Coefficients:
##                      Value Std. Error t value
## isced_cat2011_T3 -0.050052   0.051532 -0.9713
## age_T3            0.003353   0.005349  0.6268
## sex_T3            0.364969   0.084476  4.3204
## bmi_T3            0.012618   0.005592  2.2564
## 
## Intercepts:
##     Value   Std. Error t value
## 1|2 -0.8865  0.3692    -2.4012
## 2|3  0.2769  0.3684     0.7518
## 3|4  0.6117  0.3684     1.6603
## 4|5  2.3501  0.3702     6.3483
## 
## Residual Deviance: 11571.75 
## AIC: 11587.75 
## (120 observations deleted due to missingness)
```

```
ctable_edu8 <- coef(summary(edu8))
p_edu8 <- pnorm(abs(ctable_edu8[, "t value"]), lower.tail = FALSE) * 2
(ctable_edu8 <- cbind(ctable_edu8, "p-value" =  round(p_edu8, 4)))
```

```
##                         Value  Std. Error    t value p-value
## isced_cat2011_T3 -0.050052184 0.051531689 -0.9712894  0.3314
## age_T3            0.003352603 0.005348847  0.6267898  0.5308
## sex_T3            0.364969311 0.084475842  4.3203986  0.0000
## bmi_T3            0.012617638 0.005591845  2.2564358  0.0240
## 1|2              -0.886477965 0.369177834 -2.4012221  0.0163
## 2|3               0.276919209 0.368355726  0.7517712  0.4522
## 3|4               0.611679639 0.368411405  1.6603168  0.0969
## 4|5               2.350133174 0.370199170  6.3482940  0.0000
```

```
(ci_edu8 <- confint(edu8, level=0.99375))
```

```
## Waiting for profiling to be done...
```

```
##                         0.3 %     99.7 %
## isced_cat2011_T3 -0.191057399 0.09080663
## age_T3           -0.011274115 0.01798289
## sex_T3            0.133876358 0.59600004
## bmi_T3           -0.002656678 0.02793296
```

```
exp(cbind(OR = coef(edu8), ci_edu8))
```

```
##                         OR     0.3 %   99.7 %
## isced_cat2011_T3 0.9511798 0.8260852 1.095057
## age_T3           1.0033582 0.9887892 1.018146
## sex_T3           1.4404698 1.1432515 1.814845
## bmi_T3           1.0126976 0.9973468 1.028327
```

```
# path a
# Income -> Consumer attitudes

inc1 <- polr(foodst_01_T3 ~ income_cat_T3 + age_T3 + sex_T3 + bmi_T3, data = subset, Hess=TRUE)
summary(inc1)
```

```
## Call:
## polr(formula = foodst_01_T3 ~ income_cat_T3 + age_T3 + sex_T3 + 
##     bmi_T3, data = subset, Hess = TRUE)
## 
## Coefficients:
##                   Value Std. Error t value
## income_cat_T3 -0.006820   0.021305 -0.3201
## age_T3         0.008562   0.005349  1.6007
## sex_T3         0.012033   0.084429  0.1425
## bmi_T3        -0.008414   0.005657 -1.4873
## 
## Intercepts:
##     Value   Std. Error t value
## 1|2 -1.9784  0.3530    -5.6050
## 2|3 -0.8718  0.3510    -2.4841
## 3|4 -0.2829  0.3506    -0.8071
## 4|5  1.4446  0.3515     4.1095
## 
## Residual Deviance: 11422.58 
## AIC: 11438.58 
## (226 observations deleted due to missingness)
```

```
ctable_inc1 <- coef(summary(inc1))
p_inc1 <- pnorm(abs(ctable_inc1[, "t value"]), lower.tail = FALSE) * 2
(ctable_inc1 <- cbind(ctable_inc1, "p-value" =  round(p_inc1, 4)))
```

```
##                      Value  Std. Error    t value p-value
## income_cat_T3 -0.006819589 0.021304981 -0.3200937  0.7489
## age_T3         0.008562444 0.005349283  1.6006714  0.1094
## sex_T3         0.012033406 0.084428573  0.1425276  0.8867
## bmi_T3        -0.008414024 0.005657252 -1.4872989  0.1369
## 1|2           -1.978397566 0.352968965 -5.6050185  0.0000
## 2|3           -0.871783684 0.350950684 -2.4840632  0.0130
## 3|4           -0.282947223 0.350593611 -0.8070518  0.4196
## 4|5            1.444604460 0.351527232  4.1095094  0.0000
```

```
(ci_inc1 <- confint(inc1, level=0.99375))
```

```
## Waiting for profiling to be done...
```

```
##                      0.3 %     99.7 %
## income_cat_T3 -0.065095652 0.05143488
## age_T3        -0.006063394 0.02319495
## sex_T3        -0.219109910 0.24273888
## bmi_T3        -0.023874539 0.00707371
```

```
exp(cbind(OR = coef(inc1), ci_inc1))
```

```
##                      OR     0.3 %   99.7 %
## income_cat_T3 0.9932036 0.9369778 1.052781
## age_T3        1.0085992 0.9939550 1.023466
## sex_T3        1.0121061 0.8032334 1.274736
## bmi_T3        0.9916213 0.9764082 1.007099
```

```
inc2 <- polr(foodst_02_T3 ~ income_cat_T3 + age_T3 + sex_T3 + bmi_T3, data = subset, Hess=TRUE)
summary(inc2)
```

```
## Call:
## polr(formula = foodst_02_T3 ~ income_cat_T3 + age_T3 + sex_T3 + 
##     bmi_T3, data = subset, Hess = TRUE)
## 
## Coefficients:
##                   Value Std. Error t value
## income_cat_T3 -0.138494   0.022247 -6.2253
## age_T3        -0.005906   0.005560 -1.0621
## sex_T3        -0.067005   0.087915 -0.7622
## bmi_T3         0.012509   0.005913  2.1154
## 
## Intercepts:
##     Value   Std. Error t value
## 1|2 -0.5463  0.3627    -1.5063
## 2|3  0.8030  0.3627     2.2138
## 3|4  1.5006  0.3637     4.1261
## 4|5  3.1166  0.3736     8.3415
## 
## Residual Deviance: 9594.474 
## AIC: 9610.474 
## (226 observations deleted due to missingness)
```

```
ctable_inc2 <- coef(summary(inc2))
p_inc2 <- pnorm(abs(ctable_inc2[, "t value"]), lower.tail = FALSE) * 2
(ctable_inc2 <- cbind(ctable_inc2, "p-value" =  round(p_inc2, 4)))
```

```
##                     Value  Std. Error   t value p-value
## income_cat_T3 -0.13849355 0.022246791 -6.225327  0.0000
## age_T3        -0.00590597 0.005560495 -1.062130  0.2882
## sex_T3        -0.06700520 0.087915213 -0.762157  0.4460
## bmi_T3         0.01250916 0.005913420  2.115386  0.0344
## 1|2           -0.54627087 0.362656169 -1.506305  0.1320
## 2|3            0.80296942 0.362705927  2.213830  0.0268
## 3|4            1.50057070 0.363681065  4.126062  0.0000
## 4|5            3.11655708 0.373619188  8.341534  0.0000
```

```
(ci_inc2 <- confint(inc2, level=0.99375))
```

```
## Waiting for profiling to be done...
```

```
##                      0.3 %      99.7 %
## income_cat_T3 -0.199435129 -0.07774578
## age_T3        -0.021129585  0.00928617
## sex_T3        -0.306600214  0.17442550
## bmi_T3        -0.003694691  0.02865998
```

```
exp(cbind(OR = coef(inc2), ci_inc2))
```

```
##                      OR     0.3 %    99.7 %
## income_cat_T3 0.8706689 0.8191934 0.9251996
## age_T3        0.9941114 0.9790921 1.0093294
## sex_T3        0.9351903 0.7359448 1.1905620
## bmi_T3        1.0125877 0.9963121 1.0290746
```

```
inc3 <- polr(foodst_03_T3 ~ income_cat_T3 + age_T3 + sex_T3 + bmi_T3, data = subset, Hess=TRUE)
summary(inc3)
```

```
## Call:
## polr(formula = foodst_03_T3 ~ income_cat_T3 + age_T3 + sex_T3 + 
##     bmi_T3, data = subset, Hess = TRUE)
## 
## Coefficients:
##                  Value Std. Error t value
## income_cat_T3 -0.03566   0.021795  -1.636
## age_T3         0.02240   0.005444   4.114
## sex_T3         0.26987   0.085429   3.159
## bmi_T3        -0.01544   0.005804  -2.661
## 
## Intercepts:
##     Value   Std. Error t value
## 1|2 -2.2394  0.3623    -6.1804
## 2|3 -0.9732  0.3566    -2.7292
## 3|4 -0.1708  0.3559    -0.4800
## 4|5  1.3637  0.3567     3.8234
## 
## Residual Deviance: 10202.30 
## AIC: 10218.30 
## (226 observations deleted due to missingness)
```

```
ctable_inc3 <- coef(summary(inc3))
p_inc3 <- pnorm(abs(ctable_inc3[, "t value"]), lower.tail = FALSE) * 2
(ctable_inc3 <- cbind(ctable_inc3, "p-value" =  round(p_inc3, 4)))
```

```
##                     Value  Std. Error    t value p-value
## income_cat_T3 -0.03565796 0.021795492 -1.6360244  0.1018
## age_T3         0.02239599 0.005444416  4.1135703  0.0000
## sex_T3         0.26986511 0.085428754  3.1589494  0.0016
## bmi_T3        -0.01544226 0.005803502 -2.6608515  0.0078
## 1|2           -2.23935743 0.362330478 -6.1804280  0.0000
## 2|3           -0.97321571 0.356595446 -2.7291871  0.0063
## 3|4           -0.17082816 0.355878189 -0.4800186  0.6312
## 4|5            1.36366989 0.356666836  3.8233717  0.0001
```

```
(ci_inc3 <- confint(inc3, level=0.99375))
```

```
## Waiting for profiling to be done...
```

```
##                     0.3 %       99.7 %
## income_cat_T3 -0.09530923 0.0239082631
## age_T3         0.00753342 0.0373126174
## sex_T3         0.03594088 0.5032927956
## bmi_T3        -0.03129550 0.0004535244
```

```
exp(cbind(OR = coef(inc3), ci_inc3))
```

```
##                      OR     0.3 %   99.7 %
## income_cat_T3 0.9649703 0.9090918 1.024196
## age_T3        1.0226487 1.0075619 1.038017
## sex_T3        1.3097878 1.0365946 1.654159
## bmi_T3        0.9846764 0.9691891 1.000454
```

```
inc4 <- polr(foodst_04_T3 ~ income_cat_T3 + age_T3 + sex_T3 + bmi_T3, data = subset, Hess=TRUE)
summary(inc4)
```

```
## Call:
## polr(formula = foodst_04_T3 ~ income_cat_T3 + age_T3 + sex_T3 + 
##     bmi_T3, data = subset, Hess = TRUE)
## 
## Coefficients:
##                   Value Std. Error t value
## income_cat_T3 -0.086625   0.021375 -4.0526
## age_T3         0.028524   0.005415  5.2672
## sex_T3        -0.002181   0.084846 -0.0257
## bmi_T3        -0.010322   0.005708 -1.8082
## 
## Intercepts:
##     Value   Std. Error t value
## 1|2 -1.7964  0.3558    -5.0488
## 2|3 -0.4902  0.3532    -1.3878
## 3|4  0.1510  0.3530     0.4277
## 4|5  1.7640  0.3543     4.9794
## 
## Residual Deviance: 11302.07 
## AIC: 11318.07 
## (226 observations deleted due to missingness)
```

```
ctable_inc4 <- coef(summary(inc4))
p_inc4 <- pnorm(abs(ctable_inc4[, "t value"]), lower.tail = FALSE) * 2
(ctable_inc4 <- cbind(ctable_inc4, "p-value" =  round(p_inc4, 4)))
```

```
##                      Value  Std. Error     t value p-value
## income_cat_T3 -0.086624858 0.021375264 -4.05257479  0.0001
## age_T3         0.028524413 0.005415438  5.26723997  0.0000
## sex_T3        -0.002180812 0.084846004 -0.02570318  0.9795
## bmi_T3        -0.010321558 0.005708348 -1.80815147  0.0706
## 1|2           -1.796422110 0.355810710 -5.04881404  0.0000
## 2|3           -0.490180642 0.353210556 -1.38778594  0.1652
## 3|4            0.150992266 0.353039056  0.42769281  0.6689
## 4|5            1.763998563 0.354256445  4.97943958  0.0000
```

```
(ci_inc4 <- confint(inc4, level=0.99375))
```

```
## Waiting for profiling to be done...
```

```
##                     0.3 %      99.7 %
## income_cat_T3 -0.14513834 -0.02822309
## age_T3         0.01373679  0.04335757
## sex_T3        -0.23445627  0.22968500
## bmi_T3        -0.02592380  0.00530397
```

```
exp(cbind(OR = coef(inc4), ci_inc4))
```

```
##                      OR     0.3 %    99.7 %
## income_cat_T3 0.9170210 0.8649026 0.9721715
## age_T3        1.0289351 1.0138316 1.0443112
## sex_T3        0.9978216 0.7910008 1.2582036
## bmi_T3        0.9897315 0.9744093 1.0053181
```

```
inc5 <- polr(foodst_05_T3 ~ income_cat_T3 + age_T3 + sex_T3 + bmi_T3, data = subset, Hess=TRUE)
summary(inc5)
```

```
## Call:
## polr(formula = foodst_05_T3 ~ income_cat_T3 + age_T3 + sex_T3 + 
##     bmi_T3, data = subset, Hess = TRUE)
## 
## Coefficients:
##                    Value Std. Error  t value
## income_cat_T3 -0.0827307   0.023360 -3.54149
## age_T3         0.0076358   0.005908  1.29243
## sex_T3         0.1020467   0.092343  1.10508
## bmi_T3         0.0003209   0.006251  0.05134
## 
## Intercepts:
##     Value   Std. Error t value
## 1|2 -3.5534  0.3984    -8.9185
## 2|3 -2.1591  0.3872    -5.5765
## 3|4 -1.7321  0.3860    -4.4868
## 4|5 -0.1017  0.3847    -0.2642
## 
## Residual Deviance: 8004.392 
## AIC: 8020.392 
## (226 observations deleted due to missingness)
```

```
ctable_inc5 <- coef(summary(inc5))
p_inc5 <- pnorm(abs(ctable_inc5[, "t value"]), lower.tail = FALSE) * 2
(ctable_inc5 <- cbind(ctable_inc5, "p-value" =  round(p_inc5, 4)))
```

```
##                       Value  Std. Error     t value p-value
## income_cat_T3 -0.0827306521 0.023360440 -3.54148518  0.0004
## age_T3         0.0076357735 0.005908083  1.29242830  0.1962
## sex_T3         0.1020466612 0.092343438  1.10507756  0.2691
## bmi_T3         0.0003209228 0.006251465  0.05133562  0.9591
## 1|2           -3.5534075150 0.398431539 -8.91848955  0.0000
## 2|3           -2.1591417676 0.387186545 -5.57648966  0.0000
## 3|4           -1.7320868667 0.386043885 -4.48676156  0.0000
## 4|5           -0.1016551312 0.384739574 -0.26421803  0.7916
```

```
(ci_inc5 <- confint(inc5, level=0.99375))
```

```
## Waiting for profiling to be done...
```

```
##                      0.3 %      99.7 %
## income_cat_T3 -0.146740031 -0.01895159
## age_T3        -0.008491954  0.02382696
## sex_T3        -0.152243534  0.35308108
## bmi_T3        -0.016685518  0.01752532
```

```
exp(cbind(OR = coef(inc5), ci_inc5))
```

```
##                      OR     0.3 %    99.7 %
## income_cat_T3 0.9205991 0.8635184 0.9812269
## age_T3        1.0076650 0.9915440 1.0241131
## sex_T3        1.1074351 0.8587791 1.4234465
## bmi_T3        1.0003210 0.9834529 1.0176798
```

```
inc6 <- polr(foodst_06_T3 ~ income_cat_T3 + age_T3 + sex_T3 + bmi_T3, data = subset, Hess=TRUE)
summary(inc6)
```

```
## Call:
## polr(formula = foodst_06_T3 ~ income_cat_T3 + age_T3 + sex_T3 + 
##     bmi_T3, data = subset, Hess = TRUE)
## 
## Coefficients:
##                   Value Std. Error t value
## income_cat_T3  0.170060   0.022281   7.632
## age_T3        -0.020352   0.005567  -3.656
## sex_T3        -0.333059   0.086558  -3.848
## bmi_T3        -0.006149   0.005942  -1.035
## 
## Intercepts:
##     Value   Std. Error t value
## 1|2 -1.4395  0.3639    -3.9557
## 2|3  0.3561  0.3632     0.9806
## 3|4  0.8114  0.3638     2.2307
## 4|5  2.5106  0.3737     6.7185
## 
## Residual Deviance: 9379.917 
## AIC: 9395.917 
## (226 observations deleted due to missingness)
```

```
ctable_inc6 <- coef(summary(inc6))
p_inc6 <- pnorm(abs(ctable_inc6[, "t value"]), lower.tail = FALSE) * 2
(ctable_inc6 <- cbind(ctable_inc6, "p-value" =  round(p_inc6, 4)))
```

```
##                      Value  Std. Error    t value p-value
## income_cat_T3  0.170059909 0.022281134  7.6324622  0.0000
## age_T3        -0.020351863 0.005567386 -3.6555507  0.0003
## sex_T3        -0.333059265 0.086558137 -3.8478100  0.0001
## bmi_T3        -0.006149077 0.005942326 -1.0347931  0.3008
## 1|2           -1.439534486 0.363918331 -3.9556526  0.0001
## 2|3            0.356117419 0.363170885  0.9805781  0.3268
## 3|4            0.811411869 0.363750959  2.2306797  0.0257
## 4|5            2.510579525 0.373680681  6.7185157  0.0000
```

```
(ci_inc6 <- confint(inc6, level=0.99375))
```

```
## Waiting for profiling to be done...
```

```
##                     0.3 %       99.7 %
## income_cat_T3  0.10926407  0.231139842
## age_T3        -0.03560932 -0.005156541
## sex_T3        -0.56957434 -0.096041078
## bmi_T3        -0.02244974  0.010061936
```

```
exp(cbind(OR = coef(inc6), ci_inc6))
```

```
##                      OR     0.3 %    99.7 %
## income_cat_T3 1.1853759 1.1154569 1.2600354
## age_T3        0.9798538 0.9650172 0.9948567
## sex_T3        0.7167277 0.5657662 0.9084267
## bmi_T3        0.9938698 0.9778004 1.0101127
```

```
inc7 <- polr(foodst_07_T3 ~ income_cat_T3 + age_T3 + sex_T3 + bmi_T3, data = subset, Hess=TRUE)
summary(inc7)
```

```
## Call:
## polr(formula = foodst_07_T3 ~ income_cat_T3 + age_T3 + sex_T3 + 
##     bmi_T3, data = subset, Hess = TRUE)
## 
## Coefficients:
##                  Value Std. Error t value
## income_cat_T3  0.07786   0.023987   3.246
## age_T3        -0.02107   0.006025  -3.497
## sex_T3        -0.30721   0.091777  -3.347
## bmi_T3         0.01645   0.006328   2.600
## 
## Intercepts:
##     Value   Std. Error t value
## 1|2 -0.2909  0.3903    -0.7454
## 2|3  1.3522  0.3914     3.4544
## 3|4  1.8674  0.3932     4.7497
## 4|5  3.6455  0.4152     8.7803
## 
## Residual Deviance: 7564.081 
## AIC: 7580.081 
## (226 observations deleted due to missingness)
```

```
ctable_inc7 <- coef(summary(inc7))
p_inc7 <- pnorm(abs(ctable_inc7[, "t value"]), lower.tail = FALSE) * 2
(ctable_inc7 <- cbind(ctable_inc7, "p-value" =  round(p_inc7, 4)))
```

```
##                     Value  Std. Error    t value p-value
## income_cat_T3  0.07785923 0.023986991  3.2458939  0.0012
## age_T3        -0.02107233 0.006025159 -3.4973900  0.0005
## sex_T3        -0.30721183 0.091776828 -3.3473790  0.0008
## bmi_T3         0.01645370 0.006327668  2.6002780  0.0093
## 1|2           -0.29089088 0.390259263 -0.7453785  0.4560
## 2|3            1.35221107 0.391445380  3.4544055  0.0006
## 3|4            1.86739516 0.393163855  4.7496613  0.0000
## 4|5            3.64553908 0.415193347  8.7803408  0.0000
```

```
(ci_inc7 <- confint(inc7, level=0.99375))
```

```
## Waiting for profiling to be done...
```

```
##                       0.3 %       99.7 %
## income_cat_T3  0.0123727634  0.143591233
## age_T3        -0.0376085431 -0.004647152
## sex_T3        -0.5569612135 -0.054745642
## bmi_T3        -0.0009435682  0.033681321
```

```
exp(cbind(OR = coef(inc7), ci_inc7))
```

```
##                      OR     0.3 %    99.7 %
## income_cat_T3 1.0809705 1.0124496 1.1544121
## age_T3        0.9791481 0.9630899 0.9953636
## sex_T3        0.7354948 0.5729475 0.9467259
## bmi_T3        1.0165898 0.9990569 1.0342550
```

```
inc8 <- polr(foodst_08_T3 ~ income_cat_T3 + age_T3 + sex_T3 + bmi_T3, data = subset, Hess=TRUE)
summary(inc8)
```

```
## Call:
## polr(formula = foodst_08_T3 ~ income_cat_T3 + age_T3 + sex_T3 + 
##     bmi_T3, data = subset, Hess = TRUE)
## 
## Coefficients:
##                   Value Std. Error t value
## income_cat_T3 -0.056197   0.021520 -2.6114
## age_T3         0.001453   0.005371  0.2706
## sex_T3         0.329620   0.084563  3.8979
## bmi_T3         0.010945   0.005667  1.9313
## 
## Intercepts:
##     Value   Std. Error t value
## 1|2 -1.1596  0.3531    -3.2840
## 2|3  0.0181  0.3519     0.0513
## 3|4  0.3590  0.3519     1.0201
## 4|5  2.1033  0.3538     5.9457
## 
## Residual Deviance: 11244.30 
## AIC: 11260.30 
## (226 observations deleted due to missingness)
```

```
ctable_inc8 <- coef(summary(inc8))
p_inc8 <- pnorm(abs(ctable_inc8[, "t value"]), lower.tail = FALSE) * 2
(ctable_inc8 <- cbind(ctable_inc8, "p-value" =  round(p_inc8, 4)))
```

```
##                      Value Std. Error     t value p-value
## income_cat_T3 -0.056196614 0.02151969 -2.61140443  0.0090
## age_T3         0.001453217 0.00537064  0.27058539  0.7867
## sex_T3         0.329620066 0.08456285  3.89792999  0.0001
## bmi_T3         0.010945036 0.00566716  1.93130872  0.0534
## 1|2           -1.159643402 0.35311527 -3.28403639  0.0010
## 2|3            0.018063244 0.35189223  0.05133175  0.9591
## 3|4            0.358986158 0.35192639  1.02006035  0.3077
## 4|5            2.103314122 0.35375279  5.94571738  0.0000
```

```
(ci_inc8 <- confint(inc8, level=0.99375))
```

```
## Waiting for profiling to be done...
```

```
##                      0.3 %      99.7 %
## income_cat_T3 -0.115079727 0.002625074
## age_T3        -0.013225470 0.016150881
## sex_T3         0.098301778 0.560895014
## bmi_T3        -0.004537679 0.026464545
```

```
exp(cbind(OR = coef(inc8), ci_inc8))
```

```
##                      OR     0.3 %   99.7 %
## income_cat_T3 0.9453532 0.8912951 1.002629
## age_T3        1.0014543 0.9868616 1.016282
## sex_T3        1.3904398 1.1032957 1.752240
## bmi_T3        1.0110052 0.9954726 1.026818
```

```
# path a
# Migration background -> Consumer attitudes

mig1 <- polr(foodst_01_T3 ~ migration + age_T3 + sex_T3 + bmi_T3, data = subset, Hess=TRUE)
summary(mig1)
```

```
## Call:
## polr(formula = foodst_01_T3 ~ migration + age_T3 + sex_T3 + bmi_T3, 
##     data = subset, Hess = TRUE)
## 
## Coefficients:
##               Value Std. Error t value
## migration  0.181742   0.085591  2.1234
## age_T3     0.010845   0.005194  2.0879
## sex_T3     0.054131   0.082561  0.6556
## bmi_T3    -0.009229   0.005408 -1.7065
## 
## Intercepts:
##     Value   Std. Error t value
## 1|2 -1.6004  0.3458    -4.6277
## 2|3 -0.4966  0.3440    -1.4435
## 3|4  0.0909  0.3438     0.2643
## 4|5  1.7940  0.3451     5.1983
## 
## Residual Deviance: 12108.49 
## AIC: 12124.49
```

```
ctable_mig1 <- coef(summary(mig1))
p_mig1 <- pnorm(abs(ctable_mig1[, "t value"]), lower.tail = FALSE) * 2
(ctable_mig1 <- cbind(ctable_mig1, "p-value" =  round(p_mig1, 4)))
```

```
##                  Value  Std. Error    t value p-value
## migration  0.181741624 0.085591422  2.1233626  0.0337
## age_T3     0.010845490 0.005194448  2.0879003  0.0368
## sex_T3     0.054130582 0.082561400  0.6556403  0.5121
## bmi_T3    -0.009228956 0.005408269 -1.7064530  0.0879
## 1|2       -1.600412630 0.345833875 -4.6276919  0.0000
## 2|3       -0.496615073 0.344043119 -1.4434675  0.1489
## 3|4        0.090866712 0.343767875  0.2643258  0.7915
## 4|5        1.793987131 0.345107051  5.1983497  0.0000
```

```
(ci_mig1 <- confint(mig1, level=0.99375))
```

```
## Waiting for profiling to be done...
```

```
##                  0.3 %      99.7 %
## migration -0.051978122 0.416259389
## age_T3    -0.003356617 0.025054688
## sex_T3    -0.171867804 0.279764036
## bmi_T3    -0.024009957 0.005576048
```

```
exp(cbind(OR = coef(mig1), ci_mig1))
```

```
##                  OR     0.3 %   99.7 %
## migration 1.1993043 0.9493496 1.516279
## age_T3    1.0109045 0.9966490 1.025371
## sex_T3    1.0556224 0.8420905 1.322818
## bmi_T3    0.9908135 0.9762760 1.005592
```

```
mig2 <- polr(foodst_02_T3 ~ migration + age_T3 + sex_T3 + bmi_T3, data = subset, Hess=TRUE)
summary(mig2)
```

```
## Call:
## polr(formula = foodst_02_T3 ~ migration + age_T3 + sex_T3 + bmi_T3, 
##     data = subset, Hess = TRUE)
## 
## Coefficients:
##                Value Std. Error   t value
## migration  0.1442818   0.089112  1.619111
## age_T3    -0.0038423   0.005397 -0.711920
## sex_T3     0.0003707   0.085541  0.004333
## bmi_T3     0.0191903   0.005622  3.413314
## 
## Intercepts:
##     Value   Std. Error t value
## 1|2  0.4109  0.3538     1.1615
## 2|3  1.7467  0.3546     4.9260
## 3|4  2.4429  0.3559     6.8638
## 4|5  4.0227  0.3655    11.0054
## 
## Residual Deviance: 10275.54 
## AIC: 10291.54
```

```
ctable_mig2 <- coef(summary(mig2))
p_mig2 <- pnorm(abs(ctable_mig2[, "t value"]), lower.tail = FALSE) * 2
(ctable_mig2 <- cbind(ctable_mig2, "p-value" =  round(p_mig2, 4)))
```

```
##                   Value  Std. Error     t value p-value
## migration  0.1442818240 0.089111742  1.61911124  0.1054
## age_T3    -0.0038423262 0.005397132 -0.71191995  0.4765
## sex_T3     0.0003706695 0.085541349  0.00433322  0.9965
## bmi_T3     0.0191902741 0.005622183  3.41331390  0.0006
## 1|2        0.4109004773 0.353766742  1.16150115  0.2454
## 2|3        1.7466636116 0.354583476  4.92595886  0.0000
## 3|4        2.4429348385 0.355914080  6.86383309  0.0000
## 4|5        4.0227294256 0.365524767 11.00535393  0.0000
```

```
(ci_mig2 <- confint(mig2, level=0.99375))
```

```
## Waiting for profiling to be done...
```

```
##                  0.3 %     99.7 %
## migration -0.100495636 0.38712292
## age_T3    -0.018615510 0.01090619
## sex_T3    -0.232700261 0.23533225
## bmi_T3     0.003797474 0.03455699
```

```
exp(cbind(OR = coef(mig2), ci_mig2))
```

```
##                 OR     0.3 %   99.7 %
## migration 1.155210 0.9043891 1.472738
## age_T3    0.996165 0.9815567 1.010966
## sex_T3    1.000371 0.7923910 1.265329
## bmi_T3    1.019376 1.0038047 1.035161
```

```
mig3 <- polr(foodst_03_T3 ~ migration + age_T3 + sex_T3 + bmi_T3, data = subset, Hess=TRUE)
summary(mig3)
```

```
## Call:
## polr(formula = foodst_03_T3 ~ migration + age_T3 + sex_T3 + bmi_T3, 
##     data = subset, Hess = TRUE)
## 
## Coefficients:
##              Value Std. Error t value
## migration  0.15249   0.088660   1.720
## age_T3     0.02174   0.005288   4.112
## sex_T3     0.31594   0.083433   3.787
## bmi_T3    -0.01441   0.005524  -2.608
## 
## Intercepts:
##     Value   Std. Error t value
## 1|2 -1.8686  0.3532    -5.2898
## 2|3 -0.5998  0.3478    -1.7245
## 3|4  0.2047  0.3471     0.5897
## 4|5  1.7229  0.3482     4.9475
## 
## Residual Deviance: 10817.00 
## AIC: 10833.00
```

```
ctable_mig3 <- coef(summary(mig3))
p_mig3 <- pnorm(abs(ctable_mig3[, "t value"]), lower.tail = FALSE) * 2
(ctable_mig3 <- cbind(ctable_mig3, "p-value" =  round(p_mig3, 4)))
```

```
##                 Value  Std. Error    t value p-value
## migration  0.15248643 0.088660211  1.7198969  0.0855
## age_T3     0.02174284 0.005287952  4.1117686  0.0000
## sex_T3     0.31593695 0.083432769  3.7867250  0.0002
## bmi_T3    -0.01440886 0.005524138 -2.6083460  0.0091
## 1|2       -1.86860557 0.353243875 -5.2898456  0.0000
## 2|3       -0.59975069 0.347775087 -1.7245361  0.0846
## 3|4        0.20466696 0.347076759  0.5896879  0.5554
## 4|5        1.72289619 0.348238400  4.9474618  0.0000
```

```
(ci_mig3 <- confint(mig3, level=0.99375))
```

```
## Waiting for profiling to be done...
```

```
##                  0.3 %       99.7 %
## migration -0.089152235 0.3959648342
## age_T3     0.007305595 0.0362281090
## sex_T3     0.087512565 0.5439403738
## bmi_T3    -0.029493137 0.0007273322
```

```
exp(cbind(OR = coef(mig3), ci_mig3))
```

```
##                  OR     0.3 %   99.7 %
## migration 1.1647267 0.9147063 1.485817
## age_T3    1.0219809 1.0073323 1.036892
## sex_T3    1.3715438 1.0914560 1.722782
## bmi_T3    0.9856944 0.9709375 1.000728
```

```
mig4 <- polr(foodst_04_T3 ~ migration + age_T3 + sex_T3 + bmi_T3, data = subset, Hess=TRUE)
summary(mig4)
```

```
## Call:
## polr(formula = foodst_04_T3 ~ migration + age_T3 + sex_T3 + bmi_T3, 
##     data = subset, Hess = TRUE)
## 
## Coefficients:
##               Value Std. Error t value
## migration  0.281580   0.086290  3.2632
## age_T3     0.027577   0.005252  5.2513
## sex_T3     0.067304   0.082938  0.8115
## bmi_T3    -0.005877   0.005437 -1.0809
## 
## Intercepts:
##     Value   Std. Error t value
## 1|2 -1.0151  0.3465    -2.9293
## 2|3  0.2950  0.3443     0.8569
## 3|4  0.9279  0.3444     2.6945
## 4|5  2.5311  0.3466     7.3021
## 
## Residual Deviance: 11958.34 
## AIC: 11974.34
```

```
ctable_mig4 <- coef(summary(mig4))
p_mig4 <- pnorm(abs(ctable_mig4[, "t value"]), lower.tail = FALSE) * 2
(ctable_mig4 <- cbind(ctable_mig4, "p-value" =  round(p_mig4, 4)))
```

```
##                 Value  Std. Error    t value p-value
## migration  0.28157995 0.086289995  3.2631818  0.0011
## age_T3     0.02757748 0.005251580  5.2512730  0.0000
## sex_T3     0.06730417 0.082938260  0.8114973  0.4171
## bmi_T3    -0.00587731 0.005437414 -1.0809016  0.2797
## 1|2       -1.01510190 0.346538508 -2.9292615  0.0034
## 2|3        0.29500011 0.344259014  0.8569132  0.3915
## 3|4        0.92793048 0.344376372  2.6945242  0.0070
## 4|5        2.53107553 0.346621243  7.3021362  0.0000
```

```
(ci_mig4 <- confint(mig4, level=0.99375))
```

```
## Waiting for profiling to be done...
```

```
##                 0.3 %      99.7 %
## migration  0.04596312 0.518038883
## age_T3     0.01323503 0.041959191
## sex_T3    -0.15971364 0.293988918
## bmi_T3    -0.02073456 0.009010957
```

```
exp(cbind(OR = coef(mig4), ci_mig4))
```

```
##                  OR     0.3 %   99.7 %
## migration 1.3252219 1.0470358 1.678732
## age_T3    1.0279613 1.0133230 1.042852
## sex_T3    1.0696208 0.8523878 1.341769
## bmi_T3    0.9941399 0.9794789 1.009052
```

```
mig5 <- polr(foodst_05_T3 ~ migration + age_T3 + sex_T3 + bmi_T3, data = subset, Hess=TRUE)
summary(mig5)
```

```
## Call:
## polr(formula = foodst_05_T3 ~ migration + age_T3 + sex_T3 + bmi_T3, 
##     data = subset, Hess = TRUE)
## 
## Coefficients:
##              Value Std. Error t value
## migration 0.035542   0.094868  0.3746
## age_T3    0.009063   0.005733  1.5808
## sex_T3    0.147833   0.090407  1.6352
## bmi_T3    0.003142   0.005981  0.5253
## 
## Intercepts:
##     Value   Std. Error t value
## 1|2 -3.0576  0.3880    -7.8797
## 2|3 -1.6627  0.3769    -4.4116
## 3|4 -1.2356  0.3757    -3.2886
## 4|5  0.3971  0.3749     1.0593
## 
## Residual Deviance: 8431.253 
## AIC: 8447.253
```

```
ctable_mig5 <- coef(summary(mig5))
p_mig5 <- pnorm(abs(ctable_mig5[, "t value"]), lower.tail = FALSE) * 2
(ctable_mig5 <- cbind(ctable_mig5, "p-value" =  round(p_mig5, 4)))
```

```
##                  Value  Std. Error    t value p-value
## migration  0.035541701 0.094868002  0.3746437  0.7079
## age_T3     0.009063196 0.005733329  1.5807913  0.1139
## sex_T3     0.147832795 0.090406963  1.6351926  0.1020
## bmi_T3     0.003142256 0.005981360  0.5253415  0.5993
## 1|2       -3.057643295 0.388039912 -7.8797134  0.0000
## 2|3       -1.662655119 0.376879272 -4.4116385  0.0000
## 3|4       -1.235604288 0.375724682 -3.2885896  0.0010
## 4|5        0.397142191 0.374897171  1.0593363  0.2894
```

```
(ci_mig5 <- confint(mig5, level=0.99375))
```

```
## Waiting for profiling to be done...
```

```
##                  0.3 %     99.7 %
## migration -0.221561370 0.29770373
## age_T3    -0.006591711 0.02477494
## sex_T3    -0.101127955 0.39359956
## bmi_T3    -0.013123304 0.01960777
```

```
exp(cbind(OR = coef(mig5), ci_mig5))
```

```
##                 OR     0.3 %   99.7 %
## migration 1.036181 0.8012667 1.346763
## age_T3    1.009104 0.9934300 1.025084
## sex_T3    1.159319 0.9038174 1.482307
## bmi_T3    1.003147 0.9869624 1.019801
```

```
mig6 <- polr(foodst_06_T3 ~ migration + age_T3 + sex_T3 + bmi_T3, data = subset, Hess=TRUE)
summary(mig6)
```

```
## Call:
## polr(formula = foodst_06_T3 ~ migration + age_T3 + sex_T3 + bmi_T3, 
##     data = subset, Hess = TRUE)
## 
## Coefficients:
##              Value Std. Error t value
## migration -0.27121   0.091143  -2.976
## age_T3    -0.01964   0.005393  -3.643
## sex_T3    -0.40447   0.084555  -4.783
## bmi_T3    -0.01257   0.005690  -2.210
## 
## Intercepts:
##     Value   Std. Error t value
## 1|2 -2.5050  0.3554    -7.0488
## 2|3 -0.7367  0.3532    -2.0858
## 3|4 -0.2850  0.3537    -0.8058
## 4|5  1.3726  0.3630     3.7813
## 
## Residual Deviance: 9933.728 
## AIC: 9949.728
```

```
ctable_mig6 <- coef(summary(mig6))
p_mig6 <- pnorm(abs(ctable_mig6[, "t value"]), lower.tail = FALSE) * 2
(ctable_mig6 <- cbind(ctable_mig6, "p-value" =  round(p_mig6, 4)))
```

```
##                 Value  Std. Error    t value p-value
## migration -0.27120905 0.091143181 -2.9756373  0.0029
## age_T3    -0.01964414 0.005392681 -3.6427408  0.0003
## sex_T3    -0.40446814 0.084555311 -4.7834741  0.0000
## bmi_T3    -0.01257458 0.005689574 -2.2101100  0.0271
## 1|2       -2.50503695 0.355384208 -7.0488134  0.0000
## 2|3       -0.73669005 0.353191069 -2.0858117  0.0370
## 3|4       -0.28501398 0.353691671 -0.8058261  0.4203
## 4|5        1.37259705 0.362993406  3.7813278  0.0002
```

```
(ci_mig6 <- confint(mig6, level=0.99375))
```

```
## Waiting for profiling to be done...
```

```
##                 0.3 %       99.7 %
## migration -0.52186508 -0.023111948
## age_T3    -0.03442076 -0.004924118
## sex_T3    -0.63555955 -0.172987545
## bmi_T3    -0.02819107  0.002937323
```

```
exp(cbind(OR = coef(mig6), ci_mig6))
```

```
##                  OR     0.3 %    99.7 %
## migration 0.7624571 0.5934128 0.9771531
## age_T3    0.9805476 0.9661649 0.9950880
## sex_T3    0.6673316 0.5296390 0.8411481
## bmi_T3    0.9875041 0.9722026 1.0029416
```

```
mig7 <- polr(foodst_07_T3 ~ migration + age_T3 + sex_T3 + bmi_T3, data = subset, Hess=TRUE)
summary(mig7)
```

```
## Call:
## polr(formula = foodst_07_T3 ~ migration + age_T3 + sex_T3 + bmi_T3, 
##     data = subset, Hess = TRUE)
## 
## Coefficients:
##              Value Std. Error t value
## migration -0.02699   0.096124 -0.2808
## age_T3    -0.02124   0.005853 -3.6286
## sex_T3    -0.34993   0.089619 -3.9046
## bmi_T3     0.01125   0.006073  1.8528
## 
## Intercepts:
##     Value   Std. Error t value
## 1|2 -0.7769  0.3794    -2.0478
## 2|3  0.8694  0.3801     2.2874
## 3|4  1.3850  0.3817     3.6281
## 4|5  3.1209  0.4024     7.7553
## 
## Residual Deviance: 7989.372 
## AIC: 8005.372
```

```
ctable_mig7 <- coef(summary(mig7))
p_mig7 <- pnorm(abs(ctable_mig7[, "t value"]), lower.tail = FALSE) * 2
(ctable_mig7 <- cbind(ctable_mig7, "p-value" =  round(p_mig7, 4)))
```

```
##                 Value  Std. Error    t value p-value
## migration -0.02699475 0.096124413 -0.2808313  0.7788
## age_T3    -0.02123737 0.005852691 -3.6286496  0.0003
## sex_T3    -0.34993122 0.089619428 -3.9046357  0.0001
## bmi_T3     0.01125106 0.006072598  1.8527595  0.0639
## 1|2       -0.77692773 0.379400658 -2.0477764  0.0406
## 2|3        0.86937485 0.380065290  2.2874355  0.0222
## 3|4        1.38497172 0.381737881  3.6280699  0.0003
## 4|5        3.12088536 0.402417607  7.7553400  0.0000
```

```
(ci_mig7 <- confint(mig7, level=0.99375))
```

```
## Waiting for profiling to be done...
```

```
##                 0.3 %       99.7 %
## migration -0.29291889  0.233246415
## age_T3    -0.03729851 -0.005281409
## sex_T3    -0.59382951 -0.103423042
## bmi_T3    -0.00545204  0.027776528
```

```
exp(cbind(OR = coef(mig7), ci_mig7))
```

```
##                  OR     0.3 %    99.7 %
## migration 0.9733664 0.7460827 1.2626926
## age_T3    0.9789866 0.9633885 0.9947325
## sex_T3    0.7047366 0.5522085 0.9017454
## bmi_T3    1.0113146 0.9945628 1.0281659
```

```
mig8 <- polr(foodst_08_T3 ~ migration + age_T3 + sex_T3 + bmi_T3, data = subset, Hess=TRUE)
summary(mig8)
```

```
## Call:
## polr(formula = foodst_08_T3 ~ migration + age_T3 + sex_T3 + bmi_T3, 
##     data = subset, Hess = TRUE)
## 
## Coefficients:
##              Value Std. Error t value
## migration 0.154663   0.086337  1.7914
## age_T3    0.001362   0.005225  0.2606
## sex_T3    0.367983   0.082546  4.4579
## bmi_T3    0.011921   0.005409  2.2038
## 
## Intercepts:
##     Value   Std. Error t value
## 1|2 -0.6880  0.3438    -2.0013
## 2|3  0.4683  0.3427     1.3665
## 3|4  0.8029  0.3429     2.3414
## 4|5  2.5294  0.3455     7.3203
## 
## Residual Deviance: 11924.12 
## AIC: 11940.12
```

```
ctable_mig8 <- coef(summary(mig8))
p_mig8 <- pnorm(abs(ctable_mig8[, "t value"]), lower.tail = FALSE) * 2
(ctable_mig8 <- cbind(ctable_mig8, "p-value" =  round(p_mig8, 4)))
```

```
##                  Value  Std. Error    t value p-value
## migration  0.154663185 0.086337057  1.7913882  0.0732
## age_T3     0.001361524 0.005224751  0.2605911  0.7944
## sex_T3     0.367982826 0.082545535  4.4579374  0.0000
## bmi_T3     0.011921068 0.005409434  2.2037550  0.0275
## 1|2       -0.687961515 0.343752304 -2.0013292  0.0454
## 2|3        0.468328721 0.342727628  1.3664750  0.1718
## 3|4        0.802880645 0.342906862  2.3413957  0.0192
## 4|5        2.529364855 0.345527209  7.3203059  0.0000
```

```
(ci_mig8 <- confint(mig8, level=0.99375))
```

```
## Waiting for profiling to be done...
```

```
##                  0.3 %     99.7 %
## migration -0.081222908 0.39109539
## age_T3    -0.012922132 0.01565563
## sex_T3     0.142199612 0.59375602
## bmi_T3    -0.002855227 0.02673648
```

```
exp(cbind(OR = coef(mig8), ci_mig8))
```

```
##                 OR     0.3 %   99.7 %
## migration 1.167265 0.9219881 1.478600
## age_T3    1.001362 0.9871610 1.015779
## sex_T3    1.444817 1.1528067 1.810777
## bmi_T3    1.011992 0.9971488 1.027097
```

```
# path a
# Unemployment in Household -> Consumer attitudes

une1 <- polr(foodst_01_T3 ~ unemploy + age_T3 + sex_T3 + bmi_T3, data = subset, Hess=TRUE)
summary(une1)
```

```
## Call:
## polr(formula = foodst_01_T3 ~ unemploy + age_T3 + sex_T3 + bmi_T3, 
##     data = subset, Hess = TRUE)
## 
## Coefficients:
##              Value Std. Error t value
## unemploy  0.024730   0.100763  0.2454
## age_T3    0.010897   0.005195  2.0976
## sex_T3    0.047524   0.082520  0.5759
## bmi_T3   -0.009034   0.005428 -1.6644
## 
## Intercepts:
##     Value   Std. Error t value
## 1|2 -1.7818  0.3457    -5.1538
## 2|3 -0.6785  0.3438    -1.9736
## 3|4 -0.0916  0.3434    -0.2667
## 4|5  1.6101  0.3445     4.6735
## 
## Residual Deviance: 12112.95 
## AIC: 12128.95
```

```
ctable_une1 <- coef(summary(une1))
p_une1 <- pnorm(abs(ctable_une1[, "t value"]), lower.tail = FALSE) * 2
(ctable_une1 <- cbind(ctable_une1, "p-value" =  round(p_une1, 4)))
```

```
##                 Value  Std. Error    t value p-value
## unemploy  0.024729790 0.100763443  0.2454242  0.8061
## age_T3    0.010897040 0.005195084  2.0975676  0.0359
## sex_T3    0.047524022 0.082519652  0.5759116  0.5647
## bmi_T3   -0.009034155 0.005428031 -1.6643522  0.0960
## 1|2      -1.781767042 0.345718214 -5.1538130  0.0000
## 2|3      -0.678549264 0.343814303 -1.9735923  0.0484
## 3|4      -0.091586103 0.343443594 -0.2666700  0.7897
## 4|5       1.610139830 0.344527864  4.6734677  0.0000
```

```
(ci_une1 <- confint(une1, level=0.99375))
```

```
## Waiting for profiling to be done...
```

```
##                 0.3 %      99.7 %
## unemploy -0.250405598 0.300916885
## age_T3   -0.003307678 0.025106969
## sex_T3   -0.178365557 0.273038078
## bmi_T3   -0.023869164 0.005824807
```

```
exp(cbind(OR = coef(une1), ci_une1))
```

```
##                 OR     0.3 %   99.7 %
## unemploy 1.0250381 0.7784850 1.351097
## age_T3   1.0109566 0.9966978 1.025425
## sex_T3   1.0486714 0.8366365 1.313950
## bmi_T3   0.9910065 0.9764135 1.005842
```

```
une2 <- polr(foodst_02_T3 ~ unemploy + age_T3 + sex_T3 + bmi_T3, data = subset, Hess=TRUE)
summary(une2)
```

```
## Call:
## polr(formula = foodst_02_T3 ~ unemploy + age_T3 + sex_T3 + bmi_T3, 
##     data = subset, Hess = TRUE)
## 
## Coefficients:
##              Value Std. Error  t value
## unemploy  0.097209   0.104458  0.93060
## age_T3   -0.003766   0.005398 -0.69781
## sex_T3   -0.006438   0.085532 -0.07527
## bmi_T3    0.019016   0.005642  3.37052
## 
## Intercepts:
##     Value   Std. Error t value
## 1|2  0.3406  0.3535     0.9633
## 2|3  1.6763  0.3543     4.7306
## 3|4  2.3723  0.3556     6.6705
## 4|5  3.9514  0.3651    10.8224
## 
## Residual Deviance: 10277.29 
## AIC: 10293.29
```

```
ctable_une2 <- coef(summary(une2))
p_une2 <- pnorm(abs(ctable_une2[, "t value"]), lower.tail = FALSE) * 2
(ctable_une2 <- cbind(ctable_une2, "p-value" =  round(p_une2, 4)))
```

```
##                 Value  Std. Error     t value p-value
## unemploy  0.097209282 0.104458293  0.93060378  0.3521
## age_T3   -0.003766443 0.005397541 -0.69780711  0.4853
## sex_T3   -0.006437898 0.085532304 -0.07526861  0.9400
## bmi_T3    0.019015691 0.005641776  3.37051509  0.0008
## 1|2       0.340560038 0.353519836  0.96334068  0.3354
## 2|3       1.676300341 0.354349048  4.73064723  0.0000
## 3|4       2.372302813 0.355641528  6.67048876  0.0000
## 4|5       3.951400818 0.365113789 10.82238178  0.0000
```

```
(ci_une2 <- confint(une2, level=0.99375))
```

```
## Waiting for profiling to be done...
```

```
##                 0.3 %     99.7 %
## unemploy -0.190303910 0.38145858
## age_T3   -0.018542186 0.01098176
## sex_T3   -0.239469204 0.22851357
## bmi_T3    0.003568907 0.03443532
```

```
exp(cbind(OR = coef(une2), ci_une2))
```

```
##                 OR     0.3 %   99.7 %
## unemploy 1.1020910 0.8267079 1.464419
## age_T3   0.9962406 0.9816287 1.011042
## sex_T3   0.9935828 0.7870455 1.256731
## bmi_T3   1.0191976 1.0035753 1.035035
```

```
une3 <- polr(foodst_03_T3 ~ unemploy + age_T3 + sex_T3 + bmi_T3, data = subset, Hess=TRUE)
summary(une3)
```

```
## Call:
## polr(formula = foodst_03_T3 ~ unemploy + age_T3 + sex_T3 + bmi_T3, 
##     data = subset, Hess = TRUE)
## 
## Coefficients:
##             Value Std. Error t value
## unemploy  0.16830   0.106223   1.584
## age_T3    0.02189   0.005284   4.143
## sex_T3    0.30693   0.083457   3.678
## bmi_T3   -0.01488   0.005545  -2.684
## 
## Intercepts:
##     Value   Std. Error t value
## 1|2 -1.8797  0.3537    -5.3146
## 2|3 -0.6111  0.3482    -1.7552
## 3|4  0.1930  0.3474     0.5555
## 4|5  1.7111  0.3485     4.9093
## 
## Residual Deviance: 10817.44 
## AIC: 10833.44
```

```
ctable_une3 <- coef(summary(une3))
p_une3 <- pnorm(abs(ctable_une3[, "t value"]), lower.tail = FALSE) * 2
(ctable_une3 <- cbind(ctable_une3, "p-value" =  round(p_une3, 4)))
```

```
##                Value  Std. Error   t value p-value
## unemploy  0.16830157 0.106222742  1.584421  0.1131
## age_T3    0.02189332 0.005284487  4.142942  0.0000
## sex_T3    0.30692733 0.083456865  3.677676  0.0002
## bmi_T3   -0.01488380 0.005545100 -2.684135  0.0073
## 1|2      -1.87967337 0.353681884 -5.314588  0.0000
## 2|3      -0.61109587 0.348154789 -1.755242  0.0792
## 3|4       0.19298095 0.347392944  0.555512  0.5785
## 4|5       1.71107197 0.348534897  4.909328  0.0000
```

```
(ci_une3 <- confint(une3, level=0.99375))
```

```
## Waiting for profiling to be done...
```

```
##                0.3 %      99.7 %
## unemploy -0.12088202 0.460511676
## age_T3    0.00746569 0.036369285
## sex_T3    0.07843332 0.534993000
## bmi_T3   -0.03002637 0.000308599
```

```
exp(cbind(OR = coef(une3), ci_une3))
```

```
##                 OR     0.3 %   99.7 %
## unemploy 1.1832934 0.8861385 1.584885
## age_T3   1.0221347 1.0074936 1.037039
## sex_T3   1.3592422 1.0815912 1.707436
## bmi_T3   0.9852264 0.9704199 1.000309
```

```
une4 <- polr(foodst_04_T3 ~ unemploy + age_T3 + sex_T3 + bmi_T3, data = subset, Hess=TRUE)
summary(une4)
```

```
## Call:
## polr(formula = foodst_04_T3 ~ unemploy + age_T3 + sex_T3 + bmi_T3, 
##     data = subset, Hess = TRUE)
## 
## Coefficients:
##              Value Std. Error t value
## unemploy  0.284269   0.102432  2.7752
## age_T3    0.027788   0.005252  5.2911
## sex_T3    0.053867   0.082955  0.6494
## bmi_T3   -0.006642   0.005465 -1.2153
## 
## Intercepts:
##     Value   Std. Error t value
## 1|2 -1.0580  0.3470    -3.0493
## 2|3  0.2519  0.3447     0.7308
## 3|4  0.8842  0.3448     2.5646
## 4|5  2.4859  0.3469     7.1658
## 
## Residual Deviance: 11961.30 
## AIC: 11977.30
```

```
ctable_une4 <- coef(summary(une4))
p_une4 <- pnorm(abs(ctable_une4[, "t value"]), lower.tail = FALSE) * 2
(ctable_une4 <- cbind(ctable_une4, "p-value" =  round(p_une4, 4)))
```

```
##                 Value  Std. Error    t value p-value
## unemploy  0.284269397 0.102432494  2.7751877  0.0055
## age_T3    0.027787997 0.005251802  5.2911358  0.0000
## sex_T3    0.053866899 0.082955098  0.6493501  0.5161
## bmi_T3   -0.006642202 0.005465408 -1.2153168  0.2242
## 1|2      -1.058048807 0.346986105 -3.0492541  0.0023
## 2|3       0.251892610 0.344692232  0.7307754  0.4649
## 3|4       0.884206041 0.344768160  2.5646395  0.0103
## 4|5       2.485891424 0.346911468  7.1657805  0.0000
```

```
(ci_une4 <- confint(une4, level=0.99375))
```

```
## Waiting for profiling to be done...
```

```
##                 0.3 %      99.7 %
## unemploy  0.004677695 0.565173140
## age_T3    0.013444217 0.042169550
## sex_T3   -0.173210705 0.280583344
## bmi_T3   -0.021577777 0.008320673
```

```
exp(cbind(OR = coef(une4), ci_une4))
```

```
##                 OR     0.3 %   99.7 %
## unemploy 1.3287909 1.0046887 1.759752
## age_T3   1.0281777 1.0135350 1.043071
## sex_T3   1.0553441 0.8409604 1.323902
## bmi_T3   0.9933798 0.9786534 1.008355
```

```
une5 <- polr(foodst_05_T3 ~ unemploy + age_T3 + sex_T3 + bmi_T3, data = subset, Hess=TRUE)
summary(une5)
```

```
## Call:
## polr(formula = foodst_05_T3 ~ unemploy + age_T3 + sex_T3 + bmi_T3, 
##     data = subset, Hess = TRUE)
## 
## Coefficients:
##             Value Std. Error t value
## unemploy 0.370465   0.117890  3.1425
## age_T3   0.009375   0.005741  1.6331
## sex_T3   0.140318   0.090489  1.5507
## bmi_T3   0.001744   0.006003  0.2906
## 
## Intercepts:
##     Value   Std. Error t value
## 1|2 -2.7371  0.3918    -6.9859
## 2|3 -1.3419  0.3808    -3.5237
## 3|4 -0.9147  0.3797    -2.4089
## 4|5  0.7207  0.3792     1.9008
## 
## Residual Deviance: 8421.132 
## AIC: 8437.132
```

```
ctable_une5 <- coef(summary(une5))
p_une5 <- pnorm(abs(ctable_une5[, "t value"]), lower.tail = FALSE) * 2
(ctable_une5 <- cbind(ctable_une5, "p-value" =  round(p_une5, 4)))
```

```
##                 Value  Std. Error    t value p-value
## unemploy  0.370465143 0.117889562  3.1424762  0.0017
## age_T3    0.009374915 0.005740631  1.6330809  0.1025
## sex_T3    0.140317628 0.090488620  1.5506660  0.1210
## bmi_T3    0.001744237 0.006003177  0.2905523  0.7714
## 1|2      -2.737124125 0.391806490 -6.9859081  0.0000
## 2|3      -1.341864182 0.380812480 -3.5236875  0.0004
## 3|4      -0.914692143 0.379716437 -2.4088821  0.0160
## 4|5       0.720744420 0.379174613  1.9008246  0.0573
```

```
(ci_une5 <- confint(une5, level=0.99375))
```

```
## Waiting for profiling to be done...
```

```
##                 0.3 %     99.7 %
## unemploy  0.053435579 0.69923829
## age_T3   -0.006296284 0.02510638
## sex_T3   -0.108869085 0.38630438
## bmi_T3   -0.014581684 0.01826884
```

```
exp(cbind(OR = coef(une5), ci_une5))
```

```
##                OR     0.3 %   99.7 %
## unemploy 1.448408 1.0548890 2.012219
## age_T3   1.009419 0.9937235 1.025424
## sex_T3   1.150639 0.8968478 1.471533
## bmi_T3   1.001746 0.9855241 1.018437
```

```
une6 <- polr(foodst_06_T3 ~ unemploy + age_T3 + sex_T3 + bmi_T3, data = subset, Hess=TRUE)
summary(une6)
```

```
## Call:
## polr(formula = foodst_06_T3 ~ unemploy + age_T3 + sex_T3 + bmi_T3, 
##     data = subset, Hess = TRUE)
## 
## Coefficients:
##             Value Std. Error t value
## unemploy -0.45705   0.109811  -4.162
## age_T3   -0.01994   0.005398  -3.693
## sex_T3   -0.39284   0.084601  -4.643
## bmi_T3   -0.01146   0.005709  -2.008
## 
## Intercepts:
##     Value   Std. Error t value
## 1|2 -2.6581  0.3585    -7.4142
## 2|3 -0.8868  0.3562    -2.4898
## 3|4 -0.4348  0.3566    -1.2193
## 4|5  1.2229  0.3658     3.3428
## 
## Residual Deviance: 9924.972 
## AIC: 9940.972
```

```
ctable_une6 <- coef(summary(une6))
p_une6 <- pnorm(abs(ctable_une6[, "t value"]), lower.tail = FALSE) * 2
(ctable_une6 <- cbind(ctable_une6, "p-value" =  round(p_une6, 4)))
```

```
##                Value  Std. Error   t value p-value
## unemploy -0.45704589 0.109810951 -4.162116  0.0000
## age_T3   -0.01993793 0.005398242 -3.693412  0.0002
## sex_T3   -0.39283735 0.084600734 -4.643427  0.0000
## bmi_T3   -0.01146270 0.005709027 -2.007819  0.0447
## 1|2      -2.65809571 0.358516367 -7.414154  0.0000
## 2|3      -0.88675270 0.356152916 -2.489809  0.0128
## 3|4      -0.43482467 0.356626041 -1.219273  0.2227
## 4|5       1.22287803 0.365827648  3.342771  0.0008
```

```
(ci_une6 <- confint(une6, level=0.99375))
```

```
## Waiting for profiling to be done...
```

```
##                0.3 %       99.7 %
## unemploy -0.76023313 -0.159026622
## age_T3   -0.03472753 -0.005200483
## sex_T3   -0.62403357 -0.161214004
## bmi_T3   -0.02713043  0.004104280
```

```
exp(cbind(OR = coef(une6), ci_une6))
```

```
##                 OR     0.3 %    99.7 %
## unemploy 0.6331513 0.4675574 0.8529737
## age_T3   0.9802595 0.9658686 0.9948130
## sex_T3   0.6751386 0.5357790 0.8511099
## bmi_T3   0.9886028 0.9732343 1.0041127
```

```
une7 <- polr(foodst_07_T3 ~ unemploy + age_T3 + sex_T3 + bmi_T3, data = subset, Hess=TRUE)
summary(une7)
```

```
## Call:
## polr(formula = foodst_07_T3 ~ unemploy + age_T3 + sex_T3 + bmi_T3, 
##     data = subset, Hess = TRUE)
## 
## Coefficients:
##             Value Std. Error t value
## unemploy -0.34625   0.119857  -2.889
## age_T3   -0.02143   0.005858  -3.658
## sex_T3   -0.34208   0.089675  -3.815
## bmi_T3    0.01285   0.006103   2.106
## 
## Intercepts:
##     Value   Std. Error t value
## 1|2 -1.0731  0.3824    -2.8061
## 2|3  0.5756  0.3828     1.5036
## 3|4  1.0913  0.3845     2.8384
## 4|5  2.8271  0.4050     6.9800
## 
## Residual Deviance: 7980.777 
## AIC: 7996.777
```

```
ctable_une7 <- coef(summary(une7))
p_une7 <- pnorm(abs(ctable_une7[, "t value"]), lower.tail = FALSE) * 2
(ctable_une7 <- cbind(ctable_une7, "p-value" =  round(p_une7, 4)))
```

```
##                Value  Std. Error   t value p-value
## unemploy -0.34625309 0.119856796 -2.888890  0.0039
## age_T3   -0.02142816 0.005857769 -3.658076  0.0003
## sex_T3   -0.34208108 0.089674605 -3.814693  0.0001
## bmi_T3    0.01285085 0.006103415  2.105518  0.0352
## 1|2      -1.07309390 0.382421369 -2.806051  0.0050
## 2|3       0.57563973 0.382840624  1.503601  0.1327
## 3|4       1.09132044 0.384485933  2.838388  0.0045
## 4|5       2.82708553 0.405023869  6.980047  0.0000
```

```
(ci_une7 <- confint(une7, level=0.99375))
```

```
## Waiting for profiling to be done...
```

```
##                 0.3 %       99.7 %
## unemploy -0.681005028 -0.024386737
## age_T3   -0.037503322 -0.005458346
## sex_T3   -0.586121610 -0.095414340
## bmi_T3   -0.003935069  0.029461553
```

```
exp(cbind(OR = coef(une7), ci_une7))
```

```
##                 OR     0.3 %    99.7 %
## unemploy 0.7073334 0.5061081 0.9759082
## age_T3   0.9787998 0.9631912 0.9945565
## sex_T3   0.7102906 0.5564814 0.9089962
## bmi_T3   1.0129338 0.9960727 1.0298998
```

```
une8 <- polr(foodst_08_T3 ~ unemploy + age_T3 + sex_T3 + bmi_T3, data = subset, Hess=TRUE)
summary(une8)
```

```
## Call:
## polr(formula = foodst_08_T3 ~ unemploy + age_T3 + sex_T3 + bmi_T3, 
##     data = subset, Hess = TRUE)
## 
## Coefficients:
##             Value Std. Error t value
## unemploy 0.003809   0.103379 0.03685
## age_T3   0.001348   0.005224 0.25798
## sex_T3   0.364149   0.082486 4.41466
## bmi_T3   0.012264   0.005426 2.26022
## 
## Intercepts:
##     Value   Std. Error t value
## 1|2 -0.8555  0.3446    -2.4822
## 2|3  0.3007  0.3435     0.8753
## 3|4  0.6349  0.3436     1.8476
## 4|5  2.3597  0.3460     6.8202
## 
## Residual Deviance: 11927.33 
## AIC: 11943.33
```

```
ctable_une8 <- coef(summary(une8))
p_une8 <- pnorm(abs(ctable_une8[, "t value"]), lower.tail = FALSE) * 2
(ctable_une8 <- cbind(ctable_une8, "p-value" =  round(p_une8, 4)))
```

```
##                 Value  Std. Error     t value p-value
## unemploy  0.003809375 0.103378987  0.03684864  0.9706
## age_T3    0.001347786 0.005224286  0.25798481  0.7964
## sex_T3    0.364149359 0.082486419  4.41465837  0.0000
## bmi_T3    0.012263651 0.005425878  2.26021502  0.0238
## 1|2      -0.855467384 0.344634014 -2.48224885  0.0131
## 2|3       0.300698362 0.343525904  0.87532951  0.3814
## 3|4       0.634926406 0.343648010  1.84760681  0.0647
## 4|5       2.359663792 0.345981121  6.82020968  0.0000
```

```
(ci_une8 <- confint(une8, level=0.99375))
```

```
## Waiting for profiling to be done...
```

```
##                 0.3 %     99.7 %
## unemploy -0.278670922 0.28697125
## age_T3   -0.012935471 0.01563979
## sex_T3    0.138517576 0.58975032
## bmi_T3   -0.002557929 0.02712369
```

```
exp(cbind(OR = coef(une8), ci_une8))
```

```
##                OR     0.3 %   99.7 %
## unemploy 1.003817 0.7567889 1.332386
## age_T3   1.001349 0.9871478 1.015763
## sex_T3   1.439289 1.1485699 1.803538
## bmi_T3   1.012339 0.9974453 1.027495
```

```
# path a
# Single parenthood -> Consumer attitudes

sin1 <- polr(foodst_01_T3 ~ singlepar + age_T3 + sex_T3 + bmi_T3, data = subset, Hess=TRUE)
summary(sin1)
```

```
## Call:
## polr(formula = foodst_01_T3 ~ singlepar + age_T3 + sex_T3 + bmi_T3, 
##     data = subset, Hess = TRUE)
## 
## Coefficients:
##              Value Std. Error t value
## singlepar -0.39417   0.093505 -4.2155
## age_T3     0.01124   0.005199  2.1614
## sex_T3     0.07625   0.082871  0.9201
## bmi_T3    -0.00959   0.005424 -1.7680
## 
## Intercepts:
##     Value   Std. Error t value
## 1|2 -2.1969  0.3455    -6.3587
## 2|3 -1.0927  0.3433    -3.1825
## 3|4 -0.5043  0.3428    -1.4712
## 4|5  1.2076  0.3434     3.5165
## 
## Residual Deviance: 12040.34 
## AIC: 12056.34 
## (16 observations deleted due to missingness)
```

```
ctable_sin1 <- coef(summary(sin1))
p_sin1 <- pnorm(abs(ctable_sin1[, "t value"]), lower.tail = FALSE) * 2
(ctable_sin1 <- cbind(ctable_sin1, "p-value" =  round(p_sin1, 4)))
```

```
##                  Value  Std. Error    t value p-value
## singlepar -0.394169402 0.093505058 -4.2154875  0.0000
## age_T3     0.011236582 0.005198780  2.1613883  0.0307
## sex_T3     0.076248675 0.082870825  0.9200907  0.3575
## bmi_T3    -0.009590377 0.005424487 -1.7679784  0.0771
## 1|2       -2.196939083 0.345500783 -6.3587094  0.0000
## 2|3       -1.092699509 0.343343017 -3.1825302  0.0015
## 3|4       -0.504331187 0.342805029 -1.4711896  0.1412
## 4|5        1.207644153 0.343417845  3.5165446  0.0004
```

```
(ci_sin1 <- confint(sin1, level=0.99375))
```

```
## Waiting for profiling to be done...
```

```
##                  0.3 %       99.7 %
## singlepar -0.649821305 -0.138238649
## age_T3    -0.002977701  0.025457117
## sex_T3    -0.150596412  0.302727212
## bmi_T3    -0.024415954  0.005258776
```

```
exp(cbind(OR = coef(sin1), ci_sin1))
```

```
##                  OR     0.3 %    99.7 %
## singlepar 0.6742398 0.5221391 0.8708908
## age_T3    1.0112999 0.9970267 1.0257839
## sex_T3    1.0792309 0.8601948 1.3535452
## bmi_T3    0.9904555 0.9758797 1.0052726
```

```
sin2 <- polr(foodst_02_T3 ~ singlepar + age_T3 + sex_T3 + bmi_T3, data = subset, Hess=TRUE)
summary(sin2)
```

```
## Call:
## polr(formula = foodst_02_T3 ~ singlepar + age_T3 + sex_T3 + bmi_T3, 
##     data = subset, Hess = TRUE)
## 
## Coefficients:
##               Value Std. Error  t value
## singlepar -0.064971   0.095748 -0.67857
## age_T3    -0.003718   0.005405 -0.68785
## sex_T3     0.001333   0.085836  0.01553
## bmi_T3     0.019435   0.005631  3.45164
## 
## Intercepts:
##     Value   Std. Error t value
## 1|2  0.1914  0.3519     0.5440
## 2|3  1.5241  0.3526     4.3227
## 3|4  2.2178  0.3538     6.2692
## 4|5  3.7974  0.3632    10.4561
## 
## Residual Deviance: 10241.05 
## AIC: 10257.05 
## (16 observations deleted due to missingness)
```

```
ctable_sin2 <- coef(summary(sin2))
p_sin2 <- pnorm(abs(ctable_sin2[, "t value"]), lower.tail = FALSE) * 2
(ctable_sin2 <- cbind(ctable_sin2, "p-value" =  round(p_sin2, 4)))
```

```
##                  Value  Std. Error    t value p-value
## singlepar -0.064971368 0.095748036 -0.6785661  0.4974
## age_T3    -0.003717549 0.005404611 -0.6878476  0.4915
## sex_T3     0.001332873 0.085835621  0.0155282  0.9876
## bmi_T3     0.019434733 0.005630574  3.4516432  0.0006
## 1|2        0.191409784 0.351858786  0.5439960  0.5864
## 2|3        1.524075695 0.352575175  4.3226971  0.0000
## 3|4        2.217803918 0.353761582  6.2692051  0.0000
## 4|5        3.797361277 0.363171961 10.4560971  0.0000
```

```
(ci_sin2 <- confint(sin2, level=0.99375))
```

```
## Waiting for profiling to be done...
```

```
##                  0.3 %     99.7 %
## singlepar -0.328676552 0.19535534
## age_T3    -0.018511705 0.01105096
## sex_T3    -0.232562105 0.23707942
## bmi_T3     0.004019616 0.03482512
```

```
exp(cbind(OR = coef(sin2), ci_sin2))
```

```
##                  OR     0.3 %   99.7 %
## singlepar 0.9370943 0.7198758 1.215743
## age_T3    0.9962894 0.9816586 1.011112
## sex_T3    1.0013338 0.7925005 1.267542
## bmi_T3    1.0196248 1.0040277 1.035439
```

```
sin3 <- polr(foodst_03_T3 ~ singlepar + age_T3 + sex_T3 + bmi_T3, data = subset, Hess=TRUE)
summary(sin3)
```

```
## Call:
## polr(formula = foodst_03_T3 ~ singlepar + age_T3 + sex_T3 + bmi_T3, 
##     data = subset, Hess = TRUE)
## 
## Coefficients:
##              Value Std. Error t value
## singlepar -0.41759   0.095437  -4.376
## age_T3     0.02240   0.005294   4.231
## sex_T3     0.33450   0.083814   3.991
## bmi_T3    -0.01503   0.005538  -2.714
## 
## Intercepts:
##     Value   Std. Error t value
## 1|2 -2.4574  0.3529    -6.9629
## 2|3 -1.1945  0.3473    -3.4397
## 3|4 -0.3848  0.3463    -1.1112
## 4|5  1.1377  0.3467     3.2813
## 
## Residual Deviance: 10755.99 
## AIC: 10771.99 
## (16 observations deleted due to missingness)
```

```
ctable_sin3 <- coef(summary(sin3))
p_sin3 <- pnorm(abs(ctable_sin3[, "t value"]), lower.tail = FALSE) * 2
(ctable_sin3 <- cbind(ctable_sin3, "p-value" =  round(p_sin3, 4)))
```

```
##                 Value  Std. Error   t value p-value
## singlepar -0.41759302 0.095436990 -4.375589  0.0000
## age_T3     0.02239857 0.005293530  4.231311  0.0000
## sex_T3     0.33449618 0.083813920  3.990938  0.0001
## bmi_T3    -0.01503307 0.005538485 -2.714293  0.0066
## 1|2       -2.45736059 0.352922417 -6.962892  0.0000
## 2|3       -1.19450267 0.347269379 -3.439701  0.0006
## 3|4       -0.38481534 0.346295120 -1.111235  0.2665
## 4|5        1.13767726 0.346717954  3.281276  0.0010
```

```
(ci_sin3 <- confint(sin3, level=0.99375))
```

```
## Waiting for profiling to be done...
```

```
##                  0.3 %       99.7 %
## singlepar -0.678274007 -0.156084567
## age_T3     0.007947825  0.036900720
## sex_T3     0.105029020  0.563541796
## bmi_T3    -0.030160521  0.000138258
```

```
exp(cbind(OR = coef(sin3), ci_sin3))
```

```
##                  OR     0.3 %    99.7 %
## singlepar 0.6586302 0.5074922 0.8554868
## age_T3    1.0226513 1.0079795 1.0375900
## sex_T3    1.3972363 1.1107428 1.7568840
## bmi_T3    0.9850794 0.9702898 1.0001383
```

```
sin4 <- polr(foodst_04_T3 ~ singlepar + age_T3 + sex_T3 + bmi_T3, data = subset, Hess=TRUE)
summary(sin4)
```

```
## Call:
## polr(formula = foodst_04_T3 ~ singlepar + age_T3 + sex_T3 + bmi_T3, 
##     data = subset, Hess = TRUE)
## 
## Coefficients:
##               Value Std. Error t value
## singlepar -0.487575   0.093018  -5.242
## age_T3     0.027951   0.005254   5.320
## sex_T3     0.089624   0.083212   1.077
## bmi_T3    -0.006229   0.005450  -1.143
## 
## Intercepts:
##     Value   Std. Error t value
## 1|2 -1.8240  0.3465    -5.2646
## 2|3 -0.5121  0.3437    -1.4899
## 3|4  0.1244  0.3435     0.3621
## 4|5  1.7252  0.3447     5.0055
## 
## Residual Deviance: 11905.29 
## AIC: 11921.29 
## (16 observations deleted due to missingness)
```

```
ctable_sin4 <- coef(summary(sin4))
p_sin4 <- pnorm(abs(ctable_sin4[, "t value"]), lower.tail = FALSE) * 2
(ctable_sin4 <- cbind(ctable_sin4, "p-value" =  round(p_sin4, 4)))
```

```
##                  Value  Std. Error    t value p-value
## singlepar -0.487575049 0.093017978 -5.2417292  0.0000
## age_T3     0.027951474 0.005254079  5.3199574  0.0000
## sex_T3     0.089623710 0.083211919  1.0770538  0.2815
## bmi_T3    -0.006229357 0.005450207 -1.1429578  0.2531
## 1|2       -1.823981072 0.346463488 -5.2645694  0.0000
## 2|3       -0.512131926 0.343734121 -1.4899072  0.1362
## 3|4        0.124356100 0.343467537  0.3620607  0.7173
## 4|5        1.725241672 0.344670537  5.0054806  0.0000
```

```
(ci_sin4 <- confint(sin4, level=0.99375))
```

```
## Waiting for profiling to be done...
```

```
##                 0.3 %       99.7 %
## singlepar -0.74195915 -0.233040158
## age_T3     0.01360091  0.042338659
## sex_T3    -0.13815079  0.317048101
## bmi_T3    -0.02112112  0.008694042
```

```
exp(cbind(OR = coef(sin4), ci_sin4))
```

```
##                  OR     0.3 %    99.7 %
## singlepar 0.6141138 0.4761801 0.7921218
## age_T3    1.0283458 1.0136938 1.0432477
## sex_T3    1.0937626 0.8709673 1.3730686
## bmi_T3    0.9937900 0.9791004 1.0087319
```

```
sin5 <- polr(foodst_05_T3 ~ singlepar + age_T3 + sex_T3 + bmi_T3, data = subset, Hess=TRUE)
summary(sin5)
```

```
## Call:
## polr(formula = foodst_05_T3 ~ singlepar + age_T3 + sex_T3 + bmi_T3, 
##     data = subset, Hess = TRUE)
## 
## Coefficients:
##               Value Std. Error t value
## singlepar -0.385001   0.100766 -3.8208
## age_T3     0.009361   0.005739  1.6312
## sex_T3     0.167732   0.090856  1.8461
## bmi_T3     0.002301   0.005986  0.3845
## 
## Intercepts:
##     Value   Std. Error t value
## 1|2 -3.4976  0.3874    -9.0292
## 2|3 -2.1040  0.3761    -5.5942
## 3|4 -1.6746  0.3749    -4.4672
## 4|5 -0.0381  0.3735    -0.1021
## 
## Residual Deviance: 8390.313 
## AIC: 8406.313 
## (16 observations deleted due to missingness)
```

```
ctable_sin5 <- coef(summary(sin5))
p_sin5 <- pnorm(abs(ctable_sin5[, "t value"]), lower.tail = FALSE) * 2
(ctable_sin5 <- cbind(ctable_sin5, "p-value" =  round(p_sin5, 4)))
```

```
##                  Value  Std. Error    t value p-value
## singlepar -0.385001348 0.100765722 -3.8207571  0.0001
## age_T3     0.009360884 0.005738814  1.6311530  0.1029
## sex_T3     0.167731988 0.090855770  1.8461347  0.0649
## bmi_T3     0.002301470 0.005986040  0.3844728  0.7006
## 1|2       -3.497614007 0.387368125 -9.0291735  0.0000
## 2|3       -2.103957371 0.376093669 -5.5942377  0.0000
## 3|4       -1.674613056 0.374872709 -4.4671512  0.0000
## 4|5       -0.038138289 0.373534001 -0.1021013  0.9187
```

```
(ci_sin5 <- confint(sin5, level=0.99375))
```

```
## Waiting for profiling to be done...
```

```
##                  0.3 %      99.7 %
## singlepar -0.659031720 -0.10748491
## age_T3    -0.006304964  0.02508797
## sex_T3    -0.082438006  0.41474830
## bmi_T3    -0.013978465  0.01877863
```

```
exp(cbind(OR = coef(sin5), ci_sin5))
```

```
##                  OR     0.3 %    99.7 %
## singlepar 0.6804497 0.5173520 0.8980901
## age_T3    1.0094048 0.9937149 1.0254053
## sex_T3    1.1826196 0.9208685 1.5139896
## bmi_T3    1.0023041 0.9861188 1.0189561
```

```
sin6 <- polr(foodst_06_T3 ~ singlepar + age_T3 + sex_T3 + bmi_T3, data = subset, Hess=TRUE)
summary(sin6)
```

```
## Call:
## polr(formula = foodst_06_T3 ~ singlepar + age_T3 + sex_T3 + bmi_T3, 
##     data = subset, Hess = TRUE)
## 
## Coefficients:
##              Value Std. Error t value
## singlepar  0.52023   0.095502   5.447
## age_T3    -0.02086   0.005399  -3.863
## sex_T3    -0.43134   0.085039  -5.072
## bmi_T3    -0.01228   0.005682  -2.162
## 
## Intercepts:
##     Value   Std. Error t value
## 1|2 -1.7199  0.3531    -4.8715
## 2|3  0.0571  0.3520     0.1623
## 3|4  0.5095  0.3527     1.4447
## 4|5  2.1732  0.3623     5.9983
## 
## Residual Deviance: 9865.621 
## AIC: 9881.621 
## (16 observations deleted due to missingness)
```

```
ctable_sin6 <- coef(summary(sin6))
p_sin6 <- pnorm(abs(ctable_sin6[, "t value"]), lower.tail = FALSE) * 2
(ctable_sin6 <- cbind(ctable_sin6, "p-value" =  round(p_sin6, 4)))
```

```
##                 Value  Std. Error    t value p-value
## singlepar  0.52023192 0.095501816  5.4473511  0.0000
## age_T3    -0.02085752 0.005398722 -3.8634178  0.0001
## sex_T3    -0.43133826 0.085039065 -5.0722366  0.0000
## bmi_T3    -0.01228475 0.005682431 -2.1618829  0.0306
## 1|2       -1.71989387 0.353052031 -4.8715025  0.0000
## 2|3        0.05713057 0.352046527  0.1622813  0.8711
## 3|4        0.50952709 0.352692704  1.4446771  0.1485
## 4|5        2.17324670 0.362310958  5.9982914  0.0000
```

```
(ci_sin6 <- confint(sin6, level=0.99375))
```

```
## Waiting for profiling to be done...
```

```
##                 0.3 %       99.7 %
## singlepar  0.25873381  0.781260087
## age_T3    -0.03564857 -0.006118936
## sex_T3    -0.66375936 -0.198542322
## bmi_T3    -0.02788655  0.003203038
```

```
exp(cbind(OR = coef(sin6), ci_sin6))
```

```
##                  OR     0.3 %    99.7 %
## singlepar 1.6824178 1.2952890 2.1842228
## age_T3    0.9793585 0.9649794 0.9938997
## sex_T3    0.6496391 0.5149120 0.8199251
## bmi_T3    0.9877904 0.9724987 1.0032082
```

```
sin7 <- polr(foodst_07_T3 ~ singlepar + age_T3 + sex_T3 + bmi_T3, data = subset, Hess=TRUE)
summary(sin7)
```

```
## Call:
## polr(formula = foodst_07_T3 ~ singlepar + age_T3 + sex_T3 + bmi_T3, 
##     data = subset, Hess = TRUE)
## 
## Coefficients:
##              Value Std. Error t value
## singlepar  0.29628   0.102333   2.895
## age_T3    -0.02195   0.005860  -3.746
## sex_T3    -0.36080   0.090171  -4.001
## bmi_T3     0.01207   0.006079   1.985
## 
## Intercepts:
##     Value   Std. Error t value
## 1|2 -0.4477  0.3783    -1.1836
## 2|3  1.2042  0.3794     3.1742
## 3|4  1.7244  0.3811     4.5249
## 4|5  3.4695  0.4023     8.6241
## 
## Residual Deviance: 7943.762 
## AIC: 7959.762 
## (16 observations deleted due to missingness)
```

```
ctable_sin7 <- coef(summary(sin7))
p_sin7 <- pnorm(abs(ctable_sin7[, "t value"]), lower.tail = FALSE) * 2
(ctable_sin7 <- cbind(ctable_sin7, "p-value" =  round(p_sin7, 4)))
```

```
##                 Value  Std. Error   t value p-value
## singlepar  0.29627829 0.102332699  2.895246  0.0038
## age_T3    -0.02195441 0.005860170 -3.746379  0.0002
## sex_T3    -0.36079642 0.090170850 -4.001253  0.0001
## bmi_T3     0.01206685 0.006078561  1.985149  0.0471
## 1|2       -0.44774830 0.378295974 -1.183593  0.2366
## 2|3        1.20415712 0.379353968  3.174231  0.0015
## 3|4        1.72443008 0.381094128  4.524945  0.0000
## 4|5        3.46949808 0.402303485  8.624082  0.0000
```

```
(ci_sin7 <- confint(sin7, level=0.99375))
```

```
## Waiting for profiling to be done...
```

```
##                  0.3 %       99.7 %
## singlepar  0.013771664  0.573961303
## age_T3    -0.038037202 -0.005979136
## sex_T3    -0.606221017 -0.112795550
## bmi_T3    -0.004652772  0.028608500
```

```
exp(cbind(OR = coef(sin7), ci_sin7))
```

```
##                  OR     0.3 %    99.7 %
## singlepar 1.3448444 1.0138669 1.7752856
## age_T3    0.9782848 0.9626771 0.9940387
## sex_T3    0.6971209 0.5454081 0.8933333
## bmi_T3    1.0121399 0.9953580 1.0290217
```

```
sin8 <- polr(foodst_08_T3 ~ singlepar + age_T3 + sex_T3 + bmi_T3, data = subset, Hess=TRUE)
summary(sin8)
```

```
## Call:
## polr(formula = foodst_08_T3 ~ singlepar + age_T3 + sex_T3 + bmi_T3, 
##     data = subset, Hess = TRUE)
## 
## Coefficients:
##               Value Std. Error t value
## singlepar 0.1352820   0.094666  1.4290
## age_T3    0.0009608   0.005228  0.1838
## sex_T3    0.3573892   0.082800  4.3163
## bmi_T3    0.0125159   0.005417  2.3103
## 
## Intercepts:
##     Value   Std. Error t value
## 1|2 -0.7285  0.3433    -2.1224
## 2|3  0.4258  0.3422     1.2440
## 3|4  0.7600  0.3424     2.2198
## 4|5  2.4793  0.3448     7.1906
## 
## Residual Deviance: 11886.19 
## AIC: 11902.19 
## (16 observations deleted due to missingness)
```

```
ctable_sin8 <- coef(summary(sin8))
p_sin8 <- pnorm(abs(ctable_sin8[, "t value"]), lower.tail = FALSE) * 2
(ctable_sin8 <- cbind(ctable_sin8, "p-value" =  round(p_sin8, 4)))
```

```
##                   Value  Std. Error    t value p-value
## singlepar  0.1352820435 0.094666217  1.4290425  0.1530
## age_T3     0.0009608112 0.005228326  0.1837703  0.8542
## sex_T3     0.3573892445 0.082799566  4.3163179  0.0000
## bmi_T3     0.0125159075 0.005417425  2.3103056  0.0209
## 1|2       -0.7285348470 0.343264343 -2.1223726  0.0338
## 2|3        0.4257571614 0.342249149  1.2439977  0.2135
## 3|4        0.7600221966 0.342375627  2.2198490  0.0264
## 4|5        2.4793138890 0.344798138  7.1906244  0.0000
```

```
(ci_sin8 <- confint(sin8, level=0.99375))
```

```
## Waiting for profiling to be done...
```

```
##                  0.3 %     99.7 %
## singlepar -0.123139280 0.39478447
## age_T3    -0.013333671 0.01526368
## sex_T3     0.130907975 0.58385299
## bmi_T3    -0.002282132 0.02735336
```

```
exp(cbind(OR = coef(sin8), ci_sin8))
```

```
##                 OR     0.3 %   99.7 %
## singlepar 1.144860 0.8841405 1.484064
## age_T3    1.000961 0.9867548 1.015381
## sex_T3    1.429592 1.1398629 1.792933
## bmi_T3    1.012595 0.9977205 1.027731
```

### 4.1.3 Path b: Consumer attitudes -> HDAS

```
subset$foodst_01_T3 <- as.numeric(subset$foodst_01_T3)
subset$foodst_02_T3 <- as.numeric(subset$foodst_02_T3)
subset$foodst_03_T3 <- as.numeric(subset$foodst_03_T3)
subset$foodst_04_T3 <- as.numeric(subset$foodst_04_T3)
subset$foodst_05_T3 <- as.numeric(subset$foodst_05_T3)
subset$foodst_06_T3 <- as.numeric(subset$foodst_06_T3)
subset$foodst_07_T3 <- as.numeric(subset$foodst_07_T3)
subset$foodst_08_T3 <- as.numeric(subset$foodst_08_T3)
```

```
# path b
# Consumer attitudes -> HDAS 

reg1 <- lm(hds_T3 ~ foodst_01_T3 + age_T3 + sex_T3 + bmi_T3, data = subset)
summary(reg1)
```

```
## 
## Call:
## lm(formula = hds_T3 ~ foodst_01_T3 + age_T3 + sex_T3 + bmi_T3, 
##     data = subset)
## 
## Residuals:
##      Min       1Q   Median       3Q      Max 
## -25.0222  -5.9920   0.2374   6.2447  22.7475 
## 
## Coefficients:
##              Estimate Std. Error t value Pr(>|t|)    
## (Intercept)  14.02958    1.60807   8.724  < 2e-16 ***
## foodst_01_T3  1.11221    0.10568  10.525  < 2e-16 ***
## age_T3        0.14288    0.02479   5.763 8.85e-09 ***
## sex_T3        1.34284    0.39488   3.401 0.000679 ***
## bmi_T3       -0.03071    0.02603  -1.180 0.238239    
## ---
## Signif. codes:  0 '***' 0.001 '**' 0.01 '*' 0.05 '.' 0.1 ' ' 1
## 
## Residual standard error: 8.631 on 4046 degrees of freedom
## Multiple R-squared:  0.03663,    Adjusted R-squared:  0.03568 
## F-statistic: 38.46 on 4 and 4046 DF,  p-value: < 2.2e-16
```

```
confint(reg1, level=0.99375)
```

```
##                  0.312 %    99.688 %
## (Intercept)   9.63021827 18.42894200
## foodst_01_T3  0.82310301  1.40132001
## age_T3        0.07505702  0.21070072
## sex_T3        0.26253466  2.42313972
## bmi_T3       -0.10193049  0.04051385
```

```
reg2 <- lm(hds_T3 ~ foodst_02_T3 + age_T3 + sex_T3 + bmi_T3, data = subset)
summary(reg2)
```

```
## 
## Call:
## lm(formula = hds_T3 ~ foodst_02_T3 + age_T3 + sex_T3 + bmi_T3, 
##     data = subset)
## 
## Residuals:
##     Min      1Q  Median      3Q     Max 
## -24.736  -5.992   0.287   6.322  24.872 
## 
## Coefficients:
##              Estimate Std. Error t value Pr(>|t|)    
## (Intercept)  18.96400    1.59565  11.885  < 2e-16 ***
## foodst_02_T3 -0.79453    0.12438  -6.388 1.87e-10 ***
## age_T3        0.14886    0.02499   5.956 2.81e-09 ***
## sex_T3        1.36898    0.39823   3.438 0.000593 ***
## bmi_T3       -0.02636    0.02630  -1.002 0.316231    
## ---
## Signif. codes:  0 '***' 0.001 '**' 0.01 '*' 0.05 '.' 0.1 ' ' 1
## 
## Residual standard error: 8.705 on 4046 degrees of freedom
## Multiple R-squared:  0.02014,    Adjusted R-squared:  0.01917 
## F-statistic: 20.79 on 4 and 4046 DF,  p-value: < 2.2e-16
```

```
confint(reg2, level=0.99375)
```

```
##                  0.312 %    99.688 %
## (Intercept)  14.59862696 23.32937778
## foodst_02_T3 -1.13481367 -0.45423708
## age_T3        0.08047762  0.21723797
## sex_T3        0.27949995  2.45845923
## bmi_T3       -0.09831033  0.04558807
```

```
reg3 <- lm(hds_T3 ~ foodst_03_T3 + age_T3 + sex_T3 + bmi_T3, data = subset)
summary(reg3)
```

```
## 
## Call:
## lm(formula = hds_T3 ~ foodst_03_T3 + age_T3 + sex_T3 + bmi_T3, 
##     data = subset)
## 
## Residuals:
##      Min       1Q   Median       3Q      Max 
## -26.4779  -5.9759   0.2085   6.2403  23.7434 
## 
## Coefficients:
##              Estimate Std. Error t value Pr(>|t|)    
## (Intercept)  14.55950    1.63073   8.928  < 2e-16 ***
## foodst_03_T3  0.94850    0.12254   7.740 1.25e-14 ***
## age_T3        0.13704    0.02499   5.485 4.39e-08 ***
## sex_T3        1.18704    0.39806   2.982  0.00288 ** 
## bmi_T3       -0.02781    0.02621  -1.061  0.28874    
## ---
## Signif. codes:  0 '***' 0.001 '**' 0.01 '*' 0.05 '.' 0.1 ' ' 1
## 
## Residual standard error: 8.685 on 4046 degrees of freedom
## Multiple R-squared:  0.0247, Adjusted R-squared:  0.02373 
## F-statistic: 25.61 on 4 and 4046 DF,  p-value: < 2.2e-16
```

```
confint(reg3, level=0.99375)
```

```
##                  0.312 %    99.688 %
## (Intercept)  10.09814199 19.02085379
## foodst_03_T3  0.61324730  1.28375533
## age_T3        0.06868901  0.20539969
## sex_T3        0.09803522  2.27604048
## bmi_T3       -0.09952690  0.04390104
```

```
reg4 <- lm(hds_T3 ~ foodst_04_T3 + age_T3 + sex_T3 + bmi_T3, data = subset)
summary(reg4)
```

```
## 
## Call:
## lm(formula = hds_T3 ~ foodst_04_T3 + age_T3 + sex_T3 + bmi_T3, 
##     data = subset)
## 
## Residuals:
##      Min       1Q   Median       3Q      Max 
## -25.2614  -6.0536   0.2757   6.3392  24.2052 
## 
## Coefficients:
##              Estimate Std. Error t value Pr(>|t|)    
## (Intercept)  15.05623    1.60696   9.369  < 2e-16 ***
## foodst_04_T3  0.93636    0.10949   8.552  < 2e-16 ***
## age_T3        0.13245    0.02497   5.304  1.2e-07 ***
## sex_T3        1.32438    0.39671   3.338  0.00085 ***
## bmi_T3       -0.03260    0.02615  -1.247  0.21259    
## ---
## Signif. codes:  0 '***' 0.001 '**' 0.01 '*' 0.05 '.' 0.1 ' ' 1
## 
## Residual standard error: 8.671 on 4046 degrees of freedom
## Multiple R-squared:  0.02783,    Adjusted R-squared:  0.02687 
## F-statistic: 28.96 on 4 and 4046 DF,  p-value: < 2.2e-16
```

```
confint(reg4, level=0.99375)
```

```
##                  0.312 %    99.688 %
## (Intercept)  10.65990003 19.45255490
## foodst_04_T3  0.63682143  1.23589051
## age_T3        0.06412922  0.20077100
## sex_T3        0.23906051  2.40969826
## bmi_T3       -0.10414239  0.03894093
```

```
reg5 <- lm(hds_T3 ~ foodst_05_T3 + age_T3 + sex_T3 + bmi_T3, data = subset)
summary(reg5)
```

```
## 
## Call:
## lm(formula = hds_T3 ~ foodst_05_T3 + age_T3 + sex_T3 + bmi_T3, 
##     data = subset)
## 
## Residuals:
##      Min       1Q   Median       3Q      Max 
## -24.6868  -6.0207   0.2928   6.3088  23.0017 
## 
## Coefficients:
##              Estimate Std. Error t value Pr(>|t|)    
## (Intercept)  15.74471    1.69398   9.295  < 2e-16 ***
## foodst_05_T3  0.47618    0.14320   3.325 0.000891 ***
## age_T3        0.14793    0.02509   5.896 4.02e-09 ***
## sex_T3        1.33642    0.39987   3.342 0.000839 ***
## bmi_T3       -0.03689    0.02635  -1.400 0.161482    
## ---
## Signif. codes:  0 '***' 0.001 '**' 0.01 '*' 0.05 '.' 0.1 ' ' 1
## 
## Residual standard error: 8.737 on 4046 degrees of freedom
## Multiple R-squared:  0.01295,    Adjusted R-squared:  0.01198 
## F-statistic: 13.27 on 4 and 4046 DF,  p-value: 9.562e-11
```

```
confint(reg5, level=0.99375)
```

```
##                  0.312 %   99.688 %
## (Intercept)  11.11032914 20.3790968
## foodst_05_T3  0.08440203  0.8679574
## age_T3        0.07929028  0.2165661
## sex_T3        0.24245880  2.4303868
## bmi_T3       -0.10896732  0.0351822
```

```
reg6 <- lm(hds_T3 ~ foodst_06_T3 + age_T3 + sex_T3 + bmi_T3, data = subset)
summary(reg6)
```

```
## 
## Call:
## lm(formula = hds_T3 ~ foodst_06_T3 + age_T3 + sex_T3 + bmi_T3, 
##     data = subset)
## 
## Residuals:
##      Min       1Q   Median       3Q      Max 
## -26.0263  -6.0410   0.2621   6.2407  22.9212 
## 
## Coefficients:
##              Estimate Std. Error t value Pr(>|t|)    
## (Intercept)  19.16731    1.63042  11.756  < 2e-16 ***
## foodst_06_T3 -0.51472    0.12913  -3.986 6.84e-05 ***
## age_T3        0.14444    0.02510   5.755 9.31e-09 ***
## sex_T3        1.26643    0.40041   3.163  0.00157 ** 
## bmi_T3       -0.03980    0.02634  -1.511  0.13089    
## ---
## Signif. codes:  0 '***' 0.001 '**' 0.01 '*' 0.05 '.' 0.1 ' ' 1
## 
## Residual standard error: 8.732 on 4046 degrees of freedom
## Multiple R-squared:  0.01413,    Adjusted R-squared:  0.01315 
## F-statistic: 14.49 on 4 and 4046 DF,  p-value: 9.364e-12
```

```
confint(reg6, level=0.99375)
```

```
##                  0.312 %    99.688 %
## (Intercept)  14.70679143 23.62782046
## foodst_06_T3 -0.86800304 -0.16142876
## age_T3        0.07577726  0.21310977
## sex_T3        0.17100099  2.36186100
## bmi_T3       -0.11186016  0.03226471
```

```
reg7 <- lm(hds_T3 ~ foodst_07_T3 + age_T3 + sex_T3 + bmi_T3, data = subset)
summary(reg7)
```

```
## 
## Call:
## lm(formula = hds_T3 ~ foodst_07_T3 + age_T3 + sex_T3 + bmi_T3, 
##     data = subset)
## 
## Residuals:
##     Min      1Q  Median      3Q     Max 
## -25.944  -6.089   0.358   6.244  23.551 
## 
## Coefficients:
##              Estimate Std. Error t value Pr(>|t|)    
## (Intercept)  19.21057    1.61634  11.885  < 2e-16 ***
## foodst_07_T3 -0.75476    0.15403  -4.900 9.96e-07 ***
## age_T3        0.14253    0.02508   5.683 1.42e-08 ***
## sex_T3        1.27769    0.39956   3.198   0.0014 ** 
## bmi_T3       -0.03186    0.02632  -1.210   0.2262    
## ---
## Signif. codes:  0 '***' 0.001 '**' 0.01 '*' 0.05 '.' 0.1 ' ' 1
## 
## Residual standard error: 8.723 on 4046 degrees of freedom
## Multiple R-squared:  0.01609,    Adjusted R-squared:  0.01512 
## F-statistic: 16.55 on 4 and 4046 DF,  p-value: 1.867e-13
```

```
confint(reg7, level=0.99375)
```

```
##                  0.312 %    99.688 %
## (Intercept)  14.78858979 23.63254853
## foodst_07_T3 -1.17616335 -0.33336066
## age_T3        0.07391277  0.21115513
## sex_T3        0.18456735  2.37080608
## bmi_T3       -0.10387097  0.04015171
```

```
reg8 <- lm(hds_T3 ~ foodst_08_T3 + age_T3 + sex_T3 + bmi_T3, data = subset)
summary(reg8)
```

```
## 
## Call:
## lm(formula = hds_T3 ~ foodst_08_T3 + age_T3 + sex_T3 + bmi_T3, 
##     data = subset)
## 
## Residuals:
##      Min       1Q   Median       3Q      Max 
## -26.2375  -5.9759   0.2956   6.3236  22.4188 
## 
## Coefficients:
##              Estimate Std. Error t value Pr(>|t|)    
## (Intercept)  16.51693    1.61022  10.258  < 2e-16 ***
## foodst_08_T3  0.44553    0.10188   4.373 1.26e-05 ***
## age_T3        0.14924    0.02506   5.955 2.82e-09 ***
## sex_T3        1.25626    0.40024   3.139  0.00171 ** 
## bmi_T3       -0.04057    0.02633  -1.541  0.12345    
## ---
## Signif. codes:  0 '***' 0.001 '**' 0.01 '*' 0.05 '.' 0.1 ' ' 1
## 
## Residual standard error: 8.728 on 4046 degrees of freedom
## Multiple R-squared:  0.01491,    Adjusted R-squared:  0.01394 
## F-statistic: 15.31 on 4 and 4046 DF,  p-value: 1.97e-12
```

```
confint(reg8, level=0.99375)
```

```
##                  0.312 %    99.688 %
## (Intercept)  12.11168830 20.92217876
## foodst_08_T3  0.16680153  0.72426191
## age_T3        0.08067631  0.21780061
## sex_T3        0.16128910  2.35123427
## bmi_T3       -0.11261723  0.03146905
```

### 5.1.4 Path c: Socioeconomic factors -> HDAS (Total effect)

```
# Education -> HDAS 
edu_total <- lm(hds_T3 ~ isced_cat2011_T3 + age_T3 + sex_T3 + bmi_T3, data = subset)
summary(edu_total)
```

```
## 
## Call:
## lm(formula = hds_T3 ~ isced_cat2011_T3 + age_T3 + sex_T3 + bmi_T3, 
##     data = subset)
## 
## Residuals:
##      Min       1Q   Median       3Q      Max 
## -26.2573  -5.9538   0.2701   6.1794  23.2303 
## 
## Coefficients:
##                  Estimate Std. Error t value Pr(>|t|)    
## (Intercept)       9.75044    1.74216   5.597 2.33e-08 ***
## isced_cat2011_T3  2.83593    0.24122  11.757  < 2e-16 ***
## age_T3            0.11714    0.02528   4.634 3.70e-06 ***
## sex_T3            1.71025    0.40340   4.240 2.29e-05 ***
## bmi_T3            0.02774    0.02678   1.036      0.3    
## ---
## Signif. codes:  0 '***' 0.001 '**' 0.01 '*' 0.05 '.' 0.1 ' ' 1
## 
## Residual standard error: 8.595 on 3926 degrees of freedom
##   (120 observations deleted due to missingness)
## Multiple R-squared:  0.04332,    Adjusted R-squared:  0.04234 
## F-statistic: 44.44 on 4 and 3926 DF,  p-value: < 2.2e-16
```

```
confint(edu_total, level=0.99375)
```

```
##                      0.312 %   99.688 %
## (Intercept)       4.98415372 14.5167271
## isced_cat2011_T3  2.17598537  3.4958782
## age_T3            0.04798404  0.1862944
## sex_T3            0.60660351  2.8139041
## bmi_T3           -0.04552748  0.1010094
```

```
# Income -> HDAS
inc_total <- lm(hds_T3 ~ income_cat_T3 + age_T3 + sex_T3 + bmi_T3, data = subset)
summary(inc_total)
```

```
## 
## Call:
## lm(formula = hds_T3 ~ income_cat_T3 + age_T3 + sex_T3 + bmi_T3, 
##     data = subset)
## 
## Residuals:
##      Min       1Q   Median       3Q      Max 
## -25.3508  -5.9875   0.2266   6.1946  24.3266 
## 
## Coefficients:
##                Estimate Std. Error t value Pr(>|t|)    
## (Intercept)   13.218987   1.674442   7.895 3.78e-15 ***
## income_cat_T3  0.998361   0.101950   9.793  < 2e-16 ***
## age_T3         0.141672   0.025556   5.544 3.17e-08 ***
## sex_T3         1.794955   0.404294   4.440 9.26e-06 ***
## bmi_T3         0.004667   0.027293   0.171    0.864    
## ---
## Signif. codes:  0 '***' 0.001 '**' 0.01 '*' 0.05 '.' 0.1 ' ' 1
## 
## Residual standard error: 8.642 on 3820 degrees of freedom
##   (226 observations deleted due to missingness)
## Multiple R-squared:  0.03526,    Adjusted R-squared:  0.03425 
## F-statistic: 34.91 on 4 and 3820 DF,  p-value: < 2.2e-16
```

```
confint(inc_total, level=0.99375)
```

```
##                   0.312 %    99.688 %
## (Intercept)    8.63790504 17.80006897
## income_cat_T3  0.71943808  1.27728480
## age_T3         0.07175274  0.21159123
## sex_T3         0.68885257  2.90105827
## bmi_T3        -0.07000288  0.07933741
```

```
# Migration background -> HDAS
mig_total <- lm(hds_T3 ~ migration + age_T3 + sex_T3 + bmi_T3, data = subset)
summary(mig_total)
```

```
## 
## Call:
## lm(formula = hds_T3 ~ migration + age_T3 + sex_T3 + bmi_T3, data = subset)
## 
## Residuals:
##      Min       1Q   Median       3Q      Max 
## -25.6288  -5.9917   0.2796   6.2904  22.9987 
## 
## Coefficients:
##             Estimate Std. Error t value Pr(>|t|)    
## (Intercept) 18.29861    1.65292  11.070  < 2e-16 ***
## migration   -0.55498    0.41340  -1.342  0.17952    
## age_T3       0.14946    0.02512   5.951  2.9e-09 ***
## sex_T3       1.36421    0.40025   3.408  0.00066 ***
## bmi_T3      -0.03541    0.02639  -1.342  0.17976    
## ---
## Signif. codes:  0 '***' 0.001 '**' 0.01 '*' 0.05 '.' 0.1 ' ' 1
## 
## Residual standard error: 8.747 on 4046 degrees of freedom
## Multiple R-squared:  0.0107, Adjusted R-squared:  0.009718 
## F-statistic: 10.94 on 4 and 4046 DF,  p-value: 8.076e-09
```

```
confint(mig_total, level=0.99375)
```

```
##                 0.312 %    99.688 %
## (Intercept) 13.77655692 22.82066973
## migration   -1.68595976  0.57599835
## age_T3       0.08074509  0.21816724
## sex_T3       0.26919942  2.45921517
## bmi_T3      -0.10761993  0.03679476
```

```
# Unemployment in Household -> HDAS
une_total <- lm(hds_T3 ~ unemploy + age_T3 + sex_T3 + bmi_T3, data = subset)
summary(une_total)
```

```
## 
## Call:
## lm(formula = hds_T3 ~ unemploy + age_T3 + sex_T3 + bmi_T3, data = subset)
## 
## Residuals:
##      Min       1Q   Median       3Q      Max 
## -25.6787  -5.9764   0.2377   6.2548  22.5006 
## 
## Coefficients:
##             Estimate Std. Error t value Pr(>|t|)    
## (Intercept) 19.58349    1.65523  11.831  < 2e-16 ***
## unemploy    -1.96346    0.48576  -4.042  5.4e-05 ***
## age_T3       0.14765    0.02507   5.889  4.2e-09 ***
## sex_T3       1.41920    0.39956   3.552 0.000387 ***
## bmi_T3      -0.02774    0.02642  -1.050 0.293901    
## ---
## Signif. codes:  0 '***' 0.001 '**' 0.01 '*' 0.05 '.' 0.1 ' ' 1
## 
## Residual standard error: 8.731 on 4046 degrees of freedom
## Multiple R-squared:  0.01424,    Adjusted R-squared:  0.01326 
## F-statistic: 14.61 on 4 and 4046 DF,  p-value: 7.537e-12
```

```
confint(une_total, level=0.99375)
```

```
##                 0.312 %    99.688 %
## (Intercept) 15.05510962 24.11187149
## unemploy    -3.29241270 -0.63451523
## age_T3       0.07905807  0.21624483
## sex_T3       0.32606907  2.51232562
## bmi_T3      -0.10002093  0.04454884
```

```
# Single parenthood -> HDAS
sin_total <- lm(hds_T3 ~ singlepar + age_T3 + sex_T3 + bmi_T3, data = subset)
summary(sin_total)
```

```
## 
## Call:
## lm(formula = hds_T3 ~ singlepar + age_T3 + sex_T3 + bmi_T3, data = subset)
## 
## Residuals:
##      Min       1Q   Median       3Q      Max 
## -25.5548  -6.0104   0.2556   6.3154  22.6570 
## 
## Coefficients:
##             Estimate Std. Error t value Pr(>|t|)    
## (Intercept) 17.89985    1.65218  10.834  < 2e-16 ***
## singlepar   -0.19402    0.45571  -0.426 0.670303    
## age_T3       0.14869    0.02512   5.919 3.51e-09 ***
## sex_T3       1.38268    0.40136   3.445 0.000577 ***
## bmi_T3      -0.03619    0.02640  -1.371 0.170541    
## ---
## Signif. codes:  0 '***' 0.001 '**' 0.01 '*' 0.05 '.' 0.1 ' ' 1
## 
## Residual standard error: 8.739 on 4030 degrees of freedom
##   (16 observations deleted due to missingness)
## Multiple R-squared:  0.01022,    Adjusted R-squared:  0.009238 
## F-statistic:  10.4 on 4 and 4030 DF,  p-value: 2.212e-08
```

```
confint(sin_total, level=0.99375)
```

```
##                 0.312 %    99.688 %
## (Intercept) 13.37979980 22.41990204
## singlepar   -1.44075322  1.05270542
## age_T3       0.07996228  0.21741905
## sex_T3       0.28463933  2.48072971
## bmi_T3      -0.10841117  0.03603819
```

```
unloadNamespace("MASS")
```

## 4.2 Structural equation modelling using lavaan package (indirect and direct effect)

### 4.2.1 Highest level of education in household

Controlled for age, sex, BMI

```
SEM_model_edu1 <- '
    level: 1
    hds_T3 ~ isced_cat2011_T3 + a1*foodst_01_T3 + a2*foodst_02_T3 + a3*foodst_03_T3 + a4*foodst_04_T3 + a5*foodst_05_T3 + a6*foodst_06_T3 + a7*foodst_07_T3 + a8*foodst_08_T3 + age_T3 + sex_T3 + bmi_T3
    foodst_01_T3 ~ b1*isced_cat2011_T3
    foodst_02_T3 ~ b2*isced_cat2011_T3
    foodst_03_T3 ~ b3*isced_cat2011_T3
    foodst_04_T3 ~ b4*isced_cat2011_T3
    foodst_05_T3 ~ b5*isced_cat2011_T3
    foodst_06_T3 ~ b6*isced_cat2011_T3
    foodst_07_T3 ~ b7*isced_cat2011_T3
    foodst_08_T3 ~ b8*isced_cat2011_T3
    foodst_01_T3 ~~ foodst_02_T3 + foodst_03_T3 + foodst_04_T3 + foodst_05_T3 + foodst_06_T3 + foodst_07_T3 + foodst_08_T3
    foodst_02_T3 ~~ foodst_03_T3 + foodst_04_T3 + foodst_05_T3 + foodst_06_T3 + foodst_07_T3 + foodst_08_T3
    foodst_03_T3 ~~ foodst_04_T3 + foodst_05_T3 + foodst_06_T3 + foodst_07_T3 + foodst_08_T3
    foodst_04_T3 ~~ foodst_05_T3 + foodst_06_T3 + foodst_07_T3 + foodst_08_T3
    foodst_05_T3 ~~ foodst_06_T3 + foodst_07_T3 + foodst_08_T3
    foodst_06_T3 ~~ foodst_07_T3 + foodst_08_T3 
    foodst_07_T3 ~~ foodst_08_T3
    ebindfoodst_01_T3 := a1*b1 
    ebindfoodst_02_T3 := a2*b2
    ebindfoodst_03_T3 := a3*b3
    ebindfoodst_04_T3 := a4*b4
    ebindfoodst_05_T3 := a5*b5
    ebindfoodst_06_T3 := a6*b6
    ebindfoodst_07_T3 := a7*b7
    ebindfoodst_08_T3 := a8*b8

    level: 2
    hds_T3 ~ 1

'

fit_SEM_edu1 <- sem(model = SEM_model_edu1, data = subset, cluster = "country")

summary(fit_SEM_edu1)
```

```
## lavaan 0.6.16 ended normally after 126 iterations
## 
##   Estimator                                         ML
##   Optimization method                           NLMINB
##   Number of model parameters                        67
## 
##                                                   Used       Total
##   Number of observations                          3931        4051
##   Number of clusters [country]                       8            
## 
## Model Test User Model:
##                                                       
##   Test statistic                                55.391
##   Degrees of freedom                                24
##   P-value (Chi-square)                           0.000
## 
## Parameter Estimates:
## 
##   Standard errors                             Standard
##   Information                                 Observed
##   Observed information based on                Hessian
## 
## 
## Level 1 [within]:
## 
## Regressions:
##                    Estimate  Std.Err  z-value  P(>|z|)
##   hds_T3 ~                                            
##     i_2011_T3         1.547    0.232    6.678    0.000
##     fds_01_T3 (a1)    0.775    0.113    6.856    0.000
##     fds_02_T3 (a2)   -0.561    0.121   -4.627    0.000
##     fds_03_T3 (a3)    0.305    0.146    2.084    0.037
##     fds_04_T3 (a4)    0.600    0.131    4.563    0.000
##     fds_05_T3 (a5)   -0.103    0.152   -0.679    0.497
##     fds_06_T3 (a6)   -0.640    0.138   -4.651    0.000
##     fds_07_T3 (a7)   -0.018    0.162   -0.113    0.910
##     fds_08_T3 (a8)    0.341    0.098    3.495    0.000
##     age_T3            0.092    0.024    3.846    0.000
##     sex_T3            1.641    0.383    4.290    0.000
##     bmi_T3            0.075    0.025    2.963    0.003
##   foodst_01_T3 ~                                      
##     i_2011_T3 (b1)    0.079    0.035    2.253    0.024
##   foodst_02_T3 ~                                      
##     i_2011_T3 (b2)   -0.258    0.030   -8.739    0.000
##   foodst_03_T3 ~                                      
##     i_2011_T3 (b3)    0.089    0.030    2.916    0.004
##   foodst_04_T3 ~                                      
##     i_2011_T3 (b4)    0.081    0.034    2.395    0.017
##   foodst_05_T3 ~                                      
##     i_2011_T3 (b5)   -0.025    0.026   -0.939    0.348
##   foodst_06_T3 ~                                      
##     i_2011_T3 (b6)    0.190    0.029    6.570    0.000
##   foodst_07_T3 ~                                      
##     i_2011_T3 (b7)   -0.002    0.024   -0.064    0.949
##   foodst_08_T3 ~                                      
##     i_2011_T3 (b8)   -0.027    0.037   -0.739    0.460
## 
## Covariances:
##                    Estimate  Std.Err  z-value  P(>|z|)
##  .foodst_01_T3 ~~                                     
##    .foodst_02_T3      0.183    0.022    8.217    0.000
##    .foodst_03_T3      0.579    0.025   23.532    0.000
##    .foodst_04_T3      0.565    0.027   20.918    0.000
##    .foodst_05_T3      0.196    0.020    9.854    0.000
##    .foodst_06_T3     -0.108    0.022   -4.959    0.000
##    .foodst_07_T3     -0.092    0.018   -5.037    0.000
##    .foodst_08_T3      0.159    0.028    5.741    0.000
##  .foodst_02_T3 ~~                                     
##    .foodst_03_T3      0.067    0.019    3.487    0.000
##    .foodst_04_T3      0.106    0.022    4.919    0.000
##    .foodst_05_T3      0.044    0.017    2.617    0.009
##    .foodst_06_T3      0.070    0.018    3.811    0.000
##    .foodst_07_T3      0.062    0.015    4.029    0.000
##    .foodst_08_T3     -0.037    0.023   -1.597    0.110
##  .foodst_03_T3 ~~                                     
##    .foodst_04_T3      0.753    0.025   29.849    0.000
##    .foodst_05_T3      0.361    0.018   19.979    0.000
##    .foodst_06_T3     -0.239    0.019  -12.420    0.000
##    .foodst_07_T3     -0.191    0.016  -11.819    0.000
##    .foodst_08_T3      0.175    0.024    7.244    0.000
##  .foodst_04_T3 ~~                                     
##    .foodst_05_T3      0.490    0.021   23.720    0.000
##    .foodst_06_T3     -0.238    0.021  -11.137    0.000
##    .foodst_07_T3     -0.193    0.018  -10.732    0.000
##    .foodst_08_T3      0.209    0.027    7.737    0.000
##  .foodst_05_T3 ~~                                     
##    .foodst_06_T3     -0.231    0.017  -13.877    0.000
##    .foodst_07_T3     -0.174    0.014  -12.449    0.000
##    .foodst_08_T3      0.164    0.021    7.877    0.000
##  .foodst_06_T3 ~~                                     
##    .foodst_07_T3      0.400    0.016   24.472    0.000
##    .foodst_08_T3     -0.055    0.023   -2.414    0.016
##  .foodst_07_T3 ~~                                     
##    .foodst_08_T3     -0.033    0.019   -1.735    0.083
## 
## Intercepts:
##                    Estimate  Std.Err  z-value  P(>|z|)
##    .hds_T3            0.000                           
##    .foodst_01_T3      3.251    0.089   36.549    0.000
##    .foodst_02_T3      2.538    0.075   33.732    0.000
##    .foodst_03_T3      3.745    0.078   48.317    0.000
##    .foodst_04_T3      3.343    0.087   38.621    0.000
##    .foodst_05_T3      4.427    0.067   66.221    0.000
##    .foodst_06_T3      1.452    0.073   19.756    0.000
##    .foodst_07_T3      1.570    0.062   25.353    0.000
##    .foodst_08_T3      3.424    0.094   36.574    0.000
## 
## Variances:
##                    Estimate  Std.Err  z-value  P(>|z|)
##    .hds_T3           65.685    1.483   44.289    0.000
##    .foodst_01_T3      1.641    0.037   44.334    0.000
##    .foodst_02_T3      1.174    0.026   44.334    0.000
##    .foodst_03_T3      1.246    0.028   44.334    0.000
##    .foodst_04_T3      1.554    0.035   44.334    0.000
##    .foodst_05_T3      0.927    0.021   44.334    0.000
##    .foodst_06_T3      1.120    0.025   44.334    0.000
##    .foodst_07_T3      0.796    0.018   44.334    0.000
##    .foodst_08_T3      1.818    0.041   44.334    0.000
## 
## 
## Level 2 [country]:
## 
## Intercepts:
##                    Estimate  Std.Err  z-value  P(>|z|)
##    .hds_T3            9.042    2.046    4.419    0.000
## 
## Variances:
##                    Estimate  Std.Err  z-value  P(>|z|)
##    .hds_T3            6.064    3.111    1.949    0.051
## 
## Defined Parameters:
##                    Estimate  Std.Err  z-value  P(>|z|)
##     ebndfdst_01_T3    0.061    0.029    2.141    0.032
##     ebndfdst_02_T3    0.145    0.035    4.089    0.000
##     ebndfdst_03_T3    0.027    0.016    1.695    0.090
##     ebndfdst_04_T3    0.049    0.023    2.121    0.034
##     ebndfdst_05_T3    0.003    0.005    0.550    0.582
##     ebndfdst_06_T3   -0.122    0.032   -3.796    0.000
##     ebndfdst_07_T3    0.000    0.001    0.056    0.956
##     ebndfdst_08_T3   -0.009    0.013   -0.723    0.470
```

```
parameterEstimates(fit_SEM_edu1, ci=TRUE, level=.99375)
```

```
##                  lhs op              rhs block level             label    est
## 1             hds_T3  ~ isced_cat2011_T3     1     1                    1.547
## 2             hds_T3  ~     foodst_01_T3     1     1                a1  0.775
## 3             hds_T3  ~     foodst_02_T3     1     1                a2 -0.561
## 4             hds_T3  ~     foodst_03_T3     1     1                a3  0.305
## 5             hds_T3  ~     foodst_04_T3     1     1                a4  0.600
## 6             hds_T3  ~     foodst_05_T3     1     1                a5 -0.103
## 7             hds_T3  ~     foodst_06_T3     1     1                a6 -0.640
## 8             hds_T3  ~     foodst_07_T3     1     1                a7 -0.018
## 9             hds_T3  ~     foodst_08_T3     1     1                a8  0.341
## 10            hds_T3  ~           age_T3     1     1                    0.092
## 11            hds_T3  ~           sex_T3     1     1                    1.641
## 12            hds_T3  ~           bmi_T3     1     1                    0.075
## 13      foodst_01_T3  ~ isced_cat2011_T3     1     1                b1  0.079
## 14      foodst_02_T3  ~ isced_cat2011_T3     1     1                b2 -0.258
## 15      foodst_03_T3  ~ isced_cat2011_T3     1     1                b3  0.089
## 16      foodst_04_T3  ~ isced_cat2011_T3     1     1                b4  0.081
## 17      foodst_05_T3  ~ isced_cat2011_T3     1     1                b5 -0.025
## 18      foodst_06_T3  ~ isced_cat2011_T3     1     1                b6  0.190
## 19      foodst_07_T3  ~ isced_cat2011_T3     1     1                b7 -0.002
## 20      foodst_08_T3  ~ isced_cat2011_T3     1     1                b8 -0.027
## 21      foodst_01_T3 ~~     foodst_02_T3     1     1                    0.183
## 22      foodst_01_T3 ~~     foodst_03_T3     1     1                    0.579
## 23      foodst_01_T3 ~~     foodst_04_T3     1     1                    0.565
## 24      foodst_01_T3 ~~     foodst_05_T3     1     1                    0.196
## 25      foodst_01_T3 ~~     foodst_06_T3     1     1                   -0.108
## 26      foodst_01_T3 ~~     foodst_07_T3     1     1                   -0.092
## 27      foodst_01_T3 ~~     foodst_08_T3     1     1                    0.159
## 28      foodst_02_T3 ~~     foodst_03_T3     1     1                    0.067
## 29      foodst_02_T3 ~~     foodst_04_T3     1     1                    0.106
## 30      foodst_02_T3 ~~     foodst_05_T3     1     1                    0.044
## 31      foodst_02_T3 ~~     foodst_06_T3     1     1                    0.070
## 32      foodst_02_T3 ~~     foodst_07_T3     1     1                    0.062
## 33      foodst_02_T3 ~~     foodst_08_T3     1     1                   -0.037
## 34      foodst_03_T3 ~~     foodst_04_T3     1     1                    0.753
## 35      foodst_03_T3 ~~     foodst_05_T3     1     1                    0.361
## 36      foodst_03_T3 ~~     foodst_06_T3     1     1                   -0.239
## 37      foodst_03_T3 ~~     foodst_07_T3     1     1                   -0.191
## 38      foodst_03_T3 ~~     foodst_08_T3     1     1                    0.175
## 39      foodst_04_T3 ~~     foodst_05_T3     1     1                    0.490
## 40      foodst_04_T3 ~~     foodst_06_T3     1     1                   -0.238
## 41      foodst_04_T3 ~~     foodst_07_T3     1     1                   -0.193
## 42      foodst_04_T3 ~~     foodst_08_T3     1     1                    0.209
## 43      foodst_05_T3 ~~     foodst_06_T3     1     1                   -0.231
## 44      foodst_05_T3 ~~     foodst_07_T3     1     1                   -0.174
## 45      foodst_05_T3 ~~     foodst_08_T3     1     1                    0.164
## 46      foodst_06_T3 ~~     foodst_07_T3     1     1                    0.400
## 47      foodst_06_T3 ~~     foodst_08_T3     1     1                   -0.055
## 48      foodst_07_T3 ~~     foodst_08_T3     1     1                   -0.033
## 49            hds_T3 ~~           hds_T3     1     1                   65.685
## 50      foodst_01_T3 ~~     foodst_01_T3     1     1                    1.641
## 51      foodst_02_T3 ~~     foodst_02_T3     1     1                    1.174
## 52      foodst_03_T3 ~~     foodst_03_T3     1     1                    1.246
## 53      foodst_04_T3 ~~     foodst_04_T3     1     1                    1.554
## 54      foodst_05_T3 ~~     foodst_05_T3     1     1                    0.927
## 55      foodst_06_T3 ~~     foodst_06_T3     1     1                    1.120
## 56      foodst_07_T3 ~~     foodst_07_T3     1     1                    0.796
## 57      foodst_08_T3 ~~     foodst_08_T3     1     1                    1.818
## 58  isced_cat2011_T3 ~~ isced_cat2011_T3     1     1                    0.342
## 59  isced_cat2011_T3 ~~           age_T3     1     1                    0.306
## 60  isced_cat2011_T3 ~~           sex_T3     1     1                   -0.015
## 61  isced_cat2011_T3 ~~           bmi_T3     1     1                   -0.590
## 62            age_T3 ~~           age_T3     1     1                   31.331
## 63            age_T3 ~~           sex_T3     1     1                   -0.444
## 64            age_T3 ~~           bmi_T3     1     1                    2.395
## 65            sex_T3 ~~           sex_T3     1     1                    0.124
## 66            sex_T3 ~~           bmi_T3     1     1                   -0.249
## 67            bmi_T3 ~~           bmi_T3     1     1                   27.964
## 68            hds_T3 ~1                      1     1                    0.000
## 69      foodst_01_T3 ~1                      1     1                    3.251
## 70      foodst_02_T3 ~1                      1     1                    2.538
## 71      foodst_03_T3 ~1                      1     1                    3.745
## 72      foodst_04_T3 ~1                      1     1                    3.343
## 73      foodst_05_T3 ~1                      1     1                    4.427
## 74      foodst_06_T3 ~1                      1     1                    1.452
## 75      foodst_07_T3 ~1                      1     1                    1.570
## 76      foodst_08_T3 ~1                      1     1                    3.424
## 77  isced_cat2011_T3 ~1                      1     1                    2.476
## 78            age_T3 ~1                      1     1                   41.900
## 79            sex_T3 ~1                      1     1                    1.855
## 80            bmi_T3 ~1                      1     1                   26.051
## 81            hds_T3 ~1                      2     2                    9.042
## 82            hds_T3 ~~           hds_T3     2     2                    6.064
## 83 ebindfoodst_01_T3 :=            a1*b1     0     0 ebindfoodst_01_T3  0.061
## 84 ebindfoodst_02_T3 :=            a2*b2     0     0 ebindfoodst_02_T3  0.145
## 85 ebindfoodst_03_T3 :=            a3*b3     0     0 ebindfoodst_03_T3  0.027
## 86 ebindfoodst_04_T3 :=            a4*b4     0     0 ebindfoodst_04_T3  0.049
## 87 ebindfoodst_05_T3 :=            a5*b5     0     0 ebindfoodst_05_T3  0.003
## 88 ebindfoodst_06_T3 :=            a6*b6     0     0 ebindfoodst_06_T3 -0.122
## 89 ebindfoodst_07_T3 :=            a7*b7     0     0 ebindfoodst_07_T3  0.000
## 90 ebindfoodst_08_T3 :=            a8*b8     0     0 ebindfoodst_08_T3 -0.009
##       se       z pvalue ci.lower ci.upper
## 1  0.232   6.678  0.000    0.913    2.180
## 2  0.113   6.856  0.000    0.466    1.084
## 3  0.121  -4.627  0.000   -0.893   -0.229
## 4  0.146   2.084  0.037   -0.095    0.704
## 5  0.131   4.563  0.000    0.240    0.959
## 6  0.152  -0.679  0.497   -0.518    0.312
## 7  0.138  -4.651  0.000   -1.017   -0.264
## 8  0.162  -0.113  0.910   -0.463    0.426
## 9  0.098   3.495  0.000    0.074    0.607
## 10 0.024   3.846  0.000    0.027    0.158
## 11 0.383   4.290  0.000    0.595    2.687
## 12 0.025   2.963  0.003    0.006    0.144
## 13 0.035   2.253  0.024   -0.017    0.174
## 14 0.030  -8.739  0.000   -0.339   -0.178
## 15 0.030   2.916  0.004    0.006    0.172
## 16 0.034   2.395  0.017   -0.012    0.175
## 17 0.026  -0.939  0.348   -0.097    0.047
## 18 0.029   6.570  0.000    0.111    0.269
## 19 0.024  -0.064  0.949   -0.068    0.065
## 20 0.037  -0.739  0.460   -0.128    0.073
## 21 0.022   8.217  0.000    0.122    0.244
## 22 0.025  23.532  0.000    0.512    0.646
## 23 0.027  20.918  0.000    0.491    0.639
## 24 0.020   9.854  0.000    0.142    0.251
## 25 0.022  -4.959  0.000   -0.167   -0.048
## 26 0.018  -5.037  0.000   -0.142   -0.042
## 27 0.028   5.741  0.000    0.083    0.234
## 28 0.019   3.487  0.000    0.015    0.120
## 29 0.022   4.919  0.000    0.047    0.165
## 30 0.017   2.617  0.009   -0.002    0.089
## 31 0.018   3.811  0.000    0.020    0.120
## 32 0.015   4.029  0.000    0.020    0.104
## 33 0.023  -1.597  0.110   -0.101    0.027
## 34 0.025  29.849  0.000    0.684    0.822
## 35 0.018  19.979  0.000    0.312    0.411
## 36 0.019 -12.420  0.000   -0.291   -0.186
## 37 0.016 -11.819  0.000   -0.235   -0.147
## 38 0.024   7.244  0.000    0.109    0.241
## 39 0.021  23.720  0.000    0.434    0.547
## 40 0.021 -11.137  0.000   -0.297   -0.180
## 41 0.018 -10.732  0.000   -0.242   -0.144
## 42 0.027   7.737  0.000    0.135    0.283
## 43 0.017 -13.877  0.000   -0.277   -0.186
## 44 0.014 -12.449  0.000   -0.212   -0.136
## 45 0.021   7.877  0.000    0.107    0.221
## 46 0.016  24.472  0.000    0.355    0.445
## 47 0.023  -2.414  0.016   -0.117    0.007
## 48 0.019  -1.735  0.083   -0.086    0.019
## 49 1.483  44.289  0.000   61.629   69.740
## 50 0.037  44.334  0.000    1.539    1.742
## 51 0.026  44.334  0.000    1.101    1.246
## 52 0.028  44.334  0.000    1.169    1.323
## 53 0.035  44.334  0.000    1.458    1.650
## 54 0.021  44.334  0.000    0.870    0.984
## 55 0.025  44.334  0.000    1.051    1.189
## 56 0.018  44.334  0.000    0.746    0.845
## 57 0.041  44.334  0.000    1.706    1.930
## 58 0.000      NA     NA    0.342    0.342
## 59 0.000      NA     NA    0.306    0.306
## 60 0.000      NA     NA   -0.015   -0.015
## 61 0.000      NA     NA   -0.590   -0.590
## 62 0.000      NA     NA   31.331   31.331
## 63 0.000      NA     NA   -0.444   -0.444
## 64 0.000      NA     NA    2.395    2.395
## 65 0.000      NA     NA    0.124    0.124
## 66 0.000      NA     NA   -0.249   -0.249
## 67 0.000      NA     NA   27.964   27.964
## 68 0.000      NA     NA    0.000    0.000
## 69 0.089  36.549  0.000    3.008    3.494
## 70 0.075  33.732  0.000    2.332    2.744
## 71 0.078  48.317  0.000    3.533    3.957
## 72 0.087  38.621  0.000    3.106    3.580
## 73 0.067  66.221  0.000    4.245    4.610
## 74 0.073  19.756  0.000    1.251    1.653
## 75 0.062  25.353  0.000    1.401    1.740
## 76 0.094  36.574  0.000    3.168    3.680
## 77 0.000      NA     NA    2.476    2.476
## 78 0.000      NA     NA   41.900   41.900
## 79 0.000      NA     NA    1.855    1.855
## 80 0.000      NA     NA   26.051   26.051
## 81 2.046   4.419  0.000    3.447   14.637
## 82 3.111   1.949  0.051   -2.443   14.570
## 83 0.029   2.141  0.032   -0.017    0.139
## 84 0.035   4.089  0.000    0.048    0.242
## 85 0.016   1.695  0.090   -0.017    0.071
## 86 0.023   2.121  0.034   -0.014    0.112
## 87 0.005   0.550  0.582   -0.010    0.015
## 88 0.032  -3.796  0.000   -0.209   -0.034
## 89 0.001   0.056  0.956   -0.001    0.001
## 90 0.013  -0.723  0.470   -0.044    0.026
```

### 4.2.2 Household income

Controlled for age, sex, BMI

```
SEM_model_inc1 <- '
    level: 1
    hds_T3 ~ income_cat_T3 + a1*foodst_01_T3 + a2*foodst_02_T3 + a3*foodst_03_T3 + a4*foodst_04_T3 + a5*foodst_05_T3 + a6*foodst_06_T3 + a7*foodst_07_T3 + a8*foodst_08_T3 + age_T3 + sex_T3 + bmi_T3
    foodst_01_T3 ~ b1*income_cat_T3
    foodst_02_T3 ~ b2*income_cat_T3
    foodst_03_T3 ~ b3*income_cat_T3
    foodst_04_T3 ~ b4*income_cat_T3
    foodst_05_T3 ~ b5*income_cat_T3
    foodst_06_T3 ~ b6*income_cat_T3
    foodst_07_T3 ~ b7*income_cat_T3
    foodst_08_T3 ~ b8*income_cat_T3
    foodst_01_T3 ~~ foodst_02_T3 + foodst_03_T3 + foodst_04_T3 + foodst_05_T3 + foodst_06_T3 + foodst_07_T3 + foodst_08_T3
    foodst_02_T3 ~~ foodst_03_T3 + foodst_04_T3 + foodst_05_T3 + foodst_06_T3 + foodst_07_T3 + foodst_08_T3
    foodst_03_T3 ~~ foodst_04_T3 + foodst_05_T3 + foodst_06_T3 + foodst_07_T3 + foodst_08_T3
    foodst_04_T3 ~~ foodst_05_T3 + foodst_06_T3 + foodst_07_T3 + foodst_08_T3
    foodst_05_T3 ~~ foodst_06_T3 + foodst_07_T3 + foodst_08_T3
    foodst_06_T3 ~~ foodst_07_T3 + foodst_08_T3 
    foodst_07_T3 ~~ foodst_08_T3
    ebindfoodst_01_T3 := a1*b1 
    ebindfoodst_02_T3 := a2*b2
    ebindfoodst_03_T3 := a3*b3
    ebindfoodst_04_T3 := a4*b4
    ebindfoodst_05_T3 := a5*b5
    ebindfoodst_06_T3 := a6*b6
    ebindfoodst_07_T3 := a7*b7
    ebindfoodst_08_T3 := a8*b8

    level: 2
    hds_T3 ~ 1

'

fit_SEM_inc1 <- sem(model = SEM_model_inc1, data = subset, cluster = "country")

summary(fit_SEM_inc1)
```

```
## lavaan 0.6.16 ended normally after 99 iterations
## 
##   Estimator                                         ML
##   Optimization method                           NLMINB
##   Number of model parameters                        67
## 
##                                                   Used       Total
##   Number of observations                          3825        4051
##   Number of clusters [country]                       8            
## 
## Model Test User Model:
##                                                       
##   Test statistic                                62.544
##   Degrees of freedom                                24
##   P-value (Chi-square)                           0.000
## 
## Parameter Estimates:
## 
##   Standard errors                             Standard
##   Information                                 Observed
##   Observed information based on                Hessian
## 
## 
## Level 1 [within]:
## 
## Regressions:
##                    Estimate  Std.Err  z-value  P(>|z|)
##   hds_T3 ~                                            
##     incm_c_T3         0.543    0.097    5.586    0.000
##     fds_01_T3 (a1)    0.816    0.115    7.113    0.000
##     fds_02_T3 (a2)   -0.532    0.123   -4.322    0.000
##     fds_03_T3 (a3)    0.290    0.149    1.941    0.052
##     fds_04_T3 (a4)    0.678    0.134    5.045    0.000
##     fds_05_T3 (a5)   -0.065    0.153   -0.426    0.670
##     fds_06_T3 (a6)   -0.551    0.138   -3.987    0.000
##     fds_07_T3 (a7)   -0.055    0.163   -0.338    0.735
##     fds_08_T3 (a8)    0.343    0.099    3.443    0.001
##     age_T3            0.104    0.024    4.308    0.000
##     sex_T3            1.709    0.382    4.471    0.000
##     bmi_T3            0.064    0.026    2.487    0.013
##   foodst_01_T3 ~                                      
##     incm_c_T3 (b1)    0.002    0.015    0.127    0.899
##   foodst_02_T3 ~                                      
##     incm_c_T3 (b2)   -0.105    0.013   -8.316    0.000
##   foodst_03_T3 ~                                      
##     incm_c_T3 (b3)   -0.003    0.013   -0.267    0.789
##   foodst_04_T3 ~                                      
##     incm_c_T3 (b4)   -0.039    0.014   -2.665    0.008
##   foodst_05_T3 ~                                      
##     incm_c_T3 (b5)   -0.026    0.011   -2.351    0.019
##   foodst_06_T3 ~                                      
##     incm_c_T3 (b6)    0.068    0.012    5.521    0.000
##   foodst_07_T3 ~                                      
##     incm_c_T3 (b7)    0.011    0.010    1.040    0.298
##   foodst_08_T3 ~                                      
##     incm_c_T3 (b8)   -0.034    0.016   -2.170    0.030
## 
## Covariances:
##                    Estimate  Std.Err  z-value  P(>|z|)
##  .foodst_01_T3 ~~                                     
##    .foodst_02_T3      0.188    0.023    8.285    0.000
##    .foodst_03_T3      0.568    0.025   22.878    0.000
##    .foodst_04_T3      0.569    0.027   20.787    0.000
##    .foodst_05_T3      0.188    0.020    9.317    0.000
##    .foodst_06_T3     -0.099    0.022   -4.497    0.000
##    .foodst_07_T3     -0.085    0.019   -4.586    0.000
##    .foodst_08_T3      0.141    0.028    5.054    0.000
##  .foodst_02_T3 ~~                                     
##    .foodst_03_T3      0.068    0.020    3.482    0.000
##    .foodst_04_T3      0.090    0.022    4.091    0.000
##    .foodst_05_T3      0.041    0.017    2.410    0.016
##    .foodst_06_T3      0.074    0.019    3.938    0.000
##    .foodst_07_T3      0.064    0.016    4.088    0.000
##    .foodst_08_T3     -0.037    0.024   -1.554    0.120
##  .foodst_03_T3 ~~                                     
##    .foodst_04_T3      0.766    0.026   29.860    0.000
##    .foodst_05_T3      0.361    0.018   19.660    0.000
##    .foodst_06_T3     -0.238    0.020  -12.168    0.000
##    .foodst_07_T3     -0.189    0.016  -11.503    0.000
##    .foodst_08_T3      0.174    0.024    7.142    0.000
##  .foodst_04_T3 ~~                                     
##    .foodst_05_T3      0.484    0.021   23.086    0.000
##    .foodst_06_T3     -0.227    0.022  -10.459    0.000
##    .foodst_07_T3     -0.190    0.018  -10.377    0.000
##    .foodst_08_T3      0.199    0.027    7.305    0.000
##  .foodst_05_T3 ~~                                     
##    .foodst_06_T3     -0.229    0.017  -13.471    0.000
##    .foodst_07_T3     -0.172    0.014  -12.078    0.000
##    .foodst_08_T3      0.164    0.021    7.796    0.000
##  .foodst_06_T3 ~~                                     
##    .foodst_07_T3      0.389    0.017   23.426    0.000
##    .foodst_08_T3     -0.067    0.023   -2.886    0.004
##  .foodst_07_T3 ~~                                     
##    .foodst_08_T3     -0.035    0.019   -1.789    0.074
## 
## Intercepts:
##                    Estimate  Std.Err  z-value  P(>|z|)
##    .hds_T3            0.000                           
##    .foodst_01_T3      3.437    0.050   68.690    0.000
##    .foodst_02_T3      2.208    0.042   52.083    0.000
##    .foodst_03_T3      3.979    0.044   91.256    0.000
##    .foodst_04_T3      3.665    0.049   75.202    0.000
##    .foodst_05_T3      4.446    0.038  117.889    0.000
##    .foodst_06_T3      1.727    0.042   41.542    0.000
##    .foodst_07_T3      1.534    0.035   43.834    0.000
##    .foodst_08_T3      3.471    0.052   66.166    0.000
## 
## Variances:
##                    Estimate  Std.Err  z-value  P(>|z|)
##    .hds_T3           66.003    1.511   43.687    0.000
##    .foodst_01_T3      1.638    0.037   43.732    0.000
##    .foodst_02_T3      1.176    0.027   43.732    0.000
##    .foodst_03_T3      1.243    0.028   43.732    0.000
##    .foodst_04_T3      1.553    0.036   43.732    0.000
##    .foodst_05_T3      0.930    0.021   43.732    0.000
##    .foodst_06_T3      1.130    0.026   43.732    0.000
##    .foodst_07_T3      0.801    0.018   43.732    0.000
##    .foodst_08_T3      1.800    0.041   43.732    0.000
## 
## 
## Level 2 [country]:
## 
## Intercepts:
##                    Estimate  Std.Err  z-value  P(>|z|)
##    .hds_T3           10.195    2.004    5.088    0.000
## 
## Variances:
##                    Estimate  Std.Err  z-value  P(>|z|)
##    .hds_T3            5.915    3.038    1.947    0.052
## 
## Defined Parameters:
##                    Estimate  Std.Err  z-value  P(>|z|)
##     ebndfdst_01_T3    0.002    0.012    0.127    0.899
##     ebndfdst_02_T3    0.056    0.015    3.835    0.000
##     ebndfdst_03_T3   -0.001    0.004   -0.265    0.791
##     ebndfdst_04_T3   -0.026    0.011   -2.357    0.018
##     ebndfdst_05_T3    0.002    0.004    0.419    0.675
##     ebndfdst_06_T3   -0.038    0.012   -3.232    0.001
##     ebndfdst_07_T3   -0.001    0.002   -0.322    0.748
##     ebndfdst_08_T3   -0.012    0.006   -1.836    0.066
```

```
parameterEstimates(fit_SEM_inc1, ci=TRUE, level=.99375)
```

```
##                  lhs op           rhs block level             label    est
## 1             hds_T3  ~ income_cat_T3     1     1                    0.543
## 2             hds_T3  ~  foodst_01_T3     1     1                a1  0.816
## 3             hds_T3  ~  foodst_02_T3     1     1                a2 -0.532
## 4             hds_T3  ~  foodst_03_T3     1     1                a3  0.290
## 5             hds_T3  ~  foodst_04_T3     1     1                a4  0.678
## 6             hds_T3  ~  foodst_05_T3     1     1                a5 -0.065
## 7             hds_T3  ~  foodst_06_T3     1     1                a6 -0.551
## 8             hds_T3  ~  foodst_07_T3     1     1                a7 -0.055
## 9             hds_T3  ~  foodst_08_T3     1     1                a8  0.343
## 10            hds_T3  ~        age_T3     1     1                    0.104
## 11            hds_T3  ~        sex_T3     1     1                    1.709
## 12            hds_T3  ~        bmi_T3     1     1                    0.064
## 13      foodst_01_T3  ~ income_cat_T3     1     1                b1  0.002
## 14      foodst_02_T3  ~ income_cat_T3     1     1                b2 -0.105
## 15      foodst_03_T3  ~ income_cat_T3     1     1                b3 -0.003
## 16      foodst_04_T3  ~ income_cat_T3     1     1                b4 -0.039
## 17      foodst_05_T3  ~ income_cat_T3     1     1                b5 -0.026
## 18      foodst_06_T3  ~ income_cat_T3     1     1                b6  0.068
## 19      foodst_07_T3  ~ income_cat_T3     1     1                b7  0.011
## 20      foodst_08_T3  ~ income_cat_T3     1     1                b8 -0.034
## 21      foodst_01_T3 ~~  foodst_02_T3     1     1                    0.188
## 22      foodst_01_T3 ~~  foodst_03_T3     1     1                    0.568
## 23      foodst_01_T3 ~~  foodst_04_T3     1     1                    0.569
## 24      foodst_01_T3 ~~  foodst_05_T3     1     1                    0.188
## 25      foodst_01_T3 ~~  foodst_06_T3     1     1                   -0.099
## 26      foodst_01_T3 ~~  foodst_07_T3     1     1                   -0.085
## 27      foodst_01_T3 ~~  foodst_08_T3     1     1                    0.141
## 28      foodst_02_T3 ~~  foodst_03_T3     1     1                    0.068
## 29      foodst_02_T3 ~~  foodst_04_T3     1     1                    0.090
## 30      foodst_02_T3 ~~  foodst_05_T3     1     1                    0.041
## 31      foodst_02_T3 ~~  foodst_06_T3     1     1                    0.074
## 32      foodst_02_T3 ~~  foodst_07_T3     1     1                    0.064
## 33      foodst_02_T3 ~~  foodst_08_T3     1     1                   -0.037
## 34      foodst_03_T3 ~~  foodst_04_T3     1     1                    0.766
## 35      foodst_03_T3 ~~  foodst_05_T3     1     1                    0.361
## 36      foodst_03_T3 ~~  foodst_06_T3     1     1                   -0.238
## 37      foodst_03_T3 ~~  foodst_07_T3     1     1                   -0.189
## 38      foodst_03_T3 ~~  foodst_08_T3     1     1                    0.174
## 39      foodst_04_T3 ~~  foodst_05_T3     1     1                    0.484
## 40      foodst_04_T3 ~~  foodst_06_T3     1     1                   -0.227
## 41      foodst_04_T3 ~~  foodst_07_T3     1     1                   -0.190
## 42      foodst_04_T3 ~~  foodst_08_T3     1     1                    0.199
## 43      foodst_05_T3 ~~  foodst_06_T3     1     1                   -0.229
## 44      foodst_05_T3 ~~  foodst_07_T3     1     1                   -0.172
## 45      foodst_05_T3 ~~  foodst_08_T3     1     1                    0.164
## 46      foodst_06_T3 ~~  foodst_07_T3     1     1                    0.389
## 47      foodst_06_T3 ~~  foodst_08_T3     1     1                   -0.067
## 48      foodst_07_T3 ~~  foodst_08_T3     1     1                   -0.035
## 49            hds_T3 ~~        hds_T3     1     1                   66.003
## 50      foodst_01_T3 ~~  foodst_01_T3     1     1                    1.638
## 51      foodst_02_T3 ~~  foodst_02_T3     1     1                    1.176
## 52      foodst_03_T3 ~~  foodst_03_T3     1     1                    1.243
## 53      foodst_04_T3 ~~  foodst_04_T3     1     1                    1.553
## 54      foodst_05_T3 ~~  foodst_05_T3     1     1                    0.930
## 55      foodst_06_T3 ~~  foodst_06_T3     1     1                    1.130
## 56      foodst_07_T3 ~~  foodst_07_T3     1     1                    0.801
## 57      foodst_08_T3 ~~  foodst_08_T3     1     1                    1.800
## 58     income_cat_T3 ~~ income_cat_T3     1     1                    1.942
## 59     income_cat_T3 ~~        age_T3     1     1                    0.418
## 60     income_cat_T3 ~~        sex_T3     1     1                   -0.037
## 61     income_cat_T3 ~~        bmi_T3     1     1                   -1.077
## 62            age_T3 ~~        age_T3     1     1                   31.750
## 63            age_T3 ~~        sex_T3     1     1                   -0.468
## 64            age_T3 ~~        bmi_T3     1     1                    2.472
## 65            sex_T3 ~~        sex_T3     1     1                    0.129
## 66            sex_T3 ~~        bmi_T3     1     1                   -0.258
## 67            bmi_T3 ~~        bmi_T3     1     1                   27.514
## 68            hds_T3 ~1                   1     1                    0.000
## 69      foodst_01_T3 ~1                   1     1                    3.437
## 70      foodst_02_T3 ~1                   1     1                    2.208
## 71      foodst_03_T3 ~1                   1     1                    3.979
## 72      foodst_04_T3 ~1                   1     1                    3.665
## 73      foodst_05_T3 ~1                   1     1                    4.446
## 74      foodst_06_T3 ~1                   1     1                    1.727
## 75      foodst_07_T3 ~1                   1     1                    1.534
## 76      foodst_08_T3 ~1                   1     1                    3.471
## 77     income_cat_T3 ~1                   1     1                    3.069
## 78            age_T3 ~1                   1     1                   41.890
## 79            sex_T3 ~1                   1     1                    1.848
## 80            bmi_T3 ~1                   1     1                   26.041
## 81            hds_T3 ~1                   2     2                   10.195
## 82            hds_T3 ~~        hds_T3     2     2                    5.915
## 83 ebindfoodst_01_T3 :=         a1*b1     0     0 ebindfoodst_01_T3  0.002
## 84 ebindfoodst_02_T3 :=         a2*b2     0     0 ebindfoodst_02_T3  0.056
## 85 ebindfoodst_03_T3 :=         a3*b3     0     0 ebindfoodst_03_T3 -0.001
## 86 ebindfoodst_04_T3 :=         a4*b4     0     0 ebindfoodst_04_T3 -0.026
## 87 ebindfoodst_05_T3 :=         a5*b5     0     0 ebindfoodst_05_T3  0.002
## 88 ebindfoodst_06_T3 :=         a6*b6     0     0 ebindfoodst_06_T3 -0.038
## 89 ebindfoodst_07_T3 :=         a7*b7     0     0 ebindfoodst_07_T3 -0.001
## 90 ebindfoodst_08_T3 :=         a8*b8     0     0 ebindfoodst_08_T3 -0.012
##       se       z pvalue ci.lower ci.upper
## 1  0.097   5.586  0.000    0.277    0.809
## 2  0.115   7.113  0.000    0.502    1.130
## 3  0.123  -4.322  0.000   -0.869   -0.195
## 4  0.149   1.941  0.052   -0.119    0.699
## 5  0.134   5.045  0.000    0.310    1.045
## 6  0.153  -0.426  0.670   -0.485    0.354
## 7  0.138  -3.987  0.000   -0.929   -0.173
## 8  0.163  -0.338  0.735   -0.502    0.391
## 9  0.099   3.443  0.001    0.071    0.615
## 10 0.024   4.308  0.000    0.038    0.170
## 11 0.382   4.471  0.000    0.664    2.754
## 12 0.026   2.487  0.013   -0.006    0.135
## 13 0.015   0.127  0.899   -0.039    0.042
## 14 0.013  -8.316  0.000   -0.139   -0.070
## 15 0.013  -0.267  0.789   -0.039    0.032
## 16 0.014  -2.665  0.008   -0.078    0.001
## 17 0.011  -2.351  0.019   -0.057    0.004
## 18 0.012   5.521  0.000    0.034    0.102
## 19 0.010   1.040  0.298   -0.018    0.039
## 20 0.016  -2.170  0.030   -0.076    0.009
## 21 0.023   8.285  0.000    0.126    0.249
## 22 0.025  22.878  0.000    0.500    0.636
## 23 0.027  20.787  0.000    0.494    0.644
## 24 0.020   9.317  0.000    0.133    0.243
## 25 0.022  -4.497  0.000   -0.159   -0.039
## 26 0.019  -4.586  0.000   -0.136   -0.034
## 27 0.028   5.054  0.000    0.065    0.217
## 28 0.020   3.482  0.000    0.015    0.122
## 29 0.022   4.091  0.000    0.030    0.149
## 30 0.017   2.410  0.016   -0.005    0.087
## 31 0.019   3.938  0.000    0.022    0.125
## 32 0.016   4.088  0.000    0.021    0.107
## 33 0.024  -1.554  0.120   -0.101    0.028
## 34 0.026  29.860  0.000    0.696    0.836
## 35 0.018  19.660  0.000    0.310    0.411
## 36 0.020 -12.168  0.000   -0.291   -0.184
## 37 0.016 -11.503  0.000   -0.234   -0.144
## 38 0.024   7.142  0.000    0.107    0.241
## 39 0.021  23.086  0.000    0.426    0.541
## 40 0.022 -10.459  0.000   -0.287   -0.168
## 41 0.018 -10.377  0.000   -0.240   -0.140
## 42 0.027   7.305  0.000    0.124    0.273
## 43 0.017 -13.471  0.000   -0.275   -0.182
## 44 0.014 -12.078  0.000   -0.211   -0.133
## 45 0.021   7.796  0.000    0.107    0.222
## 46 0.017  23.426  0.000    0.344    0.435
## 47 0.023  -2.886  0.004   -0.130   -0.003
## 48 0.019  -1.789  0.074   -0.088    0.018
## 49 1.511  43.687  0.000   61.872   70.134
## 50 0.037  43.732  0.000    1.535    1.740
## 51 0.027  43.732  0.000    1.102    1.249
## 52 0.028  43.732  0.000    1.166    1.321
## 53 0.036  43.732  0.000    1.456    1.650
## 54 0.021  43.732  0.000    0.872    0.988
## 55 0.026  43.732  0.000    1.059    1.201
## 56 0.018  43.732  0.000    0.751    0.851
## 57 0.041  43.732  0.000    1.687    1.912
## 58 0.000      NA     NA    1.942    1.942
## 59 0.000      NA     NA    0.418    0.418
## 60 0.000      NA     NA   -0.037   -0.037
## 61 0.000      NA     NA   -1.077   -1.077
## 62 0.000      NA     NA   31.750   31.750
## 63 0.000      NA     NA   -0.468   -0.468
## 64 0.000      NA     NA    2.472    2.472
## 65 0.000      NA     NA    0.129    0.129
## 66 0.000      NA     NA   -0.258   -0.258
## 67 0.000      NA     NA   27.514   27.514
## 68 0.000      NA     NA    0.000    0.000
## 69 0.050  68.690  0.000    3.301    3.574
## 70 0.042  52.083  0.000    2.092    2.324
## 71 0.044  91.256  0.000    3.860    4.098
## 72 0.049  75.202  0.000    3.532    3.798
## 73 0.038 117.889  0.000    4.343    4.549
## 74 0.042  41.542  0.000    1.613    1.841
## 75 0.035  43.834  0.000    1.438    1.630
## 76 0.052  66.166  0.000    3.328    3.615
## 77 0.000      NA     NA    3.069    3.069
## 78 0.000      NA     NA   41.890   41.890
## 79 0.000      NA     NA    1.848    1.848
## 80 0.000      NA     NA   26.041   26.041
## 81 2.004   5.088  0.000    4.716   15.675
## 82 3.038   1.947  0.052   -2.393   14.222
## 83 0.012   0.127  0.899   -0.032    0.035
## 84 0.015   3.835  0.000    0.016    0.095
## 85 0.004  -0.265  0.791   -0.011    0.009
## 86 0.011  -2.357  0.018   -0.056    0.004
## 87 0.004   0.419  0.675   -0.009    0.013
## 88 0.012  -3.232  0.001   -0.069   -0.006
## 89 0.002  -0.322  0.748   -0.006    0.004
## 90 0.006  -1.836  0.066   -0.029    0.006
```

### 4.2.3 Migration background

Controlled for age, sex, BMI

```
SEM_model_mig <- '
    level: 1
    hds_T3 ~ migration + a1*foodst_01_T3 + a2*foodst_02_T3 + a3*foodst_03_T3 + a4*foodst_04_T3 + a5*foodst_05_T3 + a6*foodst_06_T3 + a7*foodst_07_T3 + a8*foodst_08_T3 + age_T3 + sex_T3 + bmi_T3
    foodst_01_T3 ~ b1*migration
    foodst_02_T3 ~ b2*migration
    foodst_03_T3 ~ b3*migration
    foodst_04_T3 ~ b4*migration
    foodst_05_T3 ~ b5*migration
    foodst_06_T3 ~ b6*migration
    foodst_07_T3 ~ b7*migration
    foodst_08_T3 ~ b8*migration
    foodst_01_T3 ~~ foodst_02_T3 + foodst_03_T3 + foodst_04_T3 + foodst_05_T3 + foodst_06_T3 + foodst_07_T3 + foodst_08_T3
    foodst_02_T3 ~~ foodst_03_T3 + foodst_04_T3 + foodst_05_T3 + foodst_06_T3 + foodst_07_T3 + foodst_08_T3
    foodst_03_T3 ~~ foodst_04_T3 + foodst_05_T3 + foodst_06_T3 + foodst_07_T3 + foodst_08_T3
    foodst_04_T3 ~~ foodst_05_T3 + foodst_06_T3 + foodst_07_T3 + foodst_08_T3
    foodst_05_T3 ~~ foodst_06_T3 + foodst_07_T3 + foodst_08_T3
    foodst_06_T3 ~~ foodst_07_T3 + foodst_08_T3 
    foodst_07_T3 ~~ foodst_08_T3
    ebindfoodst_01_T3 := a1*b1 
    ebindfoodst_02_T3 := a2*b2
    ebindfoodst_03_T3 := a3*b3
    ebindfoodst_04_T3 := a4*b4
    ebindfoodst_05_T3 := a5*b5
    ebindfoodst_06_T3 := a6*b6
    ebindfoodst_07_T3 := a7*b7
    ebindfoodst_08_T3 := a8*b8

    level: 2
    hds_T3 ~ 1

'

fit_SEM_mig <- sem(model = SEM_model_mig, data = subset, cluster = "country")

summary(fit_SEM_mig)
```

```
## lavaan 0.6.16 ended normally after 112 iterations
## 
##   Estimator                                         ML
##   Optimization method                           NLMINB
##   Number of model parameters                        67
## 
##   Number of observations                          4051
##   Number of clusters [country]                       8
## 
## Model Test User Model:
##                                                       
##   Test statistic                               100.641
##   Degrees of freedom                                24
##   P-value (Chi-square)                           0.000
## 
## Parameter Estimates:
## 
##   Standard errors                             Standard
##   Information                                 Observed
##   Observed information based on                Hessian
## 
## 
## Level 1 [within]:
## 
## Regressions:
##                    Estimate  Std.Err  z-value  P(>|z|)
##   hds_T3 ~                                            
##     migration        -0.550    0.386   -1.425    0.154
##     fds_01_T3 (a1)    0.825    0.112    7.392    0.000
##     fds_02_T3 (a2)   -0.650    0.118   -5.497    0.000
##     fds_03_T3 (a3)    0.342    0.144    2.372    0.018
##     fds_04_T3 (a4)    0.615    0.130    4.729    0.000
##     fds_05_T3 (a5)   -0.109    0.150   -0.721    0.471
##     fds_06_T3 (a6)   -0.508    0.135   -3.770    0.000
##     fds_07_T3 (a7)   -0.056    0.160   -0.352    0.725
##     fds_08_T3 (a8)    0.337    0.096    3.498    0.000
##     age_T3            0.109    0.024    4.635    0.000
##     sex_T3            1.424    0.375    3.795    0.000
##     bmi_T3            0.046    0.025    1.880    0.060
##   foodst_01_T3 ~                                      
##     migration (b1)    0.120    0.061    1.989    0.047
##   foodst_02_T3 ~                                      
##     migration (b2)    0.142    0.052    2.739    0.006
##   foodst_03_T3 ~                                      
##     migration (b3)    0.043    0.053    0.812    0.417
##   foodst_04_T3 ~                                      
##     migration (b4)    0.169    0.059    2.873    0.004
##   foodst_05_T3 ~                                      
##     migration (b5)   -0.016    0.045   -0.343    0.731
##   foodst_06_T3 ~                                      
##     migration (b6)   -0.085    0.050   -1.696    0.090
##   foodst_07_T3 ~                                      
##     migration (b7)    0.011    0.042    0.249    0.803
##   foodst_08_T3 ~                                      
##     migration (b8)    0.093    0.064    1.459    0.145
## 
## Covariances:
##                    Estimate  Std.Err  z-value  P(>|z|)
##  .foodst_01_T3 ~~                                     
##    .foodst_02_T3      0.184    0.022    8.222    0.000
##    .foodst_03_T3      0.577    0.024   23.751    0.000
##    .foodst_04_T3      0.568    0.027   21.304    0.000
##    .foodst_05_T3      0.190    0.020    9.703    0.000
##    .foodst_06_T3     -0.101    0.022   -4.678    0.000
##    .foodst_07_T3     -0.090    0.018   -4.976    0.000
##    .foodst_08_T3      0.145    0.027    5.301    0.000
##  .foodst_02_T3 ~~                                     
##    .foodst_03_T3      0.061    0.019    3.131    0.002
##    .foodst_04_T3      0.101    0.022    4.651    0.000
##    .foodst_05_T3      0.049    0.017    2.955    0.003
##    .foodst_06_T3      0.060    0.018    3.273    0.001
##    .foodst_07_T3      0.065    0.015    4.213    0.000
##    .foodst_08_T3     -0.040    0.023   -1.735    0.083
##  .foodst_03_T3 ~~                                     
##    .foodst_04_T3      0.756    0.025   30.325    0.000
##    .foodst_05_T3      0.356    0.018   20.078    0.000
##    .foodst_06_T3     -0.231    0.019  -12.112    0.000
##    .foodst_07_T3     -0.186    0.016  -11.657    0.000
##    .foodst_08_T3      0.174    0.024    7.283    0.000
##  .foodst_04_T3 ~~                                     
##    .foodst_05_T3      0.483    0.020   23.839    0.000
##    .foodst_06_T3     -0.226    0.021  -10.689    0.000
##    .foodst_07_T3     -0.192    0.018  -10.796    0.000
##    .foodst_08_T3      0.204    0.027    7.674    0.000
##  .foodst_05_T3 ~~                                     
##    .foodst_06_T3     -0.228    0.016  -13.849    0.000
##    .foodst_07_T3     -0.170    0.014  -12.413    0.000
##    .foodst_08_T3      0.166    0.020    8.121    0.000
##  .foodst_06_T3 ~~                                     
##    .foodst_07_T3      0.396    0.016   24.468    0.000
##    .foodst_08_T3     -0.057    0.023   -2.543    0.011
##  .foodst_07_T3 ~~                                     
##    .foodst_08_T3     -0.035    0.019   -1.847    0.065
## 
## Intercepts:
##                    Estimate  Std.Err  z-value  P(>|z|)
##    .hds_T3            0.000                           
##    .foodst_01_T3      3.314    0.071   46.550    0.000
##    .foodst_02_T3      1.741    0.061   28.510    0.000
##    .foodst_03_T3      3.920    0.062   63.206    0.000
##    .foodst_04_T3      3.366    0.069   48.669    0.000
##    .foodst_05_T3      4.391    0.053   82.550    0.000
##    .foodst_06_T3      2.017    0.059   34.130    0.000
##    .foodst_07_T3      1.551    0.050   31.332    0.000
##    .foodst_08_T3      3.262    0.075   43.578    0.000
## 
## Variances:
##                    Estimate  Std.Err  z-value  P(>|z|)
##    .hds_T3           66.360    1.476   44.962    0.000
##    .foodst_01_T3      1.647    0.037   45.005    0.000
##    .foodst_02_T3      1.212    0.027   45.005    0.000
##    .foodst_03_T3      1.250    0.028   45.005    0.000
##    .foodst_04_T3      1.555    0.035   45.005    0.000
##    .foodst_05_T3      0.920    0.020   45.006    0.000
##    .foodst_06_T3      1.135    0.025   45.005    0.000
##    .foodst_07_T3      0.797    0.018   45.006    0.000
##    .foodst_08_T3      1.821    0.040   45.006    0.000
## 
## 
## Level 2 [country]:
## 
## Intercepts:
##                    Estimate  Std.Err  z-value  P(>|z|)
##    .hds_T3           13.554    1.974    6.867    0.000
## 
## Variances:
##                    Estimate  Std.Err  z-value  P(>|z|)
##    .hds_T3            6.250    3.204    1.951    0.051
## 
## Defined Parameters:
##                    Estimate  Std.Err  z-value  P(>|z|)
##     ebndfdst_01_T3    0.099    0.052    1.921    0.055
##     ebndfdst_02_T3   -0.093    0.038   -2.452    0.014
##     ebndfdst_03_T3    0.015    0.019    0.768    0.442
##     ebndfdst_04_T3    0.104    0.042    2.455    0.014
##     ebndfdst_05_T3    0.002    0.005    0.310    0.757
##     ebndfdst_06_T3    0.043    0.028    1.547    0.122
##     ebndfdst_07_T3   -0.001    0.003   -0.204    0.839
##     ebndfdst_08_T3    0.031    0.023    1.346    0.178
```

```
parameterEstimates(fit_SEM_mig, ci=TRUE, level=.99375)
```

```
##                  lhs op          rhs block level             label    est    se
## 1             hds_T3  ~    migration     1     1                   -0.550 0.386
## 2             hds_T3  ~ foodst_01_T3     1     1                a1  0.825 0.112
## 3             hds_T3  ~ foodst_02_T3     1     1                a2 -0.650 0.118
## 4             hds_T3  ~ foodst_03_T3     1     1                a3  0.342 0.144
## 5             hds_T3  ~ foodst_04_T3     1     1                a4  0.615 0.130
## 6             hds_T3  ~ foodst_05_T3     1     1                a5 -0.109 0.150
## 7             hds_T3  ~ foodst_06_T3     1     1                a6 -0.508 0.135
## 8             hds_T3  ~ foodst_07_T3     1     1                a7 -0.056 0.160
## 9             hds_T3  ~ foodst_08_T3     1     1                a8  0.337 0.096
## 10            hds_T3  ~       age_T3     1     1                    0.109 0.024
## 11            hds_T3  ~       sex_T3     1     1                    1.424 0.375
## 12            hds_T3  ~       bmi_T3     1     1                    0.046 0.025
## 13      foodst_01_T3  ~    migration     1     1                b1  0.120 0.061
## 14      foodst_02_T3  ~    migration     1     1                b2  0.142 0.052
## 15      foodst_03_T3  ~    migration     1     1                b3  0.043 0.053
## 16      foodst_04_T3  ~    migration     1     1                b4  0.169 0.059
## 17      foodst_05_T3  ~    migration     1     1                b5 -0.016 0.045
## 18      foodst_06_T3  ~    migration     1     1                b6 -0.085 0.050
## 19      foodst_07_T3  ~    migration     1     1                b7  0.011 0.042
## 20      foodst_08_T3  ~    migration     1     1                b8  0.093 0.064
## 21      foodst_01_T3 ~~ foodst_02_T3     1     1                    0.184 0.022
## 22      foodst_01_T3 ~~ foodst_03_T3     1     1                    0.577 0.024
## 23      foodst_01_T3 ~~ foodst_04_T3     1     1                    0.568 0.027
## 24      foodst_01_T3 ~~ foodst_05_T3     1     1                    0.190 0.020
## 25      foodst_01_T3 ~~ foodst_06_T3     1     1                   -0.101 0.022
## 26      foodst_01_T3 ~~ foodst_07_T3     1     1                   -0.090 0.018
## 27      foodst_01_T3 ~~ foodst_08_T3     1     1                    0.145 0.027
## 28      foodst_02_T3 ~~ foodst_03_T3     1     1                    0.061 0.019
## 29      foodst_02_T3 ~~ foodst_04_T3     1     1                    0.101 0.022
## 30      foodst_02_T3 ~~ foodst_05_T3     1     1                    0.049 0.017
## 31      foodst_02_T3 ~~ foodst_06_T3     1     1                    0.060 0.018
## 32      foodst_02_T3 ~~ foodst_07_T3     1     1                    0.065 0.015
## 33      foodst_02_T3 ~~ foodst_08_T3     1     1                   -0.040 0.023
## 34      foodst_03_T3 ~~ foodst_04_T3     1     1                    0.756 0.025
## 35      foodst_03_T3 ~~ foodst_05_T3     1     1                    0.356 0.018
## 36      foodst_03_T3 ~~ foodst_06_T3     1     1                   -0.231 0.019
## 37      foodst_03_T3 ~~ foodst_07_T3     1     1                   -0.186 0.016
## 38      foodst_03_T3 ~~ foodst_08_T3     1     1                    0.174 0.024
## 39      foodst_04_T3 ~~ foodst_05_T3     1     1                    0.483 0.020
## 40      foodst_04_T3 ~~ foodst_06_T3     1     1                   -0.226 0.021
## 41      foodst_04_T3 ~~ foodst_07_T3     1     1                   -0.192 0.018
## 42      foodst_04_T3 ~~ foodst_08_T3     1     1                    0.204 0.027
## 43      foodst_05_T3 ~~ foodst_06_T3     1     1                   -0.228 0.016
## 44      foodst_05_T3 ~~ foodst_07_T3     1     1                   -0.170 0.014
## 45      foodst_05_T3 ~~ foodst_08_T3     1     1                    0.166 0.020
## 46      foodst_06_T3 ~~ foodst_07_T3     1     1                    0.396 0.016
## 47      foodst_06_T3 ~~ foodst_08_T3     1     1                   -0.057 0.023
## 48      foodst_07_T3 ~~ foodst_08_T3     1     1                   -0.035 0.019
## 49            hds_T3 ~~       hds_T3     1     1                   66.360 1.476
## 50      foodst_01_T3 ~~ foodst_01_T3     1     1                    1.647 0.037
## 51      foodst_02_T3 ~~ foodst_02_T3     1     1                    1.212 0.027
## 52      foodst_03_T3 ~~ foodst_03_T3     1     1                    1.250 0.028
## 53      foodst_04_T3 ~~ foodst_04_T3     1     1                    1.555 0.035
## 54      foodst_05_T3 ~~ foodst_05_T3     1     1                    0.920 0.020
## 55      foodst_06_T3 ~~ foodst_06_T3     1     1                    1.135 0.025
## 56      foodst_07_T3 ~~ foodst_07_T3     1     1                    0.797 0.018
## 57      foodst_08_T3 ~~ foodst_08_T3     1     1                    1.821 0.040
## 58         migration ~~    migration     1     1                    0.111 0.000
## 59         migration ~~       age_T3     1     1                    0.032 0.000
## 60         migration ~~       sex_T3     1     1                   -0.004 0.000
## 61         migration ~~       bmi_T3     1     1                    0.073 0.000
## 62            age_T3 ~~       age_T3     1     1                   31.686 0.000
## 63            age_T3 ~~       sex_T3     1     1                   -0.456 0.000
## 64            age_T3 ~~       bmi_T3     1     1                    2.483 0.000
## 65            sex_T3 ~~       sex_T3     1     1                    0.126 0.000
## 66            sex_T3 ~~       bmi_T3     1     1                   -0.249 0.000
## 67            bmi_T3 ~~       bmi_T3     1     1                   27.722 0.000
## 68            hds_T3 ~1                  1     1                    0.000 0.000
## 69      foodst_01_T3 ~1                  1     1                    3.314 0.071
## 70      foodst_02_T3 ~1                  1     1                    1.741 0.061
## 71      foodst_03_T3 ~1                  1     1                    3.920 0.062
## 72      foodst_04_T3 ~1                  1     1                    3.366 0.069
## 73      foodst_05_T3 ~1                  1     1                    4.391 0.053
## 74      foodst_06_T3 ~1                  1     1                    2.017 0.059
## 75      foodst_07_T3 ~1                  1     1                    1.551 0.050
## 76      foodst_08_T3 ~1                  1     1                    3.262 0.075
## 77         migration ~1                  1     1                    1.127 0.000
## 78            age_T3 ~1                  1     1                   41.916 0.000
## 79            sex_T3 ~1                  1     1                    1.852 0.000
## 80            bmi_T3 ~1                  1     1                   26.058 0.000
## 81            hds_T3 ~1                  2     2                   13.554 1.974
## 82            hds_T3 ~~       hds_T3     2     2                    6.250 3.204
## 83 ebindfoodst_01_T3 :=        a1*b1     0     0 ebindfoodst_01_T3  0.099 0.052
## 84 ebindfoodst_02_T3 :=        a2*b2     0     0 ebindfoodst_02_T3 -0.093 0.038
## 85 ebindfoodst_03_T3 :=        a3*b3     0     0 ebindfoodst_03_T3  0.015 0.019
## 86 ebindfoodst_04_T3 :=        a4*b4     0     0 ebindfoodst_04_T3  0.104 0.042
## 87 ebindfoodst_05_T3 :=        a5*b5     0     0 ebindfoodst_05_T3  0.002 0.005
## 88 ebindfoodst_06_T3 :=        a6*b6     0     0 ebindfoodst_06_T3  0.043 0.028
## 89 ebindfoodst_07_T3 :=        a7*b7     0     0 ebindfoodst_07_T3 -0.001 0.003
## 90 ebindfoodst_08_T3 :=        a8*b8     0     0 ebindfoodst_08_T3  0.031 0.023
##          z pvalue ci.lower ci.upper
## 1   -1.425  0.154   -1.606    0.506
## 2    7.392  0.000    0.520    1.130
## 3   -5.497  0.000   -0.973   -0.327
## 4    2.372  0.018   -0.052    0.737
## 5    4.729  0.000    0.259    0.971
## 6   -0.721  0.471   -0.520    0.303
## 7   -3.770  0.000   -0.877   -0.140
## 8   -0.352  0.725   -0.494    0.382
## 9    3.498  0.000    0.074    0.601
## 10   4.635  0.000    0.045    0.173
## 11   3.795  0.000    0.398    2.449
## 12   1.880  0.060   -0.021    0.114
## 13   1.989  0.047   -0.045    0.286
## 14   2.739  0.006    0.000    0.284
## 15   0.812  0.417   -0.101    0.187
## 16   2.873  0.004    0.008    0.330
## 17  -0.343  0.731   -0.139    0.108
## 18  -1.696  0.090   -0.223    0.052
## 19   0.249  0.803   -0.105    0.126
## 20   1.459  0.145   -0.081    0.267
## 21   8.222  0.000    0.123    0.245
## 22  23.751  0.000    0.511    0.644
## 23  21.304  0.000    0.495    0.641
## 24   9.703  0.000    0.136    0.243
## 25  -4.678  0.000   -0.160   -0.042
## 26  -4.976  0.000   -0.139   -0.040
## 27   5.301  0.000    0.070    0.219
## 28   3.131  0.002    0.008    0.114
## 29   4.651  0.000    0.041    0.160
## 30   2.955  0.003    0.004    0.094
## 31   3.273  0.001    0.010    0.111
## 32   4.213  0.000    0.023    0.107
## 33  -1.735  0.083   -0.104    0.023
## 34  30.325  0.000    0.687    0.824
## 35  20.078  0.000    0.308    0.405
## 36 -12.112  0.000   -0.283   -0.179
## 37 -11.657  0.000   -0.230   -0.142
## 38   7.283  0.000    0.109    0.239
## 39  23.839  0.000    0.428    0.538
## 40 -10.689  0.000   -0.284   -0.168
## 41 -10.796  0.000   -0.240   -0.143
## 42   7.674  0.000    0.132    0.277
## 43 -13.849  0.000   -0.273   -0.183
## 44 -12.413  0.000   -0.208   -0.133
## 45   8.121  0.000    0.110    0.223
## 46  24.468  0.000    0.352    0.440
## 47  -2.543  0.011   -0.119    0.004
## 48  -1.847  0.065   -0.087    0.017
## 49  44.962  0.000   62.324   70.396
## 50  45.005  0.000    1.547    1.747
## 51  45.005  0.000    1.138    1.285
## 52  45.005  0.000    1.174    1.326
## 53  45.005  0.000    1.460    1.649
## 54  45.006  0.000    0.864    0.976
## 55  45.005  0.000    1.066    1.204
## 56  45.006  0.000    0.748    0.845
## 57  45.006  0.000    1.710    1.932
## 58      NA     NA    0.111    0.111
## 59      NA     NA    0.032    0.032
## 60      NA     NA   -0.004   -0.004
## 61      NA     NA    0.073    0.073
## 62      NA     NA   31.686   31.686
## 63      NA     NA   -0.456   -0.456
## 64      NA     NA    2.483    2.483
## 65      NA     NA    0.126    0.126
## 66      NA     NA   -0.249   -0.249
## 67      NA     NA   27.722   27.722
## 68      NA     NA    0.000    0.000
## 69  46.550  0.000    3.119    3.508
## 70  28.510  0.000    1.574    1.908
## 71  63.206  0.000    3.751    4.090
## 72  48.669  0.000    3.177    3.555
## 73  82.550  0.000    4.246    4.536
## 74  34.130  0.000    1.855    2.178
## 75  31.332  0.000    1.416    1.686
## 76  43.578  0.000    3.057    3.466
## 77      NA     NA    1.127    1.127
## 78      NA     NA   41.916   41.916
## 79      NA     NA    1.852    1.852
## 80      NA     NA   26.058   26.058
## 81   6.867  0.000    8.157   18.950
## 82   1.951  0.051   -2.511   15.011
## 83   1.921  0.055   -0.042    0.241
## 84  -2.452  0.014   -0.196    0.011
## 85   0.768  0.442   -0.038    0.067
## 86   2.455  0.014   -0.012    0.220
## 87   0.310  0.757   -0.013    0.017
## 88   1.547  0.122   -0.033    0.120
## 89  -0.204  0.839   -0.009    0.007
## 90   1.346  0.178   -0.032    0.095
```

### 4.2.4 Unemployment in household

Controlled for age, sex, BMI

```
SEM_model_une <- '
    level: 1
    hds_T3 ~ unemploy + a1*foodst_01_T3 + a2*foodst_02_T3 + a3*foodst_03_T3 + a4*foodst_04_T3 + a5*foodst_05_T3 + a6*foodst_06_T3 + a7*foodst_07_T3 + a8*foodst_08_T3 + age_T3 + sex_T3 + bmi_T3
    foodst_01_T3 ~ b1*unemploy
    foodst_02_T3 ~ b2*unemploy
    foodst_03_T3 ~ b3*unemploy
    foodst_04_T3 ~ b4*unemploy
    foodst_05_T3 ~ b5*unemploy
    foodst_06_T3 ~ b6*unemploy
    foodst_07_T3 ~ b7*unemploy
    foodst_08_T3 ~ b8*unemploy
    foodst_01_T3 ~~ foodst_02_T3 + foodst_03_T3 + foodst_04_T3 + foodst_05_T3 + foodst_06_T3 + foodst_07_T3 + foodst_08_T3
    foodst_02_T3 ~~ foodst_03_T3 + foodst_04_T3 + foodst_05_T3 + foodst_06_T3 + foodst_07_T3 + foodst_08_T3
    foodst_03_T3 ~~ foodst_04_T3 + foodst_05_T3 + foodst_06_T3 + foodst_07_T3 + foodst_08_T3
    foodst_04_T3 ~~ foodst_05_T3 + foodst_06_T3 + foodst_07_T3 + foodst_08_T3
    foodst_05_T3 ~~ foodst_06_T3 + foodst_07_T3 + foodst_08_T3
    foodst_06_T3 ~~ foodst_07_T3 + foodst_08_T3 
    foodst_07_T3 ~~ foodst_08_T3
    ebindfoodst_01_T3 := a1*b1 
    ebindfoodst_02_T3 := a2*b2
    ebindfoodst_03_T3 := a3*b3
    ebindfoodst_04_T3 := a4*b4
    ebindfoodst_05_T3 := a5*b5
    ebindfoodst_06_T3 := a6*b6
    ebindfoodst_07_T3 := a7*b7
    ebindfoodst_08_T3 := a8*b8

    level: 2
    hds_T3 ~ 1

'

fit_SEM_une <- sem(model = SEM_model_une, data = subset, cluster = "country")

summary(fit_SEM_une)
```

```
## lavaan 0.6.16 ended normally after 131 iterations
## 
##   Estimator                                         ML
##   Optimization method                           NLMINB
##   Number of model parameters                        67
## 
##   Number of observations                          4051
##   Number of clusters [country]                       8
## 
## Model Test User Model:
##                                                       
##   Test statistic                                98.855
##   Degrees of freedom                                24
##   P-value (Chi-square)                           0.000
## 
## Parameter Estimates:
## 
##   Standard errors                             Standard
##   Information                                 Observed
##   Observed information based on                Hessian
## 
## 
## Level 1 [within]:
## 
## Regressions:
##                    Estimate  Std.Err  z-value  P(>|z|)
##   hds_T3 ~                                            
##     unemploy         -1.408    0.454   -3.104    0.002
##     fds_01_T3 (a1)    0.819    0.111    7.349    0.000
##     fds_02_T3 (a2)   -0.649    0.118   -5.496    0.000
##     fds_03_T3 (a3)    0.338    0.144    2.344    0.019
##     fds_04_T3 (a4)    0.619    0.130    4.768    0.000
##     fds_05_T3 (a5)   -0.096    0.150   -0.640    0.522
##     fds_06_T3 (a6)   -0.514    0.135   -3.819    0.000
##     fds_07_T3 (a7)   -0.064    0.160   -0.401    0.688
##     fds_08_T3 (a8)    0.331    0.096    3.437    0.001
##     age_T3            0.107    0.023    4.577    0.000
##     sex_T3            1.469    0.375    3.919    0.000
##     bmi_T3            0.052    0.025    2.084    0.037
##   foodst_01_T3 ~                                      
##     unemploy  (b1)    0.002    0.071    0.024    0.981
##   foodst_02_T3 ~                                      
##     unemploy  (b2)    0.099    0.061    1.626    0.104
##   foodst_03_T3 ~                                      
##     unemploy  (b3)    0.019    0.062    0.309    0.757
##   foodst_04_T3 ~                                      
##     unemploy  (b4)    0.151    0.069    2.187    0.029
##   foodst_05_T3 ~                                      
##     unemploy  (b5)    0.097    0.053    1.829    0.067
##   foodst_06_T3 ~                                      
##     unemploy  (b6)   -0.162    0.059   -2.743    0.006
##   foodst_07_T3 ~                                      
##     unemploy  (b7)   -0.080    0.049   -1.610    0.107
##   foodst_08_T3 ~                                      
##     unemploy  (b8)   -0.012    0.075   -0.156    0.876
## 
## Covariances:
##                    Estimate  Std.Err  z-value  P(>|z|)
##  .foodst_01_T3 ~~                                     
##    .foodst_02_T3      0.186    0.022    8.296    0.000
##    .foodst_03_T3      0.578    0.024   23.760    0.000
##    .foodst_04_T3      0.571    0.027   21.361    0.000
##    .foodst_05_T3      0.190    0.020    9.691    0.000
##    .foodst_06_T3     -0.102    0.022   -4.730    0.000
##    .foodst_07_T3     -0.090    0.018   -4.967    0.000
##    .foodst_08_T3      0.146    0.027    5.342    0.000
##  .foodst_02_T3 ~~                                     
##    .foodst_03_T3      0.061    0.019    3.156    0.002
##    .foodst_04_T3      0.102    0.022    4.713    0.000
##    .foodst_05_T3      0.048    0.017    2.893    0.004
##    .foodst_06_T3      0.060    0.018    3.271    0.001
##    .foodst_07_T3      0.066    0.015    4.264    0.000
##    .foodst_08_T3     -0.039    0.023   -1.667    0.096
##  .foodst_03_T3 ~~                                     
##    .foodst_04_T3      0.756    0.025   30.331    0.000
##    .foodst_05_T3      0.356    0.018   20.072    0.000
##    .foodst_06_T3     -0.231    0.019  -12.126    0.000
##    .foodst_07_T3     -0.186    0.016  -11.649    0.000
##    .foodst_08_T3      0.174    0.024    7.300    0.000
##  .foodst_04_T3 ~~                                     
##    .foodst_05_T3      0.482    0.020   23.776    0.000
##    .foodst_06_T3     -0.226    0.021  -10.673    0.000
##    .foodst_07_T3     -0.190    0.018  -10.731    0.000
##    .foodst_08_T3      0.206    0.027    7.738    0.000
##  .foodst_05_T3 ~~                                     
##    .foodst_06_T3     -0.226    0.016  -13.779    0.000
##    .foodst_07_T3     -0.170    0.014  -12.379    0.000
##    .foodst_08_T3      0.166    0.020    8.118    0.000
##  .foodst_06_T3 ~~                                     
##    .foodst_07_T3      0.395    0.016   24.427    0.000
##    .foodst_08_T3     -0.059    0.023   -2.589    0.010
##  .foodst_07_T3 ~~                                     
##    .foodst_08_T3     -0.035    0.019   -1.846    0.065
## 
## Intercepts:
##                    Estimate  Std.Err  z-value  P(>|z|)
##    .hds_T3            0.000                           
##    .foodst_01_T3      3.447    0.080   43.082    0.000
##    .foodst_02_T3      1.793    0.069   26.122    0.000
##    .foodst_03_T3      3.948    0.070   56.643    0.000
##    .foodst_04_T3      3.392    0.078   43.631    0.000
##    .foodst_05_T3      4.268    0.060   71.434    0.000
##    .foodst_06_T3      2.097    0.066   31.599    0.000
##    .foodst_07_T3      1.649    0.056   29.664    0.000
##    .foodst_08_T3      3.379    0.084   40.169    0.000
## 
## Variances:
##                    Estimate  Std.Err  z-value  P(>|z|)
##    .hds_T3           66.236    1.473   44.962    0.000
##    .foodst_01_T3      1.649    0.037   45.006    0.000
##    .foodst_02_T3      1.213    0.027   45.006    0.000
##    .foodst_03_T3      1.251    0.028   45.005    0.000
##    .foodst_04_T3      1.556    0.035   45.005    0.000
##    .foodst_05_T3      0.919    0.020   45.006    0.000
##    .foodst_06_T3      1.134    0.025   45.005    0.000
##    .foodst_07_T3      0.796    0.018   45.006    0.000
##    .foodst_08_T3      1.822    0.040   45.006    0.000
## 
## 
## Level 2 [country]:
## 
## Intercepts:
##                    Estimate  Std.Err  z-value  P(>|z|)
##    .hds_T3           14.320    1.978    7.239    0.000
## 
## Variances:
##                    Estimate  Std.Err  z-value  P(>|z|)
##    .hds_T3            6.259    3.213    1.948    0.051
## 
## Defined Parameters:
##                    Estimate  Std.Err  z-value  P(>|z|)
##     ebndfdst_01_T3    0.001    0.058    0.024    0.981
##     ebndfdst_02_T3   -0.064    0.041   -1.559    0.119
##     ebndfdst_03_T3    0.006    0.021    0.306    0.759
##     ebndfdst_04_T3    0.094    0.047    1.988    0.047
##     ebndfdst_05_T3   -0.009    0.015   -0.604    0.546
##     ebndfdst_06_T3    0.083    0.037    2.228    0.026
##     ebndfdst_07_T3    0.005    0.013    0.389    0.697
##     ebndfdst_08_T3   -0.004    0.025   -0.156    0.876
```

```
parameterEstimates(fit_SEM_une, ci=TRUE, level=.99375)
```

```
##                  lhs op          rhs block level             label    est    se
## 1             hds_T3  ~     unemploy     1     1                   -1.408 0.454
## 2             hds_T3  ~ foodst_01_T3     1     1                a1  0.819 0.111
## 3             hds_T3  ~ foodst_02_T3     1     1                a2 -0.649 0.118
## 4             hds_T3  ~ foodst_03_T3     1     1                a3  0.338 0.144
## 5             hds_T3  ~ foodst_04_T3     1     1                a4  0.619 0.130
## 6             hds_T3  ~ foodst_05_T3     1     1                a5 -0.096 0.150
## 7             hds_T3  ~ foodst_06_T3     1     1                a6 -0.514 0.135
## 8             hds_T3  ~ foodst_07_T3     1     1                a7 -0.064 0.160
## 9             hds_T3  ~ foodst_08_T3     1     1                a8  0.331 0.096
## 10            hds_T3  ~       age_T3     1     1                    0.107 0.023
## 11            hds_T3  ~       sex_T3     1     1                    1.469 0.375
## 12            hds_T3  ~       bmi_T3     1     1                    0.052 0.025
## 13      foodst_01_T3  ~     unemploy     1     1                b1  0.002 0.071
## 14      foodst_02_T3  ~     unemploy     1     1                b2  0.099 0.061
## 15      foodst_03_T3  ~     unemploy     1     1                b3  0.019 0.062
## 16      foodst_04_T3  ~     unemploy     1     1                b4  0.151 0.069
## 17      foodst_05_T3  ~     unemploy     1     1                b5  0.097 0.053
## 18      foodst_06_T3  ~     unemploy     1     1                b6 -0.162 0.059
## 19      foodst_07_T3  ~     unemploy     1     1                b7 -0.080 0.049
## 20      foodst_08_T3  ~     unemploy     1     1                b8 -0.012 0.075
## 21      foodst_01_T3 ~~ foodst_02_T3     1     1                    0.186 0.022
## 22      foodst_01_T3 ~~ foodst_03_T3     1     1                    0.578 0.024
## 23      foodst_01_T3 ~~ foodst_04_T3     1     1                    0.571 0.027
## 24      foodst_01_T3 ~~ foodst_05_T3     1     1                    0.190 0.020
## 25      foodst_01_T3 ~~ foodst_06_T3     1     1                   -0.102 0.022
## 26      foodst_01_T3 ~~ foodst_07_T3     1     1                   -0.090 0.018
## 27      foodst_01_T3 ~~ foodst_08_T3     1     1                    0.146 0.027
## 28      foodst_02_T3 ~~ foodst_03_T3     1     1                    0.061 0.019
## 29      foodst_02_T3 ~~ foodst_04_T3     1     1                    0.102 0.022
## 30      foodst_02_T3 ~~ foodst_05_T3     1     1                    0.048 0.017
## 31      foodst_02_T3 ~~ foodst_06_T3     1     1                    0.060 0.018
## 32      foodst_02_T3 ~~ foodst_07_T3     1     1                    0.066 0.015
## 33      foodst_02_T3 ~~ foodst_08_T3     1     1                   -0.039 0.023
## 34      foodst_03_T3 ~~ foodst_04_T3     1     1                    0.756 0.025
## 35      foodst_03_T3 ~~ foodst_05_T3     1     1                    0.356 0.018
## 36      foodst_03_T3 ~~ foodst_06_T3     1     1                   -0.231 0.019
## 37      foodst_03_T3 ~~ foodst_07_T3     1     1                   -0.186 0.016
## 38      foodst_03_T3 ~~ foodst_08_T3     1     1                    0.174 0.024
## 39      foodst_04_T3 ~~ foodst_05_T3     1     1                    0.482 0.020
## 40      foodst_04_T3 ~~ foodst_06_T3     1     1                   -0.226 0.021
## 41      foodst_04_T3 ~~ foodst_07_T3     1     1                   -0.190 0.018
## 42      foodst_04_T3 ~~ foodst_08_T3     1     1                    0.206 0.027
## 43      foodst_05_T3 ~~ foodst_06_T3     1     1                   -0.226 0.016
## 44      foodst_05_T3 ~~ foodst_07_T3     1     1                   -0.170 0.014
## 45      foodst_05_T3 ~~ foodst_08_T3     1     1                    0.166 0.020
## 46      foodst_06_T3 ~~ foodst_07_T3     1     1                    0.395 0.016
## 47      foodst_06_T3 ~~ foodst_08_T3     1     1                   -0.059 0.023
## 48      foodst_07_T3 ~~ foodst_08_T3     1     1                   -0.035 0.019
## 49            hds_T3 ~~       hds_T3     1     1                   66.236 1.473
## 50      foodst_01_T3 ~~ foodst_01_T3     1     1                    1.649 0.037
## 51      foodst_02_T3 ~~ foodst_02_T3     1     1                    1.213 0.027
## 52      foodst_03_T3 ~~ foodst_03_T3     1     1                    1.251 0.028
## 53      foodst_04_T3 ~~ foodst_04_T3     1     1                    1.556 0.035
## 54      foodst_05_T3 ~~ foodst_05_T3     1     1                    0.919 0.020
## 55      foodst_06_T3 ~~ foodst_06_T3     1     1                    1.134 0.025
## 56      foodst_07_T3 ~~ foodst_07_T3     1     1                    0.796 0.018
## 57      foodst_08_T3 ~~ foodst_08_T3     1     1                    1.822 0.040
## 58          unemploy ~~     unemploy     1     1                    0.080 0.000
## 59          unemploy ~~       age_T3     1     1                   -0.023 0.000
## 60          unemploy ~~       sex_T3     1     1                    0.002 0.000
## 61          unemploy ~~       bmi_T3     1     1                    0.120 0.000
## 62            age_T3 ~~       age_T3     1     1                   31.686 0.000
## 63            age_T3 ~~       sex_T3     1     1                   -0.456 0.000
## 64            age_T3 ~~       bmi_T3     1     1                    2.483 0.000
## 65            sex_T3 ~~       sex_T3     1     1                    0.126 0.000
## 66            sex_T3 ~~       bmi_T3     1     1                   -0.249 0.000
## 67            bmi_T3 ~~       bmi_T3     1     1                   27.722 0.000
## 68            hds_T3 ~1                  1     1                    0.000 0.000
## 69      foodst_01_T3 ~1                  1     1                    3.447 0.080
## 70      foodst_02_T3 ~1                  1     1                    1.793 0.069
## 71      foodst_03_T3 ~1                  1     1                    3.948 0.070
## 72      foodst_04_T3 ~1                  1     1                    3.392 0.078
## 73      foodst_05_T3 ~1                  1     1                    4.268 0.060
## 74      foodst_06_T3 ~1                  1     1                    2.097 0.066
## 75      foodst_07_T3 ~1                  1     1                    1.649 0.056
## 76      foodst_08_T3 ~1                  1     1                    3.379 0.084
## 77          unemploy ~1                  1     1                    1.088 0.000
## 78            age_T3 ~1                  1     1                   41.916 0.000
## 79            sex_T3 ~1                  1     1                    1.852 0.000
## 80            bmi_T3 ~1                  1     1                   26.058 0.000
## 81            hds_T3 ~1                  2     2                   14.320 1.978
## 82            hds_T3 ~~       hds_T3     2     2                    6.259 3.213
## 83 ebindfoodst_01_T3 :=        a1*b1     0     0 ebindfoodst_01_T3  0.001 0.058
## 84 ebindfoodst_02_T3 :=        a2*b2     0     0 ebindfoodst_02_T3 -0.064 0.041
## 85 ebindfoodst_03_T3 :=        a3*b3     0     0 ebindfoodst_03_T3  0.006 0.021
## 86 ebindfoodst_04_T3 :=        a4*b4     0     0 ebindfoodst_04_T3  0.094 0.047
## 87 ebindfoodst_05_T3 :=        a5*b5     0     0 ebindfoodst_05_T3 -0.009 0.015
## 88 ebindfoodst_06_T3 :=        a6*b6     0     0 ebindfoodst_06_T3  0.083 0.037
## 89 ebindfoodst_07_T3 :=        a7*b7     0     0 ebindfoodst_07_T3  0.005 0.013
## 90 ebindfoodst_08_T3 :=        a8*b8     0     0 ebindfoodst_08_T3 -0.004 0.025
##          z pvalue ci.lower ci.upper
## 1   -3.104  0.002   -2.649   -0.168
## 2    7.349  0.000    0.514    1.124
## 3   -5.496  0.000   -0.972   -0.326
## 4    2.344  0.019   -0.056    0.732
## 5    4.768  0.000    0.264    0.974
## 6   -0.640  0.522   -0.507    0.315
## 7   -3.819  0.000   -0.882   -0.146
## 8   -0.401  0.688   -0.502    0.373
## 9    3.437  0.001    0.068    0.595
## 10   4.577  0.000    0.043    0.172
## 11   3.919  0.000    0.444    2.494
## 12   2.084  0.037   -0.016    0.119
## 13   0.024  0.981   -0.193    0.196
## 14   1.626  0.104   -0.068    0.266
## 15   0.309  0.757   -0.150    0.189
## 16   2.187  0.029   -0.038    0.340
## 17   1.829  0.067   -0.048    0.242
## 18  -2.743  0.006   -0.323   -0.001
## 19  -1.610  0.107   -0.215    0.056
## 20  -0.156  0.876   -0.216    0.193
## 21   8.296  0.000    0.125    0.247
## 22  23.760  0.000    0.511    0.644
## 23  21.361  0.000    0.498    0.644
## 24   9.691  0.000    0.136    0.243
## 25  -4.730  0.000   -0.161   -0.043
## 26  -4.967  0.000   -0.139   -0.040
## 27   5.342  0.000    0.071    0.221
## 28   3.156  0.002    0.008    0.114
## 29   4.713  0.000    0.043    0.161
## 30   2.893  0.004    0.003    0.093
## 31   3.271  0.001    0.010    0.111
## 32   4.264  0.000    0.024    0.108
## 33  -1.667  0.096   -0.103    0.025
## 34  30.331  0.000    0.688    0.824
## 35  20.072  0.000    0.308    0.405
## 36 -12.126  0.000   -0.283   -0.179
## 37 -11.649  0.000   -0.229   -0.142
## 38   7.300  0.000    0.109    0.240
## 39  23.776  0.000    0.426    0.537
## 40 -10.673  0.000   -0.284   -0.168
## 41 -10.731  0.000   -0.239   -0.142
## 42   7.738  0.000    0.133    0.279
## 43 -13.779  0.000   -0.271   -0.181
## 44 -12.379  0.000   -0.207   -0.132
## 45   8.118  0.000    0.110    0.222
## 46  24.427  0.000    0.351    0.439
## 47  -2.589  0.010   -0.120    0.003
## 48  -1.846  0.065   -0.087    0.017
## 49  44.962  0.000   62.207   70.264
## 50  45.006  0.000    1.548    1.749
## 51  45.006  0.000    1.139    1.287
## 52  45.005  0.000    1.175    1.326
## 53  45.005  0.000    1.462    1.651
## 54  45.006  0.000    0.863    0.975
## 55  45.005  0.000    1.065    1.203
## 56  45.006  0.000    0.748    0.844
## 57  45.006  0.000    1.711    1.933
## 58      NA     NA    0.080    0.080
## 59      NA     NA   -0.023   -0.023
## 60      NA     NA    0.002    0.002
## 61      NA     NA    0.120    0.120
## 62      NA     NA   31.686   31.686
## 63      NA     NA   -0.456   -0.456
## 64      NA     NA    2.483    2.483
## 65      NA     NA    0.126    0.126
## 66      NA     NA   -0.249   -0.249
## 67      NA     NA   27.722   27.722
## 68      NA     NA    0.000    0.000
## 69  43.082  0.000    3.229    3.666
## 70  26.122  0.000    1.605    1.981
## 71  56.643  0.000    3.757    4.138
## 72  43.631  0.000    3.180    3.605
## 73  71.434  0.000    4.104    4.431
## 74  31.599  0.000    1.915    2.278
## 75  29.664  0.000    1.497    1.801
## 76  40.169  0.000    3.149    3.609
## 77      NA     NA    1.088    1.088
## 78      NA     NA   41.916   41.916
## 79      NA     NA    1.852    1.852
## 80      NA     NA   26.058   26.058
## 81   7.239  0.000    8.911   19.728
## 82   1.948  0.051   -2.526   15.044
## 83   0.024  0.981   -0.158    0.161
## 84  -1.559  0.119   -0.177    0.049
## 85   0.306  0.759   -0.051    0.064
## 86   1.988  0.047   -0.035    0.222
## 87  -0.604  0.546   -0.052    0.033
## 88   2.228  0.026   -0.019    0.185
## 89   0.389  0.697   -0.031    0.041
## 90  -0.156  0.876   -0.072    0.064
```

### 4.2.5 Single parenthood

Controlled for age, sex, BMI

```
SEM_model_sin <- '
    level: 1
    hds_T3 ~ singlepar + a1*foodst_01_T3 + a2*foodst_02_T3 + a3*foodst_03_T3 + a4*foodst_04_T3 + a5*foodst_05_T3 + a6*foodst_06_T3 + a7*foodst_07_T3 + a8*foodst_08_T3 + age_T3 + sex_T3 + bmi_T3
    foodst_01_T3 ~ b1*singlepar
    foodst_02_T3 ~ b2*singlepar
    foodst_03_T3 ~ b3*singlepar
    foodst_04_T3 ~ b4*singlepar
    foodst_05_T3 ~ b5*singlepar
    foodst_06_T3 ~ b6*singlepar
    foodst_07_T3 ~ b7*singlepar
    foodst_08_T3 ~ b8*singlepar
    foodst_01_T3 ~~ foodst_02_T3 + foodst_03_T3 + foodst_04_T3 + foodst_05_T3 + foodst_06_T3 + foodst_07_T3 + foodst_08_T3
    foodst_02_T3 ~~ foodst_03_T3 + foodst_04_T3 + foodst_05_T3 + foodst_06_T3 + foodst_07_T3 + foodst_08_T3
    foodst_03_T3 ~~ foodst_04_T3 + foodst_05_T3 + foodst_06_T3 + foodst_07_T3 + foodst_08_T3
    foodst_04_T3 ~~ foodst_05_T3 + foodst_06_T3 + foodst_07_T3 + foodst_08_T3
    foodst_05_T3 ~~ foodst_06_T3 + foodst_07_T3 + foodst_08_T3
    foodst_06_T3 ~~ foodst_07_T3 + foodst_08_T3 
    foodst_07_T3 ~~ foodst_08_T3
    ebindfoodst_01_T3 := a1*b1 
    ebindfoodst_02_T3 := a2*b2
    ebindfoodst_03_T3 := a3*b3
    ebindfoodst_04_T3 := a4*b4
    ebindfoodst_05_T3 := a5*b5
    ebindfoodst_06_T3 := a6*b6
    ebindfoodst_07_T3 := a7*b7
    ebindfoodst_08_T3 := a8*b8

    level: 2
    hds_T3 ~ 1

'

fit_SEM_sin <- sem(model = SEM_model_sin, data = subset, cluster = "country")

summary(fit_SEM_sin)
```

```
## lavaan 0.6.16 ended normally after 111 iterations
## 
##   Estimator                                         ML
##   Optimization method                           NLMINB
##   Number of model parameters                        67
## 
##                                                   Used       Total
##   Number of observations                          4035        4051
##   Number of clusters [country]                       8            
## 
## Model Test User Model:
##                                                       
##   Test statistic                               105.711
##   Degrees of freedom                                24
##   P-value (Chi-square)                           0.000
## 
## Parameter Estimates:
## 
##   Standard errors                             Standard
##   Information                                 Observed
##   Observed information based on                Hessian
## 
## 
## Level 1 [within]:
## 
## Regressions:
##                    Estimate  Std.Err  z-value  P(>|z|)
##   hds_T3 ~                                            
##     singlepar         0.291    0.427    0.680    0.496
##     fds_01_T3 (a1)    0.826    0.112    7.385    0.000
##     fds_02_T3 (a2)   -0.650    0.118   -5.500    0.000
##     fds_03_T3 (a3)    0.337    0.145    2.333    0.020
##     fds_04_T3 (a4)    0.610    0.130    4.690    0.000
##     fds_05_T3 (a5)   -0.071    0.151   -0.472    0.637
##     fds_06_T3 (a6)   -0.516    0.135   -3.819    0.000
##     fds_07_T3 (a7)   -0.047    0.161   -0.294    0.769
##     fds_08_T3 (a8)    0.330    0.096    3.421    0.001
##     age_T3            0.109    0.024    4.618    0.000
##     sex_T3            1.415    0.376    3.762    0.000
##     bmi_T3            0.046    0.025    1.850    0.064
##   foodst_01_T3 ~                                      
##     singlepar (b1)   -0.271    0.067   -4.076    0.000
##   foodst_02_T3 ~                                      
##     singlepar (b2)   -0.099    0.057   -1.724    0.085
##   foodst_03_T3 ~                                      
##     singlepar (b3)   -0.236    0.058   -4.069    0.000
##   foodst_04_T3 ~                                      
##     singlepar (b4)   -0.324    0.065   -4.997    0.000
##   foodst_05_T3 ~                                      
##     singlepar (b5)   -0.199    0.050   -3.993    0.000
##   foodst_06_T3 ~                                      
##     singlepar (b6)    0.252    0.055    4.559    0.000
##   foodst_07_T3 ~                                      
##     singlepar (b7)    0.104    0.046    2.250    0.024
##   foodst_08_T3 ~                                      
##     singlepar (b8)    0.107    0.070    1.521    0.128
## 
## Covariances:
##                    Estimate  Std.Err  z-value  P(>|z|)
##  .foodst_01_T3 ~~                                     
##    .foodst_02_T3      0.184    0.022    8.229    0.000
##    .foodst_03_T3      0.573    0.024   23.634    0.000
##    .foodst_04_T3      0.564    0.027   21.181    0.000
##    .foodst_05_T3      0.185    0.020    9.495    0.000
##    .foodst_06_T3     -0.099    0.021   -4.587    0.000
##    .foodst_07_T3     -0.087    0.018   -4.846    0.000
##    .foodst_08_T3      0.148    0.027    5.425    0.000
##  .foodst_02_T3 ~~                                     
##    .foodst_03_T3      0.059    0.019    3.045    0.002
##    .foodst_04_T3      0.101    0.022    4.642    0.000
##    .foodst_05_T3      0.047    0.017    2.835    0.005
##    .foodst_06_T3      0.061    0.018    3.279    0.001
##    .foodst_07_T3      0.065    0.015    4.231    0.000
##    .foodst_08_T3     -0.038    0.023   -1.621    0.105
##  .foodst_03_T3 ~~                                     
##    .foodst_04_T3      0.751    0.025   30.193    0.000
##    .foodst_05_T3      0.353    0.018   19.931    0.000
##    .foodst_06_T3     -0.227    0.019  -11.962    0.000
##    .foodst_07_T3     -0.183    0.016  -11.511    0.000
##    .foodst_08_T3      0.177    0.024    7.413    0.000
##  .foodst_04_T3 ~~                                     
##    .foodst_05_T3      0.478    0.020   23.634    0.000
##    .foodst_06_T3     -0.222    0.021  -10.530    0.000
##    .foodst_07_T3     -0.191    0.018  -10.765    0.000
##    .foodst_08_T3      0.210    0.027    7.850    0.000
##  .foodst_05_T3 ~~                                     
##    .foodst_06_T3     -0.223    0.016  -13.609    0.000
##    .foodst_07_T3     -0.169    0.014  -12.346    0.000
##    .foodst_08_T3      0.169    0.021    8.211    0.000
##  .foodst_06_T3 ~~                                     
##    .foodst_07_T3      0.391    0.016   24.282    0.000
##    .foodst_08_T3     -0.064    0.023   -2.808    0.005
##  .foodst_07_T3 ~~                                     
##    .foodst_08_T3     -0.038    0.019   -2.019    0.043
## 
## Intercepts:
##                    Estimate  Std.Err  z-value  P(>|z|)
##    .hds_T3            0.000                           
##    .foodst_01_T3      3.749    0.076   49.266    0.000
##    .foodst_02_T3      2.011    0.066   30.689    0.000
##    .foodst_03_T3      4.229    0.066   63.788    0.000
##    .foodst_04_T3      3.912    0.074   52.851    0.000
##    .foodst_05_T3      4.592    0.057   80.687    0.000
##    .foodst_06_T3      1.642    0.063   26.011    0.000
##    .foodst_07_T3      1.447    0.053   27.366    0.000
##    .foodst_08_T3      3.248    0.080   40.456    0.000
## 
## Variances:
##                    Estimate  Std.Err  z-value  P(>|z|)
##    .hds_T3           66.216    1.476   44.872    0.000
##    .foodst_01_T3      1.640    0.037   44.917    0.000
##    .foodst_02_T3      1.216    0.027   44.917    0.000
##    .foodst_03_T3      1.245    0.028   44.917    0.000
##    .foodst_04_T3      1.551    0.035   44.916    0.000
##    .foodst_05_T3      0.917    0.020   44.917    0.000
##    .foodst_06_T3      1.128    0.025   44.916    0.000
##    .foodst_07_T3      0.792    0.018   44.917    0.000
##    .foodst_08_T3      1.825    0.041   44.917    0.000
## 
## 
## Level 2 [country]:
## 
## Intercepts:
##                    Estimate  Std.Err  z-value  P(>|z|)
##    .hds_T3           12.545    1.978    6.344    0.000
## 
## Variances:
##                    Estimate  Std.Err  z-value  P(>|z|)
##    .hds_T3            6.197    3.179    1.950    0.051
## 
## Defined Parameters:
##                    Estimate  Std.Err  z-value  P(>|z|)
##     ebndfdst_01_T3   -0.224    0.063   -3.569    0.000
##     ebndfdst_02_T3    0.064    0.039    1.645    0.100
##     ebndfdst_03_T3   -0.080    0.039   -2.024    0.043
##     ebndfdst_04_T3   -0.197    0.058   -3.420    0.001
##     ebndfdst_05_T3    0.014    0.030    0.469    0.639
##     ebndfdst_06_T3   -0.130    0.044   -2.927    0.003
##     ebndfdst_07_T3   -0.005    0.017   -0.292    0.771
##     ebndfdst_08_T3    0.035    0.025    1.390    0.165
```

```
parameterEstimates(fit_SEM_sin, ci=TRUE, level=.99375)
```

```
##                  lhs op          rhs block level             label    est    se
## 1             hds_T3  ~    singlepar     1     1                    0.291 0.427
## 2             hds_T3  ~ foodst_01_T3     1     1                a1  0.826 0.112
## 3             hds_T3  ~ foodst_02_T3     1     1                a2 -0.650 0.118
## 4             hds_T3  ~ foodst_03_T3     1     1                a3  0.337 0.145
## 5             hds_T3  ~ foodst_04_T3     1     1                a4  0.610 0.130
## 6             hds_T3  ~ foodst_05_T3     1     1                a5 -0.071 0.151
## 7             hds_T3  ~ foodst_06_T3     1     1                a6 -0.516 0.135
## 8             hds_T3  ~ foodst_07_T3     1     1                a7 -0.047 0.161
## 9             hds_T3  ~ foodst_08_T3     1     1                a8  0.330 0.096
## 10            hds_T3  ~       age_T3     1     1                    0.109 0.024
## 11            hds_T3  ~       sex_T3     1     1                    1.415 0.376
## 12            hds_T3  ~       bmi_T3     1     1                    0.046 0.025
## 13      foodst_01_T3  ~    singlepar     1     1                b1 -0.271 0.067
## 14      foodst_02_T3  ~    singlepar     1     1                b2 -0.099 0.057
## 15      foodst_03_T3  ~    singlepar     1     1                b3 -0.236 0.058
## 16      foodst_04_T3  ~    singlepar     1     1                b4 -0.324 0.065
## 17      foodst_05_T3  ~    singlepar     1     1                b5 -0.199 0.050
## 18      foodst_06_T3  ~    singlepar     1     1                b6  0.252 0.055
## 19      foodst_07_T3  ~    singlepar     1     1                b7  0.104 0.046
## 20      foodst_08_T3  ~    singlepar     1     1                b8  0.107 0.070
## 21      foodst_01_T3 ~~ foodst_02_T3     1     1                    0.184 0.022
## 22      foodst_01_T3 ~~ foodst_03_T3     1     1                    0.573 0.024
## 23      foodst_01_T3 ~~ foodst_04_T3     1     1                    0.564 0.027
## 24      foodst_01_T3 ~~ foodst_05_T3     1     1                    0.185 0.020
## 25      foodst_01_T3 ~~ foodst_06_T3     1     1                   -0.099 0.021
## 26      foodst_01_T3 ~~ foodst_07_T3     1     1                   -0.087 0.018
## 27      foodst_01_T3 ~~ foodst_08_T3     1     1                    0.148 0.027
## 28      foodst_02_T3 ~~ foodst_03_T3     1     1                    0.059 0.019
## 29      foodst_02_T3 ~~ foodst_04_T3     1     1                    0.101 0.022
## 30      foodst_02_T3 ~~ foodst_05_T3     1     1                    0.047 0.017
## 31      foodst_02_T3 ~~ foodst_06_T3     1     1                    0.061 0.018
## 32      foodst_02_T3 ~~ foodst_07_T3     1     1                    0.065 0.015
## 33      foodst_02_T3 ~~ foodst_08_T3     1     1                   -0.038 0.023
## 34      foodst_03_T3 ~~ foodst_04_T3     1     1                    0.751 0.025
## 35      foodst_03_T3 ~~ foodst_05_T3     1     1                    0.353 0.018
## 36      foodst_03_T3 ~~ foodst_06_T3     1     1                   -0.227 0.019
## 37      foodst_03_T3 ~~ foodst_07_T3     1     1                   -0.183 0.016
## 38      foodst_03_T3 ~~ foodst_08_T3     1     1                    0.177 0.024
## 39      foodst_04_T3 ~~ foodst_05_T3     1     1                    0.478 0.020
## 40      foodst_04_T3 ~~ foodst_06_T3     1     1                   -0.222 0.021
## 41      foodst_04_T3 ~~ foodst_07_T3     1     1                   -0.191 0.018
## 42      foodst_04_T3 ~~ foodst_08_T3     1     1                    0.210 0.027
## 43      foodst_05_T3 ~~ foodst_06_T3     1     1                   -0.223 0.016
## 44      foodst_05_T3 ~~ foodst_07_T3     1     1                   -0.169 0.014
## 45      foodst_05_T3 ~~ foodst_08_T3     1     1                    0.169 0.021
## 46      foodst_06_T3 ~~ foodst_07_T3     1     1                    0.391 0.016
## 47      foodst_06_T3 ~~ foodst_08_T3     1     1                   -0.064 0.023
## 48      foodst_07_T3 ~~ foodst_08_T3     1     1                   -0.038 0.019
## 49            hds_T3 ~~       hds_T3     1     1                   66.216 1.476
## 50      foodst_01_T3 ~~ foodst_01_T3     1     1                    1.640 0.037
## 51      foodst_02_T3 ~~ foodst_02_T3     1     1                    1.216 0.027
## 52      foodst_03_T3 ~~ foodst_03_T3     1     1                    1.245 0.028
## 53      foodst_04_T3 ~~ foodst_04_T3     1     1                    1.551 0.035
## 54      foodst_05_T3 ~~ foodst_05_T3     1     1                    0.917 0.020
## 55      foodst_06_T3 ~~ foodst_06_T3     1     1                    1.128 0.025
## 56      foodst_07_T3 ~~ foodst_07_T3     1     1                    0.792 0.018
## 57      foodst_08_T3 ~~ foodst_08_T3     1     1                    1.825 0.041
## 58         singlepar ~~    singlepar     1     1                    0.092 0.000
## 59         singlepar ~~       age_T3     1     1                    0.015 0.000
## 60         singlepar ~~       sex_T3     1     1                    0.007 0.000
## 61         singlepar ~~       bmi_T3     1     1                   -0.060 0.000
## 62            age_T3 ~~       age_T3     1     1                   31.762 0.000
## 63            age_T3 ~~       sex_T3     1     1                   -0.458 0.000
## 64            age_T3 ~~       bmi_T3     1     1                    2.509 0.000
## 65            sex_T3 ~~       sex_T3     1     1                    0.126 0.000
## 66            sex_T3 ~~       bmi_T3     1     1                   -0.249 0.000
## 67            bmi_T3 ~~       bmi_T3     1     1                   27.754 0.000
## 68            hds_T3 ~1                  1     1                    0.000 0.000
## 69      foodst_01_T3 ~1                  1     1                    3.749 0.076
## 70      foodst_02_T3 ~1                  1     1                    2.011 0.066
## 71      foodst_03_T3 ~1                  1     1                    4.229 0.066
## 72      foodst_04_T3 ~1                  1     1                    3.912 0.074
## 73      foodst_05_T3 ~1                  1     1                    4.592 0.057
## 74      foodst_06_T3 ~1                  1     1                    1.642 0.063
## 75      foodst_07_T3 ~1                  1     1                    1.447 0.053
## 76      foodst_08_T3 ~1                  1     1                    3.248 0.080
## 77         singlepar ~1                  1     1                    1.102 0.000
## 78            age_T3 ~1                  1     1                   41.905 0.000
## 79            sex_T3 ~1                  1     1                    1.852 0.000
## 80            bmi_T3 ~1                  1     1                   26.068 0.000
## 81            hds_T3 ~1                  2     2                   12.545 1.978
## 82            hds_T3 ~~       hds_T3     2     2                    6.197 3.179
## 83 ebindfoodst_01_T3 :=        a1*b1     0     0 ebindfoodst_01_T3 -0.224 0.063
## 84 ebindfoodst_02_T3 :=        a2*b2     0     0 ebindfoodst_02_T3  0.064 0.039
## 85 ebindfoodst_03_T3 :=        a3*b3     0     0 ebindfoodst_03_T3 -0.080 0.039
## 86 ebindfoodst_04_T3 :=        a4*b4     0     0 ebindfoodst_04_T3 -0.197 0.058
## 87 ebindfoodst_05_T3 :=        a5*b5     0     0 ebindfoodst_05_T3  0.014 0.030
## 88 ebindfoodst_06_T3 :=        a6*b6     0     0 ebindfoodst_06_T3 -0.130 0.044
## 89 ebindfoodst_07_T3 :=        a7*b7     0     0 ebindfoodst_07_T3 -0.005 0.017
## 90 ebindfoodst_08_T3 :=        a8*b8     0     0 ebindfoodst_08_T3  0.035 0.025
##          z pvalue ci.lower ci.upper
## 1    0.680  0.496   -0.878    1.460
## 2    7.385  0.000    0.520    1.132
## 3   -5.500  0.000   -0.973   -0.327
## 4    2.333  0.020   -0.058    0.733
## 5    4.690  0.000    0.254    0.966
## 6   -0.472  0.637   -0.483    0.341
## 7   -3.819  0.000   -0.885   -0.146
## 8   -0.294  0.769   -0.486    0.392
## 9    3.421  0.001    0.066    0.594
## 10   4.618  0.000    0.044    0.173
## 11   3.762  0.000    0.387    2.444
## 12   1.850  0.064   -0.022    0.113
## 13  -4.076  0.000   -0.453   -0.089
## 14  -1.724  0.085   -0.256    0.058
## 15  -4.069  0.000   -0.395   -0.077
## 16  -4.997  0.000   -0.501   -0.147
## 17  -3.993  0.000   -0.335   -0.063
## 18   4.559  0.000    0.101    0.403
## 19   2.250  0.024   -0.022    0.231
## 20   1.521  0.128   -0.085    0.299
## 21   8.229  0.000    0.123    0.246
## 22  23.634  0.000    0.506    0.639
## 23  21.181  0.000    0.491    0.637
## 24   9.495  0.000    0.132    0.239
## 25  -4.587  0.000   -0.157   -0.040
## 26  -4.846  0.000   -0.136   -0.038
## 27   5.425  0.000    0.074    0.223
## 28   3.045  0.002    0.006    0.112
## 29   4.642  0.000    0.041    0.160
## 30   2.835  0.005    0.002    0.093
## 31   3.279  0.001    0.010    0.111
## 32   4.231  0.000    0.023    0.108
## 33  -1.621  0.105   -0.102    0.026
## 34  30.193  0.000    0.683    0.819
## 35  19.931  0.000    0.305    0.402
## 36 -11.962  0.000   -0.279   -0.175
## 37 -11.511  0.000   -0.226   -0.139
## 38   7.413  0.000    0.112    0.242
## 39  23.634  0.000    0.423    0.533
## 40 -10.530  0.000   -0.280   -0.165
## 41 -10.765  0.000   -0.239   -0.142
## 42   7.850  0.000    0.137    0.283
## 43 -13.609  0.000   -0.268   -0.178
## 44 -12.346  0.000   -0.206   -0.131
## 45   8.211  0.000    0.112    0.225
## 46  24.282  0.000    0.347    0.435
## 47  -2.808  0.005   -0.125   -0.002
## 48  -2.019  0.043   -0.090    0.014
## 49  44.872  0.000   62.181   70.251
## 50  44.917  0.000    1.540    1.740
## 51  44.917  0.000    1.142    1.290
## 52  44.917  0.000    1.169    1.321
## 53  44.916  0.000    1.457    1.646
## 54  44.917  0.000    0.861    0.973
## 55  44.916  0.000    1.060    1.197
## 56  44.917  0.000    0.744    0.840
## 57  44.917  0.000    1.714    1.936
## 58      NA     NA    0.092    0.092
## 59      NA     NA    0.015    0.015
## 60      NA     NA    0.007    0.007
## 61      NA     NA   -0.060   -0.060
## 62      NA     NA   31.762   31.762
## 63      NA     NA   -0.458   -0.458
## 64      NA     NA    2.509    2.509
## 65      NA     NA    0.126    0.126
## 66      NA     NA   -0.249   -0.249
## 67      NA     NA   27.754   27.754
## 68      NA     NA    0.000    0.000
## 69  49.266  0.000    3.541    3.957
## 70  30.689  0.000    1.832    2.190
## 71  63.788  0.000    4.048    4.411
## 72  52.851  0.000    3.709    4.114
## 73  80.687  0.000    4.436    4.747
## 74  26.011  0.000    1.469    1.815
## 75  27.366  0.000    1.303    1.592
## 76  40.456  0.000    3.028    3.467
## 77      NA     NA    1.102    1.102
## 78      NA     NA   41.905   41.905
## 79      NA     NA    1.852    1.852
## 80      NA     NA   26.068   26.068
## 81   6.344  0.000    7.137   17.952
## 82   1.950  0.051   -2.495   14.889
## 83  -3.569  0.000   -0.396   -0.052
## 84   1.645  0.100   -0.043    0.171
## 85  -2.024  0.043   -0.187    0.028
## 86  -3.420  0.001   -0.355   -0.040
## 87   0.469  0.639   -0.068    0.097
## 88  -2.927  0.003   -0.251   -0.009
## 89  -0.292  0.771   -0.051    0.041
## 90   1.390  0.165   -0.034    0.105
```
