# Supplementary material for: Consumer attitudes towards dietary behaviors: a mediator between socioeconomic status and diet quality in European adults
Source: Eur J Nutr. 2025 Mar 19;64(3):127. doi: 10.1007/s00394-025-03645-6 (PMC11922978; doi:10.1007/s00394-025-03645-6)
Supplement: Supplementary file 3 — Supplementary file3 (HTML 1047 KB) [file 394_2025_3645_MOESM3_ESM.html]

Consumer attitudes towards dietary behaviors: a mediator between socioeconomic status and diet quality in European adults


# Consumer attitudes towards dietary behaviors: a mediator between socioeconomic status and diet quality in European adults

### Online supplement - Sensitivity analysis

- Study
  description
  - Description of variables
- 0. Packages, sources,
  directories
- 1.
  Load and prepare data
- 2. Table 1: Study sample
  characteristics
- 3. Table
  2: Responses to consumer attitudes questionnaire
- 4. Analysis
  - 4.1 Path
    analysis
    - 4.1.2 Path a:
      Socioeconomic factors -> Consumer attitudes
    - 4.1.3 Path b: Consumer
      attitudes -> HDAS
    - 5.1.4 Path c:
      Socioeconomic factors -> HDAS (Total effect)
  - 4.2
    Structural equation modelling using lavaan package (indirect and direct
    effect)
    - 4.2.1 Highest level of
      education in household
    - 4.2.2
      Household income
    - 4.2.3
      Migration background
    - 4.2.4 Unemployment in
      household
    - 4.2.5 Single-parent household

**Here we present the syntax for the sensitivity analysis
excluding participants from Belgium and Spain. In these countries, the
answer categories for the questionnaire asking about consumer attitudes
were reversed.**

# Study description

Based on the I.Family survey and only using data of parents, we aimed
to investigate whether consumer attitudes as assessed in the I.Family
cohort serve as mediators in the relationship between socioeconomic
factors and diet quality as indicated by a healthy eating score.

## Description of variables

**family\_id**: family ID number  
**ID\_cohort** / **ID\_no**: Matched ID of
IDEFICS and I. family / ID-Number (I.Family ID)  
**country**: 1 Italy 2 Estonia 3 Cyprus 4 Belgium 6 Sweden
7 Germany 8 Hungary 9 Spain  
**sex\_T3**: Sex of participant (1 male, 2 female)  
**age\_status\_T3**: Age status of participant (1 child, 2
teen, 3 adult)  
**age\_T3**: Age of participant (years)  
**bmi\_T3**: Body Mass Index of participant
(kg/m2)  
**isced\_cat2011\_T3**: ISCED level maximum of both parents
(1 low, 2 medium, 3 high)  
**income\_cat\_T3**: Income categories (1 low, 2 low/medium,
3 medium, 4 medium/high, 5 high)  
**migration\_T3**: Migration status of parents (0 both
parents non-, 1 one parent migrant, 2 both parents migrant)  
**occupst\_1\_T3**: Employment status of parent (1
full-time/30hrs, 2 part-time/15-29hrs, 3 part-time/<15hrs, 4
temporary company leave, 5 apprentice/retrainee, 6 currently
unemployed)  
**occupst\_2\_T3**: Employment status of partner of parent
(…)  
**no\_occupst\_1**: Employment status of parent if working
part-time/not employed (1 attend school, 2 attend university, 3
homemaker, 4 retired, 5 unemployed less than 1 year, 6 unemployed for
more than 1 year, 7 on welfare, 8 doing military, 9 others)  
**no\_occupst\_2**: Employment status of partner of parent if
working part-time/not employed (…)  
**migration**: (Newly created) Migration status of parents
of participant (1 no migrant background, 2 migrant background)  
**unemploy**: (Newly created) Unemployment in household,
one adult/parent is unemployed (1 no unemployment, 2 unemployment)  
**singlepar**: (Newly created) Single parenthood (1 no
single parent, 2 single parent)  
**hds\_T3**: Healthy Dietary Adherence Score (range
0-50)

**Consumer attitudes as mediators**

5-point likert scale: 1 disagree, 2 moderately disagree, 3 unsure, 4
moderately agree, 5 agree

**foodst\_01\_T3**: I compare labels to select the most
nutritious food.  
**foodst\_02\_T3**: I have more confidence in food products
that I have seen advertised than in unadvertised products.  
**foodst\_03\_T3**: I try to avoid food products with
additives.  
**foodst\_04\_T3**: I make a point of using natural or
ecological products.  
**foodst\_05\_T3**: I prefer to buy meat and vegetables fresh
rather than pre-packed.  
**foodst\_06\_T3**: I frequently use ready-to-eat foods in
our household.  
**foodst\_07\_T3**: I frequently use mixes, for instance
baking mixes and powder soups.  
**foodst\_08\_T3**: The kids help in the kitchen, e.g. they
peel the potatoes and cut the vegetables.

# 0. Packages, sources, directories

```
knitr::opts_chunk$set(echo = TRUE)

require(knitr)
require(kabelExtra)
require(tidyverse)
require(table1)
require(DiagrammeR)
require(lavaan)
require(car)
```

# 1. Load and prepare data

```
## create data frame subset with all relevant variables

core_subset <- core %>%
  #only relevant variables
  select(ID_cohort, ID_no, family_id, country, age_T3, age_status_T3, sex_T3, bmi_T3, isced_cat2011_T3, income_cat_T3, migration_T3, hds_T3) %>%
  #adults only
  filter(age_status_T3 == 3) %>% 
  #exclude those with missings for HDAS and BMI
  filter(hds_T3 != "NA", bmi_T3 != "NA", country != "4", country != "9") %>%
  #create new variable for participant whose parents have migration background (no = 1, yes = 2)
  mutate(migration = ifelse(migration_T3 %in% c(1,2), 2, 1)) %>%
  #migration as integer
  mutate(migration = as.integer(migration)) %>%
  #create new categorical variable for country
  mutate(country_name = case_when(
    country == "1" ~ "Italy",
    country == "2" ~ "Estonia",
    country == "3" ~ "Cyprus",
    country == "6" ~ "Sweden",
    country == "7" ~ "Germany",
    country == "8" ~ "Hungary",
    TRUE ~ as.character(country)
  ) )

fa_subset <- fa %>%
  #only relevant variables
  select(FAMILY_ID, country, sex_fill_T3, occupst_1_T3, occupst_2_T3, no_occupst_1_T3, no_occupst_2_T3,foodst_01_T3, foodst_02_T3, foodst_03_T3, foodst_04_T3, foodst_05_T3, foodst_06_T3, foodst_07_T3, foodst_08_T3) %>%
  #exclude those with missings for consumer attitudes
  filter(foodst_01_T3 != "NA",  foodst_02_T3 != "NA", foodst_03_T3 != "NA", foodst_04_T3 != "NA", foodst_05_T3 != "NA", foodst_06_T3 != "NA", foodst_07_T3 != "NA", foodst_08_T3 != "NA"  ) %>% 
  #new variable for adults living in families with one or both adult family members unemployed (no = 1, yes = 2)
  mutate(unemploy = ifelse(no_occupst_1_T3 %in% c(5,6,7) | no_occupst_2_T3 %in% c(5,6,7), 2, 1)) %>%
  #unemployment as integer
  mutate(unemploy = as.integer(unemploy))

kh_subset <- kh %>%
  select(family_ID, country, househ_o18_T3) %>%
  #new variable for single parenthood (no = 1, yes = 2)
  mutate(singlepar = ifelse(househ_o18_T3 == 1, 2, 1)) %>%
  #singlepar as integer
  mutate(singlepar = as.integer(singlepar))

# Rename family_ID in fa and kh data frame
names(fa_subset)[names(fa_subset) == "FAMILY_ID"] <- "family_id"
names(fa_subset)[names(fa_subset) == "sex_fill_T3"] <- "sex_T3"
names(kh_subset)[names(kh_subset) == "family_ID"] <- "family_id"

# Merge data frames - automatic merging by family_id, sex, country
subset <- merge(core_subset, fa_subset)
subset <- merge(subset, kh_subset)
```

# 2. Table 1: Study sample characteristics

```
subset$isced_cat2011_T3 <-
  factor(subset$isced_cat2011_T3, levels=c(1,2,3),
         labels = c("low", "medium", "high"))
label(subset$isced_cat2011_T3) <- "Highest education level in household"

subset$income_cat_T3 <-
  factor(subset$income_cat_T3, levels=c(1,2,3,4,5),
         labels = c("low", "low/medium", "medium", "medium/high", "high"))
label(subset$income_cat_T3) <- "Household income"

subset$migration <- 
  factor(subset$migration, levels=c(2, 1),
         labels=c("Yes", 
                  "No"))
label(subset$migration) <- "Migrant background"

subset$unemploy <- 
  factor(subset$unemploy, levels=c(2, 1),
         labels=c("Yes", 
                  "No"))
label(subset$unemploy) <- "Unemployment in household"

subset$singlepar <- 
  factor(subset$singlepar, levels=c(2, 1),
         labels=c("Yes", 
                  "No"))
label(subset$singlepar) <- "Single parenthood"

subset$sex_T3 <- 
  factor(subset$sex_T3, levels=c(1, 2),
         labels=c("Male", 
                  "Female"))
label(subset$sex_T3) <- "Sex"

label(subset$hds_T3) <- "HDAS"
label(subset$bmi_T3) <- "BMI"
label(subset$age_T3) <- "Age"

units(subset$bmi_T3) <- "kg/m^2"
units(subset$age_T3) <- "years"

caption1 <- "Table 1: Study sample characteristics, excluding Belgium and Spain"
footnote1 <- "Abbreviations: HDAS = Healthy Dietary Adherence Score ; SD  = standard deviation ; BMI  = body mass index "

table1(~ hds_T3 +
         isced_cat2011_T3 + income_cat_T3 +  
         migration + unemploy + singlepar +
         bmi_T3 + age_T3 + sex_T3  | 
         country_name, data = subset, caption=caption1, footnote=footnote1)
```

Table 1: Study sample characteristics, excluding Belgium and Spain

|  | Cyprus (N=773) | Estonia (N=595) | Germany (N=509) | Hungary (N=661) | Italy (N=748) | Sweden (N=439) | Overall (N=3725) |
| --- | --- | --- | --- | --- | --- | --- | --- |
|  |  |  |  |  |  |  |  |
| --- | --- | --- | --- | --- | --- | --- | --- |
| Abbreviations: HDAS = Healthy Dietary Adherence Score ; SD = standard deviation ; BMI = body mass index | | | | | | | |
| HDAS |  |  |  |  |  |  |  |
| Mean (SD) | 25.0 (9.11) | 27.7 (7.89) | 23.0 (8.89) | 23.2 (8.88) | 24.3 (8.03) | 30.7 (8.06) | 25.4 (8.87) |
| Median [Min, Max] | 25.0 [1.00, 48.0] | 28.0 [3.00, 47.0] | 23.0 [1.00, 45.0] | 23.0 [2.00, 48.0] | 24.5 [0, 48.0] | 32.0 [7.00, 48.0] | 26.0 [0, 48.0] |
| Highest education level in household |  |  |  |  |  |  |  |
| low | 9 (1.2%) | 0 (0%) | 32 (6.3%) | 14 (2.1%) | 118 (15.8%) | 0 (0%) | 173 (4.6%) |
| medium | 283 (36.6%) | 163 (27.4%) | 293 (57.6%) | 315 (47.7%) | 453 (60.6%) | 105 (23.9%) | 1612 (43.3%) |
| high | 437 (56.5%) | 428 (71.9%) | 181 (35.6%) | 308 (46.6%) | 144 (19.3%) | 327 (74.5%) | 1825 (49.0%) |
| Missing | 44 (5.7%) | 4 (0.7%) | 3 (0.6%) | 24 (3.6%) | 33 (4.4%) | 7 (1.6%) | 115 (3.1%) |
| Household income |  |  |  |  |  |  |  |
| low | 210 (27.2%) | 71 (11.9%) | 85 (16.7%) | 93 (14.1%) | 311 (41.6%) | 10 (2.3%) | 780 (20.9%) |
| low/medium | 75 (9.7%) | 23 (3.9%) | 54 (10.6%) | 46 (7.0%) | 112 (15.0%) | 12 (2.7%) | 322 (8.6%) |
| medium | 287 (37.1%) | 148 (24.9%) | 233 (45.8%) | 240 (36.3%) | 160 (21.4%) | 146 (33.3%) | 1214 (32.6%) |
| medium/high | 80 (10.3%) | 57 (9.6%) | 59 (11.6%) | 92 (13.9%) | 11 (1.5%) | 119 (27.1%) | 418 (11.2%) |
| high | 97 (12.5%) | 284 (47.7%) | 48 (9.4%) | 149 (22.5%) | 59 (7.9%) | 146 (33.3%) | 783 (21.0%) |
| Missing | 24 (3.1%) | 12 (2.0%) | 30 (5.9%) | 41 (6.2%) | 95 (12.7%) | 6 (1.4%) | 208 (5.6%) |
| Migrant background |  |  |  |  |  |  |  |
| Yes | 152 (19.7%) | 20 (3.4%) | 100 (19.6%) | 21 (3.2%) | 132 (17.6%) | 69 (15.7%) | 494 (13.3%) |
| No | 621 (80.3%) | 575 (96.6%) | 409 (80.4%) | 640 (96.8%) | 616 (82.4%) | 370 (84.3%) | 3231 (86.7%) |
| Unemployment in household |  |  |  |  |  |  |  |
| Yes | 116 (15.0%) | 22 (3.7%) | 38 (7.5%) | 46 (7.0%) | 104 (13.9%) | 10 (2.3%) | 336 (9.0%) |
| No | 657 (85.0%) | 573 (96.3%) | 471 (92.5%) | 615 (93.0%) | 644 (86.1%) | 429 (97.7%) | 3389 (91.0%) |
| Single parenthood |  |  |  |  |  |  |  |
| Yes | 52 (6.7%) | 70 (11.8%) | 77 (15.1%) | 96 (14.5%) | 27 (3.6%) | 58 (13.2%) | 380 (10.2%) |
| No | 721 (93.3%) | 525 (88.2%) | 431 (84.7%) | 565 (85.5%) | 721 (96.4%) | 381 (86.8%) | 3344 (89.8%) |
| Missing | 0 (0%) | 0 (0%) | 1 (0.2%) | 0 (0%) | 0 (0%) | 0 (0%) | 1 (0.0%) |
| BMI (kg/m^2) |  |  |  |  |  |  |  |
| Mean (SD) | 26.2 (5.11) | 25.4 (5.37) | 26.7 (5.68) | 26.1 (5.57) | 27.4 (5.38) | 24.7 (3.90) | 26.2 (5.31) |
| Median [Min, Max] | 25.3 [17.3, 46.7] | 24.1 [16.4, 53.5] | 25.4 [16.7, 53.7] | 25.1 [15.5, 55.6] | 26.5 [17.3, 49.7] | 23.9 [17.1, 50.3] | 25.1 [15.5, 55.6] |
| Age (years) |  |  |  |  |  |  |  |
| Mean (SD) | 41.5 (5.91) | 39.6 (5.34) | 42.9 (5.87) | 40.8 (5.12) | 42.7 (5.53) | 43.8 (5.26) | 41.8 (5.68) |
| Median [Min, Max] | 41.0 [27.0, 70.6] | 39.4 [24.0, 75.9] | 42.9 [26.2, 63.0] | 40.0 [24.4, 61.1] | 42.7 [26.7, 65.9] | 43.7 [30.6, 65.4] | 41.5 [24.0, 75.9] |
| Sex |  |  |  |  |  |  |  |
| Male | 168 (21.7%) | 56 (9.4%) | 60 (11.8%) | 80 (12.1%) | 92 (12.3%) | 94 (21.4%) | 550 (14.8%) |
| Female | 605 (78.3%) | 539 (90.6%) | 449 (88.2%) | 581 (87.9%) | 656 (87.7%) | 345 (78.6%) | 3175 (85.2%) |

# 3. Table 2: Responses to consumer attitudes questionnaire

```
subset$foodst_01_T3 <-
  factor(subset$foodst_01_T3, levels=c(1,2,3,4,5),
         labels = c("Disagree", "Moderately disagree", "Unsure", "Moderately agree", "Agree"))
subset$foodst_02_T3 <-
  factor(subset$foodst_02_T3, levels=c(1,2,3,4,5),
         labels = c("Disagree", "Moderately disagree", "Unsure", "Moderately agree", "Agree"))
subset$foodst_03_T3 <-
  factor(subset$foodst_03_T3, levels=c(1,2,3,4,5),
         labels = c("Disagree", "Moderately disagree", "Unsure", "Moderately agree", "Agree"))
subset$foodst_04_T3 <-
  factor(subset$foodst_04_T3, levels=c(1,2,3,4,5),
         labels = c("Disagree", "Moderately disagree", "Unsure", "Moderately agree", "Agree"))
subset$foodst_05_T3 <-
  factor(subset$foodst_05_T3, levels=c(1,2,3,4,5),
         labels = c("Disagree", "Moderately disagree", "Unsure", "Moderately agree", "Agree"))
subset$foodst_06_T3 <-
  factor(subset$foodst_06_T3, levels=c(1,2,3,4,5),
         labels = c("Disagree", "Moderately disagree", "Unsure", "Moderately agree", "Agree"))
subset$foodst_07_T3 <-
  factor(subset$foodst_07_T3, levels=c(1,2,3,4,5),
         labels = c("Disagree", "Moderately disagree", "Unsure", "Moderately agree", "Agree"))
subset$foodst_08_T3 <-
  factor(subset$foodst_08_T3, levels=c(1,2,3,4,5),
         labels = c("Disagree", "Moderately disagree", "Unsure", "Moderately agree", "Agree"))

label(subset$foodst_01_T3) <- "Comparing food labels"
label(subset$foodst_02_T3) <- "Trusting food advertisements"
label(subset$foodst_03_T3) <- "Avoiding food additives"
label(subset$foodst_04_T3) <- "Valuing ecological products"
label(subset$foodst_05_T3) <- "Preferring fresh meat and vegetables"
label(subset$foodst_06_T3) <- "Frequently using ready-to-eat foods"
label(subset$foodst_07_T3) <- "Frequently using pre-made mixes"
label(subset$foodst_08_T3) <- "Having children help in the kitchen"

caption2 <- "Table 2: Frequency of responses in % to questions regarding various consumer attitudes, excluding Belgium and Spain"

table1(~foodst_01_T3 + foodst_02_T3 + foodst_03_T3 + foodst_04_T3 + foodst_05_T3 + foodst_06_T3 + foodst_07_T3 + foodst_08_T3 | country_name, data = subset, caption=caption2)
```

Table 2: Frequency of responses in % to questions regarding various consumer attitudes, excluding Belgium and Spain

|  | Cyprus (N=773) | Estonia (N=595) | Germany (N=509) | Hungary (N=661) | Italy (N=748) | Sweden (N=439) | Overall (N=3725) |
| --- | --- | --- | --- | --- | --- | --- | --- |
| Comparing food labels |  |  |  |  |  |  |  |
| Disagree | 58 (7.5%) | 53 (8.9%) | 40 (7.9%) | 107 (16.2%) | 66 (8.8%) | 61 (13.9%) | 385 (10.3%) |
| Moderately disagree | 74 (9.6%) | 85 (14.3%) | 147 (28.9%) | 138 (20.9%) | 58 (7.8%) | 87 (19.8%) | 589 (15.8%) |
| Unsure | 96 (12.4%) | 100 (16.8%) | 65 (12.8%) | 106 (16.0%) | 99 (13.2%) | 24 (5.5%) | 490 (13.2%) |
| Moderately agree | 292 (37.8%) | 253 (42.5%) | 203 (39.9%) | 195 (29.5%) | 291 (38.9%) | 201 (45.8%) | 1435 (38.5%) |
| Agree | 253 (32.7%) | 104 (17.5%) | 54 (10.6%) | 115 (17.4%) | 234 (31.3%) | 66 (15.0%) | 826 (22.2%) |
| Trusting food advertisements |  |  |  |  |  |  |  |
| Disagree | 336 (43.5%) | 294 (49.4%) | 230 (45.2%) | 358 (54.2%) | 260 (34.8%) | 303 (69.0%) | 1781 (47.8%) |
| Moderately disagree | 189 (24.5%) | 228 (38.3%) | 211 (41.5%) | 202 (30.6%) | 169 (22.6%) | 101 (23.0%) | 1100 (29.5%) |
| Unsure | 88 (11.4%) | 52 (8.7%) | 39 (7.7%) | 58 (8.8%) | 112 (15.0%) | 26 (5.9%) | 375 (10.1%) |
| Moderately agree | 121 (15.7%) | 20 (3.4%) | 25 (4.9%) | 34 (5.1%) | 151 (20.2%) | 9 (2.1%) | 360 (9.7%) |
| Agree | 39 (5.0%) | 1 (0.2%) | 4 (0.8%) | 9 (1.4%) | 56 (7.5%) | 0 (0%) | 109 (2.9%) |
| Avoiding food additives |  |  |  |  |  |  |  |
| Disagree | 22 (2.8%) | 17 (2.9%) | 27 (5.3%) | 25 (3.8%) | 20 (2.7%) | 31 (7.1%) | 142 (3.8%) |
| Moderately disagree | 38 (4.9%) | 35 (5.9%) | 97 (19.1%) | 42 (6.4%) | 33 (4.4%) | 78 (17.8%) | 323 (8.7%) |
| Unsure | 69 (8.9%) | 79 (13.3%) | 73 (14.3%) | 75 (11.3%) | 108 (14.4%) | 44 (10.0%) | 448 (12.0%) |
| Moderately agree | 212 (27.4%) | 260 (43.7%) | 227 (44.6%) | 218 (33.0%) | 191 (25.5%) | 189 (43.1%) | 1297 (34.8%) |
| Agree | 432 (55.9%) | 204 (34.3%) | 85 (16.7%) | 301 (45.5%) | 396 (52.9%) | 97 (22.1%) | 1515 (40.7%) |
| Valuing ecological products |  |  |  |  |  |  |  |
| Disagree | 25 (3.2%) | 60 (10.1%) | 94 (18.5%) | 46 (7.0%) | 32 (4.3%) | 44 (10.0%) | 301 (8.1%) |
| Moderately disagree | 39 (5.0%) | 136 (22.9%) | 194 (38.1%) | 67 (10.1%) | 58 (7.8%) | 109 (24.8%) | 603 (16.2%) |
| Unsure | 80 (10.3%) | 149 (25.0%) | 69 (13.6%) | 84 (12.7%) | 78 (10.4%) | 43 (9.8%) | 503 (13.5%) |
| Moderately agree | 309 (40.0%) | 183 (30.8%) | 136 (26.7%) | 249 (37.7%) | 288 (38.5%) | 192 (43.7%) | 1357 (36.4%) |
| Agree | 320 (41.4%) | 67 (11.3%) | 16 (3.1%) | 215 (32.5%) | 292 (39.0%) | 51 (11.6%) | 961 (25.8%) |
| Preferring fresh meat and vegetables |  |  |  |  |  |  |  |
| Disagree | 13 (1.7%) | 10 (1.7%) | 22 (4.3%) | 4 (0.6%) | 11 (1.5%) | 19 (4.3%) | 79 (2.1%) |
| Moderately disagree | 15 (1.9%) | 27 (4.5%) | 86 (16.9%) | 20 (3.0%) | 25 (3.3%) | 51 (11.6%) | 224 (6.0%) |
| Unsure | 16 (2.1%) | 39 (6.6%) | 41 (8.1%) | 13 (2.0%) | 10 (1.3%) | 24 (5.5%) | 143 (3.8%) |
| Moderately agree | 100 (12.9%) | 261 (43.9%) | 237 (46.6%) | 165 (25.0%) | 170 (22.7%) | 152 (34.6%) | 1085 (29.1%) |
| Agree | 629 (81.4%) | 258 (43.4%) | 123 (24.2%) | 459 (69.4%) | 532 (71.1%) | 193 (44.0%) | 2194 (58.9%) |
| Frequently using ready-to-eat foods |  |  |  |  |  |  |  |
| Disagree | 341 (44.1%) | 182 (30.6%) | 123 (24.2%) | 230 (34.8%) | 587 (78.5%) | 125 (28.5%) | 1588 (42.6%) |
| Moderately disagree | 237 (30.7%) | 311 (52.3%) | 289 (56.8%) | 309 (46.7%) | 131 (17.5%) | 169 (38.5%) | 1446 (38.8%) |
| Unsure | 41 (5.3%) | 56 (9.4%) | 45 (8.8%) | 53 (8.0%) | 9 (1.2%) | 23 (5.2%) | 227 (6.1%) |
| Moderately agree | 117 (15.1%) | 44 (7.4%) | 49 (9.6%) | 51 (7.7%) | 12 (1.6%) | 98 (22.3%) | 371 (10.0%) |
| Agree | 37 (4.8%) | 2 (0.3%) | 3 (0.6%) | 18 (2.7%) | 9 (1.2%) | 24 (5.5%) | 93 (2.5%) |
| Frequently using pre-made mixes |  |  |  |  |  |  |  |
| Disagree | 505 (65.3%) | 290 (48.7%) | 164 (32.2%) | 405 (61.3%) | 629 (84.1%) | 322 (73.3%) | 2315 (62.1%) |
| Moderately disagree | 153 (19.8%) | 223 (37.5%) | 261 (51.3%) | 199 (30.1%) | 91 (12.2%) | 87 (19.8%) | 1014 (27.2%) |
| Unsure | 34 (4.4%) | 43 (7.2%) | 33 (6.5%) | 28 (4.2%) | 5 (0.7%) | 9 (2.1%) | 152 (4.1%) |
| Moderately agree | 59 (7.6%) | 36 (6.1%) | 47 (9.2%) | 23 (3.5%) | 20 (2.7%) | 17 (3.9%) | 202 (5.4%) |
| Agree | 22 (2.8%) | 3 (0.5%) | 4 (0.8%) | 6 (0.9%) | 3 (0.4%) | 4 (0.9%) | 42 (1.1%) |
| Having children help in the kitchen |  |  |  |  |  |  |  |
| Disagree | 100 (12.9%) | 56 (9.4%) | 25 (4.9%) | 50 (7.6%) | 236 (31.6%) | 32 (7.3%) | 499 (13.4%) |
| Moderately disagree | 105 (13.6%) | 164 (27.6%) | 147 (28.9%) | 91 (13.8%) | 76 (10.2%) | 112 (25.5%) | 695 (18.7%) |
| Unsure | 61 (7.9%) | 86 (14.5%) | 33 (6.5%) | 46 (7.0%) | 36 (4.8%) | 28 (6.4%) | 290 (7.8%) |
| Moderately agree | 279 (36.1%) | 229 (38.5%) | 217 (42.6%) | 246 (37.2%) | 260 (34.8%) | 196 (44.6%) | 1427 (38.3%) |
| Agree | 228 (29.5%) | 60 (10.1%) | 87 (17.1%) | 228 (34.5%) | 140 (18.7%) | 71 (16.2%) | 814 (21.9%) |

# 4. Analysis

```
model_mc <- lm(hds_T3~income_cat_T3+isced_cat2011_T3+migration+singlepar+unemploy, data=subset)

vif(model_mc)
```

```
##    income_cat_T3 isced_cat2011_T3        migration        singlepar 
##         1.412392         1.263649         1.020964         1.054861 
##         unemploy 
##         1.080933
```

## 4.1 Path analysis

We performed regression analysis for the association between
socioeconomic factors / vulnerabilities and consumer attitudes (path a),
the association between consumer attitudes and HDAS (path b) and the
association between socioeconomic factors / vulnerabilites and HDAS
(total effect, path c). All models are adjusted for age, sex and
BMI.

```
require(MASS)
```

### 4.1.2 Path a: Socioeconomic factors -> Consumer attitudes

```
# Consumer attitudes as factors

subset$foodst_01_T3 <- relevel(as.factor(subset$foodst_01_T3), ref = 1)
subset$foodst_02_T3 <- relevel(as.factor(subset$foodst_02_T3), ref = 1)
subset$foodst_03_T3 <- relevel(as.factor(subset$foodst_03_T3), ref = 1)
subset$foodst_04_T3 <- relevel(as.factor(subset$foodst_04_T3), ref = 1)
subset$foodst_05_T3 <- relevel(as.factor(subset$foodst_05_T3), ref = 1)
subset$foodst_06_T3 <- relevel(as.factor(subset$foodst_06_T3), ref = 1)
subset$foodst_07_T3 <- relevel(as.factor(subset$foodst_07_T3), ref = 1)
subset$foodst_08_T3 <- relevel(as.factor(subset$foodst_08_T3), ref = 1)
```

```
# path a
# Education -> Consumer attitudes

edu1 <- polr(foodst_01_T3 ~ isced_cat2011_T3 + age_T3 + sex_T3 + bmi_T3, data = subset, Hess=TRUE)
summary(edu1)
```

```
## Call:
## polr(formula = foodst_01_T3 ~ isced_cat2011_T3 + age_T3 + sex_T3 + 
##     bmi_T3, data = subset, Hess = TRUE)
## 
## Coefficients:
##                      Value Std. Error t value
## isced_cat2011_T3  0.120259   0.052574  2.2874
## age_T3            0.009579   0.005519  1.7357
## sex_T3            0.057953   0.088249  0.6567
## bmi_T3           -0.011225   0.005759 -1.9489
## 
## Intercepts:
##     Value   Std. Error t value
## 1|2 -1.6686  0.3817    -4.3720
## 2|3 -0.5315  0.3796    -1.4002
## 3|4  0.0759  0.3793     0.2001
## 4|5  1.7807  0.3807     4.6779
## 
## Residual Deviance: 10770.13 
## AIC: 10786.13 
## (115 observations deleted due to missingness)
```

```
ctable_edu1 <- coef(summary(edu1))
p_edu1 <- pnorm(abs(ctable_edu1[, "t value"]), lower.tail = FALSE) * 2
(ctable_edu1 <- cbind(ctable_edu1, "p-value" =  round(p_edu1, 4)))
```

```
##                         Value  Std. Error    t value p-value
## isced_cat2011_T3  0.120258749 0.052573761  2.2874291  0.0222
## age_T3            0.009579035 0.005518926  1.7356702  0.0826
## sex_T3            0.057952655 0.088248615  0.6566976  0.5114
## bmi_T3           -0.011224508 0.005759457 -1.9488831  0.0513
## 1|2              -1.668597646 0.381651188 -4.3720489  0.0000
## 2|3              -0.531536419 0.379614842 -1.4001993  0.1615
## 3|4               0.075910267 0.379298690  0.2001332  0.8414
## 4|5               1.780740129 0.380673187  4.6778712  0.0000
```

```
(ci_edu1 <- confint(edu1, level=0.99375))
```

```
## Waiting for profiling to be done...
```

```
##                         0.3 %      99.7 %
## isced_cat2011_T3 -0.023449158 0.264122418
## age_T3           -0.005512975 0.024673652
## sex_T3           -0.183636041 0.299127006
## bmi_T3           -0.026969021 0.004539792
```

```
exp(cbind(OR = coef(edu1), ci_edu1))
```

```
##                         OR     0.3 %   99.7 %
## isced_cat2011_T3 1.1277886 0.9768236 1.302288
## age_T3           1.0096251 0.9945022 1.024981
## sex_T3           1.0596648 0.8322386 1.348681
## bmi_T3           0.9888383 0.9733914 1.004550
```

```
edu2 <- polr(foodst_02_T3 ~ isced_cat2011_T3 + age_T3 + sex_T3 + bmi_T3, data = subset, Hess=TRUE)
summary(edu2)
```

```
## Call:
## polr(formula = foodst_02_T3 ~ isced_cat2011_T3 + age_T3 + sex_T3 + 
##     bmi_T3, data = subset, Hess = TRUE)
## 
## Coefficients:
##                       Value Std. Error  t value
## isced_cat2011_T3 -0.3708374   0.054976 -6.74549
## age_T3            0.0001831   0.005716  0.03202
## sex_T3           -0.0644322   0.091721 -0.70248
## bmi_T3            0.0100237   0.006015  1.66651
## 
## Intercepts:
##     Value   Std. Error t value
## 1|2 -0.8667  0.3911    -2.2158
## 2|3  0.4817  0.3909     1.2322
## 3|4  1.2164  0.3918     3.1044
## 4|5  2.7997  0.4010     6.9816
## 
## Residual Deviance: 9123.511 
## AIC: 9139.511 
## (115 observations deleted due to missingness)
```

```
ctable_edu2 <- coef(summary(edu2))
p_edu2 <- pnorm(abs(ctable_edu2[, "t value"]), lower.tail = FALSE) * 2
(ctable_edu2 <- cbind(ctable_edu2, "p-value" =  round(p_edu2, 4)))
```

```
##                          Value  Std. Error     t value p-value
## isced_cat2011_T3 -0.3708373753 0.054975631 -6.74548642  0.0000
## age_T3            0.0001830579 0.005716401  0.03202329  0.9745
## sex_T3           -0.0644322374 0.091720942 -0.70248120  0.4824
## bmi_T3            0.0100237244 0.006014792  1.66651235  0.0956
## 1|2              -0.8666555914 0.391122556 -2.21581594  0.0267
## 2|3               0.4817050842 0.390939471  1.23217306  0.2179
## 3|4               1.2164051552 0.391829629  3.10442362  0.0019
## 4|5               2.7997129248 0.401011838  6.98162163  0.0000
```

```
(ci_edu2 <- confint(edu2, level=0.99375))
```

```
## Waiting for profiling to be done...
```

```
##                         0.3 %      99.7 %
## isced_cat2011_T3 -0.521276853 -0.22055802
## age_T3           -0.015461876  0.01580728
## sex_T3           -0.314340620  0.18753239
## bmi_T3           -0.006457386  0.02645140
```

```
exp(cbind(OR = coef(edu2), ci_edu2))
```

```
##                         OR     0.3 %    99.7 %
## isced_cat2011_T3 0.6901562 0.5937619 0.8020711
## age_T3           1.0001831 0.9846570 1.0159329
## sex_T3           0.9375996 0.7302702 1.2062693
## bmi_T3           1.0100741 0.9935634 1.0268043
```

```
edu3 <- polr(foodst_03_T3 ~ isced_cat2011_T3 + age_T3 + sex_T3 + bmi_T3, data = subset, Hess=TRUE)
summary(edu3)
```

```
## Call:
## polr(formula = foodst_03_T3 ~ isced_cat2011_T3 + age_T3 + sex_T3 + 
##     bmi_T3, data = subset, Hess = TRUE)
## 
## Coefficients:
##                     Value Std. Error t value
## isced_cat2011_T3  0.13057   0.054203   2.409
## age_T3            0.02183   0.005591   3.904
## sex_T3            0.30909   0.089585   3.450
## bmi_T3           -0.01349   0.005892  -2.290
## 
## Intercepts:
##     Value   Std. Error t value
## 1|2 -1.7886  0.3916    -4.5679
## 2|3 -0.5085  0.3857    -1.3184
## 3|4  0.3210  0.3850     0.8338
## 4|5  1.8494  0.3863     4.7880
## 
## Residual Deviance: 9527.228 
## AIC: 9543.228 
## (115 observations deleted due to missingness)
```

```
ctable_edu3 <- coef(summary(edu3))
p_edu3 <- pnorm(abs(ctable_edu3[, "t value"]), lower.tail = FALSE) * 2
(ctable_edu3 <- cbind(ctable_edu3, "p-value" =  round(p_edu3, 4)))
```

```
##                        Value  Std. Error    t value p-value
## isced_cat2011_T3  0.13057391 0.054203182  2.4089712  0.0160
## age_T3            0.02182955 0.005591238  3.9042428  0.0001
## sex_T3            0.30908699 0.089584593  3.4502249  0.0006
## bmi_T3           -0.01349002 0.005892001 -2.2895478  0.0220
## 1|2              -1.78864415 0.391566786 -4.5679159  0.0000
## 2|3              -0.50847029 0.385670042 -1.3184075  0.1874
## 3|4               0.32104928 0.385025156  0.8338398  0.4044
## 4|5               1.84944104 0.386268785  4.7879640  0.0000
```

```
(ci_edu3 <- confint(edu3, level=0.99375))
```

```
## Waiting for profiling to be done...
```

```
##                         0.3 %      99.7 %
## isced_cat2011_T3 -0.017679403 0.278812780
## age_T3            0.006567108 0.037150189
## sex_T3            0.063756288 0.553867207
## bmi_T3           -0.029579659 0.002654445
```

```
exp(cbind(OR = coef(edu3), ci_edu3))
```

```
##                         OR     0.3 %   99.7 %
## isced_cat2011_T3 1.1394822 0.9824760 1.321560
## age_T3           1.0220696 1.0065887 1.037849
## sex_T3           1.3621809 1.0658326 1.739969
## bmi_T3           0.9866006 0.9708535 1.002658
```

```
edu4 <- polr(foodst_04_T3 ~ isced_cat2011_T3 + age_T3 + sex_T3 + bmi_T3, data = subset, Hess=TRUE)
summary(edu4)
```

```
## Call:
## polr(formula = foodst_04_T3 ~ isced_cat2011_T3 + age_T3 + sex_T3 + 
##     bmi_T3, data = subset, Hess = TRUE)
## 
## Coefficients:
##                      Value Std. Error t value
## isced_cat2011_T3  0.042842   0.053429  0.8018
## age_T3            0.025158   0.005552  4.5315
## sex_T3            0.058070   0.088485  0.6563
## bmi_T3           -0.006029   0.005786 -1.0420
## 
## Intercepts:
##     Value   Std. Error t value
## 1|2 -1.3253  0.3839    -3.4519
## 2|3 -0.0226  0.3818    -0.0592
## 3|4  0.6222  0.3818     1.6296
## 4|5  2.1932  0.3835     5.7193
## 
## Residual Deviance: 10722.18 
## AIC: 10738.18 
## (115 observations deleted due to missingness)
```

```
ctable_edu4 <- coef(summary(edu4))
p_edu4 <- pnorm(abs(ctable_edu4[, "t value"]), lower.tail = FALSE) * 2
(ctable_edu4 <- cbind(ctable_edu4, "p-value" =  round(p_edu4, 4)))
```

```
##                         Value  Std. Error     t value p-value
## isced_cat2011_T3  0.042841761 0.053428787  0.80184791  0.4226
## age_T3            0.025157729 0.005551796  4.53145792  0.0000
## sex_T3            0.058069535 0.088485132  0.65626319  0.5117
## bmi_T3           -0.006029155 0.005786238 -1.04198177  0.2974
## 1|2              -1.325306718 0.383933536 -3.45191705  0.0006
## 2|3              -0.022613739 0.381774348 -0.05923326  0.9528
## 3|4               0.622233057 0.381840191  1.62956407  0.1032
## 4|5               2.193184141 0.383467777  5.71934404  0.0000
```

```
(ci_edu4 <- confint(edu4, level=0.99375))
```

```
## Waiting for profiling to be done...
```

```
##                         0.3 %      99.7 %
## isced_cat2011_T3 -0.103242669 0.189002555
## age_T3            0.009992149 0.040359298
## sex_T3           -0.184178594 0.299887914
## bmi_T3           -0.021842968 0.009811726
```

```
exp(cbind(OR = coef(edu4), ci_edu4))
```

```
##                        OR     0.3 %   99.7 %
## isced_cat2011_T3 1.043773 0.9019081 1.208044
## age_T3           1.025477 1.0100422 1.041185
## sex_T3           1.059789 0.8317872 1.349708
## bmi_T3           0.993989 0.9783939 1.009860
```

```
edu5 <- polr(foodst_05_T3 ~ isced_cat2011_T3 + age_T3 + sex_T3 + bmi_T3, data = subset, Hess=TRUE)
summary(edu5)
```

```
## Call:
## polr(formula = foodst_05_T3 ~ isced_cat2011_T3 + age_T3 + sex_T3 + 
##     bmi_T3, data = subset, Hess = TRUE)
## 
## Coefficients:
##                      Value Std. Error t value
## isced_cat2011_T3 -0.137315   0.058058 -2.3651
## age_T3            0.005336   0.006048  0.8824
## sex_T3            0.073003   0.096771  0.7544
## bmi_T3            0.003560   0.006363  0.5595
## 
## Intercepts:
##     Value   Std. Error t value
## 1|2 -3.7034  0.4292    -8.6287
## 2|3 -2.2991  0.4181    -5.4991
## 3|4 -1.8758  0.4170    -4.4988
## 4|5 -0.2285  0.4154    -0.5500
## 
## Residual Deviance: 7588.362 
## AIC: 7604.362 
## (115 observations deleted due to missingness)
```

```
ctable_edu5 <- coef(summary(edu5))
p_edu5 <- pnorm(abs(ctable_edu5[, "t value"]), lower.tail = FALSE) * 2
(ctable_edu5 <- cbind(ctable_edu5, "p-value" =  round(p_edu5, 4)))
```

```
##                         Value  Std. Error    t value p-value
## isced_cat2011_T3 -0.137315018 0.058058306 -2.3651227  0.0180
## age_T3            0.005336195 0.006047701  0.8823510  0.3776
## sex_T3            0.073002501 0.096771248  0.7543821  0.4506
## bmi_T3            0.003560140 0.006362737  0.5595297  0.5758
## 1|2              -3.703413169 0.429198140 -8.6286795  0.0000
## 2|3              -2.299147700 0.418094111 -5.4991153  0.0000
## 3|4              -1.875789907 0.416957372 -4.4987570  0.0000
## 4|5              -0.228492221 0.415449648 -0.5499878  0.5823
```

```
(ci_edu5 <- confint(edu5, level=0.99375))
```

```
## Waiting for profiling to be done...
```

```
##                        0.3 %     99.7 %
## isced_cat2011_T3 -0.29655696 0.02106225
## age_T3           -0.01116872 0.02191590
## sex_T3           -0.19366757 0.33593132
## bmi_T3           -0.01374106 0.02107930
```

```
exp(cbind(OR = coef(edu5), ci_edu5))
```

```
##                         OR     0.3 %   99.7 %
## isced_cat2011_T3 0.8716956 0.7433733 1.021286
## age_T3           1.0053505 0.9888934 1.022158
## sex_T3           1.0757332 0.8239318 1.399243
## bmi_T3           1.0035665 0.9863529 1.021303
```

```
edu6 <- polr(foodst_06_T3 ~ isced_cat2011_T3 + age_T3 + sex_T3 + bmi_T3, data = subset, Hess=TRUE)
summary(edu6)
```

```
## Call:
## polr(formula = foodst_06_T3 ~ isced_cat2011_T3 + age_T3 + sex_T3 + 
##     bmi_T3, data = subset, Hess = TRUE)
## 
## Coefficients:
##                      Value Std. Error t value
## isced_cat2011_T3  0.484022   0.056265  8.6026
## age_T3           -0.030104   0.005775 -5.2130
## sex_T3           -0.323526   0.091277 -3.5444
## bmi_T3           -0.004284   0.006073 -0.7054
## 
## Intercepts:
##     Value   Std. Error t value
## 1|2 -1.0892  0.3966    -2.7463
## 2|3  0.7466  0.3964     1.8833
## 3|4  1.2156  0.3971     3.0610
## 4|5  2.9275  0.4076     7.1814
## 
## Residual Deviance: 8713.257 
## AIC: 8729.257 
## (115 observations deleted due to missingness)
```

```
ctable_edu6 <- coef(summary(edu6))
p_edu6 <- pnorm(abs(ctable_edu6[, "t value"]), lower.tail = FALSE) * 2
(ctable_edu6 <- cbind(ctable_edu6, "p-value" =  round(p_edu6, 4)))
```

```
##                         Value  Std. Error    t value p-value
## isced_cat2011_T3  0.484021633 0.056264633  8.6025912  0.0000
## age_T3           -0.030103550 0.005774698 -5.2130087  0.0000
## sex_T3           -0.323525631 0.091277088 -3.5444342  0.0004
## bmi_T3           -0.004284136 0.006073209 -0.7054155  0.4806
## 1|2              -1.089234878 0.396625154 -2.7462577  0.0060
## 2|3               0.746603500 0.396427191  1.8833307  0.0597
## 3|4               1.215638548 0.397131675  3.0610466  0.0022
## 4|5               2.927472826 0.407646562  7.1813995  0.0000
```

```
(ci_edu6 <- confint(edu6, level=0.99375))
```

```
## Waiting for profiling to be done...
```

```
##                        0.3 %      99.7 %
## isced_cat2011_T3  0.33070479  0.63849107
## age_T3           -0.04594495 -0.01435716
## sex_T3           -0.57292998 -0.07356158
## bmi_T3           -0.02094860  0.01227982
```

```
exp(cbind(OR = coef(edu6), ci_edu6))
```

```
##                         OR     0.3 %    99.7 %
## isced_cat2011_T3 1.6225867 1.3919488 1.8936214
## age_T3           0.9703450 0.9550945 0.9857454
## sex_T3           0.7235934 0.5638709 0.9290789
## bmi_T3           0.9957250 0.9792693 1.0123555
```

```
edu7 <- polr(foodst_07_T3 ~ isced_cat2011_T3 + age_T3 + sex_T3 + bmi_T3, data = subset, Hess=TRUE)
summary(edu7)
```

```
## Call:
## polr(formula = foodst_07_T3 ~ isced_cat2011_T3 + age_T3 + sex_T3 + 
##     bmi_T3, data = subset, Hess = TRUE)
## 
## Coefficients:
##                     Value Std. Error t value
## isced_cat2011_T3  0.13459   0.059453   2.264
## age_T3           -0.02415   0.006200  -3.895
## sex_T3           -0.34425   0.096100  -3.582
## bmi_T3            0.01371   0.006459   2.122
## 
## Intercepts:
##     Value   Std. Error t value
## 1|2 -0.4741  0.4220    -1.1233
## 2|3  1.1833  0.4232     2.7964
## 3|4  1.7151  0.4249     4.0366
## 4|5  3.5774  0.4492     7.9642
## 
## Residual Deviance: 7129.302 
## AIC: 7145.302 
## (115 observations deleted due to missingness)
```

```
ctable_edu7 <- coef(summary(edu7))
p_edu7 <- pnorm(abs(ctable_edu7[, "t value"]), lower.tail = FALSE) * 2
(ctable_edu7 <- cbind(ctable_edu7, "p-value" =  round(p_edu7, 4)))
```

```
##                        Value  Std. Error   t value p-value
## isced_cat2011_T3  0.13458917 0.059453079  2.263788  0.0236
## age_T3           -0.02415071 0.006199767 -3.895423  0.0001
## sex_T3           -0.34424894 0.096100143 -3.582190  0.0003
## bmi_T3            0.01370835 0.006459202  2.122298  0.0338
## 1|2              -0.47405092 0.422008807 -1.123320  0.2613
## 2|3               1.18331282 0.423152335  2.796423  0.0052
## 3|4               1.71514788 0.424899252  4.036599  0.0001
## 4|5               3.57736923 0.449179281  7.964235  0.0000
```

```
(ci_edu7 <- confint(edu7, level=0.99375))
```

```
## Waiting for profiling to be done...
```

```
##                         0.3 %       99.7 %
## isced_cat2011_T3 -0.027462617  0.297794251
## age_T3           -0.041179131 -0.007261695
## sex_T3           -0.605738681 -0.079828953
## bmi_T3           -0.004057472  0.031288064
```

```
exp(cbind(OR = coef(edu7), ci_edu7))
```

```
##                         OR     0.3 %    99.7 %
## isced_cat2011_T3 1.1440667 0.9729111 1.3468846
## age_T3           0.9761386 0.9596572 0.9927646
## sex_T3           0.7087525 0.5456712 0.9232743
## bmi_T3           1.0138027 0.9959507 1.0317827
```

```
edu8 <- polr(foodst_08_T3 ~ isced_cat2011_T3 + age_T3 + sex_T3 + bmi_T3, data = subset, Hess=TRUE)
summary(edu8)
```

```
## Call:
## polr(formula = foodst_08_T3 ~ isced_cat2011_T3 + age_T3 + sex_T3 + 
##     bmi_T3, data = subset, Hess = TRUE)
## 
## Coefficients:
##                      Value Std. Error  t value
## isced_cat2011_T3 -0.029869   0.053379 -0.55957
## age_T3            0.000124   0.005504  0.02254
## sex_T3            0.360188   0.088231  4.08231
## bmi_T3            0.012314   0.005758  2.13876
## 
## Intercepts:
##     Value   Std. Error t value
## 1|2 -0.9468  0.3808    -2.4864
## 2|3  0.1772  0.3800     0.4664
## 3|4  0.5197  0.3800     1.3677
## 4|5  2.2254  0.3817     5.8309
## 
## Residual Deviance: 10677.23 
## AIC: 10693.23 
## (115 observations deleted due to missingness)
```

```
ctable_edu8 <- coef(summary(edu8))
p_edu8 <- pnorm(abs(ctable_edu8[, "t value"]), lower.tail = FALSE) * 2
(ctable_edu8 <- cbind(ctable_edu8, "p-value" =  round(p_edu8, 4)))
```

```
##                          Value  Std. Error     t value p-value
## isced_cat2011_T3 -0.0298693777 0.053379088 -0.55957078  0.5758
## age_T3            0.0001240483 0.005503825  0.02253855  0.9820
## sex_T3            0.3601880741 0.088231384  4.08231243  0.0000
## bmi_T3            0.0123142500 0.005757662  2.13875887  0.0325
## 1|2              -0.9467850166 0.380778186 -2.48644762  0.0129
## 2|3               0.1772316166 0.379976189  0.46642822  0.6409
## 3|4               0.5197375861 0.380019573  1.36766004  0.1714
## 4|5               2.2254389036 0.381662936  5.83090128  0.0000
```

```
(ci_edu8 <- confint(edu8, level=0.99375))
```

```
## Waiting for profiling to be done...
```

```
##                         0.3 %     99.7 %
## isced_cat2011_T3 -0.175912893 0.11605763
## age_T3           -0.014928988 0.01517625
## sex_T3            0.118829154 0.60150985
## bmi_T3           -0.003409189 0.02808833
```

```
exp(cbind(OR = coef(edu8), ci_edu8))
```

```
##                         OR     0.3 %   99.7 %
## isced_cat2011_T3 0.9705723 0.8386910 1.123061
## age_T3           1.0001241 0.9851819 1.015292
## sex_T3           1.4335990 1.1261775 1.824872
## bmi_T3           1.0123904 0.9965966 1.028487
```

```
# path a
# Income -> Consumer attitudes

inc1 <- polr(foodst_01_T3 ~ income_cat_T3 + age_T3 + sex_T3 + bmi_T3, data = subset, Hess=TRUE)
summary(inc1)
```

```
## Call:
## polr(formula = foodst_01_T3 ~ income_cat_T3 + age_T3 + sex_T3 + 
##     bmi_T3, data = subset, Hess = TRUE)
## 
## Coefficients:
##                   Value Std. Error t value
## income_cat_T3 -0.012083   0.021957 -0.5503
## age_T3         0.007974   0.005546  1.4379
## sex_T3         0.016510   0.088002  0.1876
## bmi_T3        -0.013042   0.005838 -2.2340
## 
## Intercepts:
##     Value   Std. Error t value
## 1|2 -2.1795  0.3658    -5.9587
## 2|3 -1.0566  0.3634    -2.9072
## 3|4 -0.4481  0.3630    -1.2345
## 4|5  1.2718  0.3637     3.4970
## 
## Residual Deviance: 10490.96 
## AIC: 10506.96 
## (208 observations deleted due to missingness)
```

```
ctable_inc1 <- coef(summary(inc1))
p_inc1 <- pnorm(abs(ctable_inc1[, "t value"]), lower.tail = FALSE) * 2
(ctable_inc1 <- cbind(ctable_inc1, "p-value" =  round(p_inc1, 4)))
```

```
##                      Value  Std. Error    t value p-value
## income_cat_T3 -0.012083070 0.021956761 -0.5503121  0.5821
## age_T3         0.007973897 0.005545569  1.4378863  0.1505
## sex_T3         0.016510070 0.088002279  0.1876096  0.8512
## bmi_T3        -0.013042262 0.005838023 -2.2340205  0.0255
## 1|2           -2.179529003 0.365771389 -5.9587192  0.0000
## 2|3           -1.056589022 0.363442053 -2.9071733  0.0036
## 3|4           -0.448074071 0.362956549 -1.2345116  0.2170
## 4|5            1.271794585 0.363683134  3.4969853  0.0005
```

```
(ci_inc1 <- confint(inc1, level=0.99375))
```

```
## Waiting for profiling to be done...
```

```
##                      0.3 %      99.7 %
## income_cat_T3 -0.072146059 0.047950839
## age_T3        -0.007190767 0.023141822
## sex_T3        -0.224428797 0.256981135
## bmi_T3        -0.029003247 0.002935149
```

```
exp(cbind(OR = coef(inc1), ci_inc1))
```

```
##                      OR     0.3 %   99.7 %
## income_cat_T3 0.9879896 0.9303950 1.049119
## age_T3        1.0080058 0.9928350 1.023412
## sex_T3        1.0166471 0.7989725 1.293021
## bmi_T3        0.9870424 0.9714133 1.002939
```

```
inc2 <- polr(foodst_02_T3 ~ income_cat_T3 + age_T3 + sex_T3 + bmi_T3, data = subset, Hess=TRUE)
summary(inc2)
```

```
## Call:
## polr(formula = foodst_02_T3 ~ income_cat_T3 + age_T3 + sex_T3 + 
##     bmi_T3, data = subset, Hess = TRUE)
## 
## Coefficients:
##                   Value Std. Error t value
## income_cat_T3 -0.144289   0.022920 -6.2955
## age_T3        -0.006046   0.005756 -1.0506
## sex_T3        -0.073190   0.091548 -0.7995
## bmi_T3         0.012897   0.006104  2.1128
## 
## Intercepts:
##     Value   Std. Error t value
## 1|2 -0.5599  0.3750    -1.4932
## 2|3  0.7702  0.3751     2.0535
## 3|4  1.4820  0.3761     3.9406
## 4|5  3.0864  0.3863     7.9897
## 
## Residual Deviance: 8838.307 
## AIC: 8854.307 
## (208 observations deleted due to missingness)
```

```
ctable_inc2 <- coef(summary(inc2))
p_inc2 <- pnorm(abs(ctable_inc2[, "t value"]), lower.tail = FALSE) * 2
(ctable_inc2 <- cbind(ctable_inc2, "p-value" =  round(p_inc2, 4)))
```

```
##                      Value  Std. Error    t value p-value
## income_cat_T3 -0.144289167 0.022919532 -6.2954675  0.0000
## age_T3        -0.006046471 0.005755510 -1.0505535  0.2935
## sex_T3        -0.073190401 0.091547907 -0.7994765  0.4240
## bmi_T3         0.012897264 0.006104309  2.1128129  0.0346
## 1|2           -0.559925571 0.374985434 -1.4931929  0.1354
## 2|3            0.770203310 0.375064482  2.0535224  0.0400
## 3|4            1.482032372 0.376094206  3.9405882  0.0001
## 4|5            3.086383565 0.386294276  7.9897212  0.0000
```

```
(ci_inc2 <- confint(inc2, level=0.99375))
```

```
## Waiting for profiling to be done...
```

```
##                      0.3 %       99.7 %
## income_cat_T3 -0.207083583 -0.081712376
## age_T3        -0.021806121  0.009677565
## sex_T3        -0.322628498  0.178295041
## bmi_T3        -0.003830274  0.029570386
```

```
exp(cbind(OR = coef(inc2), ci_inc2))
```

```
##                      OR     0.3 %   99.7 %
## income_cat_T3 0.8656374 0.8129517 0.921537
## age_T3        0.9939718 0.9784299 1.009725
## sex_T3        0.9294239 0.7242429 1.195178
## bmi_T3        1.0129808 0.9961771 1.030012
```

```
inc3 <- polr(foodst_03_T3 ~ income_cat_T3 + age_T3 + sex_T3 + bmi_T3, data = subset, Hess=TRUE)
summary(inc3)
```

```
## Call:
## polr(formula = foodst_03_T3 ~ income_cat_T3 + age_T3 + sex_T3 + 
##     bmi_T3, data = subset, Hess = TRUE)
## 
## Coefficients:
##                  Value Std. Error t value
## income_cat_T3 -0.03247   0.022519  -1.442
## age_T3         0.02230   0.005645   3.951
## sex_T3         0.27552   0.089383   3.082
## bmi_T3        -0.01905   0.006002  -3.174
## 
## Intercepts:
##     Value   Std. Error t value
## 1|2 -2.4079  0.3763    -6.3985
## 2|3 -1.1262  0.3697    -3.0463
## 3|4 -0.3010  0.3688    -0.8162
## 4|5  1.2315  0.3694     3.3336
## 
## Residual Deviance: 9263.745 
## AIC: 9279.745 
## (208 observations deleted due to missingness)
```

```
ctable_inc3 <- coef(summary(inc3))
p_inc3 <- pnorm(abs(ctable_inc3[, "t value"]), lower.tail = FALSE) * 2
(ctable_inc3 <- cbind(ctable_inc3, "p-value" =  round(p_inc3, 4)))
```

```
##                     Value  Std. Error    t value p-value
## income_cat_T3 -0.03247337 0.022518654 -1.4420655  0.1493
## age_T3         0.02230213 0.005644972  3.9507949  0.0001
## sex_T3         0.27551715 0.089382809  3.0824401  0.0021
## bmi_T3        -0.01905061 0.006001785 -3.1741569  0.0015
## 1|2           -2.40788331 0.376321908 -6.3984670  0.0000
## 2|3           -1.12616075 0.369686946 -3.0462551  0.0023
## 3|4           -0.30099783 0.368774230 -0.8162117  0.4144
## 4|5            1.23145788 0.369405821  3.3336180  0.0009
```

```
(ci_inc3 <- confint(inc3, level=0.99375))
```

```
## Waiting for profiling to be done...
```

```
##                      0.3 %       99.7 %
## income_cat_T3 -0.094104475  0.029070873
## age_T3         0.006893104  0.037770534
## sex_T3         0.030718942  0.519723986
## bmi_T3        -0.035447285 -0.002612261
```

```
exp(cbind(OR = coef(inc3), ci_inc3))
```

```
##                      OR     0.3 %    99.7 %
## income_cat_T3 0.9680482 0.9101877 1.0294976
## age_T3        1.0225527 1.0069169 1.0384929
## sex_T3        1.3172117 1.0311956 1.6815635
## bmi_T3        0.9811297 0.9651736 0.9973911
```

```
inc4 <- polr(foodst_04_T3 ~ income_cat_T3 + age_T3 + sex_T3 + bmi_T3, data = subset, Hess=TRUE)
summary(inc4)
```

```
## Call:
## polr(formula = foodst_04_T3 ~ income_cat_T3 + age_T3 + sex_T3 + 
##     bmi_T3, data = subset, Hess = TRUE)
## 
## Coefficients:
##                   Value Std. Error  t value
## income_cat_T3 -0.101588   0.021990 -4.61971
## age_T3         0.026449   0.005585  4.73574
## sex_T3        -0.007861   0.088319 -0.08901
## bmi_T3        -0.012832   0.005892 -2.17807
## 
## Intercepts:
##     Value   Std. Error t value
## 1|2 -1.9937  0.3678    -5.4213
## 2|3 -0.6968  0.3649    -1.9093
## 3|4 -0.0458  0.3647    -0.1256
## 4|5  1.5318  0.3656     4.1892
## 
## Residual Deviance: 10426.75 
## AIC: 10442.75 
## (208 observations deleted due to missingness)
```

```
ctable_inc4 <- coef(summary(inc4))
p_inc4 <- pnorm(abs(ctable_inc4[, "t value"]), lower.tail = FALSE) * 2
(ctable_inc4 <- cbind(ctable_inc4, "p-value" =  round(p_inc4, 4)))
```

```
##                      Value  Std. Error     t value p-value
## income_cat_T3 -0.101587563 0.021990019 -4.61971240  0.0000
## age_T3         0.026449360 0.005585050  4.73574290  0.0000
## sex_T3        -0.007860828 0.088318656 -0.08900529  0.9291
## bmi_T3        -0.012832453 0.005891657 -2.17807183  0.0294
## 1|2           -1.993721713 0.367753925 -5.42134720  0.0000
## 2|3           -0.696775395 0.364939992 -1.90928758  0.0562
## 3|4           -0.045817688 0.364669703 -0.12564161  0.9000
## 4|5            1.531782270 0.365649919  4.18920446  0.0000
```

```
(ci_inc4 <- confint(inc4, level=0.99375))
```

```
## Waiting for profiling to be done...
```

```
##                     0.3 %       99.7 %
## income_cat_T3 -0.16179493 -0.041515705
## age_T3         0.01119711  0.041746513
## sex_T3        -0.24968543  0.233468226
## bmi_T3        -0.02893839  0.003293283
```

```
exp(cbind(OR = coef(inc4), ci_inc4))
```

```
##                      OR     0.3 %    99.7 %
## income_cat_T3 0.9034021 0.8506156 0.9593343
## age_T3        1.0268022 1.0112600 1.0426302
## sex_T3        0.9921700 0.7790458 1.2629727
## bmi_T3        0.9872495 0.9714763 1.0032987
```

```
inc5 <- polr(foodst_05_T3 ~ income_cat_T3 + age_T3 + sex_T3 + bmi_T3, data = subset, Hess=TRUE)
summary(inc5)
```

```
## Call:
## polr(formula = foodst_05_T3 ~ income_cat_T3 + age_T3 + sex_T3 + 
##     bmi_T3, data = subset, Hess = TRUE)
## 
## Coefficients:
##                   Value Std. Error t value
## income_cat_T3 -0.100654   0.024016 -4.1912
## age_T3         0.001421   0.006100  0.2330
## sex_T3         0.028990   0.096532  0.3003
## bmi_T3         0.002593   0.006467  0.4009
## 
## Intercepts:
##     Value   Std. Error t value
## 1|2 -3.9473  0.4134    -9.5478
## 2|3 -2.5401  0.4015    -6.3270
## 3|4 -2.1108  0.4002    -5.2737
## 4|5 -0.4682  0.3985    -1.1751
## 
## Residual Deviance: 7394.743 
## AIC: 7410.743 
## (208 observations deleted due to missingness)
```

```
ctable_inc5 <- coef(summary(inc5))
p_inc5 <- pnorm(abs(ctable_inc5[, "t value"]), lower.tail = FALSE) * 2
(ctable_inc5 <- cbind(ctable_inc5, "p-value" =  round(p_inc5, 4)))
```

```
##                      Value  Std. Error    t value p-value
## income_cat_T3 -0.100654373 0.024015509 -4.1912238  0.0000
## age_T3         0.001421442 0.006100199  0.2330157  0.8157
## sex_T3         0.028989546 0.096531556  0.3003116  0.7639
## bmi_T3         0.002592536 0.006467351  0.4008652  0.6885
## 1|2           -3.947271217 0.413423266 -9.5477723  0.0000
## 2|3           -2.540146480 0.401474381 -6.3270450  0.0000
## 3|4           -2.110752110 0.400241491 -5.2736964  0.0000
## 4|5           -0.468245191 0.398462399 -1.1751302  0.2399
```

```
(ci_inc5 <- confint(inc5, level=0.99375))
```

```
## Waiting for profiling to be done...
```

```
##                     0.3 %      99.7 %
## income_cat_T3 -0.16647361 -0.03509944
## age_T3        -0.01524000  0.01813133
## sex_T3        -0.23706078  0.29121611
## bmi_T3        -0.01499495  0.02039925
```

```
exp(cbind(OR = coef(inc5), ci_inc5))
```

```
##                      OR     0.3 %    99.7 %
## income_cat_T3 0.9042455 0.8466452 0.9655094
## age_T3        1.0014225 0.9848755 1.0182967
## sex_T3        1.0294138 0.7889433 1.3380537
## bmi_T3        1.0025959 0.9851169 1.0206087
```

```
inc6 <- polr(foodst_06_T3 ~ income_cat_T3 + age_T3 + sex_T3 + bmi_T3, data = subset, Hess=TRUE)
summary(inc6)
```

```
## Call:
## polr(formula = foodst_06_T3 ~ income_cat_T3 + age_T3 + sex_T3 + 
##     bmi_T3, data = subset, Hess = TRUE)
## 
## Coefficients:
##                   Value Std. Error t value
## income_cat_T3  0.170066   0.023002  7.3935
## age_T3        -0.023367   0.005777 -4.0450
## sex_T3        -0.331224   0.090488 -3.6604
## bmi_T3        -0.005518   0.006144 -0.8981
## 
## Intercepts:
##     Value   Std. Error t value
## 1|2 -1.5531  0.3779    -4.1099
## 2|3  0.2666  0.3770     0.7071
## 3|4  0.7439  0.3776     1.9698
## 4|5  2.4761  0.3888     6.3681
## 
## Residual Deviance: 8580.854 
## AIC: 8596.854 
## (208 observations deleted due to missingness)
```

```
ctable_inc6 <- coef(summary(inc6))
p_inc6 <- pnorm(abs(ctable_inc6[, "t value"]), lower.tail = FALSE) * 2
(ctable_inc6 <- cbind(ctable_inc6, "p-value" =  round(p_inc6, 4)))
```

```
##                      Value  Std. Error    t value p-value
## income_cat_T3  0.170066128 0.023002260  7.3934530  0.0000
## age_T3        -0.023366656 0.005776639 -4.0450264  0.0001
## sex_T3        -0.331224384 0.090487570 -3.6604407  0.0003
## bmi_T3        -0.005517753 0.006144074 -0.8980609  0.3692
## 1|2           -1.553138712 0.377900729 -4.1099119  0.0000
## 2|3            0.266569391 0.377010755  0.7070604  0.4795
## 3|4            0.743862068 0.377637178  1.9697798  0.0489
## 4|5            2.476092904 0.388830282  6.3680557  0.0000
```

```
(ci_inc6 <- confint(inc6, level=0.99375))
```

```
## Waiting for profiling to be done...
```

```
##                     0.3 %       99.7 %
## income_cat_T3  0.10730403  0.233126006
## age_T3        -0.03920266 -0.007604275
## sex_T3        -0.57846281 -0.083418241
## bmi_T3        -0.02237209  0.011244504
```

```
exp(cbind(OR = coef(inc6), ci_inc6))
```

```
##                      OR     0.3 %    99.7 %
## income_cat_T3 1.1853832 1.1132727 1.2625406
## age_T3        0.9769042 0.9615558 0.9924246
## sex_T3        0.7180440 0.5607597 0.9199663
## bmi_T3        0.9944974 0.9778763 1.0113080
```

```
inc7 <- polr(foodst_07_T3 ~ income_cat_T3 + age_T3 + sex_T3 + bmi_T3, data = subset, Hess=TRUE)
summary(inc7)
```

```
## Call:
## polr(formula = foodst_07_T3 ~ income_cat_T3 + age_T3 + sex_T3 + 
##     bmi_T3, data = subset, Hess = TRUE)
## 
## Coefficients:
##                  Value Std. Error t value
## income_cat_T3  0.08652   0.024725   3.499
## age_T3        -0.02153   0.006235  -3.452
## sex_T3        -0.30587   0.095801  -3.193
## bmi_T3         0.01639   0.006541   2.506
## 
## Intercepts:
##     Value   Std. Error t value
## 1|2 -0.2855  0.4045    -0.7057
## 2|3  1.3543  0.4058     3.3373
## 3|4  1.8892  0.4077     4.6338
## 4|5  3.7390  0.4330     8.6347
## 
## Residual Deviance: 6961.806 
## AIC: 6977.806 
## (208 observations deleted due to missingness)
```

```
ctable_inc7 <- coef(summary(inc7))
p_inc7 <- pnorm(abs(ctable_inc7[, "t value"]), lower.tail = FALSE) * 2
(ctable_inc7 <- cbind(ctable_inc7, "p-value" =  round(p_inc7, 4)))
```

```
##                     Value  Std. Error    t value p-value
## income_cat_T3  0.08651568 0.024724966  3.4991223  0.0005
## age_T3        -0.02152608 0.006235175 -3.4523619  0.0006
## sex_T3        -0.30587229 0.095800910 -3.1927911  0.0014
## bmi_T3         0.01639281 0.006541040  2.5061466  0.0122
## 1|2           -0.28548891 0.404524431 -0.7057396  0.4804
## 2|3            1.35434761 0.405825535  3.3372656  0.0008
## 3|4            1.88923741 0.407710060  4.6337768  0.0000
## 4|5            3.73904655 0.433023246  8.6347479  0.0000
```

```
(ci_inc7 <- confint(inc7, level=0.99375))
```

```
## Waiting for profiling to be done...
```

```
##                      0.3 %       99.7 %
## income_cat_T3  0.019019589  0.154278734
## age_T3        -0.038647622 -0.004535784
## sex_T3        -0.566517319 -0.042252762
## bmi_T3        -0.001596579  0.034197819
```

```
exp(cbind(OR = coef(inc7), ci_inc7))
```

```
##                      OR     0.3 %    99.7 %
## income_cat_T3 1.0903685 1.0192016 1.1668161
## age_T3        0.9787040 0.9620897 0.9954745
## sex_T3        0.7364807 0.5674984 0.9586274
## bmi_T3        1.0165279 0.9984047 1.0347893
```

```
inc8 <- polr(foodst_08_T3 ~ income_cat_T3 + age_T3 + sex_T3 + bmi_T3, data = subset, Hess=TRUE)
summary(inc8)
```

```
## Call:
## polr(formula = foodst_08_T3 ~ income_cat_T3 + age_T3 + sex_T3 + 
##     bmi_T3, data = subset, Hess = TRUE)
## 
## Coefficients:
##                  Value Std. Error t value
## income_cat_T3 -0.05702   0.022136 -2.5761
## age_T3        -0.00140   0.005532 -0.2532
## sex_T3         0.32031   0.088115  3.6351
## bmi_T3         0.00983   0.005839  1.6834
## 
## Intercepts:
##     Value   Std. Error t value
## 1|2 -1.2936  0.3644    -3.5503
## 2|3 -0.1456  0.3631    -0.4010
## 3|4  0.2014  0.3631     0.5546
## 4|5  1.9153  0.3646     5.2525
## 
## Residual Deviance: 10381.71 
## AIC: 10397.71 
## (208 observations deleted due to missingness)
```

```
ctable_inc8 <- coef(summary(inc8))
p_inc8 <- pnorm(abs(ctable_inc8[, "t value"]), lower.tail = FALSE) * 2
(ctable_inc8 <- cbind(ctable_inc8, "p-value" =  round(p_inc8, 4)))
```

```
##                      Value  Std. Error    t value p-value
## income_cat_T3 -0.057024998 0.022136041 -2.5761154  0.0100
## age_T3        -0.001400393 0.005531730 -0.2531564  0.8001
## sex_T3         0.320309233 0.088115312  3.6351143  0.0003
## bmi_T3         0.009830070 0.005839466  1.6833851  0.0923
## 1|2           -1.293569729 0.364352545 -3.5503244  0.0004
## 2|3           -0.145611720 0.363094945 -0.4010293  0.6884
## 3|4            0.201369011 0.363092633  0.5545940  0.5792
## 4|5            1.915309823 0.364648343  5.2524846  0.0000
```

```
(ci_inc8 <- confint(inc8, level=0.99375))
```

```
## Waiting for profiling to be done...
```

```
##                      0.3 %      99.7 %
## income_cat_T3 -0.117596672 0.003480945
## age_T3        -0.016524339 0.013734062
## sex_T3         0.079261689 0.561302986
## bmi_T3        -0.006124005 0.025822014
```

```
exp(cbind(OR = coef(inc8), ci_inc8))
```

```
##                      OR     0.3 %   99.7 %
## income_cat_T3 0.9445705 0.8890546 1.003487
## age_T3        0.9986006 0.9836114 1.013829
## sex_T3        1.3775537 1.0824876 1.752955
## bmi_T3        1.0098785 0.9938947 1.026158
```

```
# path a
# Migration background -> Consumer attitudes

mig1 <- polr(foodst_01_T3 ~ migration + age_T3 + sex_T3 + bmi_T3, data = subset, Hess=TRUE)
summary(mig1)
```

```
## Call:
## polr(formula = foodst_01_T3 ~ migration + age_T3 + sex_T3 + bmi_T3, 
##     data = subset, Hess = TRUE)
## 
## Coefficients:
##              Value Std. Error t value
## migration  0.14225   0.087590  1.6241
## age_T3     0.01067   0.005382  1.9816
## sex_T3     0.05783   0.086063  0.6719
## bmi_T3    -0.01279   0.005578 -2.2930
## 
## Intercepts:
##     Value   Std. Error t value
## 1|2 -1.7860  0.3575    -4.9954
## 2|3 -0.6633  0.3554    -1.8660
## 3|4 -0.0585  0.3551    -0.1649
## 4|5  1.6364  0.3562     4.5934
## 
## Residual Deviance: 11122.62 
## AIC: 11138.62
```

```
ctable_mig1 <- coef(summary(mig1))
p_mig1 <- pnorm(abs(ctable_mig1[, "t value"]), lower.tail = FALSE) * 2
(ctable_mig1 <- cbind(ctable_mig1, "p-value" =  round(p_mig1, 4)))
```

```
##                 Value  Std. Error    t value p-value
## migration  0.14225437 0.087589535  1.6241024  0.1044
## age_T3     0.01066567 0.005382312  1.9816150  0.0475
## sex_T3     0.05782506 0.086062613  0.6718952  0.5017
## bmi_T3    -0.01278937 0.005577503 -2.2930275  0.0218
## 1|2       -1.78602209 0.357530995 -4.9954329  0.0000
## 2|3       -0.66326085 0.355445093 -1.8660009  0.0620
## 3|4       -0.05854195 0.355058695 -0.1648796  0.8690
## 4|5        1.63639847 0.356246245  4.5934476  0.0000
```

```
(ci_mig1 <- confint(mig1, level=0.99375))
```

```
## Waiting for profiling to be done...
```

```
##                 0.3 %      99.7 %
## migration -0.09693562 0.382239485
## age_T3    -0.00405177 0.025387704
## sex_T3    -0.17777357 0.293022683
## bmi_T3    -0.02803663 0.002476255
```

```
exp(cbind(OR = coef(mig1), ci_mig1))
```

```
##                  OR     0.3 %   99.7 %
## migration 1.1528699 0.9076144 1.465563
## age_T3    1.0107228 0.9959564 1.025713
## sex_T3    1.0595296 0.8371320 1.340473
## bmi_T3    0.9872921 0.9723528 1.002479
```

```
mig2 <- polr(foodst_02_T3 ~ migration + age_T3 + sex_T3 + bmi_T3, data = subset, Hess=TRUE)
summary(mig2)
```

```
## Call:
## polr(formula = foodst_02_T3 ~ migration + age_T3 + sex_T3 + bmi_T3, 
##     data = subset, Hess = TRUE)
## 
## Coefficients:
##               Value Std. Error  t value
## migration  0.143410   0.091075  1.57463
## age_T3    -0.003914   0.005583 -0.70098
## sex_T3    -0.006873   0.089087 -0.07715
## bmi_T3     0.019130   0.005798  3.29926
## 
## Intercepts:
##     Value   Std. Error t value
## 1|2  0.3974  0.3644     1.0906
## 2|3  1.7161  0.3653     4.6976
## 3|4  2.4277  0.3667     6.6195
## 4|5  3.9941  0.3767    10.6035
## 
## Residual Deviance: 9472.062 
## AIC: 9488.062
```

```
ctable_mig2 <- coef(summary(mig2))
p_mig2 <- pnorm(abs(ctable_mig2[, "t value"]), lower.tail = FALSE) * 2
(ctable_mig2 <- cbind(ctable_mig2, "p-value" =  round(p_mig2, 4)))
```

```
##                  Value  Std. Error     t value p-value
## migration  0.143409711 0.091075063  1.57463202  0.1153
## age_T3    -0.003913733 0.005583226 -0.70098052  0.4833
## sex_T3    -0.006872909 0.089087058 -0.07714823  0.9385
## bmi_T3     0.019129851 0.005798227  3.29925863  0.0010
## 1|2        0.397446861 0.364417995  1.09063456  0.2754
## 2|3        1.716052155 0.365300875  4.69764042  0.0000
## 3|4        2.427677925 0.366748059  6.61947041  0.0000
## 4|5        3.994119591 0.376679208 10.60350427  0.0000
```

```
(ci_mig2 <- confint(mig2, level=0.99375))
```

```
## Waiting for profiling to be done...
```

```
##                  0.3 %     99.7 %
## migration -0.106789251 0.39158218
## age_T3    -0.019198993 0.01134175
## sex_T3    -0.249574245 0.23787948
## bmi_T3     0.003254186 0.03497847
```

```
exp(cbind(OR = coef(mig2), ci_mig2))
```

```
##                  OR     0.3 %   99.7 %
## migration 1.1542026 0.8987151 1.479319
## age_T3    0.9960939 0.9809841 1.011406
## sex_T3    0.9931507 0.7791324 1.268556
## bmi_T3    1.0193140 1.0032595 1.035597
```

```
mig3 <- polr(foodst_03_T3 ~ migration + age_T3 + sex_T3 + bmi_T3, data = subset, Hess=TRUE)
summary(mig3)
```

```
## Call:
## polr(formula = foodst_03_T3 ~ migration + age_T3 + sex_T3 + bmi_T3, 
##     data = subset, Hess = TRUE)
## 
## Coefficients:
##              Value Std. Error t value
## migration  0.11329   0.090872   1.247
## age_T3     0.02216   0.005481   4.043
## sex_T3     0.31618   0.087345   3.620
## bmi_T3    -0.01742   0.005709  -3.051
## 
## Intercepts:
##     Value   Std. Error t value
## 1|2 -2.0602  0.3663    -5.6245
## 2|3 -0.7762  0.3599    -2.1565
## 3|4  0.0518  0.3590     0.1442
## 4|5  1.5663  0.3601     4.3503
## 
## Residual Deviance: 9831.888 
## AIC: 9847.888
```

```
ctable_mig3 <- coef(summary(mig3))
p_mig3 <- pnorm(abs(ctable_mig3[, "t value"]), lower.tail = FALSE) * 2
(ctable_mig3 <- cbind(ctable_mig3, "p-value" =  round(p_mig3, 4)))
```

```
##                 Value  Std. Error    t value p-value
## migration  0.11329325 0.090872045  1.2467338  0.2125
## age_T3     0.02216009 0.005481224  4.0429084  0.0001
## sex_T3     0.31617688 0.087344580  3.6198797  0.0003
## bmi_T3    -0.01742061 0.005709251 -3.0512950  0.0023
## 1|2       -2.06017672 0.366288686 -5.6244618  0.0000
## 2|3       -0.77622104 0.359947269 -2.1564854  0.0310
## 3|4        0.05176081 0.359039619  0.1441646  0.8854
## 4|5        1.56634091 0.360050062  4.3503420  0.0000
```

```
(ci_mig3 <- confint(mig3, level=0.99375))
```

```
## Waiting for profiling to be done...
```

```
##                 0.3 %       99.7 %
## migration -0.13436833  0.362863958
## age_T3     0.00719821  0.037179012
## sex_T3     0.07699292  0.554841624
## bmi_T3    -0.03301220 -0.001777988
```

```
exp(cbind(OR = coef(mig3), ci_mig3))
```

```
##                  OR     0.3 %    99.7 %
## migration 1.1199603 0.8742680 1.4374403
## age_T3    1.0224074 1.0072242 1.0378788
## sex_T3    1.3718729 1.0800344 1.7416651
## bmi_T3    0.9827303 0.9675268 0.9982236
```

```
mig4 <- polr(foodst_04_T3 ~ migration + age_T3 + sex_T3 + bmi_T3, data = subset, Hess=TRUE)
summary(mig4)
```

```
## Call:
## polr(formula = foodst_04_T3 ~ migration + age_T3 + sex_T3 + bmi_T3, 
##     data = subset, Hess = TRUE)
## 
## Coefficients:
##               Value Std. Error t value
## migration  0.266473   0.088131  3.0236
## age_T3     0.025747   0.005415  4.7551
## sex_T3     0.077738   0.086345  0.9003
## bmi_T3    -0.007121   0.005605 -1.2704
## 
## Intercepts:
##     Value   Std. Error t value
## 1|2 -1.1091  0.3573    -3.1044
## 2|3  0.1874  0.3549     0.5282
## 3|4  0.8302  0.3549     2.3390
## 4|5  2.3982  0.3571     6.7154
## 
## Residual Deviance: 11036.34 
## AIC: 11052.34
```

```
ctable_mig4 <- coef(summary(mig4))
p_mig4 <- pnorm(abs(ctable_mig4[, "t value"]), lower.tail = FALSE) * 2
(ctable_mig4 <- cbind(ctable_mig4, "p-value" =  round(p_mig4, 4)))
```

```
##                  Value  Std. Error    t value p-value
## migration  0.266472688 0.088130726  3.0236071  0.0025
## age_T3     0.025746608 0.005414580  4.7550516  0.0000
## sex_T3     0.077738455 0.086344989  0.9003239  0.3679
## bmi_T3    -0.007120702 0.005605106 -1.2703955  0.2039
## 1|2       -1.109138035 0.357274323 -3.1044437  0.0019
## 2|3        0.187427534 0.354856922  0.5281778  0.5974
## 3|4        0.830237907 0.354948027  2.3390408  0.0193
## 4|5        2.398240329 0.357127802  6.7153560  0.0000
```

```
(ci_mig4 <- confint(mig4, level=0.99375))
```

```
## Waiting for profiling to be done...
```

```
##                 0.3 %     99.7 %
## migration  0.02582332 0.50797918
## age_T3     0.01095821 0.04057482
## sex_T3    -0.15862396 0.31373006
## bmi_T3    -0.02243710 0.00822682
```

```
exp(cbind(OR = coef(mig4), ci_mig4))
```

```
##                  OR     0.3 %   99.7 %
## migration 1.3053519 1.0261596 1.661929
## age_T3    1.0260809 1.0110185 1.041409
## sex_T3    1.0808399 0.8533172 1.368520
## bmi_T3    0.9929046 0.9778127 1.008261
```

```
mig5 <- polr(foodst_05_T3 ~ migration + age_T3 + sex_T3 + bmi_T3, data = subset, Hess=TRUE)
summary(mig5)
```

```
## Call:
## polr(formula = foodst_05_T3 ~ migration + age_T3 + sex_T3 + bmi_T3, 
##     data = subset, Hess = TRUE)
## 
## Coefficients:
##              Value Std. Error t value
## migration 0.065858   0.097368  0.6764
## age_T3    0.003254   0.005911  0.5504
## sex_T3    0.092398   0.094399  0.9788
## bmi_T3    0.006082   0.006183  0.9836
## 
## Intercepts:
##     Value   Std. Error t value
## 1|2 -3.2923  0.4010    -8.2107
## 2|3 -1.8847  0.3891    -4.8435
## 3|4 -1.4554  0.3879    -3.7521
## 4|5  0.1807  0.3868     0.4671
## 
## Residual Deviance: 7797.598 
## AIC: 7813.598
```

```
ctable_mig5 <- coef(summary(mig5))
p_mig5 <- pnorm(abs(ctable_mig5[, "t value"]), lower.tail = FALSE) * 2
(ctable_mig5 <- cbind(ctable_mig5, "p-value" =  round(p_mig5, 4)))
```

```
##                  Value  Std. Error    t value p-value
## migration  0.065857637 0.097368184  0.6763774  0.4988
## age_T3     0.003253809 0.005911270  0.5504417  0.5820
## sex_T3     0.092397950 0.094399260  0.9787995  0.3277
## bmi_T3     0.006081789 0.006183386  0.9835693  0.3253
## 1|2       -3.292315474 0.400978153 -8.2107104  0.0000
## 2|3       -1.884715341 0.389119839 -4.8435344  0.0000
## 3|4       -1.455363841 0.387878832 -3.7521095  0.0002
## 4|5        0.180667436 0.386786206  0.4670990  0.6404
```

```
(ci_mig5 <- confint(mig5, level=0.99375))
```

```
## Waiting for profiling to be done...
```

```
##                 0.3 %     99.7 %
## migration -0.19805187 0.33491849
## age_T3    -0.01288550 0.01945189
## sex_T3    -0.16779979 0.34881562
## bmi_T3    -0.01072761 0.02311072
```

```
exp(cbind(OR = coef(mig5), ci_mig5))
```

```
##                 OR     0.3 %   99.7 %
## migration 1.068075 0.8203273 1.397826
## age_T3    1.003259 0.9871972 1.019642
## sex_T3    1.096801 0.8455231 1.417388
## bmi_T3    1.006100 0.9893297 1.023380
```

```
mig6 <- polr(foodst_06_T3 ~ migration + age_T3 + sex_T3 + bmi_T3, data = subset, Hess=TRUE)
summary(mig6)
```

```
## Call:
## polr(formula = foodst_06_T3 ~ migration + age_T3 + sex_T3 + bmi_T3, 
##     data = subset, Hess = TRUE)
## 
## Coefficients:
##              Value Std. Error t value
## migration -0.30179   0.093504  -3.228
## age_T3    -0.02317   0.005597  -4.139
## sex_T3    -0.40863   0.088494  -4.618
## bmi_T3    -0.01204   0.005883  -2.047
## 
## Intercepts:
##     Value   Std. Error t value
## 1|2 -2.6793  0.3689    -7.2620
## 2|3 -0.8853  0.3664    -2.4161
## 3|4 -0.4130  0.3670    -1.1254
## 4|5  1.3046  0.3779     3.4522
## 
## Residual Deviance: 9066.911 
## AIC: 9082.911
```

```
ctable_mig6 <- coef(summary(mig6))
p_mig6 <- pnorm(abs(ctable_mig6[, "t value"]), lower.tail = FALSE) * 2
(ctable_mig6 <- cbind(ctable_mig6, "p-value" =  round(p_mig6, 4)))
```

```
##                 Value  Std. Error   t value p-value
## migration -0.30178643 0.093503572 -3.227539  0.0012
## age_T3    -0.02316661 0.005596737 -4.139306  0.0000
## sex_T3    -0.40863070 0.088494012 -4.617608  0.0000
## bmi_T3    -0.01204080 0.005883420 -2.046564  0.0407
## 1|2       -2.67926101 0.368940217 -7.262046  0.0000
## 2|3       -0.88533595 0.366438712 -2.416055  0.0157
## 3|4       -0.41299521 0.366983560 -1.125378  0.2604
## 4|5        1.30461309 0.377904959  3.452225  0.0006
```

```
(ci_mig6 <- confint(mig6, level=0.99375))
```

```
## Waiting for profiling to be done...
```

```
##                 0.3 %       99.7 %
## migration -0.55900873 -0.047324271
## age_T3    -0.03850850 -0.007894863
## sex_T3    -0.65046701 -0.166333509
## bmi_T3    -0.02818922  0.004001486
```

```
exp(cbind(OR = coef(mig6), ci_mig6))
```

```
##                  OR     0.3 %    99.7 %
## migration 0.7394960 0.5717756 0.9537781
## age_T3    0.9770997 0.9622235 0.9921362
## sex_T3    0.6645596 0.5218020 0.8467638
## bmi_T3    0.9880314 0.9722044 1.0040095
```

```
mig7 <- polr(foodst_07_T3 ~ migration + age_T3 + sex_T3 + bmi_T3, data = subset, Hess=TRUE)
summary(mig7)
```

```
## Call:
## polr(formula = foodst_07_T3 ~ migration + age_T3 + sex_T3 + bmi_T3, 
##     data = subset, Hess = TRUE)
## 
## Coefficients:
##              Value Std. Error t value
## migration -0.08936   0.099471 -0.8984
## age_T3    -0.02184   0.006058 -3.6050
## sex_T3    -0.35245   0.093662 -3.7629
## bmi_T3     0.01098   0.006278  1.7488
## 
## Intercepts:
##     Value   Std. Error t value
## 1|2 -0.8807  0.3930    -2.2407
## 2|3  0.7619  0.3937     1.9354
## 3|4  1.2925  0.3955     3.2681
## 4|5  3.1094  0.4195     7.4114
## 
## Residual Deviance: 7341.113 
## AIC: 7357.113
```

```
ctable_mig7 <- coef(summary(mig7))
p_mig7 <- pnorm(abs(ctable_mig7[, "t value"]), lower.tail = FALSE) * 2
(ctable_mig7 <- cbind(ctable_mig7, "p-value" =  round(p_mig7, 4)))
```

```
##                 Value  Std. Error    t value p-value
## migration -0.08936108 0.099471149 -0.8983618  0.3690
## age_T3    -0.02183853 0.006057864 -3.6049895  0.0003
## sex_T3    -0.35244505 0.093662031 -3.7629448  0.0002
## bmi_T3     0.01097808 0.006277536  1.7487879  0.0803
## 1|2       -0.88068641 0.393035571 -2.2407295  0.0250
## 2|3        0.76189348 0.393664833  1.9353862  0.0529
## 3|4        1.29245015 0.395475428  3.2680922  0.0011
## 4|5        3.10941182 0.419542815  7.4114291  0.0000
```

```
(ci_mig7 <- confint(mig7, level=0.99375))
```

```
## Waiting for profiling to be done...
```

```
##                  0.3 %       99.7 %
## migration -0.364824803  0.179701966
## age_T3    -0.038471461 -0.005330629
## sex_T3    -0.607290238 -0.094732346
## bmi_T3    -0.006294427  0.028057384
```

```
exp(cbind(OR = coef(mig7), ci_mig7))
```

```
##                  OR     0.3 %    99.7 %
## migration 0.9145153 0.6943183 1.1968606
## age_T3    0.9783982 0.9622592 0.9946836
## sex_T3    0.7029672 0.5448252 0.9096164
## bmi_T3    1.0110386 0.9937253 1.0284547
```

```
mig8 <- polr(foodst_08_T3 ~ migration + age_T3 + sex_T3 + bmi_T3, data = subset, Hess=TRUE)
summary(mig8)
```

```
## Call:
## polr(formula = foodst_08_T3 ~ migration + age_T3 + sex_T3 + bmi_T3, 
##     data = subset, Hess = TRUE)
## 
## Coefficients:
##               Value Std. Error t value
## migration  0.152613   0.088003  1.7342
## age_T3    -0.001321   0.005378 -0.2456
## sex_T3     0.355322   0.086033  4.1300
## bmi_T3     0.010936   0.005569  1.9635
## 
## Intercepts:
##     Value   Std. Error t value
## 1|2 -0.8128  0.3538    -2.2971
## 2|3  0.3060  0.3528     0.8672
## 3|4  0.6474  0.3530     1.8342
## 4|5  2.3433  0.3554     6.5935
## 
## Residual Deviance: 11011.50 
## AIC: 11027.50
```

```
ctable_mig8 <- coef(summary(mig8))
p_mig8 <- pnorm(abs(ctable_mig8[, "t value"]), lower.tail = FALSE) * 2
(ctable_mig8 <- cbind(ctable_mig8, "p-value" =  round(p_mig8, 4)))
```

```
##                  Value  Std. Error    t value p-value
## migration  0.152612992 0.088002814  1.7341831  0.0829
## age_T3    -0.001321032 0.005377791 -0.2456459  0.8060
## sex_T3     0.355322287 0.086033460  4.1300476  0.0000
## bmi_T3     0.010935765 0.005569417  1.9635388  0.0496
## 1|2       -0.812780250 0.353822828 -2.2971391  0.0216
## 2|3        0.305950385 0.352786852  0.8672386  0.3858
## 3|4        0.647391973 0.352964050  1.8341584  0.0666
## 4|5        2.343317429 0.355396445  6.5935309  0.0000
```

```
(ci_mig8 <- confint(mig8, level=0.99375))
```

```
## Waiting for profiling to be done...
```

```
##                  0.3 %     99.7 %
## migration -0.087839557 0.39360074
## age_T3    -0.016025417 0.01339023
## sex_T3     0.119985038 0.59063568
## bmi_T3    -0.004278438 0.02618946
```

```
exp(cbind(OR = coef(mig8), ci_mig8))
```

```
##                  OR     0.3 %   99.7 %
## migration 1.1648741 0.9159078 1.482309
## age_T3    0.9986798 0.9841023 1.013480
## sex_T3    1.4266404 1.1274800 1.805136
## bmi_T3    1.0109958 0.9957307 1.026535
```

```
# path a
# Unemployment in Household -> Consumer attitudes

une1 <- polr(foodst_01_T3 ~ unemploy + age_T3 + sex_T3 + bmi_T3, data = subset, Hess=TRUE)
summary(une1)
```

```
## Call:
## polr(formula = foodst_01_T3 ~ unemploy + age_T3 + sex_T3 + bmi_T3, 
##     data = subset, Hess = TRUE)
## 
## Coefficients:
##             Value Std. Error t value
## unemploy  0.03701   0.103936  0.3561
## age_T3    0.01073   0.005383  1.9939
## sex_T3    0.05221   0.086038  0.6068
## bmi_T3   -0.01276   0.005599 -2.2780
## 
## Intercepts:
##     Value   Std. Error t value
## 1|2 -1.9124  0.3573    -5.3522
## 2|3 -0.7901  0.3551    -2.2248
## 3|4 -0.1857  0.3547    -0.5236
## 4|5  1.5084  0.3557     4.2408
## 
## Residual Deviance: 11125.14 
## AIC: 11141.14
```

```
ctable_une1 <- coef(summary(une1))
p_une1 <- pnorm(abs(ctable_une1[, "t value"]), lower.tail = FALSE) * 2
(ctable_une1 <- cbind(ctable_une1, "p-value" =  round(p_une1, 4)))
```

```
##                Value  Std. Error    t value p-value
## unemploy  0.03700718 0.103935756  0.3560582  0.7218
## age_T3    0.01073305 0.005382889  1.9939204  0.0462
## sex_T3    0.05220766 0.086037873  0.6067986  0.5440
## bmi_T3   -0.01275549 0.005599469 -2.2779817  0.0227
## 1|2      -1.91244692 0.357317966 -5.3522272  0.0000
## 2|3      -0.79010738 0.355142629 -2.2247608  0.0261
## 3|4      -0.18569258 0.354677431 -0.5235534  0.6006
## 4|5       1.50836036 0.355674984  4.2408390  0.0000
```

```
(ci_une1 <- confint(une1, level=0.99375))
```

```
## Waiting for profiling to be done...
```

```
##                 0.3 %      99.7 %
## unemploy -0.246777102 0.321921670
## age_T3   -0.003984288 0.025458268
## sex_T3   -0.183336660 0.287325557
## bmi_T3   -0.028066607 0.002566376
```

```
exp(cbind(OR = coef(une1), ci_une1))
```

```
##                 OR     0.3 %   99.7 %
## unemploy 1.0377005 0.7813148 1.379777
## age_T3   1.0107909 0.9960236 1.025785
## sex_T3   1.0535945 0.8324878 1.332858
## bmi_T3   0.9873255 0.9723236 1.002570
```

```
une2 <- polr(foodst_02_T3 ~ unemploy + age_T3 + sex_T3 + bmi_T3, data = subset, Hess=TRUE)
summary(une2)
```

```
## Call:
## polr(formula = foodst_02_T3 ~ unemploy + age_T3 + sex_T3 + bmi_T3, 
##     data = subset, Hess = TRUE)
## 
## Coefficients:
##             Value Std. Error t value
## unemploy  0.09266   0.107297  0.8636
## age_T3   -0.00377   0.005583 -0.6752
## sex_T3   -0.01378   0.089090 -0.1547
## bmi_T3    0.01892   0.005819  3.2517
## 
## Intercepts:
##     Value   Std. Error t value
## 1|2  0.3244  0.3644     0.8902
## 2|3  1.6429  0.3653     4.4978
## 3|4  2.3542  0.3667     6.4206
## 4|5  3.9198  0.3764    10.4130
## 
## Residual Deviance: 9473.786 
## AIC: 9489.786
```

```
ctable_une2 <- coef(summary(une2))
p_une2 <- pnorm(abs(ctable_une2[, "t value"]), lower.tail = FALSE) * 2
(ctable_une2 <- cbind(ctable_une2, "p-value" =  round(p_une2, 4)))
```

```
##                 Value  Std. Error    t value p-value
## unemploy  0.092661621 0.107297137  0.8635983  0.3878
## age_T3   -0.003769783 0.005583043 -0.6752201  0.4995
## sex_T3   -0.013784843 0.089090418 -0.1547287  0.8770
## bmi_T3    0.018923199 0.005819495  3.2516907  0.0011
## 1|2       0.324381675 0.364384174  0.8902189  0.3733
## 2|3       1.642917774 0.365274712  4.4977594  0.0000
## 3|4       2.354183941 0.366662227  6.4205794  0.0000
## 4|5       3.919772184 0.376432048 10.4129609  0.0000
```

```
(ci_une2 <- confint(une2, level=0.99375))
```

```
## Waiting for profiling to be done...
```

```
##                 0.3 %     99.7 %
## unemploy -0.202766242 0.38456545
## age_T3   -0.019055053 0.01148472
## sex_T3   -0.256505194 0.23096596
## bmi_T3    0.002989459 0.03482951
```

```
exp(cbind(OR = coef(une2), ci_une2))
```

```
##                 OR     0.3 %   99.7 %
## unemploy 1.0970904 0.8164691 1.468976
## age_T3   0.9962373 0.9811253 1.011551
## sex_T3   0.9863097 0.7737510 1.259816
## bmi_T3   1.0191034 1.0029939 1.035443
```

```
une3 <- polr(foodst_03_T3 ~ unemploy + age_T3 + sex_T3 + bmi_T3, data = subset, Hess=TRUE)
summary(une3)
```

```
## Call:
## polr(formula = foodst_03_T3 ~ unemploy + age_T3 + sex_T3 + bmi_T3, 
##     data = subset, Hess = TRUE)
## 
## Coefficients:
##             Value Std. Error t value
## unemploy  0.18248   0.109480   1.667
## age_T3    0.02231   0.005479   4.072
## sex_T3    0.30745   0.087379   3.519
## bmi_T3   -0.01805   0.005731  -3.150
## 
## Intercepts:
##     Value   Std. Error t value
## 1|2 -2.0165  0.3667    -5.4997
## 2|3 -0.7327  0.3603    -2.0336
## 3|4  0.0951  0.3593     0.2647
## 4|5  1.6100  0.3604     4.4677
## 
## Residual Deviance: 9830.652 
## AIC: 9846.652
```

```
ctable_une3 <- coef(summary(une3))
p_une3 <- pnorm(abs(ctable_une3[, "t value"]), lower.tail = FALSE) * 2
(ctable_une3 <- cbind(ctable_une3, "p-value" =  round(p_une3, 4)))
```

```
##                Value  Std. Error    t value p-value
## unemploy  0.18247568 0.109479650  1.6667544  0.0956
## age_T3    0.02230621 0.005478532  4.0715677  0.0000
## sex_T3    0.30745145 0.087378692  3.5186090  0.0004
## bmi_T3   -0.01805235 0.005731359 -3.1497512  0.0016
## 1|2      -2.01646831 0.366652565 -5.4996706  0.0000
## 2|3      -0.73268747 0.360282576 -2.0336467  0.0420
## 3|4       0.09511216 0.359335000  0.2646894  0.7912
## 4|5       1.61003868 0.360369154  4.4677483  0.0000
```

```
(ci_une3 <- confint(une3, level=0.99375))
```

```
## Waiting for profiling to be done...
```

```
##                 0.3 %       99.7 %
## unemploy -0.115402299  0.483865258
## age_T3    0.007351508  0.037317585
## sex_T3    0.068162618  0.546197121
## bmi_T3   -0.033706013 -0.002351058
```

```
exp(cbind(OR = coef(une3), ci_une3))
```

```
##                 OR     0.3 %    99.7 %
## unemploy 1.2001850 0.8910076 1.6223330
## age_T3   1.0225569 1.0073786 1.0380226
## sex_T3   1.3599548 1.0705394 1.7266742
## bmi_T3   0.9821096 0.9668557 0.9976517
```

```
une4 <- polr(foodst_04_T3 ~ unemploy + age_T3 + sex_T3 + bmi_T3, data = subset, Hess=TRUE)
summary(une4)
```

```
## Call:
## polr(formula = foodst_04_T3 ~ unemploy + age_T3 + sex_T3 + bmi_T3, 
##     data = subset, Hess = TRUE)
## 
## Coefficients:
##              Value Std. Error t value
## unemploy  0.315912   0.105817  2.9855
## age_T3    0.026109   0.005415  4.8214
## sex_T3    0.063729   0.086387  0.7377
## bmi_T3   -0.008177   0.005636 -1.4509
## 
## Intercepts:
##     Value   Std. Error t value
## 1|2 -1.1050  0.3582    -3.0847
## 2|3  0.1916  0.3558     0.5386
## 3|4  0.8340  0.3559     2.3436
## 4|5  2.4015  0.3580     6.7079
## 
## Residual Deviance: 11036.56 
## AIC: 11052.56
```

```
ctable_une4 <- coef(summary(une4))
p_une4 <- pnorm(abs(ctable_une4[, "t value"]), lower.tail = FALSE) * 2
(ctable_une4 <- cbind(ctable_une4, "p-value" =  round(p_une4, 4)))
```

```
##                 Value  Std. Error    t value p-value
## unemploy  0.315911832 0.105816605  2.9854656  0.0028
## age_T3    0.026109294 0.005415325  4.8213715  0.0000
## sex_T3    0.063728606 0.086387261  0.7377084  0.4607
## bmi_T3   -0.008177233 0.005636128 -1.4508601  0.1468
## 1|2      -1.105016186 0.358228858 -3.0846655  0.0020
## 2|3       0.191643369 0.355815067  0.5386039  0.5902
## 3|4       0.834033229 0.355872723  2.3436279  0.0191
## 4|5       2.401486191 0.358009550  6.7078830  0.0000
```

```
(ci_une4 <- confint(une4, level=0.99375))
```

```
## Waiting for profiling to be done...
```

```
##                0.3 %     99.7 %
## unemploy  0.02712398 0.60616972
## age_T3    0.01131797 0.04093855
## sex_T3   -0.17276002 0.29982367
## bmi_T3   -0.02358084 0.00725252
```

```
exp(cbind(OR = coef(une4), ci_une4))
```

```
##                 OR     0.3 %   99.7 %
## unemploy 1.3715093 1.0274952 1.833396
## age_T3   1.0264531 1.0113823 1.041788
## sex_T3   1.0658031 0.8413395 1.349621
## bmi_T3   0.9918561 0.9766950 1.007279
```

```
une5 <- polr(foodst_05_T3 ~ unemploy + age_T3 + sex_T3 + bmi_T3, data = subset, Hess=TRUE)
summary(une5)
```

```
## Call:
## polr(formula = foodst_05_T3 ~ unemploy + age_T3 + sex_T3 + bmi_T3, 
##     data = subset, Hess = TRUE)
## 
## Coefficients:
##             Value Std. Error t value
## unemploy 0.387744   0.121785  3.1838
## age_T3   0.003679   0.005919  0.6215
## sex_T3   0.083409   0.094500  0.8826
## bmi_T3   0.004672   0.006208  0.7526
## 
## Intercepts:
##     Value   Std. Error t value
## 1|2 -2.9853  0.4052    -7.3674
## 2|3 -1.5781  0.3935    -4.0101
## 3|4 -1.1488  0.3924    -2.9278
## 4|5  0.4908  0.3916     1.2535
## 
## Residual Deviance: 7787.503 
## AIC: 7803.503
```

```
ctable_une5 <- coef(summary(une5))
p_une5 <- pnorm(abs(ctable_une5[, "t value"]), lower.tail = FALSE) * 2
(ctable_une5 <- cbind(ctable_une5, "p-value" =  round(p_une5, 4)))
```

```
##                 Value  Std. Error    t value p-value
## unemploy  0.387743851 0.121785323  3.1838307  0.0015
## age_T3    0.003678732 0.005919414  0.6214689  0.5343
## sex_T3    0.083409119 0.094499852  0.8826376  0.3774
## bmi_T3    0.004671696 0.006207585  0.7525787  0.4517
## 1|2      -2.985285355 0.405201986 -7.3674006  0.0000
## 2|3      -1.578132865 0.393538806 -4.0101074  0.0001
## 3|4      -1.148764020 0.392365537 -2.9277903  0.0034
## 4|5       0.490815664 0.391561436  1.2534832  0.2100
```

```
(ci_une5 <- confint(une5, level=0.99375))
```

```
## Waiting for profiling to be done...
```

```
##                0.3 %     99.7 %
## unemploy  0.06044451 0.72767444
## age_T3   -0.01249169 0.01989003
## sex_T3   -0.17715211 0.34000784
## bmi_T3   -0.01220143 0.02176921
```

```
exp(cbind(OR = coef(une5), ci_une5))
```

```
##                OR     0.3 %   99.7 %
## unemploy 1.473652 1.0623087 2.070260
## age_T3   1.003686 0.9875860 1.020089
## sex_T3   1.086986 0.8376524 1.404959
## bmi_T3   1.004683 0.9878727 1.022008
```

```
une6 <- polr(foodst_06_T3 ~ unemploy + age_T3 + sex_T3 + bmi_T3, data = subset, Hess=TRUE)
summary(une6)
```

```
## Call:
## polr(formula = foodst_06_T3 ~ unemploy + age_T3 + sex_T3 + bmi_T3, 
##     data = subset, Hess = TRUE)
## 
## Coefficients:
##             Value Std. Error t value
## unemploy -0.46702   0.113874  -4.101
## age_T3   -0.02359   0.005602  -4.211
## sex_T3   -0.39443   0.088553  -4.454
## bmi_T3   -0.01086   0.005904  -1.840
## 
## Intercepts:
##     Value   Std. Error t value
## 1|2 -2.8066  0.3723    -7.5395
## 2|3 -1.0102  0.3696    -2.7332
## 3|4 -0.5378  0.3701    -1.4529
## 4|5  1.1797  0.3810     3.0968
## 
## Residual Deviance: 9060.253 
## AIC: 9076.253
```

```
ctable_une6 <- coef(summary(une6))
p_une6 <- pnorm(abs(ctable_une6[, "t value"]), lower.tail = FALSE) * 2
(ctable_une6 <- cbind(ctable_une6, "p-value" =  round(p_une6, 4)))
```

```
##                Value  Std. Error   t value p-value
## unemploy -0.46701545 0.113873831 -4.101166  0.0000
## age_T3   -0.02359312 0.005602386 -4.211263  0.0000
## sex_T3   -0.39443384 0.088552502 -4.454237  0.0000
## bmi_T3   -0.01086392 0.005904052 -1.840078  0.0658
## 1|2      -2.80664980 0.372258144 -7.539526  0.0000
## 2|3      -1.01020484 0.369609586 -2.733167  0.0063
## 3|4      -0.53775566 0.370135583 -1.452861  0.1463
## 4|5       1.17972892 0.380956872  3.096752  0.0020
```

```
(ci_une6 <- confint(une6, level=0.99375))
```

```
## Waiting for profiling to be done...
```

```
##                0.3 %       99.7 %
## unemploy -0.78154630 -0.158045665
## age_T3   -0.03894996 -0.008305295
## sex_T3   -0.63642245 -0.151968958
## bmi_T3   -0.02706731  0.005235633
```

```
exp(cbind(OR = coef(une6), ci_une6))
```

```
##                 OR     0.3 %    99.7 %
## unemploy 0.6268704 0.4576977 0.8538108
## age_T3   0.9766830 0.9617988 0.9917291
## sex_T3   0.6740616 0.5291822 0.8590149
## bmi_T3   0.9891949 0.9732957 1.0052494
```

```
une7 <- polr(foodst_07_T3 ~ unemploy + age_T3 + sex_T3 + bmi_T3, data = subset, Hess=TRUE)
summary(une7)
```

```
## Call:
## polr(formula = foodst_07_T3 ~ unemploy + age_T3 + sex_T3 + bmi_T3, 
##     data = subset, Hess = TRUE)
## 
## Coefficients:
##             Value Std. Error t value
## unemploy -0.33640   0.123615  -2.721
## age_T3   -0.02213   0.006063  -3.650
## sex_T3   -0.34250   0.093718  -3.655
## bmi_T3    0.01240   0.006310   1.966
## 
## Intercepts:
##     Value   Std. Error t value
## 1|2 -1.1009  0.3958    -2.7815
## 2|3  0.5437  0.3963     1.3721
## 3|4  1.0744  0.3981     2.6990
## 4|5  2.8912  0.4220     6.8516
## 
## Residual Deviance: 7334.24 
## AIC: 7350.24
```

```
ctable_une7 <- coef(summary(une7))
p_une7 <- pnorm(abs(ctable_une7[, "t value"]), lower.tail = FALSE) * 2
(ctable_une7 <- cbind(ctable_une7, "p-value" =  round(p_une7, 4)))
```

```
##                Value  Std. Error   t value p-value
## unemploy -0.33640093 0.123614921 -2.721362  0.0065
## age_T3   -0.02212881 0.006062540 -3.650089  0.0003
## sex_T3   -0.34250455 0.093717698 -3.654641  0.0003
## bmi_T3    0.01240285 0.006309748  1.965664  0.0493
## 1|2      -1.10093668 0.395807872 -2.781493  0.0054
## 2|3       0.54370261 0.396269729  1.372052  0.1700
## 3|4       1.07435726 0.398058075  2.698996  0.0070
## 4|5       2.89121893 0.421975745  6.851623  0.0000
```

```
(ci_une7 <- confint(une7, level=0.99375))
```

```
## Waiting for profiling to be done...
```

```
##                 0.3 %       99.7 %
## unemploy -0.681808938 -0.004537173
## age_T3   -0.038774800 -0.005608209
## sex_T3   -0.597490627 -0.084629391
## bmi_T3   -0.004956371  0.029571043
```

```
exp(cbind(OR = coef(une7), ci_une7))
```

```
##                 OR     0.3 %    99.7 %
## unemploy 0.7143366 0.5057014 0.9954731
## age_T3   0.9781142 0.9619673 0.9944075
## sex_T3   0.7099899 0.5501905 0.9188528
## bmi_T3   1.0124801 0.9950559 1.0300126
```

```
une8 <- polr(foodst_08_T3 ~ unemploy + age_T3 + sex_T3 + bmi_T3, data = subset, Hess=TRUE)
summary(une8)
```

```
## Call:
## polr(formula = foodst_08_T3 ~ unemploy + age_T3 + sex_T3 + bmi_T3, 
##     data = subset, Hess = TRUE)
## 
## Coefficients:
##              Value Std. Error  t value
## unemploy  0.001331   0.106241  0.01253
## age_T3   -0.001317   0.005378 -0.24493
## sex_T3    0.351523   0.085982  4.08831
## bmi_T3    0.011215   0.005587  2.00724
## 
## Intercepts:
##     Value   Std. Error t value
## 1|2 -0.9823  0.3546    -2.7702
## 2|3  0.1362  0.3535     0.3854
## 3|4  0.4773  0.3536     1.3499
## 4|5  2.1714  0.3557     6.1048
## 
## Residual Deviance: 11014.51 
## AIC: 11030.51
```

```
ctable_une8 <- coef(summary(une8))
p_une8 <- pnorm(abs(ctable_une8[, "t value"]), lower.tail = FALSE) * 2
(ctable_une8 <- cbind(ctable_une8, "p-value" =  round(p_une8, 4)))
```

```
##                 Value  Std. Error    t value p-value
## unemploy  0.001330807 0.106241031  0.0125263  0.9900
## age_T3   -0.001317175 0.005377669 -0.2449342  0.8065
## sex_T3    0.351522598 0.085982442  4.0883067  0.0000
## bmi_T3    0.011215123 0.005587341  2.0072379  0.0447
## 1|2      -0.982296638 0.354589500 -2.7702361  0.0056
## 2|3       0.136238424 0.353462025  0.3854401  0.6999
## 3|4       0.477269765 0.353566022  1.3498745  0.1771
## 4|5       2.171367121 0.355682205  6.1047955  0.0000
```

```
(ci_une8 <- confint(une8, level=0.99375))
```

```
## Waiting for profiling to be done...
```

```
##                 0.3 %     99.7 %
## unemploy -0.288962994 0.29236508
## age_T3   -0.016021329 0.01339375
## sex_T3    0.116316538 0.58668873
## bmi_T3   -0.004050391 0.02651554
```

```
exp(cbind(OR = coef(une8), ci_une8))
```

```
##                 OR     0.3 %   99.7 %
## unemploy 1.0013317 0.7490399 1.339592
## age_T3   0.9986837 0.9841063 1.013484
## sex_T3   1.4212299 1.1233514 1.798025
## bmi_T3   1.0112782 0.9959578 1.026870
```

```
# path a
# Single parenthood -> Consumer attitudes

sin1 <- polr(foodst_01_T3 ~ singlepar + age_T3 + sex_T3 + bmi_T3, data = subset, Hess=TRUE)
summary(sin1)
```

```
## Call:
## polr(formula = foodst_01_T3 ~ singlepar + age_T3 + sex_T3 + bmi_T3, 
##     data = subset, Hess = TRUE)
## 
## Coefficients:
##              Value Std. Error t value
## singlepar -0.39825   0.097522 -4.0837
## age_T3     0.01104   0.005381  2.0524
## sex_T3     0.08168   0.086286  0.9467
## bmi_T3    -0.01320   0.005587 -2.3623
## 
## Intercepts:
##     Value   Std. Error t value
## 1|2 -2.3412  0.3570    -6.5572
## 2|3 -1.2175  0.3546    -3.4335
## 3|4 -0.6108  0.3540    -1.7257
## 4|5  1.0886  0.3545     3.0712
## 
## Residual Deviance: 11104.86 
## AIC: 11120.86 
## (1 observation deleted due to missingness)
```

```
ctable_sin1 <- coef(summary(sin1))
p_sin1 <- pnorm(abs(ctable_sin1[, "t value"]), lower.tail = FALSE) * 2
(ctable_sin1 <- cbind(ctable_sin1, "p-value" =  round(p_sin1, 4)))
```

```
##                 Value  Std. Error    t value p-value
## singlepar -0.39825003 0.097521739 -4.0837051  0.0000
## age_T3     0.01104385 0.005380999  2.0523786  0.0401
## sex_T3     0.08168469 0.086285581  0.9466784  0.3438
## bmi_T3    -0.01319926 0.005587476 -2.3622939  0.0182
## 1|2       -2.34121135 0.357045785 -6.5571740  0.0000
## 2|3       -1.21750677 0.354597852 -3.4334860  0.0006
## 3|4       -0.61084100 0.353960800 -1.7257306  0.0844
## 4|5        1.08858980 0.354456137  3.0711552  0.0021
```

```
(ci_sin1 <- confint(sin1, level=0.99375))
```

```
## Waiting for profiling to be done...
```

```
##                  0.3 %       99.7 %
## singlepar -0.664935431 -0.131357712
## age_T3    -0.003666897  0.025765270
## sex_T3    -0.154528202  0.317487302
## bmi_T3    -0.028479767  0.002087691
```

```
exp(cbind(OR = coef(sin1), ci_sin1))
```

```
##                  OR     0.3 %   99.7 %
## singlepar 0.6714941 0.5143067 0.876904
## age_T3    1.0111051 0.9963398 1.026100
## sex_T3    1.0851136 0.8568193 1.373672
## bmi_T3    0.9868875 0.9719220 1.002090
```

```
sin2 <- polr(foodst_02_T3 ~ singlepar + age_T3 + sex_T3 + bmi_T3, data = subset, Hess=TRUE)
summary(sin2)
```

```
## Call:
## polr(formula = foodst_02_T3 ~ singlepar + age_T3 + sex_T3 + bmi_T3, 
##     data = subset, Hess = TRUE)
## 
## Coefficients:
##               Value Std. Error  t value
## singlepar -0.065951   0.100045 -0.65921
## age_T3    -0.003781   0.005586 -0.67697
## sex_T3    -0.007921   0.089289 -0.08871
## bmi_T3     0.019324   0.005800  3.33169
## 
## Intercepts:
##     Value   Std. Error t value
## 1|2  0.1720  0.3629     0.4740
## 2|3  1.4910  0.3637     4.1001
## 3|4  2.2008  0.3650     6.0305
## 4|5  3.7666  0.3747    10.0529
## 
## Residual Deviance: 9469.397 
## AIC: 9485.397 
## (1 observation deleted due to missingness)
```

```
ctable_sin2 <- coef(summary(sin2))
p_sin2 <- pnorm(abs(ctable_sin2[, "t value"]), lower.tail = FALSE) * 2
(ctable_sin2 <- cbind(ctable_sin2, "p-value" =  round(p_sin2, 4)))
```

```
##                  Value  Std. Error     t value p-value
## singlepar -0.065950635 0.100045060 -0.65920931  0.5098
## age_T3    -0.003781274 0.005585562 -0.67697277  0.4984
## sex_T3    -0.007920863 0.089288629 -0.08871077  0.9293
## bmi_T3     0.019324264 0.005800140  3.33168920  0.0009
## 1|2        0.172004408 0.362894570  0.47397901  0.6355
## 2|3        1.491041583 0.363658183  4.10011833  0.0000
## 3|4        2.200835363 0.364951974  6.03047941  0.0000
## 4|5        3.766608279 0.374677263 10.05294062  0.0000
```

```
(ci_sin2 <- confint(sin2, level=0.99375))
```

```
## Waiting for profiling to be done...
```

```
##                  0.3 %     99.7 %
## singlepar -0.341625913 0.20596425
## age_T3    -0.019073128 0.01148042
## sex_T3    -0.251184564 0.23736988
## bmi_T3     0.003443435 0.03517791
```

```
exp(cbind(OR = coef(sin2), ci_sin2))
```

```
##                  OR     0.3 %   99.7 %
## singlepar 0.9361771 0.7106140 1.228709
## age_T3    0.9962259 0.9811076 1.011547
## sex_T3    0.9921104 0.7778788 1.267910
## bmi_T3    1.0195122 1.0034494 1.035804
```

```
sin3 <- polr(foodst_03_T3 ~ singlepar + age_T3 + sex_T3 + bmi_T3, data = subset, Hess=TRUE)
summary(sin3)
```

```
## Call:
## polr(formula = foodst_03_T3 ~ singlepar + age_T3 + sex_T3 + bmi_T3, 
##     data = subset, Hess = TRUE)
## 
## Coefficients:
##              Value Std. Error t value
## singlepar -0.45144   0.099659  -4.530
## age_T3     0.02286   0.005485   4.167
## sex_T3     0.34396   0.087613   3.926
## bmi_T3    -0.01810   0.005718  -3.166
## 
## Intercepts:
##     Value   Std. Error t value
## 1|2 -2.6313  0.3661    -7.1874
## 2|3 -1.3470  0.3596    -3.7463
## 3|4 -0.5148  0.3584    -1.4365
## 4|5  1.0062  0.3586     2.8057
## 
## Residual Deviance: 9807.962 
## AIC: 9823.962 
## (1 observation deleted due to missingness)
```

```
ctable_sin3 <- coef(summary(sin3))
p_sin3 <- pnorm(abs(ctable_sin3[, "t value"]), lower.tail = FALSE) * 2
(ctable_sin3 <- cbind(ctable_sin3, "p-value" =  round(p_sin3, 4)))
```

```
##                 Value  Std. Error   t value p-value
## singlepar -0.45144450 0.099658811 -4.529901  0.0000
## age_T3     0.02285686 0.005484776  4.167328  0.0000
## sex_T3     0.34395521 0.087612523  3.925868  0.0001
## bmi_T3    -0.01810334 0.005717691 -3.166198  0.0015
## 1|2       -2.63128993 0.366098095 -7.187390  0.0000
## 2|3       -1.34699776 0.359555875 -3.746282  0.0002
## 3|4       -0.51478126 0.358358772 -1.436497  0.1509
## 4|5        1.00616944 0.358611994  2.805733  0.0050
```

```
(ci_sin3 <- confint(sin3, level=0.99375))
```

```
## Waiting for profiling to be done...
```

```
##                  0.3 %       99.7 %
## singlepar -0.723661727 -0.178345425
## age_T3     0.007885957  0.037886132
## sex_T3     0.104048990  0.583361378
## bmi_T3    -0.033719590 -0.002439417
```

```
exp(cbind(OR = coef(sin3), ci_sin3))
```

```
##                  OR     0.3 %    99.7 %
## singlepar 0.6367078 0.4849732 0.8366534
## age_T3    1.0231201 1.0079171 1.0386130
## sex_T3    1.4105155 1.1096548 1.7920521
## bmi_T3    0.9820595 0.9668426 0.9975636
```

```
sin4 <- polr(foodst_04_T3 ~ singlepar + age_T3 + sex_T3 + bmi_T3, data = subset, Hess=TRUE)
summary(sin4)
```

```
## Call:
## polr(formula = foodst_04_T3 ~ singlepar + age_T3 + sex_T3 + bmi_T3, 
##     data = subset, Hess = TRUE)
## 
## Coefficients:
##               Value Std. Error t value
## singlepar -0.520571   0.096617  -5.388
## age_T3     0.026586   0.005417   4.908
## sex_T3     0.104919   0.086533   1.212
## bmi_T3    -0.007879   0.005614  -1.403
## 
## Intercepts:
##     Value   Std. Error t value
## 1|2 -1.9269  0.3575    -5.3906
## 2|3 -0.6259  0.3545    -1.7655
## 3|4  0.0193  0.3542     0.0546
## 4|5  1.5914  0.3553     4.4790
## 
## Residual Deviance: 11014.61 
## AIC: 11030.61 
## (1 observation deleted due to missingness)
```

```
ctable_sin4 <- coef(summary(sin4))
p_sin4 <- pnorm(abs(ctable_sin4[, "t value"]), lower.tail = FALSE) * 2
(ctable_sin4 <- cbind(ctable_sin4, "p-value" =  round(p_sin4, 4)))
```

```
##                  Value  Std. Error     t value p-value
## singlepar -0.520571118 0.096617363 -5.38796654  0.0000
## age_T3     0.026586074 0.005417061  4.90784073  0.0000
## sex_T3     0.104918871 0.086533115  1.21247075  0.2253
## bmi_T3    -0.007878871 0.005614494 -1.40330919  0.1605
## 1|2       -1.926879018 0.357452616 -5.39058586  0.0000
## 2|3       -0.625941662 0.354541855 -1.76549441  0.0775
## 3|4        0.019345643 0.354205244  0.05461704  0.9564
## 4|5        1.591413713 0.355303014  4.47903240  0.0000
```

```
(ci_sin4 <- confint(sin4, level=0.99375))
```

```
## Waiting for profiling to be done...
```

```
##                 0.3 %       99.7 %
## singlepar -0.78485130 -0.256216873
## age_T3     0.01178968  0.041419715
## sex_T3    -0.13195501  0.341426182
## bmi_T3    -0.02322169  0.007493114
```

```
exp(cbind(OR = coef(sin4), ci_sin4))
```

```
##                  OR     0.3 %    99.7 %
## singlepar 0.5941811 0.4561875 0.7739741
## age_T3    1.0269426 1.0118595 1.0422895
## sex_T3    1.1106205 0.8763804 1.4069527
## bmi_T3    0.9921521 0.9770459 1.0075213
```

```
sin5 <- polr(foodst_05_T3 ~ singlepar + age_T3 + sex_T3 + bmi_T3, data = subset, Hess=TRUE)
summary(sin5)
```

```
## Call:
## polr(formula = foodst_05_T3 ~ singlepar + age_T3 + sex_T3 + bmi_T3, 
##     data = subset, Hess = TRUE)
## 
## Coefficients:
##               Value Std. Error t value
## singlepar -0.403369   0.104407 -3.8634
## age_T3     0.003895   0.005914  0.6586
## sex_T3     0.116150   0.094704  1.2264
## bmi_T3     0.005623   0.006183  0.9093
## 
## Intercepts:
##     Value   Std. Error t value
## 1|2 -3.7601  0.4004    -9.3908
## 2|3 -2.3507  0.3884    -6.0520
## 3|4 -1.9201  0.3871    -4.9601
## 4|5 -0.2789  0.3854    -0.7237
## 
## Residual Deviance: 7782.388 
## AIC: 7798.388 
## (1 observation deleted due to missingness)
```

```
ctable_sin5 <- coef(summary(sin5))
p_sin5 <- pnorm(abs(ctable_sin5[, "t value"]), lower.tail = FALSE) * 2
(ctable_sin5 <- cbind(ctable_sin5, "p-value" =  round(p_sin5, 4)))
```

```
##                  Value  Std. Error    t value p-value
## singlepar -0.403369368 0.104406959 -3.8634338  0.0001
## age_T3     0.003894729 0.005913564  0.6586095  0.5101
## sex_T3     0.116149847 0.094704331  1.2264470  0.2200
## bmi_T3     0.005622534 0.006183089  0.9093407  0.3632
## 1|2       -3.760147384 0.400409626 -9.3907517  0.0000
## 2|3       -2.350726121 0.388419731 -6.0520255  0.0000
## 3|4       -1.920128884 0.387115824 -4.9600889  0.0000
## 4|5       -0.278926990 0.385444318 -0.7236505  0.4693
```

```
(ci_sin5 <- confint(sin5, level=0.99375))
```

```
## Waiting for profiling to be done...
```

```
##                 0.3 %      99.7 %
## singlepar -0.68716174 -0.11564650
## age_T3    -0.01225901  0.02009068
## sex_T3    -0.14476671  0.37350557
## bmi_T3    -0.01119294  0.02264379
```

```
exp(cbind(OR = coef(sin5), ci_sin5))
```

```
##                  OR     0.3 %    99.7 %
## singlepar 0.6680653 0.5030017 0.8907901
## age_T3    1.0039023 0.9878158 1.0202939
## sex_T3    1.1231642 0.8652241 1.4528187
## bmi_T3    1.0056384 0.9888695 1.0229021
```

```
sin6 <- polr(foodst_06_T3 ~ singlepar + age_T3 + sex_T3 + bmi_T3, data = subset, Hess=TRUE)
summary(sin6)
```

```
## Call:
## polr(formula = foodst_06_T3 ~ singlepar + age_T3 + sex_T3 + bmi_T3, 
##     data = subset, Hess = TRUE)
## 
## Coefficients:
##              Value Std. Error t value
## singlepar  0.57108   0.099518   5.739
## age_T3    -0.02414   0.005598  -4.313
## sex_T3    -0.44212   0.088867  -4.975
## bmi_T3    -0.01176   0.005866  -2.004
## 
## Intercepts:
##     Value   Std. Error t value
## 1|2 -1.8063  0.3662    -4.9327
## 2|3 -0.0025  0.3651    -0.0069
## 3|4  0.4702  0.3658     1.2853
## 4|5  2.1911  0.3770     5.8119
## 
## Residual Deviance: 9039.094 
## AIC: 9055.094 
## (1 observation deleted due to missingness)
```

```
ctable_sin6 <- coef(summary(sin6))
p_sin6 <- pnorm(abs(ctable_sin6[, "t value"]), lower.tail = FALSE) * 2
(ctable_sin6 <- cbind(ctable_sin6, "p-value" =  round(p_sin6, 4)))
```

```
##                  Value  Std. Error      t value p-value
## singlepar  0.571084266 0.099517860  5.738510328  0.0000
## age_T3    -0.024144202 0.005598470 -4.312642812  0.0000
## sex_T3    -0.442123989 0.088867447 -4.975095005  0.0000
## bmi_T3    -0.011756578 0.005866112 -2.004151666  0.0451
## 1|2       -1.806283734 0.366184288 -4.932717741  0.0000
## 2|3       -0.002519133 0.365071553 -0.006900382  0.9945
## 3|4        0.470150678 0.365790297  1.285301118  0.1987
## 4|5        2.191072186 0.376995512  5.811931755  0.0000
```

```
(ci_sin6 <- confint(sin6, level=0.99375))
```

```
## Waiting for profiling to be done...
```

```
##                 0.3 %       99.7 %
## singlepar  0.29863923  0.843153297
## age_T3    -0.03948982 -0.008866610
## sex_T3    -0.68500723 -0.198832097
## bmi_T3    -0.02786254  0.004233229
```

```
exp(cbind(OR = coef(sin6), ci_sin6))
```

```
##                  OR     0.3 %    99.7 %
## singlepar 1.7701854 1.3480232 2.3236827
## age_T3    0.9761449 0.9612797 0.9911726
## sex_T3    0.6426699 0.5040866 0.8196875
## bmi_T3    0.9883123 0.9725220 1.0042422
```

```
sin7 <- polr(foodst_07_T3 ~ singlepar + age_T3 + sex_T3 + bmi_T3, data = subset, Hess=TRUE)
summary(sin7)
```

```
## Call:
## polr(formula = foodst_07_T3 ~ singlepar + age_T3 + sex_T3 + bmi_T3, 
##     data = subset, Hess = TRUE)
## 
## Coefficients:
##              Value Std. Error t value
## singlepar  0.29069   0.106488   2.730
## age_T3    -0.02242   0.006060  -3.700
## sex_T3    -0.37123   0.094019  -3.949
## bmi_T3     0.01150   0.006277   1.833
## 
## Intercepts:
##     Value   Std. Error t value
## 1|2 -0.5032  0.3911    -1.2865
## 2|3  1.1431  0.3922     2.9146
## 3|4  1.6756  0.3941     4.2519
## 4|5  3.4887  0.4182     8.3416
## 
## Residual Deviance: 7328.548 
## AIC: 7344.548 
## (1 observation deleted due to missingness)
```

```
ctable_sin7 <- coef(summary(sin7))
p_sin7 <- pnorm(abs(ctable_sin7[, "t value"]), lower.tail = FALSE) * 2
(ctable_sin7 <- cbind(ctable_sin7, "p-value" =  round(p_sin7, 4)))
```

```
##                 Value  Std. Error   t value p-value
## singlepar  0.29068524 0.106488019  2.729746  0.0063
## age_T3    -0.02242343 0.006060010 -3.700230  0.0002
## sex_T3    -0.37123436 0.094019031 -3.948502  0.0001
## bmi_T3     0.01150410 0.006276543  1.832871  0.0668
## 1|2       -0.50317875 0.391131256 -1.286470  0.1983
## 2|3        1.14313430 0.392214919  2.914561  0.0036
## 3|4        1.67557189 0.394075412  4.251907  0.0000
## 4|5        3.48874411 0.418236559  8.341557  0.0000
```

```
(ci_sin7 <- confint(sin7, level=0.99375))
```

```
## Waiting for profiling to be done...
```

```
##                  0.3 %       99.7 %
## singlepar -0.003457421  0.579529295
## age_T3    -0.039063515 -0.005910811
## sex_T3    -0.627091824 -0.112581007
## bmi_T3    -0.005765970  0.028580420
```

```
exp(cbind(OR = coef(sin7), ci_sin7))
```

```
##                  OR     0.3 %    99.7 %
## singlepar 1.3373436 0.9965485 1.7851979
## age_T3    0.9778261 0.9616896 0.9941066
## sex_T3    0.6898822 0.5341429 0.8935250
## bmi_T3    1.0115705 0.9942506 1.0289928
```

```
sin8 <- polr(foodst_08_T3 ~ singlepar + age_T3 + sex_T3 + bmi_T3, data = subset, Hess=TRUE)
summary(sin8)
```

```
## Call:
## polr(formula = foodst_08_T3 ~ singlepar + age_T3 + sex_T3 + bmi_T3, 
##     data = subset, Hess = TRUE)
## 
## Coefficients:
##               Value Std. Error t value
## singlepar  0.170496   0.098500  1.7309
## age_T3    -0.001526   0.005381 -0.2836
## sex_T3     0.340349   0.086189  3.9489
## bmi_T3     0.011571   0.005574  2.0760
## 
## Intercepts:
##     Value   Std. Error t value
## 1|2 -0.8164  0.3534    -2.3101
## 2|3  0.3026  0.3524     0.8587
## 3|4  0.6437  0.3525     1.8261
## 4|5  2.3379  0.3548     6.5899
## 
## Residual Deviance: 11009.60 
## AIC: 11025.60 
## (1 observation deleted due to missingness)
```

```
ctable_sin8 <- coef(summary(sin8))
p_sin8 <- pnorm(abs(ctable_sin8[, "t value"]), lower.tail = FALSE) * 2
(ctable_sin8 <- cbind(ctable_sin8, "p-value" =  round(p_sin8, 4)))
```

```
##                  Value  Std. Error    t value p-value
## singlepar  0.170495523 0.098499879  1.7309212  0.0835
## age_T3    -0.001526053 0.005380920 -0.2836045  0.7767
## sex_T3     0.340349220 0.086188519  3.9488928  0.0001
## bmi_T3     0.011571068 0.005573648  2.0760315  0.0379
## 1|2       -0.816362017 0.353390734 -2.3100833  0.0209
## 2|3        0.302563576 0.352369784  0.8586536  0.3905
## 3|4        0.643673739 0.352486173  1.8260964  0.0678
## 4|5        2.337860211 0.354761730  6.5899448  0.0000
```

```
(ci_sin8 <- confint(sin8, level=0.99375))
```

```
## Waiting for profiling to be done...
```

```
##                  0.3 %     99.7 %
## singlepar -0.098349153 0.44057052
## age_T3    -0.016239857 0.01319293
## sex_T3     0.104585092 0.57608235
## bmi_T3    -0.003655103 0.02683596
```

```
exp(cbind(OR = coef(sin8), ci_sin8))
```

```
##                  OR     0.3 %   99.7 %
## singlepar 1.1858923 0.9063324 1.553593
## age_T3    0.9984751 0.9838913 1.013280
## sex_T3    1.4054383 1.1102499 1.779055
## bmi_T3    1.0116383 0.9963516 1.027199
```

### 4.1.3 Path b: Consumer attitudes -> HDAS

```
subset$foodst_01_T3 <- as.numeric(subset$foodst_01_T3)
subset$foodst_02_T3 <- as.numeric(subset$foodst_02_T3)
subset$foodst_03_T3 <- as.numeric(subset$foodst_03_T3)
subset$foodst_04_T3 <- as.numeric(subset$foodst_04_T3)
subset$foodst_05_T3 <- as.numeric(subset$foodst_05_T3)
subset$foodst_06_T3 <- as.numeric(subset$foodst_06_T3)
subset$foodst_07_T3 <- as.numeric(subset$foodst_07_T3)
subset$foodst_08_T3 <- as.numeric(subset$foodst_08_T3)
```

```
# path b
# Consumer attitudes -> HDAS 

reg1 <- lm(hds_T3 ~ foodst_01_T3 + age_T3 + sex_T3 + bmi_T3, data = subset)
summary(reg1)
```

```
## 
## Call:
## lm(formula = hds_T3 ~ foodst_01_T3 + age_T3 + sex_T3 + bmi_T3, 
##     data = subset)
## 
## Residuals:
##      Min       1Q   Median       3Q      Max 
## -24.9212  -5.9251   0.2444   6.2289  22.8926 
## 
## Coefficients:
##              Estimate Std. Error t value Pr(>|t|)    
## (Intercept)  13.93866    1.67784   8.307  < 2e-16 ***
## foodst_01_T3  1.19573    0.11177  10.698  < 2e-16 ***
## age_T3        0.13976    0.02583   5.412 6.64e-08 ***
## sex_T3        1.14778    0.41526   2.764  0.00574 ** 
## bmi_T3       -0.02549    0.02709  -0.941  0.34681    
## ---
## Signif. codes:  0 '***' 0.001 '**' 0.01 '*' 0.05 '.' 0.1 ' ' 1
## 
## Residual standard error: 8.698 on 3720 degrees of freedom
## Multiple R-squared:  0.03893,    Adjusted R-squared:  0.0379 
## F-statistic: 37.68 on 4 and 3720 DF,  p-value: < 2.2e-16
```

```
confint(reg1, level=0.99375)
```

```
##                  0.312 %    99.688 %
## (Intercept)   9.34820879 18.52910907
## foodst_01_T3  0.88993977  1.50152597
## age_T3        0.06910105  0.21041989
## sex_T3        0.01166354  2.28389821
## bmi_T3       -0.09961339  0.04863098
```

```
reg2 <- lm(hds_T3 ~ foodst_02_T3 + age_T3 + sex_T3 + bmi_T3, data = subset)
summary(reg2)
```

```
## 
## Call:
## lm(formula = hds_T3 ~ foodst_02_T3 + age_T3 + sex_T3 + bmi_T3, 
##     data = subset)
## 
## Residuals:
##      Min       1Q   Median       3Q      Max 
## -24.5355  -6.0166   0.2925   6.3629  25.1775 
## 
## Coefficients:
##              Estimate Std. Error t value Pr(>|t|)    
## (Intercept)  19.35137    1.66383  11.631  < 2e-16 ***
## foodst_02_T3 -0.84334    0.13055  -6.460 1.18e-10 ***
## age_T3        0.14588    0.02607   5.596 2.35e-08 ***
## sex_T3        1.17753    0.41924   2.809    0.005 ** 
## bmi_T3       -0.02457    0.02739  -0.897    0.370    
## ---
## Signif. codes:  0 '***' 0.001 '**' 0.01 '*' 0.05 '.' 0.1 ' ' 1
## 
## Residual standard error: 8.782 on 3720 degrees of freedom
## Multiple R-squared:  0.02036,    Adjusted R-squared:  0.0193 
## F-statistic: 19.32 on 4 and 3720 DF,  p-value: 9.48e-16
```

```
confint(reg2, level=0.99375)
```

```
##                  0.312 %    99.688 %
## (Intercept)  14.79925946 23.90347496
## foodst_02_T3 -1.20051569 -0.48616954
## age_T3        0.07455782  0.21719591
## sex_T3        0.03051884  2.32453244
## bmi_T3       -0.09950607  0.05035761
```

```
reg3 <- lm(hds_T3 ~ foodst_03_T3 + age_T3 + sex_T3 + bmi_T3, data = subset)
summary(reg3)
```

```
## 
## Call:
## lm(formula = hds_T3 ~ foodst_03_T3 + age_T3 + sex_T3 + bmi_T3, 
##     data = subset)
## 
## Residuals:
##      Min       1Q   Median       3Q      Max 
## -26.3961  -6.0264   0.2004   6.2839  24.2117 
## 
## Coefficients:
##              Estimate Std. Error t value Pr(>|t|)    
## (Intercept)  14.32492    1.70279   8.413  < 2e-16 ***
## foodst_03_T3  1.08251    0.13062   8.287  < 2e-16 ***
## age_T3        0.13238    0.02603   5.086 3.84e-07 ***
## sex_T3        0.96909    0.41859   2.315   0.0207 *  
## bmi_T3       -0.02296    0.02728  -0.842   0.4000    
## ---
## Signif. codes:  0 '***' 0.001 '**' 0.01 '*' 0.05 '.' 0.1 ' ' 1
## 
## Residual standard error: 8.75 on 3720 degrees of freedom
## Multiple R-squared:  0.02732,    Adjusted R-squared:  0.02628 
## F-statistic: 26.12 on 4 and 3720 DF,  p-value: < 2.2e-16
```

```
confint(reg3, level=0.99375)
```

```
##                  0.312 %    99.688 %
## (Intercept)   9.66622798 18.98362173
## foodst_03_T3  0.72513319  1.43988832
## age_T3        0.06116827  0.20359715
## sex_T3       -0.17613749  2.11431820
## bmi_T3       -0.09759953  0.05167655
```

```
reg4 <- lm(hds_T3 ~ foodst_04_T3 + age_T3 + sex_T3 + bmi_T3, data = subset)
summary(reg4)
```

```
## 
## Call:
## lm(formula = hds_T3 ~ foodst_04_T3 + age_T3 + sex_T3 + bmi_T3, 
##     data = subset)
## 
## Residuals:
##      Min       1Q   Median       3Q      Max 
## -25.1415  -6.0146   0.3127   6.3840  24.4674 
## 
## Coefficients:
##              Estimate Std. Error t value Pr(>|t|)    
## (Intercept)  15.18600    1.67653   9.058  < 2e-16 ***
## foodst_04_T3  0.97311    0.11465   8.487  < 2e-16 ***
## age_T3        0.13002    0.02603   4.994 6.18e-07 ***
## sex_T3        1.13010    0.41762   2.706  0.00684 ** 
## bmi_T3       -0.03035    0.02723  -1.114  0.26518    
## ---
## Signif. codes:  0 '***' 0.001 '**' 0.01 '*' 0.05 '.' 0.1 ' ' 1
## 
## Residual standard error: 8.746 on 3720 degrees of freedom
## Multiple R-squared:  0.02818,    Adjusted R-squared:  0.02714 
## F-statistic: 26.97 on 4 and 3720 DF,  p-value: < 2.2e-16
```

```
confint(reg4, level=0.99375)
```

```
##                  0.312 %    99.688 %
## (Intercept)  10.59913418 19.77285585
## foodst_04_T3  0.65942895  1.28680080
## age_T3        0.05879596  0.20125246
## sex_T3       -0.01246348  2.27266552
## bmi_T3       -0.10485899  0.04416061
```

```
reg5 <- lm(hds_T3 ~ foodst_05_T3 + age_T3 + sex_T3 + bmi_T3, data = subset)
summary(reg5)
```

```
## 
## Call:
## lm(formula = hds_T3 ~ foodst_05_T3 + age_T3 + sex_T3 + bmi_T3, 
##     data = subset)
## 
## Residuals:
##     Min      1Q  Median      3Q     Max 
## -24.537  -6.083   0.270   6.378  23.177 
## 
## Coefficients:
##              Estimate Std. Error t value Pr(>|t|)    
## (Intercept)  15.95155    1.77341   8.995  < 2e-16 ***
## foodst_05_T3  0.48456    0.15032   3.223  0.00128 ** 
## age_T3        0.14614    0.02618   5.583 2.53e-08 ***
## sex_T3        1.16103    0.42109   2.757  0.00586 ** 
## bmi_T3       -0.03612    0.02745  -1.316  0.18837    
## ---
## Signif. codes:  0 '***' 0.001 '**' 0.01 '*' 0.05 '.' 0.1 ' ' 1
## 
## Residual standard error: 8.818 on 3720 degrees of freedom
## Multiple R-squared:  0.01213,    Adjusted R-squared:  0.01106 
## F-statistic: 11.41 on 4 and 3720 DF,  p-value: 3.292e-09
```

```
confint(reg5, level=0.99375)
```

```
##                   0.312 %    99.688 %
## (Intercept)  11.099636578 20.80346511
## foodst_05_T3  0.073285799  0.89582467
## age_T3        0.074526793  0.21776270
## sex_T3        0.008965655  2.31308761
## bmi_T3       -0.111225009  0.03898994
```

```
reg6 <- lm(hds_T3 ~ foodst_06_T3 + age_T3 + sex_T3 + bmi_T3, data = subset)
summary(reg6)
```

```
## 
## Call:
## lm(formula = hds_T3 ~ foodst_06_T3 + age_T3 + sex_T3 + bmi_T3, 
##     data = subset)
## 
## Residuals:
##      Min       1Q   Median       3Q      Max 
## -25.8900  -6.0522   0.1795   6.3574  23.1628 
## 
## Coefficients:
##              Estimate Std. Error t value Pr(>|t|)    
## (Intercept)  19.60374    1.70435  11.502  < 2e-16 ***
## foodst_06_T3 -0.55399    0.13806  -4.013 6.12e-05 ***
## age_T3        0.14005    0.02620   5.345 9.60e-08 ***
## sex_T3        1.06887    0.42175   2.534   0.0113 *  
## bmi_T3       -0.03817    0.02744  -1.391   0.1643    
## ---
## Signif. codes:  0 '***' 0.001 '**' 0.01 '*' 0.05 '.' 0.1 ' ' 1
## 
## Residual standard error: 8.812 on 3720 degrees of freedom
## Multiple R-squared:  0.01364,    Adjusted R-squared:  0.01257 
## F-statistic: 12.86 on 4 and 3720 DF,  p-value: 2.141e-10
```

```
confint(reg6, level=0.99375)
```

```
##                  0.312 %    99.688 %
## (Intercept)  14.94074983 24.26673193
## foodst_06_T3 -0.93170271 -0.17627624
## age_T3        0.06835824  0.21174294
## sex_T3       -0.08499784  2.22274000
## bmi_T3       -0.11323655  0.03690599
```

```
reg7 <- lm(hds_T3 ~ foodst_07_T3 + age_T3 + sex_T3 + bmi_T3, data = subset)
summary(reg7)
```

```
## 
## Call:
## lm(formula = hds_T3 ~ foodst_07_T3 + age_T3 + sex_T3 + bmi_T3, 
##     data = subset)
## 
## Residuals:
##      Min       1Q   Median       3Q      Max 
## -25.8325  -6.0728   0.3457   6.2775  23.8623 
## 
## Coefficients:
##              Estimate Std. Error t value Pr(>|t|)    
## (Intercept)  19.74511    1.68659  11.707  < 2e-16 ***
## foodst_07_T3 -0.86040    0.16288  -5.283 1.35e-07 ***
## age_T3        0.13824    0.02616   5.284 1.33e-07 ***
## sex_T3        1.07237    0.42059   2.550   0.0108 *  
## bmi_T3       -0.02999    0.02741  -1.094   0.2739    
## ---
## Signif. codes:  0 '***' 0.001 '**' 0.01 '*' 0.05 '.' 0.1 ' ' 1
## 
## Residual standard error: 8.798 on 3720 degrees of freedom
## Multiple R-squared:  0.01674,    Adjusted R-squared:  0.01568 
## F-statistic: 15.83 on 4 and 3720 DF,  p-value: 7.392e-13
```

```
confint(reg7, level=0.99375)
```

```
##                  0.312 %    99.688 %
## (Intercept)  15.13073562 24.35948834
## foodst_07_T3 -1.30601504 -0.41477923
## age_T3        0.06666782  0.20981571
## sex_T3       -0.07834581  2.22308521
## bmi_T3       -0.10497465  0.04498852
```

```
reg8 <- lm(hds_T3 ~ foodst_08_T3 + age_T3 + sex_T3 + bmi_T3, data = subset)
summary(reg8)
```

```
## 
## Call:
## lm(formula = hds_T3 ~ foodst_08_T3 + age_T3 + sex_T3 + bmi_T3, 
##     data = subset)
## 
## Residuals:
##      Min       1Q   Median       3Q      Max 
## -26.0725  -6.0653   0.3154   6.3930  22.5974 
## 
## Coefficients:
##              Estimate Std. Error t value Pr(>|t|)    
## (Intercept)  16.75870    1.68098   9.970  < 2e-16 ***
## foodst_08_T3  0.44964    0.10654   4.220 2.50e-05 ***
## age_T3        0.14722    0.02615   5.629 1.94e-08 ***
## sex_T3        1.07052    0.42152   2.540   0.0111 *  
## bmi_T3       -0.03924    0.02744  -1.430   0.1528    
## ---
## Signif. codes:  0 '***' 0.001 '**' 0.01 '*' 0.05 '.' 0.1 ' ' 1
## 
## Residual standard error: 8.81 on 3720 degrees of freedom
## Multiple R-squared:  0.01409,    Adjusted R-squared:  0.01303 
## F-statistic: 13.29 on 4 and 3720 DF,  p-value: 9.434e-11
```

```
confint(reg8, level=0.99375)
```

```
##                  0.312 %    99.688 %
## (Intercept)  12.15966708 21.35773947
## foodst_08_T3  0.15815180  0.74112046
## age_T3        0.07567061  0.21876805
## sex_T3       -0.08272724  2.22377599
## bmi_T3       -0.11431447  0.03582908
```

### 5.1.4 Path c: Socioeconomic factors -> HDAS (Total effect)

```
# Education -> HDAS 
edu_total <- lm(hds_T3 ~ isced_cat2011_T3 + age_T3 + sex_T3 + bmi_T3, data = subset)
summary(edu_total)
```

```
## 
## Call:
## lm(formula = hds_T3 ~ isced_cat2011_T3 + age_T3 + sex_T3 + bmi_T3, 
##     data = subset)
## 
## Residuals:
##      Min       1Q   Median       3Q      Max 
## -26.2289  -5.9992   0.2969   6.2489  23.4079 
## 
## Coefficients:
##                  Estimate Std. Error t value Pr(>|t|)    
## (Intercept)       9.74141    1.81855   5.357 9.01e-08 ***
## isced_cat2011_T3  2.93866    0.25291  11.620  < 2e-16 ***
## age_T3            0.11323    0.02633   4.300 1.75e-05 ***
## sex_T3            1.57587    0.42543   3.704 0.000215 ***
## bmi_T3            0.03052    0.02787   1.095 0.273561    
## ---
## Signif. codes:  0 '***' 0.001 '**' 0.01 '*' 0.05 '.' 0.1 ' ' 1
## 
## Residual standard error: 8.669 on 3605 degrees of freedom
##   (115 observations deleted due to missingness)
## Multiple R-squared:  0.04447,    Adjusted R-squared:  0.04341 
## F-statistic: 41.95 on 4 and 3605 DF,  p-value: < 2.2e-16
```

```
confint(edu_total, level=0.99375)
```

```
##                      0.312 %   99.688 %
## (Intercept)       4.76590546 14.7169110
## isced_cat2011_T3  2.24671962  3.6306080
## age_T3            0.04118358  0.1852853
## sex_T3            0.41191943  2.7398300
## bmi_T3           -0.04573917  0.1067873
```

```
# Income -> HDAS
inc_total <- lm(hds_T3 ~ income_cat_T3 + age_T3 + sex_T3 + bmi_T3, data = subset)
summary(inc_total)
```

```
## 
## Call:
## lm(formula = hds_T3 ~ income_cat_T3 + age_T3 + sex_T3 + bmi_T3, 
##     data = subset)
## 
## Residuals:
##      Min       1Q   Median       3Q      Max 
## -25.2770  -5.9623   0.2383   6.2759  24.4759 
## 
## Coefficients:
##                Estimate Std. Error t value Pr(>|t|)    
## (Intercept)   13.496084   1.751118   7.707 1.66e-14 ***
## income_cat_T3  1.018351   0.106146   9.594  < 2e-16 ***
## age_T3         0.139841   0.026700   5.237 1.72e-07 ***
## sex_T3         1.607880   0.426090   3.774 0.000164 ***
## bmi_T3         0.004805   0.028471   0.169 0.865991    
## ---
## Signif. codes:  0 '***' 0.001 '**' 0.01 '*' 0.05 '.' 0.1 ' ' 1
## 
## Residual standard error: 8.729 on 3512 degrees of freedom
##   (208 observations deleted due to missingness)
## Multiple R-squared:  0.03515,    Adjusted R-squared:  0.03405 
## F-statistic: 31.99 on 4 and 3512 DF,  p-value: < 2.2e-16
```

```
confint(inc_total, level=0.99375)
```

```
##                   0.312 %    99.688 %
## (Intercept)    8.70499194 18.28717595
## income_cat_T3  0.72793419  1.30876727
## age_T3         0.06678826  0.21289322
## sex_T3         0.44208922  2.77367053
## bmi_T3        -0.07309216  0.08270197
```

```
# Migration background -> HDAS
mig_total <- lm(hds_T3 ~ migration + age_T3 + sex_T3 + bmi_T3, data = subset)
summary(mig_total)
```

```
## 
## Call:
## lm(formula = hds_T3 ~ migration + age_T3 + sex_T3 + bmi_T3, data = subset)
## 
## Residuals:
##      Min       1Q   Median       3Q      Max 
## -25.4316  -6.0393   0.2648   6.3942  22.8526 
## 
## Coefficients:
##             Estimate Std. Error t value Pr(>|t|)    
## (Intercept) 18.43309    1.72390  10.693  < 2e-16 ***
## migration   -0.40839    0.42700  -0.956  0.33892    
## age_T3       0.14668    0.02621   5.596 2.36e-08 ***
## sex_T3       1.18010    0.42165   2.799  0.00516 ** 
## bmi_T3      -0.03461    0.02750  -1.259  0.20824    
## ---
## Signif. codes:  0 '***' 0.001 '**' 0.01 '*' 0.05 '.' 0.1 ' ' 1
## 
## Residual standard error: 8.83 on 3720 degrees of freedom
## Multiple R-squared:  0.00961,    Adjusted R-squared:  0.008545 
## F-statistic: 9.024 on 4 and 3720 DF,  p-value: 2.991e-07
```

```
confint(mig_total, level=0.99375)
```

```
##                 0.312 %    99.688 %
## (Intercept) 13.71663059 23.14955210
## migration   -1.57662389  0.75984447
## age_T3       0.07496093  0.21838956
## sex_T3       0.02650089  2.33369772
## bmi_T3      -0.10984526  0.04062334
```

```
# Unemployment in Household -> HDAS
une_total <- lm(hds_T3 ~ unemploy + age_T3 + sex_T3 + bmi_T3, data = subset)
summary(une_total)
```

```
## 
## Call:
## lm(formula = hds_T3 ~ unemploy + age_T3 + sex_T3 + bmi_T3, data = subset)
## 
## Residuals:
##      Min       1Q   Median       3Q      Max 
## -25.5230  -6.0019   0.2671   6.3495  22.6487 
## 
## Coefficients:
##             Estimate Std. Error t value Pr(>|t|)    
## (Intercept) 19.96283    1.72643  11.563  < 2e-16 ***
## unemploy    -2.04773    0.50587  -4.048 5.27e-05 ***
## age_T3       0.14456    0.02616   5.526 3.50e-08 ***
## sex_T3       1.23793    0.42082   2.942  0.00328 ** 
## bmi_T3      -0.02616    0.02752  -0.950  0.34201    
## ---
## Signif. codes:  0 '***' 0.001 '**' 0.01 '*' 0.05 '.' 0.1 ' ' 1
## 
## Residual standard error: 8.811 on 3720 degrees of freedom
## Multiple R-squared:  0.01371,    Adjusted R-squared:  0.01265 
## F-statistic: 12.93 on 4 and 3720 DF,  p-value: 1.869e-10
```

```
confint(une_total, level=0.99375)
```

```
##                 0.312 %    99.688 %
## (Intercept) 15.23943207 24.68622614
## unemploy    -3.43175973 -0.66369488
## age_T3       0.07299188  0.21613309
## sex_T3       0.08659327  2.38927280
## bmi_T3      -0.10146119  0.04914724
```

```
# Single parenthood -> HDAS
sin_total <- lm(hds_T3 ~ singlepar + age_T3 + sex_T3 + bmi_T3, data = subset)
summary(sin_total)
```

```
## 
## Call:
## lm(formula = hds_T3 ~ singlepar + age_T3 + sex_T3 + bmi_T3, data = subset)
## 
## Residuals:
##      Min       1Q   Median       3Q      Max 
## -25.4025  -6.0357   0.2519   6.3964  22.8090 
## 
## Coefficients:
##             Estimate Std. Error t value Pr(>|t|)    
## (Intercept) 18.14382    1.72343  10.528  < 2e-16 ***
## singlepar   -0.16379    0.47928  -0.342  0.73257    
## age_T3       0.14691    0.02621   5.606 2.22e-08 ***
## sex_T3       1.20606    0.42239   2.855  0.00432 ** 
## bmi_T3      -0.03634    0.02749  -1.322  0.18624    
## ---
## Signif. codes:  0 '***' 0.001 '**' 0.01 '*' 0.05 '.' 0.1 ' ' 1
## 
## Residual standard error: 8.826 on 3719 degrees of freedom
##   (1 observation deleted due to missingness)
## Multiple R-squared:  0.009473,   Adjusted R-squared:  0.008407 
## F-statistic: 8.891 on 4 and 3719 DF,  p-value: 3.834e-07
```

```
confint(sin_total, level=0.99375)
```

```
##                 0.312 %    99.688 %
## (Intercept) 13.42863787 22.85900094
## singlepar   -1.47506375  1.14748688
## age_T3       0.07520855  0.21860570
## sex_T3       0.05043696  2.36168711
## bmi_T3      -0.11154142  0.03886394
```

```
unloadNamespace("MASS")
```

## 4.2 Structural equation modelling using lavaan package (indirect and direct effect)

### 4.2.1 Highest level of education in household

Controlled for age, sex, BMI

```
SEM_model_edu1 <- '
    level: 1
    hds_T3 ~ isced_cat2011_T3 + a1*foodst_01_T3 + a2*foodst_02_T3 + a3*foodst_03_T3 + a4*foodst_04_T3 + a5*foodst_05_T3 + a6*foodst_06_T3 + a7*foodst_07_T3 + a8*foodst_08_T3 + age_T3 + sex_T3 + bmi_T3
    foodst_01_T3 ~ b1*isced_cat2011_T3
    foodst_02_T3 ~ b2*isced_cat2011_T3
    foodst_03_T3 ~ b3*isced_cat2011_T3
    foodst_04_T3 ~ b4*isced_cat2011_T3
    foodst_05_T3 ~ b5*isced_cat2011_T3
    foodst_06_T3 ~ b6*isced_cat2011_T3
    foodst_07_T3 ~ b7*isced_cat2011_T3
    foodst_08_T3 ~ b8*isced_cat2011_T3
    foodst_01_T3 ~~ foodst_02_T3 + foodst_03_T3 + foodst_04_T3 + foodst_05_T3 + foodst_06_T3 + foodst_07_T3 + foodst_08_T3
    foodst_02_T3 ~~ foodst_03_T3 + foodst_04_T3 + foodst_05_T3 + foodst_06_T3 + foodst_07_T3 + foodst_08_T3
    foodst_03_T3 ~~ foodst_04_T3 + foodst_05_T3 + foodst_06_T3 + foodst_07_T3 + foodst_08_T3
    foodst_04_T3 ~~ foodst_05_T3 + foodst_06_T3 + foodst_07_T3 + foodst_08_T3
    foodst_05_T3 ~~ foodst_06_T3 + foodst_07_T3 + foodst_08_T3
    foodst_06_T3 ~~ foodst_07_T3 + foodst_08_T3 
    foodst_07_T3 ~~ foodst_08_T3
    ebindfoodst_01_T3 := a1*b1 
    ebindfoodst_02_T3 := a2*b2
    ebindfoodst_03_T3 := a3*b3
    ebindfoodst_04_T3 := a4*b4
    ebindfoodst_05_T3 := a5*b5
    ebindfoodst_06_T3 := a6*b6
    ebindfoodst_07_T3 := a7*b7
    ebindfoodst_08_T3 := a8*b8

    level: 2
    hds_T3 ~ 1

'

fit_SEM_edu1 <- sem(model = SEM_model_edu1, data = subset, cluster = "country")

summary(fit_SEM_edu1)
```

```
## lavaan 0.6.16 ended normally after 123 iterations
## 
##   Estimator                                         ML
##   Optimization method                           NLMINB
##   Number of model parameters                        67
## 
##                                                   Used       Total
##   Number of observations                          3610        3725
##   Number of clusters [country]                       6            
## 
## Model Test User Model:
##                                                       
##   Test statistic                                59.777
##   Degrees of freedom                                24
##   P-value (Chi-square)                           0.000
## 
## Parameter Estimates:
## 
##   Standard errors                             Standard
##   Information                                 Observed
##   Observed information based on                Hessian
## 
## 
## Level 1 [within]:
## 
## Regressions:
##                    Estimate  Std.Err  z-value  P(>|z|)
##   hds_T3 ~                                            
##     i_2011_T3         1.603    0.243    6.601    0.000
##     fds_01_T3 (a1)    0.800    0.119    6.706    0.000
##     fds_02_T3 (a2)   -0.599    0.127   -4.707    0.000
##     fds_03_T3 (a3)    0.361    0.156    2.306    0.021
##     fds_04_T3 (a4)    0.615    0.138    4.470    0.000
##     fds_05_T3 (a5)   -0.128    0.160   -0.797    0.425
##     fds_06_T3 (a6)   -0.651    0.147   -4.415    0.000
##     fds_07_T3 (a7)   -0.063    0.172   -0.367    0.714
##     fds_08_T3 (a8)    0.356    0.102    3.503    0.000
##     age_T3            0.094    0.025    3.767    0.000
##     sex_T3            1.480    0.402    3.679    0.000
##     bmi_T3            0.078    0.026    2.959    0.003
##   foodst_01_T3 ~                                      
##     i_2011_T3 (b1)    0.106    0.036    2.928    0.003
##   foodst_02_T3 ~                                      
##     i_2011_T3 (b2)   -0.266    0.031   -8.651    0.000
##   foodst_03_T3 ~                                      
##     i_2011_T3 (b3)    0.120    0.031    3.856    0.000
##   foodst_04_T3 ~                                      
##     i_2011_T3 (b4)    0.082    0.036    2.306    0.021
##   foodst_05_T3 ~                                      
##     i_2011_T3 (b5)   -0.032    0.027   -1.176    0.240
##   foodst_06_T3 ~                                      
##     i_2011_T3 (b6)    0.199    0.030    6.720    0.000
##   foodst_07_T3 ~                                      
##     i_2011_T3 (b7)    0.006    0.025    0.256    0.798
##   foodst_08_T3 ~                                      
##     i_2011_T3 (b8)   -0.017    0.039   -0.451    0.652
## 
## Covariances:
##                    Estimate  Std.Err  z-value  P(>|z|)
##  .foodst_01_T3 ~~                                     
##    .foodst_02_T3      0.198    0.023    8.550    0.000
##    .foodst_03_T3      0.559    0.025   22.325    0.000
##    .foodst_04_T3      0.564    0.028   20.067    0.000
##    .foodst_05_T3      0.202    0.021    9.769    0.000
##    .foodst_06_T3     -0.137    0.022   -6.193    0.000
##    .foodst_07_T3     -0.105    0.019   -5.575    0.000
##    .foodst_08_T3      0.142    0.029    4.934    0.000
##  .foodst_02_T3 ~~                                     
##    .foodst_03_T3      0.078    0.020    3.932    0.000
##    .foodst_04_T3      0.115    0.023    5.048    0.000
##    .foodst_05_T3      0.056    0.017    3.182    0.001
##    .foodst_06_T3      0.064    0.019    3.377    0.001
##    .foodst_07_T3      0.055    0.016    3.422    0.001
##    .foodst_08_T3     -0.042    0.025   -1.695    0.090
##  .foodst_03_T3 ~~                                     
##    .foodst_04_T3      0.749    0.026   28.704    0.000
##    .foodst_05_T3      0.379    0.019   20.193    0.000
##    .foodst_06_T3     -0.257    0.020  -13.134    0.000
##    .foodst_07_T3     -0.200    0.017  -12.074    0.000
##    .foodst_08_T3      0.161    0.025    6.437    0.000
##  .foodst_04_T3 ~~                                     
##    .foodst_05_T3      0.508    0.022   23.278    0.000
##    .foodst_06_T3     -0.251    0.022  -11.335    0.000
##    .foodst_07_T3     -0.206    0.019  -10.956    0.000
##    .foodst_08_T3      0.198    0.028    6.941    0.000
##  .foodst_05_T3 ~~                                     
##    .foodst_06_T3     -0.231    0.017  -13.442    0.000
##    .foodst_07_T3     -0.170    0.015  -11.686    0.000
##    .foodst_08_T3      0.164    0.022    7.483    0.000
##  .foodst_06_T3 ~~                                     
##    .foodst_07_T3      0.397    0.017   23.704    0.000
##    .foodst_08_T3     -0.063    0.024   -2.685    0.007
##  .foodst_07_T3 ~~                                     
##    .foodst_08_T3     -0.032    0.020   -1.573    0.116
## 
## Intercepts:
##                    Estimate  Std.Err  z-value  P(>|z|)
##    .hds_T3            0.000                           
##    .foodst_01_T3      3.202    0.091   35.131    0.000
##    .foodst_02_T3      2.555    0.078   32.855    0.000
##    .foodst_03_T3      3.700    0.079   46.945    0.000
##    .foodst_04_T3      3.343    0.090   37.228    0.000
##    .foodst_05_T3      4.439    0.069   64.154    0.000
##    .foodst_06_T3      1.423    0.075   19.033    0.000
##    .foodst_07_T3      1.550    0.064   24.372    0.000
##    .foodst_08_T3      3.398    0.097   34.917    0.000
## 
## Variances:
##                    Estimate  Std.Err  z-value  P(>|z|)
##    .hds_T3           66.394    1.564   42.450    0.000
##    .foodst_01_T3      1.616    0.038   42.485    0.000
##    .foodst_02_T3      1.177    0.028   42.485    0.000
##    .foodst_03_T3      1.209    0.028   42.485    0.000
##    .foodst_04_T3      1.569    0.037   42.485    0.000
##    .foodst_05_T3      0.931    0.022   42.485    0.000
##    .foodst_06_T3      1.087    0.026   42.485    0.000
##    .foodst_07_T3      0.786    0.019   42.486    0.000
##    .foodst_08_T3      1.843    0.043   42.485    0.000
## 
## 
## Level 2 [country]:
## 
## Intercepts:
##                    Estimate  Std.Err  z-value  P(>|z|)
##    .hds_T3            8.485    2.226    3.812    0.000
## 
## Variances:
##                    Estimate  Std.Err  z-value  P(>|z|)
##    .hds_T3            7.234    4.258    1.699    0.089
## 
## Defined Parameters:
##                    Estimate  Std.Err  z-value  P(>|z|)
##     ebndfdst_01_T3    0.085    0.032    2.683    0.007
##     ebndfdst_02_T3    0.160    0.039    4.135    0.000
##     ebndfdst_03_T3    0.043    0.022    1.979    0.048
##     ebndfdst_04_T3    0.050    0.025    2.049    0.040
##     ebndfdst_05_T3    0.004    0.006    0.660    0.509
##     ebndfdst_06_T3   -0.129    0.035   -3.690    0.000
##     ebndfdst_07_T3   -0.000    0.002   -0.210    0.834
##     ebndfdst_08_T3   -0.006    0.014   -0.447    0.655
```

```
parameterEstimates(fit_SEM_edu1, ci=TRUE, level=.99375)
```

```
##                  lhs op              rhs block level             label    est
## 1             hds_T3  ~ isced_cat2011_T3     1     1                    1.603
## 2             hds_T3  ~     foodst_01_T3     1     1                a1  0.800
## 3             hds_T3  ~     foodst_02_T3     1     1                a2 -0.599
## 4             hds_T3  ~     foodst_03_T3     1     1                a3  0.361
## 5             hds_T3  ~     foodst_04_T3     1     1                a4  0.615
## 6             hds_T3  ~     foodst_05_T3     1     1                a5 -0.128
## 7             hds_T3  ~     foodst_06_T3     1     1                a6 -0.651
## 8             hds_T3  ~     foodst_07_T3     1     1                a7 -0.063
## 9             hds_T3  ~     foodst_08_T3     1     1                a8  0.356
## 10            hds_T3  ~           age_T3     1     1                    0.094
## 11            hds_T3  ~           sex_T3     1     1                    1.480
## 12            hds_T3  ~           bmi_T3     1     1                    0.078
## 13      foodst_01_T3  ~ isced_cat2011_T3     1     1                b1  0.106
## 14      foodst_02_T3  ~ isced_cat2011_T3     1     1                b2 -0.266
## 15      foodst_03_T3  ~ isced_cat2011_T3     1     1                b3  0.120
## 16      foodst_04_T3  ~ isced_cat2011_T3     1     1                b4  0.082
## 17      foodst_05_T3  ~ isced_cat2011_T3     1     1                b5 -0.032
## 18      foodst_06_T3  ~ isced_cat2011_T3     1     1                b6  0.199
## 19      foodst_07_T3  ~ isced_cat2011_T3     1     1                b7  0.006
## 20      foodst_08_T3  ~ isced_cat2011_T3     1     1                b8 -0.017
## 21      foodst_01_T3 ~~     foodst_02_T3     1     1                    0.198
## 22      foodst_01_T3 ~~     foodst_03_T3     1     1                    0.559
## 23      foodst_01_T3 ~~     foodst_04_T3     1     1                    0.564
## 24      foodst_01_T3 ~~     foodst_05_T3     1     1                    0.202
## 25      foodst_01_T3 ~~     foodst_06_T3     1     1                   -0.137
## 26      foodst_01_T3 ~~     foodst_07_T3     1     1                   -0.105
## 27      foodst_01_T3 ~~     foodst_08_T3     1     1                    0.142
## 28      foodst_02_T3 ~~     foodst_03_T3     1     1                    0.078
## 29      foodst_02_T3 ~~     foodst_04_T3     1     1                    0.115
## 30      foodst_02_T3 ~~     foodst_05_T3     1     1                    0.056
## 31      foodst_02_T3 ~~     foodst_06_T3     1     1                    0.064
## 32      foodst_02_T3 ~~     foodst_07_T3     1     1                    0.055
## 33      foodst_02_T3 ~~     foodst_08_T3     1     1                   -0.042
## 34      foodst_03_T3 ~~     foodst_04_T3     1     1                    0.749
## 35      foodst_03_T3 ~~     foodst_05_T3     1     1                    0.379
## 36      foodst_03_T3 ~~     foodst_06_T3     1     1                   -0.257
## 37      foodst_03_T3 ~~     foodst_07_T3     1     1                   -0.200
## 38      foodst_03_T3 ~~     foodst_08_T3     1     1                    0.161
## 39      foodst_04_T3 ~~     foodst_05_T3     1     1                    0.508
## 40      foodst_04_T3 ~~     foodst_06_T3     1     1                   -0.251
## 41      foodst_04_T3 ~~     foodst_07_T3     1     1                   -0.206
## 42      foodst_04_T3 ~~     foodst_08_T3     1     1                    0.198
## 43      foodst_05_T3 ~~     foodst_06_T3     1     1                   -0.231
## 44      foodst_05_T3 ~~     foodst_07_T3     1     1                   -0.170
## 45      foodst_05_T3 ~~     foodst_08_T3     1     1                    0.164
## 46      foodst_06_T3 ~~     foodst_07_T3     1     1                    0.397
## 47      foodst_06_T3 ~~     foodst_08_T3     1     1                   -0.063
## 48      foodst_07_T3 ~~     foodst_08_T3     1     1                   -0.032
## 49            hds_T3 ~~           hds_T3     1     1                   66.394
## 50      foodst_01_T3 ~~     foodst_01_T3     1     1                    1.616
## 51      foodst_02_T3 ~~     foodst_02_T3     1     1                    1.177
## 52      foodst_03_T3 ~~     foodst_03_T3     1     1                    1.209
## 53      foodst_04_T3 ~~     foodst_04_T3     1     1                    1.569
## 54      foodst_05_T3 ~~     foodst_05_T3     1     1                    0.931
## 55      foodst_06_T3 ~~     foodst_06_T3     1     1                    1.087
## 56      foodst_07_T3 ~~     foodst_07_T3     1     1                    0.786
## 57      foodst_08_T3 ~~     foodst_08_T3     1     1                    1.843
## 58  isced_cat2011_T3 ~~ isced_cat2011_T3     1     1                    0.344
## 59  isced_cat2011_T3 ~~           age_T3     1     1                    0.306
## 60  isced_cat2011_T3 ~~           sex_T3     1     1                   -0.017
## 61  isced_cat2011_T3 ~~           bmi_T3     1     1                   -0.589
## 62            age_T3 ~~           age_T3     1     1                   32.026
## 63            age_T3 ~~           sex_T3     1     1                   -0.453
## 64            age_T3 ~~           bmi_T3     1     1                    2.566
## 65            sex_T3 ~~           sex_T3     1     1                    0.124
## 66            sex_T3 ~~           bmi_T3     1     1                   -0.236
## 67            bmi_T3 ~~           bmi_T3     1     1                   28.520
## 68            hds_T3 ~1                      1     1                    0.000
## 69      foodst_01_T3 ~1                      1     1                    3.202
## 70      foodst_02_T3 ~1                      1     1                    2.555
## 71      foodst_03_T3 ~1                      1     1                    3.700
## 72      foodst_04_T3 ~1                      1     1                    3.343
## 73      foodst_05_T3 ~1                      1     1                    4.439
## 74      foodst_06_T3 ~1                      1     1                    1.423
## 75      foodst_07_T3 ~1                      1     1                    1.550
## 76      foodst_08_T3 ~1                      1     1                    3.398
## 77  isced_cat2011_T3 ~1                      1     1                    2.458
## 78            age_T3 ~1                      1     1                   41.760
## 79            sex_T3 ~1                      1     1                    1.855
## 80            bmi_T3 ~1                      1     1                   26.173
## 81            hds_T3 ~1                      2     2                    8.485
## 82            hds_T3 ~~           hds_T3     2     2                    7.234
## 83 ebindfoodst_01_T3 :=            a1*b1     0     0 ebindfoodst_01_T3  0.085
## 84 ebindfoodst_02_T3 :=            a2*b2     0     0 ebindfoodst_02_T3  0.160
## 85 ebindfoodst_03_T3 :=            a3*b3     0     0 ebindfoodst_03_T3  0.043
## 86 ebindfoodst_04_T3 :=            a4*b4     0     0 ebindfoodst_04_T3  0.050
## 87 ebindfoodst_05_T3 :=            a5*b5     0     0 ebindfoodst_05_T3  0.004
## 88 ebindfoodst_06_T3 :=            a6*b6     0     0 ebindfoodst_06_T3 -0.129
## 89 ebindfoodst_07_T3 :=            a7*b7     0     0 ebindfoodst_07_T3  0.000
## 90 ebindfoodst_08_T3 :=            a8*b8     0     0 ebindfoodst_08_T3 -0.006
##       se       z pvalue ci.lower ci.upper
## 1  0.243   6.601  0.000    0.939    2.267
## 2  0.119   6.706  0.000    0.474    1.127
## 3  0.127  -4.707  0.000   -0.947   -0.251
## 4  0.156   2.306  0.021   -0.067    0.788
## 5  0.138   4.470  0.000    0.239    0.991
## 6  0.160  -0.797  0.425   -0.565    0.310
## 7  0.147  -4.415  0.000   -1.054   -0.248
## 8  0.172  -0.367  0.714   -0.533    0.407
## 9  0.102   3.503  0.000    0.078    0.633
## 10 0.025   3.767  0.000    0.026    0.162
## 11 0.402   3.679  0.000    0.380    2.580
## 12 0.026   2.959  0.003    0.006    0.150
## 13 0.036   2.928  0.003    0.007    0.204
## 14 0.031  -8.651  0.000   -0.350   -0.182
## 15 0.031   3.856  0.000    0.035    0.206
## 16 0.036   2.306  0.021   -0.015    0.179
## 17 0.027  -1.176  0.240   -0.107    0.043
## 18 0.030   6.720  0.000    0.118    0.280
## 19 0.025   0.256  0.798   -0.062    0.075
## 20 0.039  -0.451  0.652   -0.123    0.088
## 21 0.023   8.550  0.000    0.135    0.262
## 22 0.025  22.325  0.000    0.491    0.628
## 23 0.028  20.067  0.000    0.487    0.641
## 24 0.021   9.769  0.000    0.146    0.259
## 25 0.022  -6.193  0.000   -0.198   -0.077
## 26 0.019  -5.575  0.000   -0.157   -0.054
## 27 0.029   4.934  0.000    0.063    0.221
## 28 0.020   3.932  0.000    0.024    0.133
## 29 0.023   5.048  0.000    0.052    0.177
## 30 0.017   3.182  0.001    0.008    0.103
## 31 0.019   3.377  0.001    0.012    0.115
## 32 0.016   3.422  0.001    0.011    0.099
## 33 0.025  -1.695  0.090   -0.109    0.025
## 34 0.026  28.704  0.000    0.678    0.820
## 35 0.019  20.193  0.000    0.327    0.430
## 36 0.020 -13.134  0.000   -0.310   -0.203
## 37 0.017 -12.074  0.000   -0.245   -0.155
## 38 0.025   6.437  0.000    0.093    0.229
## 39 0.022  23.278  0.000    0.448    0.568
## 40 0.022 -11.335  0.000   -0.311   -0.190
## 41 0.019 -10.956  0.000   -0.257   -0.155
## 42 0.028   6.941  0.000    0.120    0.276
## 43 0.017 -13.442  0.000   -0.278   -0.184
## 44 0.015 -11.686  0.000   -0.209   -0.130
## 45 0.022   7.483  0.000    0.104    0.225
## 46 0.017  23.704  0.000    0.351    0.443
## 47 0.024  -2.685  0.007   -0.128    0.001
## 48 0.020  -1.573  0.116   -0.086    0.023
## 49 1.564  42.450  0.000   62.118   70.671
## 50 0.038  42.485  0.000    1.512    1.720
## 51 0.028  42.485  0.000    1.101    1.252
## 52 0.028  42.485  0.000    1.131    1.287
## 53 0.037  42.485  0.000    1.468    1.670
## 54 0.022  42.485  0.000    0.871    0.991
## 55 0.026  42.485  0.000    1.017    1.157
## 56 0.019  42.486  0.000    0.736    0.837
## 57 0.043  42.485  0.000    1.724    1.962
## 58 0.000      NA     NA    0.344    0.344
## 59 0.000      NA     NA    0.306    0.306
## 60 0.000      NA     NA   -0.017   -0.017
## 61 0.000      NA     NA   -0.589   -0.589
## 62 0.000      NA     NA   32.026   32.026
## 63 0.000      NA     NA   -0.453   -0.453
## 64 0.000      NA     NA    2.566    2.566
## 65 0.000      NA     NA    0.124    0.124
## 66 0.000      NA     NA   -0.236   -0.236
## 67 0.000      NA     NA   28.520   28.520
## 68 0.000      NA     NA    0.000    0.000
## 69 0.091  35.131  0.000    2.953    3.451
## 70 0.078  32.855  0.000    2.342    2.768
## 71 0.079  46.945  0.000    3.485    3.916
## 72 0.090  37.228  0.000    3.097    3.588
## 73 0.069  64.154  0.000    4.250    4.628
## 74 0.075  19.033  0.000    1.218    1.627
## 75 0.064  24.372  0.000    1.376    1.723
## 76 0.097  34.917  0.000    3.132    3.664
## 77 0.000      NA     NA    2.458    2.458
## 78 0.000      NA     NA   41.760   41.760
## 79 0.000      NA     NA    1.855    1.855
## 80 0.000      NA     NA   26.173   26.173
## 81 2.226   3.812  0.000    2.398   14.571
## 82 4.258   1.699  0.089   -4.410   18.877
## 83 0.032   2.683  0.007   -0.002    0.171
## 84 0.039   4.135  0.000    0.054    0.265
## 85 0.022   1.979  0.048   -0.017    0.103
## 86 0.025   2.049  0.040   -0.017    0.118
## 87 0.006   0.660  0.509   -0.013    0.021
## 88 0.035  -3.690  0.000   -0.225   -0.033
## 89 0.002  -0.210  0.834   -0.006    0.005
## 90 0.014  -0.447  0.655   -0.044    0.032
```

### 4.2.2 Household income

Controlled for age, sex, BMI

```
SEM_model_inc1 <- '
    level: 1
    hds_T3 ~ income_cat_T3 + a1*foodst_01_T3 + a2*foodst_02_T3 + a3*foodst_03_T3 + a4*foodst_04_T3 + a5*foodst_05_T3 + a6*foodst_06_T3 + a7*foodst_07_T3 + a8*foodst_08_T3 + age_T3 + sex_T3 + bmi_T3
    foodst_01_T3 ~ b1*income_cat_T3
    foodst_02_T3 ~ b2*income_cat_T3
    foodst_03_T3 ~ b3*income_cat_T3
    foodst_04_T3 ~ b4*income_cat_T3
    foodst_05_T3 ~ b5*income_cat_T3
    foodst_06_T3 ~ b6*income_cat_T3
    foodst_07_T3 ~ b7*income_cat_T3
    foodst_08_T3 ~ b8*income_cat_T3
    foodst_01_T3 ~~ foodst_02_T3 + foodst_03_T3 + foodst_04_T3 + foodst_05_T3 + foodst_06_T3 + foodst_07_T3 + foodst_08_T3
    foodst_02_T3 ~~ foodst_03_T3 + foodst_04_T3 + foodst_05_T3 + foodst_06_T3 + foodst_07_T3 + foodst_08_T3
    foodst_03_T3 ~~ foodst_04_T3 + foodst_05_T3 + foodst_06_T3 + foodst_07_T3 + foodst_08_T3
    foodst_04_T3 ~~ foodst_05_T3 + foodst_06_T3 + foodst_07_T3 + foodst_08_T3
    foodst_05_T3 ~~ foodst_06_T3 + foodst_07_T3 + foodst_08_T3
    foodst_06_T3 ~~ foodst_07_T3 + foodst_08_T3 
    foodst_07_T3 ~~ foodst_08_T3
    ebindfoodst_01_T3 := a1*b1 
    ebindfoodst_02_T3 := a2*b2
    ebindfoodst_03_T3 := a3*b3
    ebindfoodst_04_T3 := a4*b4
    ebindfoodst_05_T3 := a5*b5
    ebindfoodst_06_T3 := a6*b6
    ebindfoodst_07_T3 := a7*b7
    ebindfoodst_08_T3 := a8*b8

    level: 2
    hds_T3 ~ 1

'

fit_SEM_inc1 <- sem(model = SEM_model_inc1, data = subset, cluster = "country")

summary(fit_SEM_inc1)
```

```
## lavaan 0.6.16 ended normally after 114 iterations
## 
##   Estimator                                         ML
##   Optimization method                           NLMINB
##   Number of model parameters                        67
## 
##                                                   Used       Total
##   Number of observations                          3517        3725
##   Number of clusters [country]                       6            
## 
## Model Test User Model:
##                                                       
##   Test statistic                                69.849
##   Degrees of freedom                                24
##   P-value (Chi-square)                           0.000
## 
## Parameter Estimates:
## 
##   Standard errors                             Standard
##   Information                                 Observed
##   Observed information based on                Hessian
## 
## 
## Level 1 [within]:
## 
## Regressions:
##                    Estimate  Std.Err  z-value  P(>|z|)
##   hds_T3 ~                                            
##     incm_c_T3         0.564    0.101    5.586    0.000
##     fds_01_T3 (a1)    0.865    0.121    7.143    0.000
##     fds_02_T3 (a2)   -0.580    0.129   -4.491    0.000
##     fds_03_T3 (a3)    0.348    0.160    2.177    0.029
##     fds_04_T3 (a4)    0.696    0.141    4.942    0.000
##     fds_05_T3 (a5)   -0.079    0.162   -0.486    0.627
##     fds_06_T3 (a6)   -0.523    0.148   -3.543    0.000
##     fds_07_T3 (a7)   -0.103    0.173   -0.599    0.549
##     fds_08_T3 (a8)    0.358    0.104    3.450    0.001
##     age_T3            0.106    0.025    4.215    0.000
##     sex_T3            1.524    0.401    3.798    0.000
##     bmi_T3            0.068    0.027    2.531    0.011
##   foodst_01_T3 ~                                      
##     incm_c_T3 (b1)    0.000    0.015    0.021    0.983
##   foodst_02_T3 ~                                      
##     incm_c_T3 (b2)   -0.110    0.013   -8.453    0.000
##   foodst_03_T3 ~                                      
##     incm_c_T3 (b3)   -0.000    0.013   -0.001    0.999
##   foodst_04_T3 ~                                      
##     incm_c_T3 (b4)   -0.050    0.015   -3.323    0.001
##   foodst_05_T3 ~                                      
##     incm_c_T3 (b5)   -0.033    0.012   -2.844    0.004
##   foodst_06_T3 ~                                      
##     incm_c_T3 (b6)    0.066    0.013    5.246    0.000
##   foodst_07_T3 ~                                      
##     incm_c_T3 (b7)    0.014    0.011    1.290    0.197
##   foodst_08_T3 ~                                      
##     incm_c_T3 (b8)   -0.035    0.016   -2.183    0.029
## 
## Covariances:
##                    Estimate  Std.Err  z-value  P(>|z|)
##  .foodst_01_T3 ~~                                     
##    .foodst_02_T3      0.198    0.024    8.434    0.000
##    .foodst_03_T3      0.552    0.025   21.767    0.000
##    .foodst_04_T3      0.570    0.029   20.007    0.000
##    .foodst_05_T3      0.191    0.021    9.129    0.000
##    .foodst_06_T3     -0.124    0.023   -5.493    0.000
##    .foodst_07_T3     -0.095    0.019   -4.942    0.000
##    .foodst_08_T3      0.121    0.029    4.187    0.000
##  .foodst_02_T3 ~~                                     
##    .foodst_03_T3      0.076    0.020    3.753    0.000
##    .foodst_04_T3      0.094    0.023    4.090    0.000
##    .foodst_05_T3      0.052    0.018    2.919    0.004
##    .foodst_06_T3      0.069    0.019    3.605    0.000
##    .foodst_07_T3      0.058    0.016    3.527    0.000
##    .foodst_08_T3     -0.045    0.025   -1.826    0.068
##  .foodst_03_T3 ~~                                     
##    .foodst_04_T3      0.762    0.027   28.728    0.000
##    .foodst_05_T3      0.377    0.019   19.843    0.000
##    .foodst_06_T3     -0.251    0.020  -12.618    0.000
##    .foodst_07_T3     -0.195    0.017  -11.560    0.000
##    .foodst_08_T3      0.158    0.025    6.272    0.000
##  .foodst_04_T3 ~~                                     
##    .foodst_05_T3      0.498    0.022   22.623    0.000
##    .foodst_06_T3     -0.232    0.022  -10.307    0.000
##    .foodst_07_T3     -0.200    0.019  -10.446    0.000
##    .foodst_08_T3      0.186    0.029    6.476    0.000
##  .foodst_05_T3 ~~                                     
##    .foodst_06_T3     -0.227    0.018  -12.974    0.000
##    .foodst_07_T3     -0.166    0.015  -11.263    0.000
##    .foodst_08_T3      0.163    0.022    7.361    0.000
##  .foodst_06_T3 ~~                                     
##    .foodst_07_T3      0.390    0.017   22.791    0.000
##    .foodst_08_T3     -0.073    0.024   -3.065    0.002
##  .foodst_07_T3 ~~                                     
##    .foodst_08_T3     -0.035    0.020   -1.745    0.081
## 
## Intercepts:
##                    Estimate  Std.Err  z-value  P(>|z|)
##    .hds_T3            0.000                           
##    .foodst_01_T3      3.457    0.051   68.033    0.000
##    .foodst_02_T3      2.222    0.043   51.189    0.000
##    .foodst_03_T3      4.001    0.044   91.082    0.000
##    .foodst_04_T3      3.696    0.050   73.918    0.000
##    .foodst_05_T3      4.458    0.039  115.557    0.000
##    .foodst_06_T3      1.728    0.042   41.175    0.000
##    .foodst_07_T3      1.526    0.036   42.848    0.000
##    .foodst_08_T3      3.473    0.054   64.398    0.000
## 
## Variances:
##                    Estimate  Std.Err  z-value  P(>|z|)
##    .hds_T3           66.805    1.594   41.898    0.000
##    .foodst_01_T3      1.617    0.039   41.934    0.000
##    .foodst_02_T3      1.180    0.028   41.934    0.000
##    .foodst_03_T3      1.208    0.029   41.934    0.000
##    .foodst_04_T3      1.566    0.037   41.935    0.000
##    .foodst_05_T3      0.932    0.022   41.935    0.000
##    .foodst_06_T3      1.102    0.026   41.935    0.000
##    .foodst_07_T3      0.794    0.019   41.934    0.000
##    .foodst_08_T3      1.821    0.043   41.934    0.000
## 
## 
## Level 2 [country]:
## 
## Intercepts:
##                    Estimate  Std.Err  z-value  P(>|z|)
##    .hds_T3            9.563    2.195    4.357    0.000
## 
## Variances:
##                    Estimate  Std.Err  z-value  P(>|z|)
##    .hds_T3            7.190    4.232    1.699    0.089
## 
## Defined Parameters:
##                    Estimate  Std.Err  z-value  P(>|z|)
##     ebndfdst_01_T3    0.000    0.013    0.021    0.983
##     ebndfdst_02_T3    0.064    0.016    3.966    0.000
##     ebndfdst_03_T3   -0.000    0.005   -0.001    0.999
##     ebndfdst_04_T3   -0.035    0.013   -2.758    0.006
##     ebndfdst_05_T3    0.003    0.005    0.479    0.632
##     ebndfdst_06_T3   -0.034    0.012   -2.936    0.003
##     ebndfdst_07_T3   -0.001    0.003   -0.544    0.587
##     ebndfdst_08_T3   -0.013    0.007   -1.845    0.065
```

```
parameterEstimates(fit_SEM_inc1, ci=TRUE, level=.99375)
```

```
##                  lhs op           rhs block level             label    est
## 1             hds_T3  ~ income_cat_T3     1     1                    0.564
## 2             hds_T3  ~  foodst_01_T3     1     1                a1  0.865
## 3             hds_T3  ~  foodst_02_T3     1     1                a2 -0.580
## 4             hds_T3  ~  foodst_03_T3     1     1                a3  0.348
## 5             hds_T3  ~  foodst_04_T3     1     1                a4  0.696
## 6             hds_T3  ~  foodst_05_T3     1     1                a5 -0.079
## 7             hds_T3  ~  foodst_06_T3     1     1                a6 -0.523
## 8             hds_T3  ~  foodst_07_T3     1     1                a7 -0.103
## 9             hds_T3  ~  foodst_08_T3     1     1                a8  0.358
## 10            hds_T3  ~        age_T3     1     1                    0.106
## 11            hds_T3  ~        sex_T3     1     1                    1.524
## 12            hds_T3  ~        bmi_T3     1     1                    0.068
## 13      foodst_01_T3  ~ income_cat_T3     1     1                b1  0.000
## 14      foodst_02_T3  ~ income_cat_T3     1     1                b2 -0.110
## 15      foodst_03_T3  ~ income_cat_T3     1     1                b3  0.000
## 16      foodst_04_T3  ~ income_cat_T3     1     1                b4 -0.050
## 17      foodst_05_T3  ~ income_cat_T3     1     1                b5 -0.033
## 18      foodst_06_T3  ~ income_cat_T3     1     1                b6  0.066
## 19      foodst_07_T3  ~ income_cat_T3     1     1                b7  0.014
## 20      foodst_08_T3  ~ income_cat_T3     1     1                b8 -0.035
## 21      foodst_01_T3 ~~  foodst_02_T3     1     1                    0.198
## 22      foodst_01_T3 ~~  foodst_03_T3     1     1                    0.552
## 23      foodst_01_T3 ~~  foodst_04_T3     1     1                    0.570
## 24      foodst_01_T3 ~~  foodst_05_T3     1     1                    0.191
## 25      foodst_01_T3 ~~  foodst_06_T3     1     1                   -0.124
## 26      foodst_01_T3 ~~  foodst_07_T3     1     1                   -0.095
## 27      foodst_01_T3 ~~  foodst_08_T3     1     1                    0.121
## 28      foodst_02_T3 ~~  foodst_03_T3     1     1                    0.076
## 29      foodst_02_T3 ~~  foodst_04_T3     1     1                    0.094
## 30      foodst_02_T3 ~~  foodst_05_T3     1     1                    0.052
## 31      foodst_02_T3 ~~  foodst_06_T3     1     1                    0.069
## 32      foodst_02_T3 ~~  foodst_07_T3     1     1                    0.058
## 33      foodst_02_T3 ~~  foodst_08_T3     1     1                   -0.045
## 34      foodst_03_T3 ~~  foodst_04_T3     1     1                    0.762
## 35      foodst_03_T3 ~~  foodst_05_T3     1     1                    0.377
## 36      foodst_03_T3 ~~  foodst_06_T3     1     1                   -0.251
## 37      foodst_03_T3 ~~  foodst_07_T3     1     1                   -0.195
## 38      foodst_03_T3 ~~  foodst_08_T3     1     1                    0.158
## 39      foodst_04_T3 ~~  foodst_05_T3     1     1                    0.498
## 40      foodst_04_T3 ~~  foodst_06_T3     1     1                   -0.232
## 41      foodst_04_T3 ~~  foodst_07_T3     1     1                   -0.200
## 42      foodst_04_T3 ~~  foodst_08_T3     1     1                    0.186
## 43      foodst_05_T3 ~~  foodst_06_T3     1     1                   -0.227
## 44      foodst_05_T3 ~~  foodst_07_T3     1     1                   -0.166
## 45      foodst_05_T3 ~~  foodst_08_T3     1     1                    0.163
## 46      foodst_06_T3 ~~  foodst_07_T3     1     1                    0.390
## 47      foodst_06_T3 ~~  foodst_08_T3     1     1                   -0.073
## 48      foodst_07_T3 ~~  foodst_08_T3     1     1                   -0.035
## 49            hds_T3 ~~        hds_T3     1     1                   66.805
## 50      foodst_01_T3 ~~  foodst_01_T3     1     1                    1.617
## 51      foodst_02_T3 ~~  foodst_02_T3     1     1                    1.180
## 52      foodst_03_T3 ~~  foodst_03_T3     1     1                    1.208
## 53      foodst_04_T3 ~~  foodst_04_T3     1     1                    1.566
## 54      foodst_05_T3 ~~  foodst_05_T3     1     1                    0.932
## 55      foodst_06_T3 ~~  foodst_06_T3     1     1                    1.102
## 56      foodst_07_T3 ~~  foodst_07_T3     1     1                    0.794
## 57      foodst_08_T3 ~~  foodst_08_T3     1     1                    1.821
## 58     income_cat_T3 ~~ income_cat_T3     1     1                    1.987
## 59     income_cat_T3 ~~        age_T3     1     1                    0.334
## 60     income_cat_T3 ~~        sex_T3     1     1                   -0.038
## 61     income_cat_T3 ~~        bmi_T3     1     1                   -1.111
## 62            age_T3 ~~        age_T3     1     1                   32.297
## 63            age_T3 ~~        sex_T3     1     1                   -0.476
## 64            age_T3 ~~        bmi_T3     1     1                    2.594
## 65            sex_T3 ~~        sex_T3     1     1                    0.129
## 66            sex_T3 ~~        bmi_T3     1     1                   -0.244
## 67            bmi_T3 ~~        bmi_T3     1     1                   28.009
## 68            hds_T3 ~1                   1     1                    0.000
## 69      foodst_01_T3 ~1                   1     1                    3.457
## 70      foodst_02_T3 ~1                   1     1                    2.222
## 71      foodst_03_T3 ~1                   1     1                    4.001
## 72      foodst_04_T3 ~1                   1     1                    3.696
## 73      foodst_05_T3 ~1                   1     1                    4.458
## 74      foodst_06_T3 ~1                   1     1                    1.728
## 75      foodst_07_T3 ~1                   1     1                    1.526
## 76      foodst_08_T3 ~1                   1     1                    3.473
## 77     income_cat_T3 ~1                   1     1                    3.029
## 78            age_T3 ~1                   1     1                   41.738
## 79            sex_T3 ~1                   1     1                    1.848
## 80            bmi_T3 ~1                   1     1                   26.149
## 81            hds_T3 ~1                   2     2                    9.563
## 82            hds_T3 ~~        hds_T3     2     2                    7.190
## 83 ebindfoodst_01_T3 :=         a1*b1     0     0 ebindfoodst_01_T3  0.000
## 84 ebindfoodst_02_T3 :=         a2*b2     0     0 ebindfoodst_02_T3  0.064
## 85 ebindfoodst_03_T3 :=         a3*b3     0     0 ebindfoodst_03_T3  0.000
## 86 ebindfoodst_04_T3 :=         a4*b4     0     0 ebindfoodst_04_T3 -0.035
## 87 ebindfoodst_05_T3 :=         a5*b5     0     0 ebindfoodst_05_T3  0.003
## 88 ebindfoodst_06_T3 :=         a6*b6     0     0 ebindfoodst_06_T3 -0.034
## 89 ebindfoodst_07_T3 :=         a7*b7     0     0 ebindfoodst_07_T3 -0.001
## 90 ebindfoodst_08_T3 :=         a8*b8     0     0 ebindfoodst_08_T3 -0.013
##       se       z pvalue ci.lower ci.upper
## 1  0.101   5.586  0.000    0.288    0.840
## 2  0.121   7.143  0.000    0.534    1.196
## 3  0.129  -4.491  0.000   -0.933   -0.227
## 4  0.160   2.177  0.029   -0.089    0.785
## 5  0.141   4.942  0.000    0.311    1.081
## 6  0.162  -0.486  0.627   -0.522    0.364
## 7  0.148  -3.543  0.000   -0.927   -0.119
## 8  0.173  -0.599  0.549   -0.575    0.368
## 9  0.104   3.450  0.001    0.074    0.641
## 10 0.025   4.215  0.000    0.037    0.175
## 11 0.401   3.798  0.000    0.427    2.622
## 12 0.027   2.531  0.011   -0.005    0.141
## 13 0.015   0.021  0.983   -0.041    0.042
## 14 0.013  -8.453  0.000   -0.145   -0.074
## 15 0.013  -0.001  0.999   -0.036    0.036
## 16 0.015  -3.323  0.001   -0.091   -0.009
## 17 0.012  -2.844  0.004   -0.064   -0.001
## 18 0.013   5.246  0.000    0.032    0.100
## 19 0.011   1.290  0.197   -0.015    0.043
## 20 0.016  -2.183  0.029   -0.079    0.009
## 21 0.024   8.434  0.000    0.134    0.263
## 22 0.025  21.767  0.000    0.482    0.621
## 23 0.029  20.007  0.000    0.492    0.648
## 24 0.021   9.129  0.000    0.134    0.249
## 25 0.023  -5.493  0.000   -0.186   -0.062
## 26 0.019  -4.942  0.000   -0.147   -0.042
## 27 0.029   4.187  0.000    0.042    0.201
## 28 0.020   3.753  0.000    0.021    0.131
## 29 0.023   4.090  0.000    0.031    0.157
## 30 0.018   2.919  0.004    0.003    0.100
## 31 0.019   3.605  0.000    0.017    0.122
## 32 0.016   3.527  0.000    0.013    0.102
## 33 0.025  -1.826  0.068   -0.113    0.022
## 34 0.027  28.728  0.000    0.689    0.834
## 35 0.019  19.843  0.000    0.325    0.429
## 36 0.020 -12.618  0.000   -0.306   -0.197
## 37 0.017 -11.560  0.000   -0.241   -0.149
## 38 0.025   6.272  0.000    0.089    0.227
## 39 0.022  22.623  0.000    0.438    0.559
## 40 0.022 -10.307  0.000   -0.293   -0.170
## 41 0.019 -10.446  0.000   -0.252   -0.147
## 42 0.029   6.476  0.000    0.107    0.264
## 43 0.018 -12.974  0.000   -0.275   -0.179
## 44 0.015 -11.263  0.000   -0.207   -0.126
## 45 0.022   7.361  0.000    0.102    0.223
## 46 0.017  22.791  0.000    0.343    0.436
## 47 0.024  -3.065  0.002   -0.139   -0.008
## 48 0.020  -1.745  0.081   -0.091    0.020
## 49 1.594  41.898  0.000   62.445   71.165
## 50 0.039  41.934  0.000    1.512    1.722
## 51 0.028  41.934  0.000    1.103    1.257
## 52 0.029  41.934  0.000    1.129    1.287
## 53 0.037  41.935  0.000    1.464    1.668
## 54 0.022  41.935  0.000    0.871    0.993
## 55 0.026  41.935  0.000    1.030    1.174
## 56 0.019  41.934  0.000    0.743    0.846
## 57 0.043  41.934  0.000    1.703    1.940
## 58 0.000      NA     NA    1.987    1.987
## 59 0.000      NA     NA    0.334    0.334
## 60 0.000      NA     NA   -0.038   -0.038
## 61 0.000      NA     NA   -1.111   -1.111
## 62 0.000      NA     NA   32.297   32.297
## 63 0.000      NA     NA   -0.476   -0.476
## 64 0.000      NA     NA    2.594    2.594
## 65 0.000      NA     NA    0.129    0.129
## 66 0.000      NA     NA   -0.244   -0.244
## 67 0.000      NA     NA   28.009   28.009
## 68 0.000      NA     NA    0.000    0.000
## 69 0.051  68.033  0.000    3.318    3.596
## 70 0.043  51.189  0.000    2.104    2.341
## 71 0.044  91.082  0.000    3.881    4.121
## 72 0.050  73.918  0.000    3.560    3.833
## 73 0.039 115.557  0.000    4.352    4.563
## 74 0.042  41.175  0.000    1.613    1.842
## 75 0.036  42.848  0.000    1.429    1.624
## 76 0.054  64.398  0.000    3.326    3.621
## 77 0.000      NA     NA    3.029    3.029
## 78 0.000      NA     NA   41.738   41.738
## 79 0.000      NA     NA    1.848    1.848
## 80 0.000      NA     NA   26.149   26.149
## 81 2.195   4.357  0.000    3.562   15.564
## 82 4.232   1.699  0.089   -4.383   18.763
## 83 0.013   0.021  0.983   -0.036    0.036
## 84 0.016   3.966  0.000    0.020    0.108
## 85 0.005  -0.001  0.999   -0.013    0.013
## 86 0.013  -2.758  0.006   -0.069    0.000
## 87 0.005   0.479  0.632   -0.012    0.017
## 88 0.012  -2.936  0.003   -0.067   -0.002
## 89 0.003  -0.544  0.587   -0.009    0.006
## 90 0.007  -1.845  0.065   -0.031    0.006
```

### 4.2.3 Migration background

Controlled for age, sex, BMI

```
SEM_model_mig <- '
    level: 1
    hds_T3 ~ migration + a1*foodst_01_T3 + a2*foodst_02_T3 + a3*foodst_03_T3 + a4*foodst_04_T3 + a5*foodst_05_T3 + a6*foodst_06_T3 + a7*foodst_07_T3 + a8*foodst_08_T3 + age_T3 + sex_T3 + bmi_T3
    foodst_01_T3 ~ b1*migration
    foodst_02_T3 ~ b2*migration
    foodst_03_T3 ~ b3*migration
    foodst_04_T3 ~ b4*migration
    foodst_05_T3 ~ b5*migration
    foodst_06_T3 ~ b6*migration
    foodst_07_T3 ~ b7*migration
    foodst_08_T3 ~ b8*migration
    foodst_01_T3 ~~ foodst_02_T3 + foodst_03_T3 + foodst_04_T3 + foodst_05_T3 + foodst_06_T3 + foodst_07_T3 + foodst_08_T3
    foodst_02_T3 ~~ foodst_03_T3 + foodst_04_T3 + foodst_05_T3 + foodst_06_T3 + foodst_07_T3 + foodst_08_T3
    foodst_03_T3 ~~ foodst_04_T3 + foodst_05_T3 + foodst_06_T3 + foodst_07_T3 + foodst_08_T3
    foodst_04_T3 ~~ foodst_05_T3 + foodst_06_T3 + foodst_07_T3 + foodst_08_T3
    foodst_05_T3 ~~ foodst_06_T3 + foodst_07_T3 + foodst_08_T3
    foodst_06_T3 ~~ foodst_07_T3 + foodst_08_T3 
    foodst_07_T3 ~~ foodst_08_T3
    ebindfoodst_01_T3 := a1*b1 
    ebindfoodst_02_T3 := a2*b2
    ebindfoodst_03_T3 := a3*b3
    ebindfoodst_04_T3 := a4*b4
    ebindfoodst_05_T3 := a5*b5
    ebindfoodst_06_T3 := a6*b6
    ebindfoodst_07_T3 := a7*b7
    ebindfoodst_08_T3 := a8*b8

    level: 2
    hds_T3 ~ 1

'

fit_SEM_mig <- sem(model = SEM_model_mig, data = subset, cluster = "country")

summary(fit_SEM_mig)
```

```
## lavaan 0.6.16 ended normally after 120 iterations
## 
##   Estimator                                         ML
##   Optimization method                           NLMINB
##   Number of model parameters                        67
## 
##   Number of observations                          3725
##   Number of clusters [country]                       6
## 
## Model Test User Model:
##                                                       
##   Test statistic                               103.423
##   Degrees of freedom                                24
##   P-value (Chi-square)                           0.000
## 
## Parameter Estimates:
## 
##   Standard errors                             Standard
##   Information                                 Observed
##   Observed information based on                Hessian
## 
## 
## Level 1 [within]:
## 
## Regressions:
##                    Estimate  Std.Err  z-value  P(>|z|)
##   hds_T3 ~                                            
##     migration        -0.463    0.397   -1.164    0.244
##     fds_01_T3 (a1)    0.861    0.118    7.316    0.000
##     fds_02_T3 (a2)   -0.704    0.124   -5.684    0.000
##     fds_03_T3 (a3)    0.421    0.154    2.736    0.006
##     fds_04_T3 (a4)    0.622    0.136    4.567    0.000
##     fds_05_T3 (a5)   -0.135    0.159   -0.851    0.395
##     fds_06_T3 (a6)   -0.492    0.144   -3.407    0.001
##     fds_07_T3 (a7)   -0.107    0.169   -0.634    0.526
##     fds_08_T3 (a8)    0.350    0.100    3.483    0.000
##     age_T3            0.111    0.024    4.558    0.000
##     sex_T3            1.233    0.394    3.131    0.002
##     bmi_T3            0.049    0.026    1.890    0.059
##   foodst_01_T3 ~                                      
##     migration (b1)    0.091    0.062    1.471    0.141
##   foodst_02_T3 ~                                      
##     migration (b2)    0.137    0.053    2.567    0.010
##   foodst_03_T3 ~                                      
##     migration (b3)    0.018    0.053    0.335    0.738
##   foodst_04_T3 ~                                      
##     migration (b4)    0.161    0.060    2.659    0.008
##   foodst_05_T3 ~                                      
##     migration (b5)   -0.010    0.046   -0.209    0.835
##   foodst_06_T3 ~                                      
##     migration (b6)   -0.098    0.051   -1.929    0.054
##   foodst_07_T3 ~                                      
##     migration (b7)   -0.001    0.043   -0.023    0.981
##   foodst_08_T3 ~                                      
##     migration (b8)    0.097    0.066    1.471    0.141
## 
## Covariances:
##                    Estimate  Std.Err  z-value  P(>|z|)
##  .foodst_01_T3 ~~                                     
##    .foodst_02_T3      0.197    0.023    8.475    0.000
##    .foodst_03_T3      0.561    0.025   22.589    0.000
##    .foodst_04_T3      0.569    0.028   20.486    0.000
##    .foodst_05_T3      0.194    0.020    9.535    0.000
##    .foodst_06_T3     -0.128    0.022   -5.830    0.000
##    .foodst_07_T3     -0.102    0.019   -5.451    0.000
##    .foodst_08_T3      0.128    0.028    4.483    0.000
##  .foodst_02_T3 ~~                                     
##    .foodst_03_T3      0.069    0.020    3.461    0.001
##    .foodst_04_T3      0.110    0.023    4.840    0.000
##    .foodst_05_T3      0.062    0.017    3.535    0.000
##    .foodst_06_T3      0.053    0.019    2.799    0.005
##    .foodst_07_T3      0.057    0.016    3.567    0.000
##    .foodst_08_T3     -0.047    0.025   -1.906    0.057
##  .foodst_03_T3 ~~                                     
##    .foodst_04_T3      0.751    0.026   29.158    0.000
##    .foodst_05_T3      0.373    0.018   20.230    0.000
##    .foodst_06_T3     -0.246    0.019  -12.680    0.000
##    .foodst_07_T3     -0.194    0.016  -11.850    0.000
##    .foodst_08_T3      0.159    0.025    6.439    0.000
##  .foodst_04_T3 ~~                                     
##    .foodst_05_T3      0.500    0.021   23.393    0.000
##    .foodst_06_T3     -0.237    0.022  -10.835    0.000
##    .foodst_07_T3     -0.204    0.019  -10.996    0.000
##    .foodst_08_T3      0.192    0.028    6.835    0.000
##  .foodst_05_T3 ~~                                     
##    .foodst_06_T3     -0.229    0.017  -13.507    0.000
##    .foodst_07_T3     -0.166    0.014  -11.646    0.000
##    .foodst_08_T3      0.167    0.022    7.729    0.000
##  .foodst_06_T3 ~~                                     
##    .foodst_07_T3      0.394    0.017   23.762    0.000
##    .foodst_08_T3     -0.064    0.023   -2.741    0.006
##  .foodst_07_T3 ~~                                     
##    .foodst_08_T3     -0.034    0.020   -1.723    0.085
## 
## Intercepts:
##                    Estimate  Std.Err  z-value  P(>|z|)
##    .hds_T3            0.000                           
##    .foodst_01_T3      3.361    0.073   46.128    0.000
##    .foodst_02_T3      1.749    0.063   27.749    0.000
##    .foodst_03_T3      3.978    0.063   63.144    0.000
##    .foodst_04_T3      3.375    0.072   47.177    0.000
##    .foodst_05_T3      4.378    0.055   79.722    0.000
##    .foodst_06_T3      2.019    0.060   33.698    0.000
##    .foodst_07_T3      1.563    0.051   30.813    0.000
##    .foodst_08_T3      3.256    0.078   41.974    0.000
## 
## Variances:
##                    Estimate  Std.Err  z-value  P(>|z|)
##    .hds_T3           67.110    1.556   43.121    0.000
##    .foodst_01_T3      1.628    0.038   43.157    0.000
##    .foodst_02_T3      1.217    0.028   43.157    0.000
##    .foodst_03_T3      1.217    0.028   43.156    0.000
##    .foodst_04_T3      1.568    0.036   43.157    0.000
##    .foodst_05_T3      0.924    0.021   43.157    0.000
##    .foodst_06_T3      1.101    0.026   43.157    0.000
##    .foodst_07_T3      0.788    0.018   43.157    0.000
##    .foodst_08_T3      1.845    0.043   43.157    0.000
## 
## 
## Level 2 [country]:
## 
## Intercepts:
##                    Estimate  Std.Err  z-value  P(>|z|)
##    .hds_T3           12.947    2.160    5.994    0.000
## 
## Variances:
##                    Estimate  Std.Err  z-value  P(>|z|)
##    .hds_T3            7.434    4.370    1.701    0.089
## 
## Defined Parameters:
##                    Estimate  Std.Err  z-value  P(>|z|)
##     ebndfdst_01_T3    0.078    0.054    1.442    0.149
##     ebndfdst_02_T3   -0.096    0.041   -2.339    0.019
##     ebndfdst_03_T3    0.008    0.023    0.332    0.740
##     ebndfdst_04_T3    0.100    0.044    2.298    0.022
##     ebndfdst_05_T3    0.001    0.006    0.203    0.839
##     ebndfdst_06_T3    0.048    0.029    1.679    0.093
##     ebndfdst_07_T3    0.000    0.005    0.023    0.981
##     ebndfdst_08_T3    0.034    0.025    1.355    0.175
```

```
parameterEstimates(fit_SEM_mig, ci=TRUE, level=.99375)
```

```
##                  lhs op          rhs block level             label    est    se
## 1             hds_T3  ~    migration     1     1                   -0.463 0.397
## 2             hds_T3  ~ foodst_01_T3     1     1                a1  0.861 0.118
## 3             hds_T3  ~ foodst_02_T3     1     1                a2 -0.704 0.124
## 4             hds_T3  ~ foodst_03_T3     1     1                a3  0.421 0.154
## 5             hds_T3  ~ foodst_04_T3     1     1                a4  0.622 0.136
## 6             hds_T3  ~ foodst_05_T3     1     1                a5 -0.135 0.159
## 7             hds_T3  ~ foodst_06_T3     1     1                a6 -0.492 0.144
## 8             hds_T3  ~ foodst_07_T3     1     1                a7 -0.107 0.169
## 9             hds_T3  ~ foodst_08_T3     1     1                a8  0.350 0.100
## 10            hds_T3  ~       age_T3     1     1                    0.111 0.024
## 11            hds_T3  ~       sex_T3     1     1                    1.233 0.394
## 12            hds_T3  ~       bmi_T3     1     1                    0.049 0.026
## 13      foodst_01_T3  ~    migration     1     1                b1  0.091 0.062
## 14      foodst_02_T3  ~    migration     1     1                b2  0.137 0.053
## 15      foodst_03_T3  ~    migration     1     1                b3  0.018 0.053
## 16      foodst_04_T3  ~    migration     1     1                b4  0.161 0.060
## 17      foodst_05_T3  ~    migration     1     1                b5 -0.010 0.046
## 18      foodst_06_T3  ~    migration     1     1                b6 -0.098 0.051
## 19      foodst_07_T3  ~    migration     1     1                b7 -0.001 0.043
## 20      foodst_08_T3  ~    migration     1     1                b8  0.097 0.066
## 21      foodst_01_T3 ~~ foodst_02_T3     1     1                    0.197 0.023
## 22      foodst_01_T3 ~~ foodst_03_T3     1     1                    0.561 0.025
## 23      foodst_01_T3 ~~ foodst_04_T3     1     1                    0.569 0.028
## 24      foodst_01_T3 ~~ foodst_05_T3     1     1                    0.194 0.020
## 25      foodst_01_T3 ~~ foodst_06_T3     1     1                   -0.128 0.022
## 26      foodst_01_T3 ~~ foodst_07_T3     1     1                   -0.102 0.019
## 27      foodst_01_T3 ~~ foodst_08_T3     1     1                    0.128 0.028
## 28      foodst_02_T3 ~~ foodst_03_T3     1     1                    0.069 0.020
## 29      foodst_02_T3 ~~ foodst_04_T3     1     1                    0.110 0.023
## 30      foodst_02_T3 ~~ foodst_05_T3     1     1                    0.062 0.017
## 31      foodst_02_T3 ~~ foodst_06_T3     1     1                    0.053 0.019
## 32      foodst_02_T3 ~~ foodst_07_T3     1     1                    0.057 0.016
## 33      foodst_02_T3 ~~ foodst_08_T3     1     1                   -0.047 0.025
## 34      foodst_03_T3 ~~ foodst_04_T3     1     1                    0.751 0.026
## 35      foodst_03_T3 ~~ foodst_05_T3     1     1                    0.373 0.018
## 36      foodst_03_T3 ~~ foodst_06_T3     1     1                   -0.246 0.019
## 37      foodst_03_T3 ~~ foodst_07_T3     1     1                   -0.194 0.016
## 38      foodst_03_T3 ~~ foodst_08_T3     1     1                    0.159 0.025
## 39      foodst_04_T3 ~~ foodst_05_T3     1     1                    0.500 0.021
## 40      foodst_04_T3 ~~ foodst_06_T3     1     1                   -0.237 0.022
## 41      foodst_04_T3 ~~ foodst_07_T3     1     1                   -0.204 0.019
## 42      foodst_04_T3 ~~ foodst_08_T3     1     1                    0.192 0.028
## 43      foodst_05_T3 ~~ foodst_06_T3     1     1                   -0.229 0.017
## 44      foodst_05_T3 ~~ foodst_07_T3     1     1                   -0.166 0.014
## 45      foodst_05_T3 ~~ foodst_08_T3     1     1                    0.167 0.022
## 46      foodst_06_T3 ~~ foodst_07_T3     1     1                    0.394 0.017
## 47      foodst_06_T3 ~~ foodst_08_T3     1     1                   -0.064 0.023
## 48      foodst_07_T3 ~~ foodst_08_T3     1     1                   -0.034 0.020
## 49            hds_T3 ~~       hds_T3     1     1                   67.110 1.556
## 50      foodst_01_T3 ~~ foodst_01_T3     1     1                    1.628 0.038
## 51      foodst_02_T3 ~~ foodst_02_T3     1     1                    1.217 0.028
## 52      foodst_03_T3 ~~ foodst_03_T3     1     1                    1.217 0.028
## 53      foodst_04_T3 ~~ foodst_04_T3     1     1                    1.568 0.036
## 54      foodst_05_T3 ~~ foodst_05_T3     1     1                    0.924 0.021
## 55      foodst_06_T3 ~~ foodst_06_T3     1     1                    1.101 0.026
## 56      foodst_07_T3 ~~ foodst_07_T3     1     1                    0.788 0.018
## 57      foodst_08_T3 ~~ foodst_08_T3     1     1                    1.845 0.043
## 58         migration ~~    migration     1     1                    0.115 0.000
## 59         migration ~~       age_T3     1     1                    0.040 0.000
## 60         migration ~~       sex_T3     1     1                   -0.004 0.000
## 61         migration ~~       bmi_T3     1     1                    0.062 0.000
## 62            age_T3 ~~       age_T3     1     1                   32.288 0.000
## 63            age_T3 ~~       sex_T3     1     1                   -0.464 0.000
## 64            age_T3 ~~       bmi_T3     1     1                    2.609 0.000
## 65            sex_T3 ~~       sex_T3     1     1                    0.126 0.000
## 66            sex_T3 ~~       bmi_T3     1     1                   -0.235 0.000
## 67            bmi_T3 ~~       bmi_T3     1     1                   28.240 0.000
## 68            hds_T3 ~1                  1     1                    0.000 0.000
## 69      foodst_01_T3 ~1                  1     1                    3.361 0.073
## 70      foodst_02_T3 ~1                  1     1                    1.749 0.063
## 71      foodst_03_T3 ~1                  1     1                    3.978 0.063
## 72      foodst_04_T3 ~1                  1     1                    3.375 0.072
## 73      foodst_05_T3 ~1                  1     1                    4.378 0.055
## 74      foodst_06_T3 ~1                  1     1                    2.019 0.060
## 75      foodst_07_T3 ~1                  1     1                    1.563 0.051
## 76      foodst_08_T3 ~1                  1     1                    3.256 0.078
## 77         migration ~1                  1     1                    1.133 0.000
## 78            age_T3 ~1                  1     1                   41.772 0.000
## 79            sex_T3 ~1                  1     1                    1.852 0.000
## 80            bmi_T3 ~1                  1     1                   26.174 0.000
## 81            hds_T3 ~1                  2     2                   12.947 2.160
## 82            hds_T3 ~~       hds_T3     2     2                    7.434 4.370
## 83 ebindfoodst_01_T3 :=        a1*b1     0     0 ebindfoodst_01_T3  0.078 0.054
## 84 ebindfoodst_02_T3 :=        a2*b2     0     0 ebindfoodst_02_T3 -0.096 0.041
## 85 ebindfoodst_03_T3 :=        a3*b3     0     0 ebindfoodst_03_T3  0.008 0.023
## 86 ebindfoodst_04_T3 :=        a4*b4     0     0 ebindfoodst_04_T3  0.100 0.044
## 87 ebindfoodst_05_T3 :=        a5*b5     0     0 ebindfoodst_05_T3  0.001 0.006
## 88 ebindfoodst_06_T3 :=        a6*b6     0     0 ebindfoodst_06_T3  0.048 0.029
## 89 ebindfoodst_07_T3 :=        a7*b7     0     0 ebindfoodst_07_T3  0.000 0.005
## 90 ebindfoodst_08_T3 :=        a8*b8     0     0 ebindfoodst_08_T3  0.034 0.025
##          z pvalue ci.lower ci.upper
## 1   -1.164  0.244   -1.549    0.624
## 2    7.316  0.000    0.539    1.183
## 3   -5.684  0.000   -1.043   -0.366
## 4    2.736  0.006    0.000    0.843
## 5    4.567  0.000    0.250    0.994
## 6   -0.851  0.395   -0.569    0.299
## 7   -3.407  0.001   -0.886   -0.097
## 8   -0.634  0.526   -0.570    0.356
## 9    3.483  0.000    0.075    0.624
## 10   4.558  0.000    0.045    0.178
## 11   3.131  0.002    0.156    2.309
## 12   1.890  0.059   -0.022    0.119
## 13   1.471  0.141   -0.078    0.259
## 14   2.567  0.010   -0.009    0.283
## 15   0.335  0.738   -0.128    0.164
## 16   2.659  0.008   -0.005    0.326
## 17  -0.209  0.835   -0.137    0.117
## 18  -1.929  0.054   -0.236    0.041
## 19  -0.023  0.981   -0.118    0.116
## 20   1.471  0.141   -0.083    0.276
## 21   8.475  0.000    0.134    0.261
## 22  22.589  0.000    0.493    0.629
## 23  20.486  0.000    0.493    0.645
## 24   9.535  0.000    0.138    0.250
## 25  -5.830  0.000   -0.189   -0.068
## 26  -5.451  0.000   -0.153   -0.051
## 27   4.483  0.000    0.050    0.205
## 28   3.461  0.001    0.015    0.124
## 29   4.840  0.000    0.048    0.172
## 30   3.535  0.000    0.014    0.109
## 31   2.799  0.005    0.001    0.105
## 32   3.567  0.000    0.013    0.101
## 33  -1.906  0.057   -0.114    0.020
## 34  29.158  0.000    0.681    0.822
## 35  20.230  0.000    0.322    0.423
## 36 -12.680  0.000   -0.299   -0.193
## 37 -11.850  0.000   -0.239   -0.149
## 38   6.439  0.000    0.091    0.226
## 39  23.393  0.000    0.441    0.558
## 40 -10.835  0.000   -0.297   -0.177
## 41 -10.996  0.000   -0.254   -0.153
## 42   6.835  0.000    0.115    0.268
## 43 -13.507  0.000   -0.275   -0.183
## 44 -11.646  0.000   -0.205   -0.127
## 45   7.729  0.000    0.108    0.226
## 46  23.762  0.000    0.348    0.439
## 47  -2.741  0.006   -0.128    0.000
## 48  -1.723  0.085   -0.088    0.020
## 49  43.121  0.000   62.854   71.365
## 50  43.157  0.000    1.524    1.731
## 51  43.157  0.000    1.140    1.294
## 52  43.156  0.000    1.140    1.294
## 53  43.157  0.000    1.469    1.668
## 54  43.157  0.000    0.866    0.983
## 55  43.157  0.000    1.031    1.171
## 56  43.157  0.000    0.739    0.838
## 57  43.157  0.000    1.728    1.962
## 58      NA     NA    0.115    0.115
## 59      NA     NA    0.040    0.040
## 60      NA     NA   -0.004   -0.004
## 61      NA     NA    0.062    0.062
## 62      NA     NA   32.288   32.288
## 63      NA     NA   -0.464   -0.464
## 64      NA     NA    2.609    2.609
## 65      NA     NA    0.126    0.126
## 66      NA     NA   -0.235   -0.235
## 67      NA     NA   28.240   28.240
## 68      NA     NA    0.000    0.000
## 69  46.128  0.000    3.162    3.560
## 70  27.749  0.000    1.576    1.921
## 71  63.144  0.000    3.806    4.151
## 72  47.177  0.000    3.179    3.570
## 73  79.722  0.000    4.228    4.528
## 74  33.698  0.000    1.856    2.183
## 75  30.813  0.000    1.424    1.701
## 76  41.974  0.000    3.044    3.468
## 77      NA     NA    1.133    1.133
## 78      NA     NA   41.772   41.772
## 79      NA     NA    1.852    1.852
## 80      NA     NA   26.174   26.174
## 81   5.994  0.000    7.041   18.853
## 82   1.701  0.089   -4.515   19.383
## 83   1.442  0.149   -0.070    0.226
## 84  -2.339  0.019   -0.209    0.016
## 85   0.332  0.740   -0.054    0.069
## 86   2.298  0.022   -0.019    0.219
## 87   0.203  0.839   -0.016    0.019
## 88   1.679  0.093   -0.030    0.126
## 89   0.023  0.981   -0.012    0.013
## 90   1.355  0.175   -0.034    0.102
```

### 4.2.4 Unemployment in household

Controlled for age, sex, BMI

```
SEM_model_une <- '
    level: 1
    hds_T3 ~ unemploy + a1*foodst_01_T3 + a2*foodst_02_T3 + a3*foodst_03_T3 + a4*foodst_04_T3 + a5*foodst_05_T3 + a6*foodst_06_T3 + a7*foodst_07_T3 + a8*foodst_08_T3 + age_T3 + sex_T3 + bmi_T3
    foodst_01_T3 ~ b1*unemploy
    foodst_02_T3 ~ b2*unemploy
    foodst_03_T3 ~ b3*unemploy
    foodst_04_T3 ~ b4*unemploy
    foodst_05_T3 ~ b5*unemploy
    foodst_06_T3 ~ b6*unemploy
    foodst_07_T3 ~ b7*unemploy
    foodst_08_T3 ~ b8*unemploy
    foodst_01_T3 ~~ foodst_02_T3 + foodst_03_T3 + foodst_04_T3 + foodst_05_T3 + foodst_06_T3 + foodst_07_T3 + foodst_08_T3
    foodst_02_T3 ~~ foodst_03_T3 + foodst_04_T3 + foodst_05_T3 + foodst_06_T3 + foodst_07_T3 + foodst_08_T3
    foodst_03_T3 ~~ foodst_04_T3 + foodst_05_T3 + foodst_06_T3 + foodst_07_T3 + foodst_08_T3
    foodst_04_T3 ~~ foodst_05_T3 + foodst_06_T3 + foodst_07_T3 + foodst_08_T3
    foodst_05_T3 ~~ foodst_06_T3 + foodst_07_T3 + foodst_08_T3
    foodst_06_T3 ~~ foodst_07_T3 + foodst_08_T3 
    foodst_07_T3 ~~ foodst_08_T3
    ebindfoodst_01_T3 := a1*b1 
    ebindfoodst_02_T3 := a2*b2
    ebindfoodst_03_T3 := a3*b3
    ebindfoodst_04_T3 := a4*b4
    ebindfoodst_05_T3 := a5*b5
    ebindfoodst_06_T3 := a6*b6
    ebindfoodst_07_T3 := a7*b7
    ebindfoodst_08_T3 := a8*b8

    level: 2
    hds_T3 ~ 1

'

fit_SEM_une <- sem(model = SEM_model_une, data = subset, cluster = "country")

summary(fit_SEM_une)
```

```
## lavaan 0.6.16 ended normally after 141 iterations
## 
##   Estimator                                         ML
##   Optimization method                           NLMINB
##   Number of model parameters                        67
## 
##   Number of observations                          3725
##   Number of clusters [country]                       6
## 
## Model Test User Model:
##                                                       
##   Test statistic                               101.730
##   Degrees of freedom                                24
##   P-value (Chi-square)                           0.000
## 
## Parameter Estimates:
## 
##   Standard errors                             Standard
##   Information                                 Observed
##   Observed information based on                Hessian
## 
## 
## Level 1 [within]:
## 
## Regressions:
##                    Estimate  Std.Err  z-value  P(>|z|)
##   hds_T3 ~                                            
##     unemploy         -1.525    0.471   -3.240    0.001
##     fds_01_T3 (a1)    0.856    0.118    7.279    0.000
##     fds_02_T3 (a2)   -0.704    0.124   -5.687    0.000
##     fds_03_T3 (a3)    0.418    0.154    2.720    0.007
##     fds_04_T3 (a4)    0.630    0.136    4.633    0.000
##     fds_05_T3 (a5)   -0.125    0.158   -0.787    0.431
##     fds_06_T3 (a6)   -0.499    0.144   -3.463    0.001
##     fds_07_T3 (a7)   -0.113    0.169   -0.669    0.503
##     fds_08_T3 (a8)    0.343    0.100    3.423    0.001
##     age_T3            0.110    0.024    4.485    0.000
##     sex_T3            1.280    0.393    3.256    0.001
##     bmi_T3            0.055    0.026    2.119    0.034
##   foodst_01_T3 ~                                      
##     unemploy  (b1)    0.007    0.073    0.097    0.922
##   foodst_02_T3 ~                                      
##     unemploy  (b2)    0.087    0.063    1.371    0.171
##   foodst_03_T3 ~                                      
##     unemploy  (b3)    0.034    0.063    0.543    0.587
##   foodst_04_T3 ~                                      
##     unemploy  (b4)    0.167    0.072    2.326    0.020
##   foodst_05_T3 ~                                      
##     unemploy  (b5)    0.101    0.055    1.832    0.067
##   foodst_06_T3 ~                                      
##     unemploy  (b6)   -0.155    0.060   -2.581    0.010
##   foodst_07_T3 ~                                      
##     unemploy  (b7)   -0.071    0.051   -1.398    0.162
##   foodst_08_T3 ~                                      
##     unemploy  (b8)   -0.009    0.078   -0.122    0.903
## 
## Covariances:
##                    Estimate  Std.Err  z-value  P(>|z|)
##  .foodst_01_T3 ~~                                     
##    .foodst_02_T3      0.199    0.023    8.526    0.000
##    .foodst_03_T3      0.561    0.025   22.590    0.000
##    .foodst_04_T3      0.571    0.028   20.527    0.000
##    .foodst_05_T3      0.194    0.020    9.529    0.000
##    .foodst_06_T3     -0.129    0.022   -5.872    0.000
##    .foodst_07_T3     -0.102    0.019   -5.450    0.000
##    .foodst_08_T3      0.129    0.028    4.516    0.000
##  .foodst_02_T3 ~~                                     
##    .foodst_03_T3      0.069    0.020    3.460    0.001
##    .foodst_04_T3      0.111    0.023    4.895    0.000
##    .foodst_05_T3      0.061    0.017    3.484    0.000
##    .foodst_06_T3      0.053    0.019    2.775    0.006
##    .foodst_07_T3      0.058    0.016    3.596    0.000
##    .foodst_08_T3     -0.045    0.025   -1.839    0.066
##  .foodst_03_T3 ~~                                     
##    .foodst_04_T3      0.751    0.026   29.150    0.000
##    .foodst_05_T3      0.372    0.018   20.224    0.000
##    .foodst_06_T3     -0.246    0.019  -12.673    0.000
##    .foodst_07_T3     -0.194    0.016  -11.842    0.000
##    .foodst_08_T3      0.159    0.025    6.446    0.000
##  .foodst_04_T3 ~~                                     
##    .foodst_05_T3      0.498    0.021   23.335    0.000
##    .foodst_06_T3     -0.237    0.022  -10.823    0.000
##    .foodst_07_T3     -0.203    0.019  -10.947    0.000
##    .foodst_08_T3      0.194    0.028    6.898    0.000
##  .foodst_05_T3 ~~                                     
##    .foodst_06_T3     -0.228    0.017  -13.440    0.000
##    .foodst_07_T3     -0.165    0.014  -11.614    0.000
##    .foodst_08_T3      0.167    0.022    7.728    0.000
##  .foodst_06_T3 ~~                                     
##    .foodst_07_T3      0.393    0.017   23.729    0.000
##    .foodst_08_T3     -0.065    0.023   -2.792    0.005
##  .foodst_07_T3 ~~                                     
##    .foodst_08_T3     -0.034    0.020   -1.726    0.084
## 
## Intercepts:
##                    Estimate  Std.Err  z-value  P(>|z|)
##    .hds_T3            0.000                           
##    .foodst_01_T3      3.456    0.082   42.008    0.000
##    .foodst_02_T3      1.809    0.071   25.420    0.000
##    .foodst_03_T3      3.961    0.071   55.701    0.000
##    .foodst_04_T3      3.375    0.081   41.793    0.000
##    .foodst_05_T3      4.257    0.062   68.710    0.000
##    .foodst_06_T3      2.077    0.068   30.724    0.000
##    .foodst_07_T3      1.639    0.057   28.637    0.000
##    .foodst_08_T3      3.376    0.088   38.542    0.000
## 
## Variances:
##                    Estimate  Std.Err  z-value  P(>|z|)
##    .hds_T3           66.944    1.552   43.122    0.000
##    .foodst_01_T3      1.629    0.038   43.157    0.000
##    .foodst_02_T3      1.219    0.028   43.157    0.000
##    .foodst_03_T3      1.217    0.028   43.157    0.000
##    .foodst_04_T3      1.569    0.036   43.157    0.000
##    .foodst_05_T3      0.923    0.021   43.157    0.000
##    .foodst_06_T3      1.100    0.025   43.157    0.000
##    .foodst_07_T3      0.788    0.018   43.157    0.000
##    .foodst_08_T3      1.846    0.043   43.157    0.000
## 
## 
## Level 2 [country]:
## 
## Intercepts:
##                    Estimate  Std.Err  z-value  P(>|z|)
##    .hds_T3           13.922    2.163    6.438    0.000
## 
## Variances:
##                    Estimate  Std.Err  z-value  P(>|z|)
##    .hds_T3            7.435    4.370    1.701    0.089
## 
## Defined Parameters:
##                    Estimate  Std.Err  z-value  P(>|z|)
##     ebndfdst_01_T3    0.006    0.062    0.097    0.922
##     ebndfdst_02_T3   -0.061    0.046   -1.332    0.183
##     ebndfdst_03_T3    0.014    0.027    0.532    0.594
##     ebndfdst_04_T3    0.105    0.050    2.079    0.038
##     ebndfdst_05_T3   -0.013    0.017   -0.723    0.470
##     ebndfdst_06_T3    0.077    0.037    2.069    0.039
##     ebndfdst_07_T3    0.008    0.013    0.604    0.546
##     ebndfdst_08_T3   -0.003    0.027   -0.122    0.903
```

```
parameterEstimates(fit_SEM_une, ci=TRUE, level=.99375)
```

```
##                  lhs op          rhs block level             label    est    se
## 1             hds_T3  ~     unemploy     1     1                   -1.525 0.471
## 2             hds_T3  ~ foodst_01_T3     1     1                a1  0.856 0.118
## 3             hds_T3  ~ foodst_02_T3     1     1                a2 -0.704 0.124
## 4             hds_T3  ~ foodst_03_T3     1     1                a3  0.418 0.154
## 5             hds_T3  ~ foodst_04_T3     1     1                a4  0.630 0.136
## 6             hds_T3  ~ foodst_05_T3     1     1                a5 -0.125 0.158
## 7             hds_T3  ~ foodst_06_T3     1     1                a6 -0.499 0.144
## 8             hds_T3  ~ foodst_07_T3     1     1                a7 -0.113 0.169
## 9             hds_T3  ~ foodst_08_T3     1     1                a8  0.343 0.100
## 10            hds_T3  ~       age_T3     1     1                    0.110 0.024
## 11            hds_T3  ~       sex_T3     1     1                    1.280 0.393
## 12            hds_T3  ~       bmi_T3     1     1                    0.055 0.026
## 13      foodst_01_T3  ~     unemploy     1     1                b1  0.007 0.073
## 14      foodst_02_T3  ~     unemploy     1     1                b2  0.087 0.063
## 15      foodst_03_T3  ~     unemploy     1     1                b3  0.034 0.063
## 16      foodst_04_T3  ~     unemploy     1     1                b4  0.167 0.072
## 17      foodst_05_T3  ~     unemploy     1     1                b5  0.101 0.055
## 18      foodst_06_T3  ~     unemploy     1     1                b6 -0.155 0.060
## 19      foodst_07_T3  ~     unemploy     1     1                b7 -0.071 0.051
## 20      foodst_08_T3  ~     unemploy     1     1                b8 -0.009 0.078
## 21      foodst_01_T3 ~~ foodst_02_T3     1     1                    0.199 0.023
## 22      foodst_01_T3 ~~ foodst_03_T3     1     1                    0.561 0.025
## 23      foodst_01_T3 ~~ foodst_04_T3     1     1                    0.571 0.028
## 24      foodst_01_T3 ~~ foodst_05_T3     1     1                    0.194 0.020
## 25      foodst_01_T3 ~~ foodst_06_T3     1     1                   -0.129 0.022
## 26      foodst_01_T3 ~~ foodst_07_T3     1     1                   -0.102 0.019
## 27      foodst_01_T3 ~~ foodst_08_T3     1     1                    0.129 0.028
## 28      foodst_02_T3 ~~ foodst_03_T3     1     1                    0.069 0.020
## 29      foodst_02_T3 ~~ foodst_04_T3     1     1                    0.111 0.023
## 30      foodst_02_T3 ~~ foodst_05_T3     1     1                    0.061 0.017
## 31      foodst_02_T3 ~~ foodst_06_T3     1     1                    0.053 0.019
## 32      foodst_02_T3 ~~ foodst_07_T3     1     1                    0.058 0.016
## 33      foodst_02_T3 ~~ foodst_08_T3     1     1                   -0.045 0.025
## 34      foodst_03_T3 ~~ foodst_04_T3     1     1                    0.751 0.026
## 35      foodst_03_T3 ~~ foodst_05_T3     1     1                    0.372 0.018
## 36      foodst_03_T3 ~~ foodst_06_T3     1     1                   -0.246 0.019
## 37      foodst_03_T3 ~~ foodst_07_T3     1     1                   -0.194 0.016
## 38      foodst_03_T3 ~~ foodst_08_T3     1     1                    0.159 0.025
## 39      foodst_04_T3 ~~ foodst_05_T3     1     1                    0.498 0.021
## 40      foodst_04_T3 ~~ foodst_06_T3     1     1                   -0.237 0.022
## 41      foodst_04_T3 ~~ foodst_07_T3     1     1                   -0.203 0.019
## 42      foodst_04_T3 ~~ foodst_08_T3     1     1                    0.194 0.028
## 43      foodst_05_T3 ~~ foodst_06_T3     1     1                   -0.228 0.017
## 44      foodst_05_T3 ~~ foodst_07_T3     1     1                   -0.165 0.014
## 45      foodst_05_T3 ~~ foodst_08_T3     1     1                    0.167 0.022
## 46      foodst_06_T3 ~~ foodst_07_T3     1     1                    0.393 0.017
## 47      foodst_06_T3 ~~ foodst_08_T3     1     1                   -0.065 0.023
## 48      foodst_07_T3 ~~ foodst_08_T3     1     1                   -0.034 0.020
## 49            hds_T3 ~~       hds_T3     1     1                   66.944 1.552
## 50      foodst_01_T3 ~~ foodst_01_T3     1     1                    1.629 0.038
## 51      foodst_02_T3 ~~ foodst_02_T3     1     1                    1.219 0.028
## 52      foodst_03_T3 ~~ foodst_03_T3     1     1                    1.217 0.028
## 53      foodst_04_T3 ~~ foodst_04_T3     1     1                    1.569 0.036
## 54      foodst_05_T3 ~~ foodst_05_T3     1     1                    0.923 0.021
## 55      foodst_06_T3 ~~ foodst_06_T3     1     1                    1.100 0.025
## 56      foodst_07_T3 ~~ foodst_07_T3     1     1                    0.788 0.018
## 57      foodst_08_T3 ~~ foodst_08_T3     1     1                    1.846 0.043
## 58          unemploy ~~     unemploy     1     1                    0.082 0.000
## 59          unemploy ~~       age_T3     1     1                   -0.028 0.000
## 60          unemploy ~~       sex_T3     1     1                    0.002 0.000
## 61          unemploy ~~       bmi_T3     1     1                    0.120 0.000
## 62            age_T3 ~~       age_T3     1     1                   32.288 0.000
## 63            age_T3 ~~       sex_T3     1     1                   -0.464 0.000
## 64            age_T3 ~~       bmi_T3     1     1                    2.609 0.000
## 65            sex_T3 ~~       sex_T3     1     1                    0.126 0.000
## 66            sex_T3 ~~       bmi_T3     1     1                   -0.235 0.000
## 67            bmi_T3 ~~       bmi_T3     1     1                   28.240 0.000
## 68            hds_T3 ~1                  1     1                    0.000 0.000
## 69      foodst_01_T3 ~1                  1     1                    3.456 0.082
## 70      foodst_02_T3 ~1                  1     1                    1.809 0.071
## 71      foodst_03_T3 ~1                  1     1                    3.961 0.071
## 72      foodst_04_T3 ~1                  1     1                    3.375 0.081
## 73      foodst_05_T3 ~1                  1     1                    4.257 0.062
## 74      foodst_06_T3 ~1                  1     1                    2.077 0.068
## 75      foodst_07_T3 ~1                  1     1                    1.639 0.057
## 76      foodst_08_T3 ~1                  1     1                    3.376 0.088
## 77          unemploy ~1                  1     1                    1.090 0.000
## 78            age_T3 ~1                  1     1                   41.772 0.000
## 79            sex_T3 ~1                  1     1                    1.852 0.000
## 80            bmi_T3 ~1                  1     1                   26.174 0.000
## 81            hds_T3 ~1                  2     2                   13.922 2.163
## 82            hds_T3 ~~       hds_T3     2     2                    7.435 4.370
## 83 ebindfoodst_01_T3 :=        a1*b1     0     0 ebindfoodst_01_T3  0.006 0.062
## 84 ebindfoodst_02_T3 :=        a2*b2     0     0 ebindfoodst_02_T3 -0.061 0.046
## 85 ebindfoodst_03_T3 :=        a3*b3     0     0 ebindfoodst_03_T3  0.014 0.027
## 86 ebindfoodst_04_T3 :=        a4*b4     0     0 ebindfoodst_04_T3  0.105 0.050
## 87 ebindfoodst_05_T3 :=        a5*b5     0     0 ebindfoodst_05_T3 -0.013 0.017
## 88 ebindfoodst_06_T3 :=        a6*b6     0     0 ebindfoodst_06_T3  0.077 0.037
## 89 ebindfoodst_07_T3 :=        a7*b7     0     0 ebindfoodst_07_T3  0.008 0.013
## 90 ebindfoodst_08_T3 :=        a8*b8     0     0 ebindfoodst_08_T3 -0.003 0.027
##          z pvalue ci.lower ci.upper
## 1   -3.240  0.001   -2.812   -0.238
## 2    7.279  0.000    0.534    1.177
## 3   -5.687  0.000   -1.042   -0.365
## 4    2.720  0.007   -0.002    0.839
## 5    4.633  0.000    0.258    1.002
## 6   -0.787  0.431   -0.558    0.308
## 7   -3.463  0.001   -0.893   -0.105
## 8   -0.669  0.503   -0.575    0.349
## 9    3.423  0.001    0.069    0.617
## 10   4.485  0.000    0.043    0.176
## 11   3.256  0.001    0.205    2.356
## 12   2.119  0.034   -0.016    0.125
## 13   0.097  0.922   -0.192    0.207
## 14   1.371  0.171   -0.086    0.259
## 15   0.543  0.587   -0.138    0.207
## 16   2.326  0.020   -0.029    0.363
## 17   1.832  0.067   -0.050    0.251
## 18  -2.581  0.010   -0.319    0.009
## 19  -1.398  0.162   -0.210    0.068
## 20  -0.122  0.903   -0.222    0.203
## 21   8.526  0.000    0.135    0.262
## 22  22.590  0.000    0.493    0.629
## 23  20.527  0.000    0.495    0.647
## 24   9.529  0.000    0.138    0.249
## 25  -5.872  0.000   -0.190   -0.069
## 26  -5.450  0.000   -0.153   -0.051
## 27   4.516  0.000    0.051    0.207
## 28   3.460  0.001    0.015    0.124
## 29   4.895  0.000    0.049    0.173
## 30   3.484  0.000    0.013    0.108
## 31   2.775  0.006    0.001    0.105
## 32   3.596  0.000    0.014    0.102
## 33  -1.839  0.066   -0.112    0.022
## 34  29.150  0.000    0.681    0.822
## 35  20.224  0.000    0.322    0.423
## 36 -12.673  0.000   -0.299   -0.193
## 37 -11.842  0.000   -0.238   -0.149
## 38   6.446  0.000    0.092    0.227
## 39  23.335  0.000    0.440    0.556
## 40 -10.823  0.000   -0.297   -0.177
## 41 -10.947  0.000   -0.253   -0.152
## 42   6.898  0.000    0.117    0.270
## 43 -13.440  0.000   -0.274   -0.181
## 44 -11.614  0.000   -0.204   -0.126
## 45   7.728  0.000    0.108    0.226
## 46  23.729  0.000    0.348    0.438
## 47  -2.792  0.005   -0.129   -0.001
## 48  -1.726  0.084   -0.088    0.020
## 49  43.122  0.000   62.699   71.189
## 50  43.157  0.000    1.525    1.732
## 51  43.157  0.000    1.142    1.296
## 52  43.157  0.000    1.140    1.294
## 53  43.157  0.000    1.470    1.668
## 54  43.157  0.000    0.865    0.982
## 55  43.157  0.000    1.030    1.170
## 56  43.157  0.000    0.738    0.838
## 57  43.157  0.000    1.729    1.963
## 58      NA     NA    0.082    0.082
## 59      NA     NA   -0.028   -0.028
## 60      NA     NA    0.002    0.002
## 61      NA     NA    0.120    0.120
## 62      NA     NA   32.288   32.288
## 63      NA     NA   -0.464   -0.464
## 64      NA     NA    2.609    2.609
## 65      NA     NA    0.126    0.126
## 66      NA     NA   -0.235   -0.235
## 67      NA     NA   28.240   28.240
## 68      NA     NA    0.000    0.000
## 69  42.008  0.000    3.231    3.681
## 70  25.420  0.000    1.615    2.004
## 71  55.701  0.000    3.767    4.156
## 72  41.793  0.000    3.154    3.596
## 73  68.710  0.000    4.088    4.426
## 74  30.724  0.000    1.893    2.262
## 75  28.637  0.000    1.482    1.795
## 76  38.542  0.000    3.136    3.615
## 77      NA     NA    1.090    1.090
## 78      NA     NA   41.772   41.772
## 79      NA     NA    1.852    1.852
## 80      NA     NA   26.174   26.174
## 81   6.438  0.000    8.008   19.835
## 82   1.701  0.089   -4.515   19.384
## 83   0.097  0.922   -0.165    0.177
## 84  -1.332  0.183   -0.186    0.064
## 85   0.532  0.594   -0.059    0.088
## 86   2.079  0.038   -0.033    0.243
## 87  -0.723  0.470   -0.060    0.035
## 88   2.069  0.039   -0.025    0.179
## 89   0.604  0.546   -0.028    0.044
## 90  -0.122  0.903   -0.076    0.070
```

### 4.2.5 Single-parent household

Controlled for age, sex, BMI

```
SEM_model_sin <- '
    level: 1
    hds_T3 ~ singlepar + a1*foodst_01_T3 + a2*foodst_02_T3 + a3*foodst_03_T3 + a4*foodst_04_T3 + a5*foodst_05_T3 + a6*foodst_06_T3 + a7*foodst_07_T3 + a8*foodst_08_T3 + age_T3 + sex_T3 + bmi_T3
    foodst_01_T3 ~ b1*singlepar
    foodst_02_T3 ~ b2*singlepar
    foodst_03_T3 ~ b3*singlepar
    foodst_04_T3 ~ b4*singlepar
    foodst_05_T3 ~ b5*singlepar
    foodst_06_T3 ~ b6*singlepar
    foodst_07_T3 ~ b7*singlepar
    foodst_08_T3 ~ b8*singlepar
    foodst_01_T3 ~~ foodst_02_T3 + foodst_03_T3 + foodst_04_T3 + foodst_05_T3 + foodst_06_T3 + foodst_07_T3 + foodst_08_T3
    foodst_02_T3 ~~ foodst_03_T3 + foodst_04_T3 + foodst_05_T3 + foodst_06_T3 + foodst_07_T3 + foodst_08_T3
    foodst_03_T3 ~~ foodst_04_T3 + foodst_05_T3 + foodst_06_T3 + foodst_07_T3 + foodst_08_T3
    foodst_04_T3 ~~ foodst_05_T3 + foodst_06_T3 + foodst_07_T3 + foodst_08_T3
    foodst_05_T3 ~~ foodst_06_T3 + foodst_07_T3 + foodst_08_T3
    foodst_06_T3 ~~ foodst_07_T3 + foodst_08_T3 
    foodst_07_T3 ~~ foodst_08_T3
    ebindfoodst_01_T3 := a1*b1 
    ebindfoodst_02_T3 := a2*b2
    ebindfoodst_03_T3 := a3*b3
    ebindfoodst_04_T3 := a4*b4
    ebindfoodst_05_T3 := a5*b5
    ebindfoodst_06_T3 := a6*b6
    ebindfoodst_07_T3 := a7*b7
    ebindfoodst_08_T3 := a8*b8

    level: 2
    hds_T3 ~ 1

'

fit_SEM_sin <- sem(model = SEM_model_sin, data = subset, cluster = "country")

summary(fit_SEM_sin)
```

```
## lavaan 0.6.16 ended normally after 119 iterations
## 
##   Estimator                                         ML
##   Optimization method                           NLMINB
##   Number of model parameters                        67
## 
##                                                   Used       Total
##   Number of observations                          3724        3725
##   Number of clusters [country]                       6            
## 
## Model Test User Model:
##                                                       
##   Test statistic                               109.069
##   Degrees of freedom                                24
##   P-value (Chi-square)                           0.000
## 
## Parameter Estimates:
## 
##   Standard errors                             Standard
##   Information                                 Observed
##   Observed information based on                Hessian
## 
## 
## Level 1 [within]:
## 
## Regressions:
##                    Estimate  Std.Err  z-value  P(>|z|)
##   hds_T3 ~                                            
##     singlepar         0.351    0.448    0.783    0.434
##     fds_01_T3 (a1)    0.859    0.118    7.301    0.000
##     fds_02_T3 (a2)   -0.705    0.124   -5.694    0.000
##     fds_03_T3 (a3)    0.418    0.154    2.715    0.007
##     fds_04_T3 (a4)    0.627    0.136    4.603    0.000
##     fds_05_T3 (a5)   -0.120    0.159   -0.756    0.450
##     fds_06_T3 (a6)   -0.492    0.144   -3.406    0.001
##     fds_07_T3 (a7)   -0.099    0.169   -0.582    0.560
##     fds_08_T3 (a8)    0.344    0.100    3.430    0.001
##     age_T3            0.111    0.024    4.528    0.000
##     sex_T3            1.229    0.395    3.115    0.002
##     bmi_T3            0.048    0.026    1.854    0.064
##   foodst_01_T3 ~                                      
##     singlepar (b1)   -0.274    0.069   -3.973    0.000
##   foodst_02_T3 ~                                      
##     singlepar (b2)   -0.092    0.060   -1.532    0.126
##   foodst_03_T3 ~                                      
##     singlepar (b3)   -0.254    0.060   -4.268    0.000
##   foodst_04_T3 ~                                      
##     singlepar (b4)   -0.347    0.068   -5.138    0.000
##   foodst_05_T3 ~                                      
##     singlepar (b5)   -0.209    0.052   -4.021    0.000
##   foodst_06_T3 ~                                      
##     singlepar (b6)    0.275    0.057    4.853    0.000
##   foodst_07_T3 ~                                      
##     singlepar (b7)    0.099    0.048    2.066    0.039
##   foodst_08_T3 ~                                      
##     singlepar (b8)    0.135    0.074    1.838    0.066
## 
## Covariances:
##                    Estimate  Std.Err  z-value  P(>|z|)
##  .foodst_01_T3 ~~                                     
##    .foodst_02_T3      0.197    0.023    8.469    0.000
##    .foodst_03_T3      0.554    0.025   22.438    0.000
##    .foodst_04_T3      0.563    0.028   20.346    0.000
##    .foodst_05_T3      0.189    0.020    9.328    0.000
##    .foodst_06_T3     -0.122    0.022   -5.572    0.000
##    .foodst_07_T3     -0.098    0.019   -5.286    0.000
##    .foodst_08_T3      0.132    0.028    4.655    0.000
##  .foodst_02_T3 ~~                                     
##    .foodst_03_T3      0.068    0.020    3.405    0.001
##    .foodst_04_T3      0.109    0.023    4.827    0.000
##    .foodst_05_T3      0.059    0.017    3.421    0.001
##    .foodst_06_T3      0.054    0.019    2.829    0.005
##    .foodst_07_T3      0.057    0.016    3.577    0.000
##    .foodst_08_T3     -0.044    0.025   -1.805    0.071
##  .foodst_03_T3 ~~                                     
##    .foodst_04_T3      0.744    0.026   29.051    0.000
##    .foodst_05_T3      0.368    0.018   20.098    0.000
##    .foodst_06_T3     -0.239    0.019  -12.410    0.000
##    .foodst_07_T3     -0.190    0.016  -11.686    0.000
##    .foodst_08_T3      0.163    0.025    6.605    0.000
##  .foodst_04_T3 ~~                                     
##    .foodst_05_T3      0.493    0.021   23.208    0.000
##    .foodst_06_T3     -0.230    0.022  -10.587    0.000
##    .foodst_07_T3     -0.201    0.018  -10.890    0.000
##    .foodst_08_T3      0.198    0.028    7.064    0.000
##  .foodst_05_T3 ~~                                     
##    .foodst_06_T3     -0.224    0.017  -13.277    0.000
##    .foodst_07_T3     -0.165    0.014  -11.587    0.000
##    .foodst_08_T3      0.169    0.022    7.854    0.000
##  .foodst_06_T3 ~~                                     
##    .foodst_07_T3      0.391    0.016   23.682    0.000
##    .foodst_08_T3     -0.069    0.023   -2.949    0.003
##  .foodst_07_T3 ~~                                     
##    .foodst_08_T3     -0.036    0.020   -1.809    0.070
## 
## Intercepts:
##                    Estimate  Std.Err  z-value  P(>|z|)
##    .hds_T3            0.000                           
##    .foodst_01_T3      3.766    0.079   47.803    0.000
##    .foodst_02_T3      2.004    0.068   29.345    0.000
##    .foodst_03_T3      4.279    0.068   62.873    0.000
##    .foodst_04_T3      3.940    0.077   50.971    0.000
##    .foodst_05_T3      4.597    0.059   77.441    0.000
##    .foodst_06_T3      1.605    0.065   24.798    0.000
##    .foodst_07_T3      1.452    0.055   26.462    0.000
##    .foodst_08_T3      3.217    0.084   38.279    0.000
## 
## Variances:
##                    Estimate  Std.Err  z-value  P(>|z|)
##    .hds_T3           67.081    1.556   43.116    0.000
##    .foodst_01_T3      1.622    0.038   43.151    0.000
##    .foodst_02_T3      1.219    0.028   43.151    0.000
##    .foodst_03_T3      1.210    0.028   43.151    0.000
##    .foodst_04_T3      1.561    0.036   43.151    0.000
##    .foodst_05_T3      0.920    0.021   43.151    0.000
##    .foodst_06_T3      1.095    0.025   43.151    0.000
##    .foodst_07_T3      0.786    0.018   43.151    0.000
##    .foodst_08_T3      1.845    0.043   43.151    0.000
## 
## 
## Level 2 [country]:
## 
## Intercepts:
##                    Estimate  Std.Err  z-value  P(>|z|)
##    .hds_T3           12.042    2.162    5.569    0.000
## 
## Variances:
##                    Estimate  Std.Err  z-value  P(>|z|)
##    .hds_T3            7.405    4.354    1.701    0.089
## 
## Defined Parameters:
##                    Estimate  Std.Err  z-value  P(>|z|)
##     ebndfdst_01_T3   -0.235    0.067   -3.490    0.000
##     ebndfdst_02_T3    0.065    0.044    1.479    0.139
##     ebndfdst_03_T3   -0.106    0.046   -2.290    0.022
##     ebndfdst_04_T3   -0.218    0.064   -3.428    0.001
##     ebndfdst_05_T3    0.025    0.034    0.743    0.458
##     ebndfdst_06_T3   -0.135    0.049   -2.788    0.005
##     ebndfdst_07_T3   -0.010    0.017   -0.560    0.575
##     ebndfdst_08_T3    0.047    0.029    1.620    0.105
```

```
parameterEstimates(fit_SEM_sin, ci=TRUE, level=.99375)
```

```
##                  lhs op          rhs block level             label    est    se
## 1             hds_T3  ~    singlepar     1     1                    0.351 0.448
## 2             hds_T3  ~ foodst_01_T3     1     1                a1  0.859 0.118
## 3             hds_T3  ~ foodst_02_T3     1     1                a2 -0.705 0.124
## 4             hds_T3  ~ foodst_03_T3     1     1                a3  0.418 0.154
## 5             hds_T3  ~ foodst_04_T3     1     1                a4  0.627 0.136
## 6             hds_T3  ~ foodst_05_T3     1     1                a5 -0.120 0.159
## 7             hds_T3  ~ foodst_06_T3     1     1                a6 -0.492 0.144
## 8             hds_T3  ~ foodst_07_T3     1     1                a7 -0.099 0.169
## 9             hds_T3  ~ foodst_08_T3     1     1                a8  0.344 0.100
## 10            hds_T3  ~       age_T3     1     1                    0.111 0.024
## 11            hds_T3  ~       sex_T3     1     1                    1.229 0.395
## 12            hds_T3  ~       bmi_T3     1     1                    0.048 0.026
## 13      foodst_01_T3  ~    singlepar     1     1                b1 -0.274 0.069
## 14      foodst_02_T3  ~    singlepar     1     1                b2 -0.092 0.060
## 15      foodst_03_T3  ~    singlepar     1     1                b3 -0.254 0.060
## 16      foodst_04_T3  ~    singlepar     1     1                b4 -0.347 0.068
## 17      foodst_05_T3  ~    singlepar     1     1                b5 -0.209 0.052
## 18      foodst_06_T3  ~    singlepar     1     1                b6  0.275 0.057
## 19      foodst_07_T3  ~    singlepar     1     1                b7  0.099 0.048
## 20      foodst_08_T3  ~    singlepar     1     1                b8  0.135 0.074
## 21      foodst_01_T3 ~~ foodst_02_T3     1     1                    0.197 0.023
## 22      foodst_01_T3 ~~ foodst_03_T3     1     1                    0.554 0.025
## 23      foodst_01_T3 ~~ foodst_04_T3     1     1                    0.563 0.028
## 24      foodst_01_T3 ~~ foodst_05_T3     1     1                    0.189 0.020
## 25      foodst_01_T3 ~~ foodst_06_T3     1     1                   -0.122 0.022
## 26      foodst_01_T3 ~~ foodst_07_T3     1     1                   -0.098 0.019
## 27      foodst_01_T3 ~~ foodst_08_T3     1     1                    0.132 0.028
## 28      foodst_02_T3 ~~ foodst_03_T3     1     1                    0.068 0.020
## 29      foodst_02_T3 ~~ foodst_04_T3     1     1                    0.109 0.023
## 30      foodst_02_T3 ~~ foodst_05_T3     1     1                    0.059 0.017
## 31      foodst_02_T3 ~~ foodst_06_T3     1     1                    0.054 0.019
## 32      foodst_02_T3 ~~ foodst_07_T3     1     1                    0.057 0.016
## 33      foodst_02_T3 ~~ foodst_08_T3     1     1                   -0.044 0.025
## 34      foodst_03_T3 ~~ foodst_04_T3     1     1                    0.744 0.026
## 35      foodst_03_T3 ~~ foodst_05_T3     1     1                    0.368 0.018
## 36      foodst_03_T3 ~~ foodst_06_T3     1     1                   -0.239 0.019
## 37      foodst_03_T3 ~~ foodst_07_T3     1     1                   -0.190 0.016
## 38      foodst_03_T3 ~~ foodst_08_T3     1     1                    0.163 0.025
## 39      foodst_04_T3 ~~ foodst_05_T3     1     1                    0.493 0.021
## 40      foodst_04_T3 ~~ foodst_06_T3     1     1                   -0.230 0.022
## 41      foodst_04_T3 ~~ foodst_07_T3     1     1                   -0.201 0.018
## 42      foodst_04_T3 ~~ foodst_08_T3     1     1                    0.198 0.028
## 43      foodst_05_T3 ~~ foodst_06_T3     1     1                   -0.224 0.017
## 44      foodst_05_T3 ~~ foodst_07_T3     1     1                   -0.165 0.014
## 45      foodst_05_T3 ~~ foodst_08_T3     1     1                    0.169 0.022
## 46      foodst_06_T3 ~~ foodst_07_T3     1     1                    0.391 0.016
## 47      foodst_06_T3 ~~ foodst_08_T3     1     1                   -0.069 0.023
## 48      foodst_07_T3 ~~ foodst_08_T3     1     1                   -0.036 0.020
## 49            hds_T3 ~~       hds_T3     1     1                   67.081 1.556
## 50      foodst_01_T3 ~~ foodst_01_T3     1     1                    1.622 0.038
## 51      foodst_02_T3 ~~ foodst_02_T3     1     1                    1.219 0.028
## 52      foodst_03_T3 ~~ foodst_03_T3     1     1                    1.210 0.028
## 53      foodst_04_T3 ~~ foodst_04_T3     1     1                    1.561 0.036
## 54      foodst_05_T3 ~~ foodst_05_T3     1     1                    0.920 0.021
## 55      foodst_06_T3 ~~ foodst_06_T3     1     1                    1.095 0.025
## 56      foodst_07_T3 ~~ foodst_07_T3     1     1                    0.786 0.018
## 57      foodst_08_T3 ~~ foodst_08_T3     1     1                    1.845 0.043
## 58         singlepar ~~    singlepar     1     1                    0.092 0.000
## 59         singlepar ~~       age_T3     1     1                    0.009 0.000
## 60         singlepar ~~       sex_T3     1     1                    0.008 0.000
## 61         singlepar ~~       bmi_T3     1     1                   -0.057 0.000
## 62            age_T3 ~~       age_T3     1     1                   32.296 0.000
## 63            age_T3 ~~       sex_T3     1     1                   -0.464 0.000
## 64            age_T3 ~~       bmi_T3     1     1                    2.611 0.000
## 65            sex_T3 ~~       sex_T3     1     1                    0.126 0.000
## 66            sex_T3 ~~       bmi_T3     1     1                   -0.235 0.000
## 67            bmi_T3 ~~       bmi_T3     1     1                   28.244 0.000
## 68            hds_T3 ~1                  1     1                    0.000 0.000
## 69      foodst_01_T3 ~1                  1     1                    3.766 0.079
## 70      foodst_02_T3 ~1                  1     1                    2.004 0.068
## 71      foodst_03_T3 ~1                  1     1                    4.279 0.068
## 72      foodst_04_T3 ~1                  1     1                    3.940 0.077
## 73      foodst_05_T3 ~1                  1     1                    4.597 0.059
## 74      foodst_06_T3 ~1                  1     1                    1.605 0.065
## 75      foodst_07_T3 ~1                  1     1                    1.452 0.055
## 76      foodst_08_T3 ~1                  1     1                    3.217 0.084
## 77         singlepar ~1                  1     1                    1.102 0.000
## 78            age_T3 ~1                  1     1                   41.771 0.000
## 79            sex_T3 ~1                  1     1                    1.852 0.000
## 80            bmi_T3 ~1                  1     1                   26.175 0.000
## 81            hds_T3 ~1                  2     2                   12.042 2.162
## 82            hds_T3 ~~       hds_T3     2     2                    7.405 4.354
## 83 ebindfoodst_01_T3 :=        a1*b1     0     0 ebindfoodst_01_T3 -0.235 0.067
## 84 ebindfoodst_02_T3 :=        a2*b2     0     0 ebindfoodst_02_T3  0.065 0.044
## 85 ebindfoodst_03_T3 :=        a3*b3     0     0 ebindfoodst_03_T3 -0.106 0.046
## 86 ebindfoodst_04_T3 :=        a4*b4     0     0 ebindfoodst_04_T3 -0.218 0.064
## 87 ebindfoodst_05_T3 :=        a5*b5     0     0 ebindfoodst_05_T3  0.025 0.034
## 88 ebindfoodst_06_T3 :=        a6*b6     0     0 ebindfoodst_06_T3 -0.135 0.049
## 89 ebindfoodst_07_T3 :=        a7*b7     0     0 ebindfoodst_07_T3 -0.010 0.017
## 90 ebindfoodst_08_T3 :=        a8*b8     0     0 ebindfoodst_08_T3  0.047 0.029
##          z pvalue ci.lower ci.upper
## 1    0.783  0.434   -0.875    1.577
## 2    7.301  0.000    0.538    1.181
## 3   -5.694  0.000   -1.044   -0.367
## 4    2.715  0.007   -0.003    0.840
## 5    4.603  0.000    0.254    0.999
## 6   -0.756  0.450   -0.554    0.314
## 7   -3.406  0.001   -0.887   -0.097
## 8   -0.582  0.560   -0.562    0.365
## 9    3.430  0.001    0.070    0.619
## 10   4.528  0.000    0.044    0.178
## 11   3.115  0.002    0.150    2.308
## 12   1.854  0.064   -0.023    0.118
## 13  -3.973  0.000   -0.462   -0.085
## 14  -1.532  0.126   -0.255    0.072
## 15  -4.268  0.000   -0.417   -0.091
## 16  -5.138  0.000   -0.532   -0.163
## 17  -4.021  0.000   -0.351   -0.067
## 18   4.853  0.000    0.120    0.430
## 19   2.066  0.039   -0.032    0.230
## 20   1.838  0.066   -0.066    0.336
## 21   8.469  0.000    0.133    0.261
## 22  22.438  0.000    0.486    0.621
## 23  20.346  0.000    0.487    0.638
## 24   9.328  0.000    0.134    0.244
## 25  -5.572  0.000   -0.182   -0.062
## 26  -5.286  0.000   -0.149   -0.047
## 27   4.655  0.000    0.055    0.210
## 28   3.405  0.001    0.013    0.122
## 29   4.827  0.000    0.047    0.171
## 30   3.421  0.001    0.012    0.107
## 31   2.829  0.005    0.002    0.105
## 32   3.577  0.000    0.014    0.101
## 33  -1.805  0.071   -0.112    0.023
## 34  29.051  0.000    0.674    0.814
## 35  20.098  0.000    0.318    0.418
## 36 -12.410  0.000   -0.292   -0.186
## 37 -11.686  0.000   -0.235   -0.146
## 38   6.605  0.000    0.095    0.230
## 39  23.208  0.000    0.435    0.551
## 40 -10.587  0.000   -0.290   -0.171
## 41 -10.890  0.000   -0.251   -0.150
## 42   7.064  0.000    0.121    0.274
## 43 -13.277  0.000   -0.270   -0.178
## 44 -11.587  0.000   -0.203   -0.126
## 45   7.854  0.000    0.110    0.228
## 46  23.682  0.000    0.346    0.436
## 47  -2.949  0.003   -0.133   -0.005
## 48  -1.809  0.070   -0.090    0.018
## 49  43.116  0.000   62.827   71.335
## 50  43.151  0.000    1.519    1.724
## 51  43.151  0.000    1.141    1.296
## 52  43.151  0.000    1.134    1.287
## 53  43.151  0.000    1.462    1.660
## 54  43.151  0.000    0.862    0.979
## 55  43.151  0.000    1.026    1.164
## 56  43.151  0.000    0.736    0.836
## 57  43.151  0.000    1.728    1.962
## 58      NA     NA    0.092    0.092
## 59      NA     NA    0.009    0.009
## 60      NA     NA    0.008    0.008
## 61      NA     NA   -0.057   -0.057
## 62      NA     NA   32.296   32.296
## 63      NA     NA   -0.464   -0.464
## 64      NA     NA    2.611    2.611
## 65      NA     NA    0.126    0.126
## 66      NA     NA   -0.235   -0.235
## 67      NA     NA   28.244   28.244
## 68      NA     NA    0.000    0.000
## 69  47.803  0.000    3.551    3.982
## 70  29.345  0.000    1.817    2.191
## 71  62.873  0.000    4.093    4.465
## 72  50.971  0.000    3.728    4.151
## 73  77.441  0.000    4.434    4.759
## 74  24.798  0.000    1.428    1.783
## 75  26.462  0.000    1.302    1.602
## 76  38.279  0.000    2.987    3.446
## 77      NA     NA    1.102    1.102
## 78      NA     NA   41.771   41.771
## 79      NA     NA    1.852    1.852
## 80      NA     NA   26.175   26.175
## 81   5.569  0.000    6.129   17.954
## 82   1.701  0.089   -4.500   19.311
## 83  -3.490  0.000   -0.420   -0.051
## 84   1.479  0.139   -0.055    0.184
## 85  -2.290  0.022   -0.233    0.021
## 86  -3.428  0.001   -0.391   -0.044
## 87   0.743  0.458   -0.067    0.117
## 88  -2.788  0.005   -0.268   -0.003
## 89  -0.560  0.575   -0.058    0.038
## 90   1.620  0.105   -0.032    0.125
```
